# Integrated System Built for Small-Molecule Semiconductors via High-Throughput Approaches

**DOI:** 10.1021/jacs.3c03271

**Published:** 2023-07-19

**Authors:** Jianchang Wu, Jiyun Zhang, Manman Hu, Patrick Reiser, Luca Torresi, Pascal Friederich, Leopold Lahn, Olga Kasian, Dirk M. Guldi, M. Eugenia Pérez-Ojeda, Anastasia Barabash, Juan S. Rocha-Ortiz, Yicheng Zhao, Zhiqiang Xie, Junsheng Luo, Yunuo Wang, Sang Il Seok, Jens A. Hauch, Christoph J. Brabec

**Affiliations:** † Forschungszentrum Jülich GmbH, Helmholtz-Institute Erlangen−Nürnberg (HI-ERN), Immerwahrstraße 2, 91058 Erlangen, Germany; ‡ Faculty of Engineering, Department of Material Science, Materials for Electronics and Energy Technology (i-MEET), Friedrich-Alexander-Universität Erlangen−Nürnberg (FAU), Martensstrasse 7, 91058 Erlangen, Germany; § Department of Energy Engineering, School of Energy and Chemical Engineering, Ulsan National Institute of Science and Technology (UNIST), 50 UNIST-gil, Eonyang-eup, Ulju-gun, Ulsan 44919, Korea; ∥ Institute of Nanotechnology, Karlsruhe Institute of Technology (KIT), Hermann-von-Helmholtz-Platz 1, 76344 Eggenstein-Leopoldshafen, Germany; ⊥ Institute of Theoretical Informatics, Karlsruhe Institute of Technology (KIT), Ham Fasanengarten 5, 76131 Karlsruhe, Germany; # Helmholtz-Zentrum Berlin GmbH, Helmholtz Institut Erlangen−Nürnberg, Cauerstraße 1, 91058 Erlangen, Germany; ∇ Department of Chemistry and Pharmacy & Interdisciplinary Center of Molecular Materials (ICMM), Friedrich-Alexander-Universität Erlangen−Nürnberg (FAU), 91058 Erlangen, Germany; ○ Department of Chemistry and Pharmacy, Friedrich-Alexander-Universität Erlangen−Nürnberg (FAU), Nikolaus-Fiebiger-Straße 10, 91058 Erlangen, Germany; ◆ University of Electronic Science and Technology of China, School of Electronic Science and Engineering, State Key Laboratory of Electronic Thin Films and Integrated Devices, 611731 Chengdu, P. R. China

## Abstract

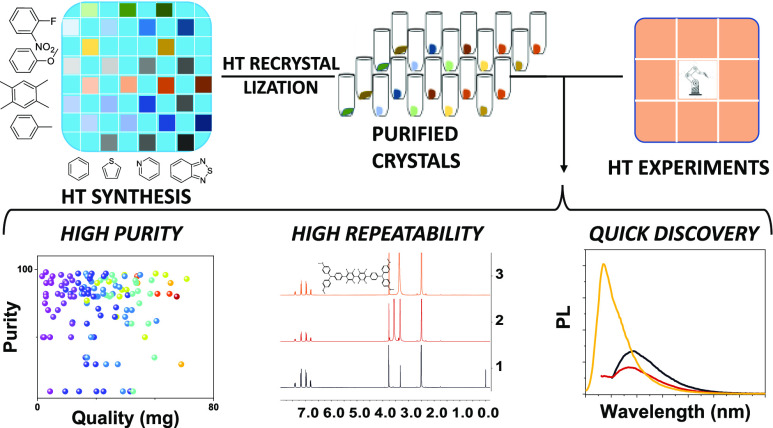

High-throughput synthesis of solution-processable structurally variable small-molecule semiconductors is both an opportunity and a challenge. A large number of diverse molecules provide a possibility for quick material discovery and machine learning based on experimental data. However, the diversity of the molecular structure leads to the complexity of molecular properties, such as solubility, polarity, and crystallinity, which poses great challenges to solution processing and purification. Here, we first report an integrated system for the high-throughput synthesis, purification, and characterization of molecules with a large variety. Based on the principle “Like dissolves like,” we combine theoretical calculations and a robotic platform to accelerate the purification of those molecules. With this platform, a material library containing 125 molecules and their optical-electronic properties was built within a timeframe of weeks. More importantly, the high repeatability of recrystallization we design is a reliable approach to further upgrading and industrial production.

## Introduction

Conjugated small molecules have been used in a vast number of optoelectronic applications, such as organic light-emitting diodes (OLEDs),^1^ organic solar cells (OSCs),^2,3^ perovskite solar cells (PSCs),^4,5^ organic thin-film transistors (OTFTs),^6^ and optical imaging^7^ owing to their unique optical-electronic properties, batch-to-batch reproducibility, and solution processability. Breakthroughs in materials science always rely on two key factors, namely, establishing broadly applicable quantitative structure–property relationships and discovering materials beyond current rules. Implicit in the former is, for example, the relationship between open-circuit voltage (*V*_oc_) and the energy difference between the highest occupied molecular orbital (HOMO) of the electron donor and the lowest unoccupied molecular orbital (LUMO) of the electron acceptor.^8,9^ This per se provides the direction for the design of conjugated molecules and is essential for promoting the performance of organic solar cells beyond 10%. The latter has opened up in the form of conducting polymers’ new realms for investigation.^10,11^ Achieving these two goals calls for a material library containing solution-processable structurally variable small-molecule semiconductors and the corresponding properties. Literally, millions of molecules could be synthesized via coupling of different commercial building blocks. Moreover, their optoelectronic properties are tunable by means of employing proper building blocks. This suggests that creating a material library containing a large number of diverse molecules is feasible. With this material library at hand, we should be able to construct more widely applicable structure–property relationships. These enable not only the rational design and synthesis of tailor-made materials but also the potential discovery of materials with unexpected properties. However, the individual synthetic procedure, purification, and properties evaluation process of a large number of materials are time-consuming and labor-intense.

Solid-phase synthesis offers an efficient way to realize high-throughput (HT) organic syntheses.^12^ Bäuerle et al. prepared, for example, a library of 256 oligo(3-arylthiophene)s by utilizing solid-phase synthesis.^13,14^ It is based on the stepwise addition of functional building blocks to a growing conjugated oligothiophene chain, which was covalently bound to a solid resin particle. It provided a procedure whereby reagents and byproducts were simply removed by filtration, and recrystallization of any intermediates was eliminated. Other types of π-conjugated oligomers have been reported and synthesized by a solid-phase approach, including oligo-(dialkylfluorene)s,^15^ oligo-(triarylamine)s,^16^ and alternating cooligomers thereof.^17^ Also notable is that Burke et al. developed a new type of catch-and-release purification protocol for *N*-methyliminodiacetic acid (MIDA)-boronate-containing intermediates.^18,19^ MIDA boronates absorbed on silica gel were purified by switching the eluent, thanks to their binary elution properties. It reflects a general and automated purification process based on an automated iterative MIDA-boronates assembly. However, both solid-phase syntheses and MIDA-boronates-based platforms serve to simplify the purification of the intermediates in the multistep synthesis. Still, the final products require to be processed and purified by conventional means, which are unsuitable for one-step reactions. Meanwhile, starting from the commercial building blocks, one-step coupling reactions afford up to millions of molecules with different properties that are promising for semiconductor device applications.

Thus oriented, here, we present a semiautomatic platform to conduct the HT one-step synthesis and purification by combining a microwave reactor, vacuum manifold, sample concentrator, and robot system. Based on the principle “Like dissolves like,” we combine theoretical calculations and a robotic platform to accelerate the purification of those molecules. With this platform, more than 20 products were purified in parallel. In total, more than 100 small-molecule organic semiconductors with large variations in molecular structure have been synthesized, purified, and characterized. All of them were analyzed by ^1^H NMR to confirm their purity. Their optoelectronic properties were characterized by means of absorption, photoluminescence, and cyclic voltammetry using HT characterization equipment.

## Results and Discussion

**Figure 1:**
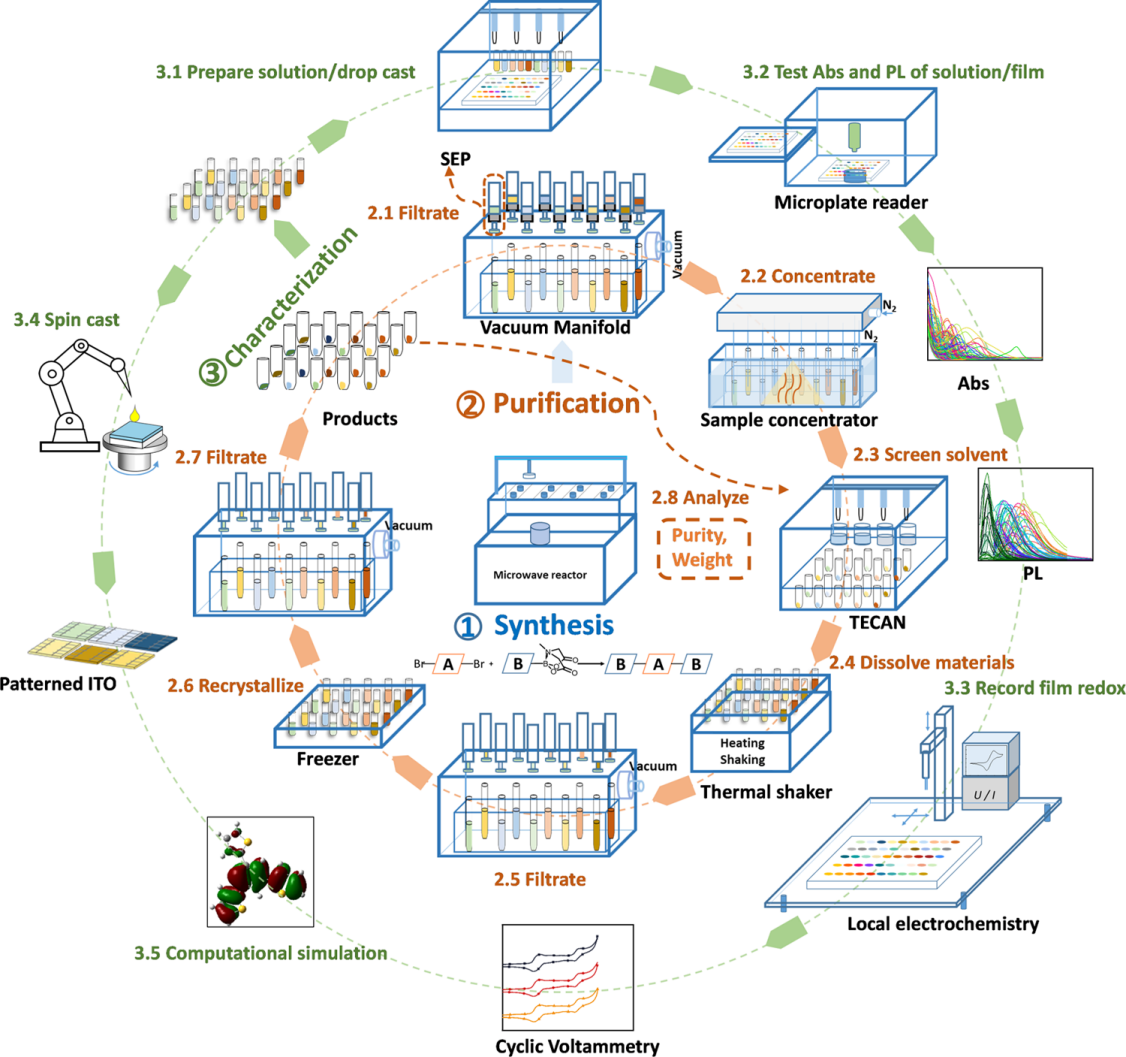
Workflow of the high-throughput synthesis, purification, and characterization. (1) HT synthesis with microwave reactor; (2) HT purification: filtrate, concentrate, screen the solvent, dissolve the materials, filtrate, recrystallize, filtrate, and analyze; (3) HT characterization: prepare the solution/drop-cast, test UV–vis absorption and PL in solution/film, record films redox, spin cast, and computational simulation.

The entire workflow consists (Figure 1) of three high-throughput (HT) processes: HT synthesis, HT purification, and HT characterization. In the first step, the reactions are carried out in a microwave reactor. This allows to shorten the reaction time from 1 day to 1 h and also automatically runs up to 48 reactions one by one. After full optimization, the Suzuki–Miyaura coupling reaction is completed within 30 min with over 95% yield. This per se greatly reduces the difficulty of subsequent purification. For the second step of purification, we developed a two-step purification process that combines filtration and recrystallization. Filtration, on the one hand, helps to remove the reagents from the reaction mixture, such as the catalyst, acidic raw materials, and inorganic salts. Recrystallization, on the other hand, is used for further purification.

In detail, the organic layers of the reaction mixtures are transferred into the vacuum manifold, which speeds up the filtration process. They are then filtered through homemade solid-phase extraction (SEP) (step 2.1), which consists of activated carbon, basic alumina, and silica gel, followed by solvent removal using a sample concentrator (step 2.2).

Then, recrystallization is implemented through solvent screening by a liquid handling and automation system (TECAN, step 2.3), dissolving the material (step 2.4), filtrating (step 2.5, removing insoluble impurities by cotton), and freezing (step 2.6). After freezing for a few hours, crystals precipitate from the solution and are collected by removing the solvent through filtration (step 2.7, filter: cotton). Throughout this process, some materials may be in the form of crystals from recrystallization (step 2.7) or filter cake from filtration (steps 2.1 and 2.5), especially for materials with low solubility. We name them product-C and product-F, respectively. Sample purity and sample weight are decisive in terms of another recrystallization step, which will be discussed in detail in the next part (HT synthesis and purification). The purified materials are characterized by means of absorption, photoluminescence (PL), and cyclic voltammetry (CV). Absorption and PL are tested both in solution and thin films. We use an in-house-developed HT electrochemical setup based on local electrochemistry for the CV characterization of films formed by drop-casting through the TECAN robotic system. In addition, an in-house spin-coating robot allows depositing thin films on patterned indium tin oxide (ITO) substrates, which allows us to conduct electronic characterizations. Theoretical properties, including molecular dipole, energy, and energy levels, are obtained by computational simulation.

### Reaction Optimization Based on Reference Materials **Re1** and **Re2**

We used a microwave-assisted Suzuki coupling reaction to synthesize the material library. To first optimize one cycle of HT synthesis, HT purification, and HT characterization, we subjected methoxy-substituted triphenylamines MIDA ester (**B1**) and phenyl MIDA ester (**B7**) to Suzuki coupling with 2,5-dibromothiophene (**A34**) and tris(4-bromophenyl)amine (**A30**), respectively.

**Figure 2:**
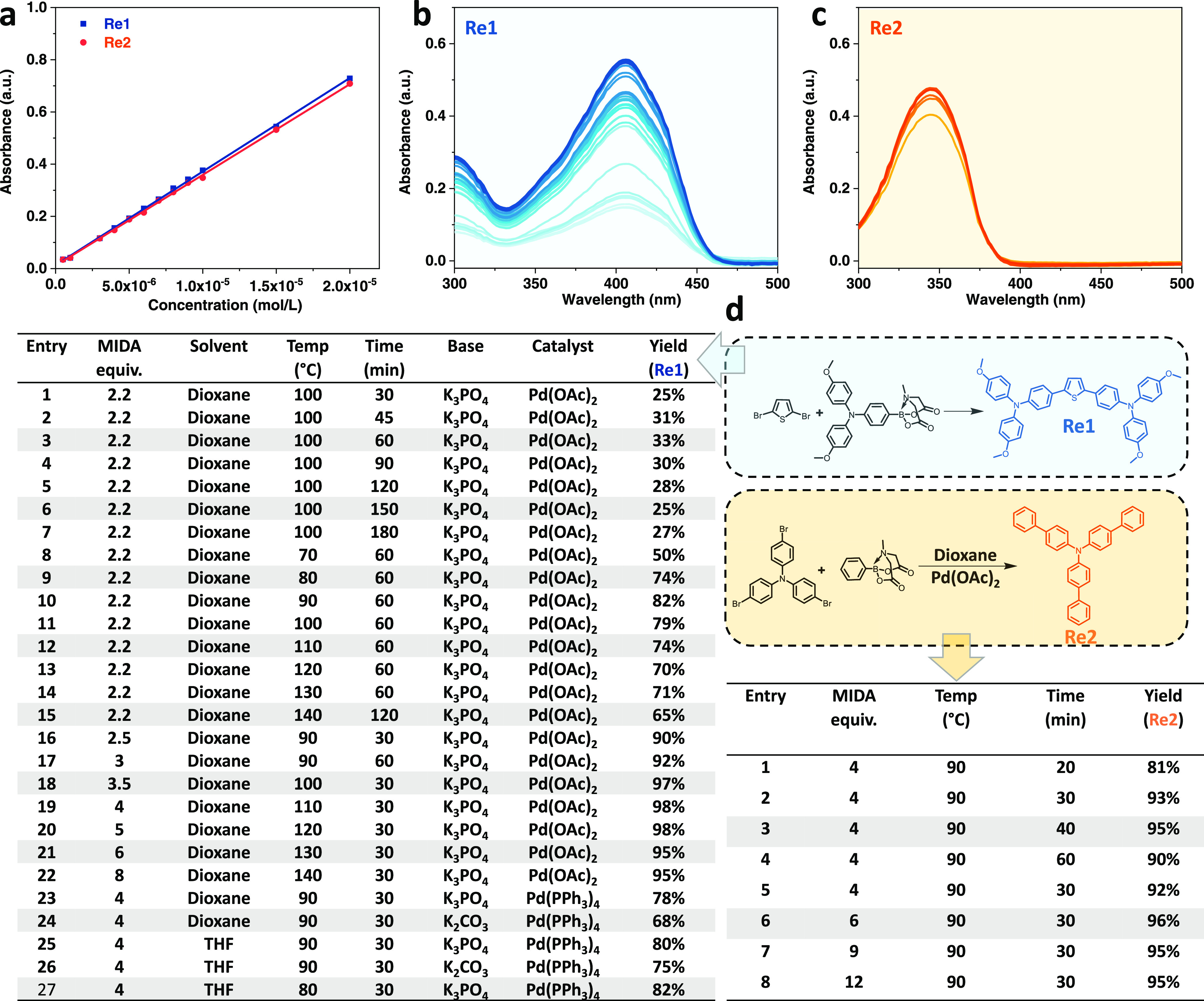
Optimization of reaction conditions based on reference materials. (a) Absorbance–concentration curve for **Re1** and **Re2** in the range from 5 × 10^–7^ to 2 × 10^–5^ M. (b, c) UV–vis absorption of the diluted reaction solution at different conditions. (d) Chemical structures and synthetic routes of **Re1** and **Re2**.

We termed the products **Re1** (2,5-bis(4,4′-bis(methoxyphenyl)aminophen-4″-yl)-thiophene) and **Re2** (tris(4-biphenylyl)amine), respectively (Figure 2d). Here, we mainly focus on p-type organic semiconductors, which is the case for most **Re1** and **Re2**. Differences in the structure of the two reference materials allow determining a general approach to process most molecules in the material library.

For HT monitoring of the yield of the reactions, we established the corresponding concentration–absorbance dependencies based on **Re1**(20) and **Re2** (Figures 2a and S1) before the syntheses. The **Re1** and **Re2** yields under various reaction conditions were calculated from the ratio of the absorbances at 404 nm for **Re1** or 344 nm for **Re2** of the diluted solutions to the theoretical value of 100% yield (Figure 2b,c). It is worth mentioning that the remaining nonreacted starting materials have almost no absorption after 300 nm and thus no influence on the yield calculation. All absorption data were obtained by a HT Microplate Reader. To obtain the optimal reaction protocol, the reaction time, temperature, solvent, catalyst, base, and stoichiometric ratio (equiv) of the reaction were systematically optimized. We found that the equiv of boronic monomer relative to bromine has a significant impact on the reactions, in general, and the reaction time as well as the reaction yield, in particular. An excess of boronic monomer (2 equiv for each reaction site) speeds up the reaction and increases the yield. Therefore, we assigned the amount of boronic acid to 2 equiv for each reactive position. It is noted that the excess boronic monomers do not interfere with the purification. Any residual boronic monomers are effectively absorbed by the silica gel and alumina, and, thus, finally removed from the reaction medium through simple filtration. The optimum yields of **Re1** and **Re2** using a methyliminodiacetic boronic acid ester (MIDA) reactant are 98 and 96%, respectively. Such values ease the purification process. Notable is the limited scale for building blocks with MIDA compared to that of boronic acid or boronic ester. To extend our material library, several building blocks with boronic acid or boronic ester groups were selected for synthesis. After optimization, the **Re1** yield using boronic acid was boosted to 95%, similar to that of the MIDA reactant (Table S1 and Figure S2).

Recrystallization is an effective way to purify organic semiconductors, especially those with short or no alkyl chains at all. Compared to chromatographic methods, recrystallization is a better way to purify a large number of compounds in parallel. It is also much easier to obtain purified products by simple filtration. Recrystallization is rarely employed in high-throughput synthesis, especially in cases where molecules with large structural variations are expected.^14,18,21,22^ This is due to the time-consuming process of finding an optimal solvent. The solvent–antisolvent method and the solvent ratio are hard to transfer to molecules with different structures. Here, we adapted a high-throughput platform that integrated solution distribution, heating, stirring, and cooling to screen the optimal solvent composition for recrystallization. Nine good solvents (chlorobenzene, toluene, 1,4-dioxane, tetrahydrofuran (THF), chloroform (CHCl_3_), ethyl acetate, *N*,*N*-dimethylformamide, and acetonitrile) and three poor solvents (methanol (MeOH), hexane, and cyclohexane) were selected for the preliminary solvent screening. Among them, THF and CHCl_3_ were chosen as good solvents, and MeOH and hexane as antisolvents. The ratio of good solvent to poor solvent varied from 3:1 to 1:3. The detailed solvent ratio and the corresponding yields are collected in Tables S2 and S3 and Figures S3 and S4.

The whole process was also recorded in Video 1. Figures S3 and S4 clearly show that the mix-solvent of THF/hexane (1:2) gives the highest isolated yield of 81% for **Re1** and 92% for **Re2**. For the mixed solvent, when the concentration of antisolvent is more than 75%, that is, 1:3, it is difficult to dissolve the materials. Therefore, in the subsequent high-throughput purification of the 125 molecules, we took THF/hexane (1:2) as the preferred solvent for recrystallizing molecules with triphenylamine at the periphery and THF/MeOH (1:2) for those with either triphenylamine in the core or without triphenylamine.

### High-Throughput Syntheses and Purification

**Figure 3:**
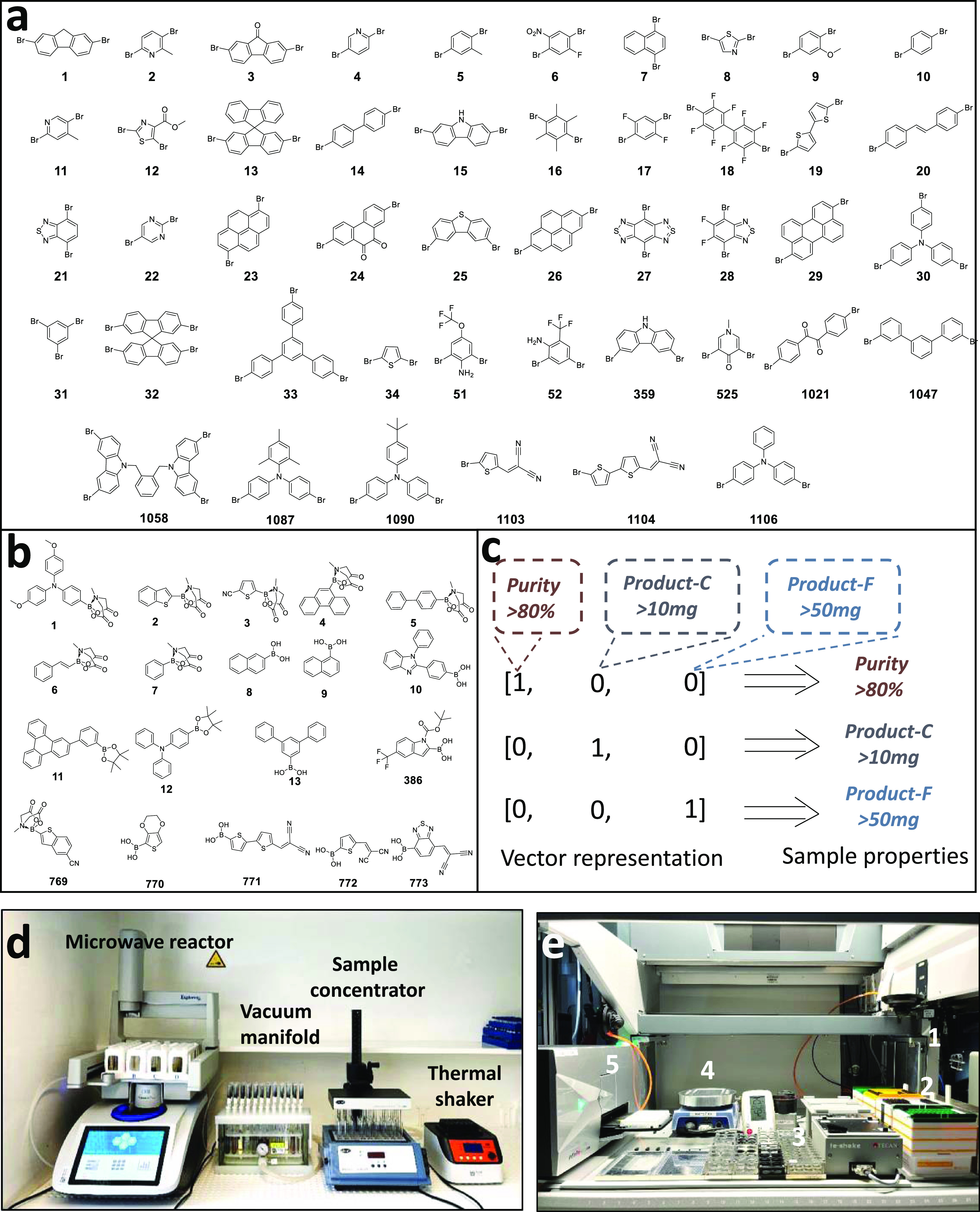
High-throughput synthesis and purification. (a) Brominated blocks (monomer A); (b) boronic acid blocks (monomer B) used in the coupling reactions; (c) encoding sample properties; (d) photograph of high-throughput setups; and (e) TECAN system: (1) robot arm with four pipettes; (2) pipet tips; (3) stock solutions; (4) hot plate; and (5) microplate reader.

Having optimized the entire process chain for **Re1** and **Re2**, we targeted the semiautomated synthesis and purification of a wide range of conjugated small molecules. To this end, 45 bis, tris, or tetrafunctional bromides and 19 boronic acid derivatives (Figure 3) were chosen from thousands of commercial monomers. A general selection criterion is to diversify the synthesized molecules, including their conjugation length, heteroatom species, solubility, band gap, dipole, energy, etc. We named the products according to the combinations of the serial numbers of two reactants, A*x*B*y* (Figure S5). The detailed chemical structures and their names are summarized in Figure S6. Similar to the semiautomated **Re1** and **Re2** syntheses, all of the generated products were purified using filtration and recrystallization.

During the synthesis process, we did not directly combine monomers **A** and **B**. Instead, we first combined **A1**–**A30** with **B1**–**B2** to obtain 54 molecules. Among them, six **A** monomers were randomly removed during the synthesis of **A*x*B2**. **B1**, 4-methoxytriphenylaniline, is a commonly used functional group in organic semiconductors, especially in perovskite solar cells, due to the excellent electron-donating properties and solubility. On the other hand, **B2**, benzo[*b*]thiophene, has a distinctly different structure from **B1**, which aligns with our monomer selection principle of diversifying molecules. Based on the material properties of the first 54 molecules and by incorporating chemical intuition, we predicted new molecular structures and synthesized them, resulting in samples numbered 54–101. In the second iteration, machine learning was applied to build the model and predict new molecules (molecules 102–125). This will be reported in a separate paper. As a result, starting from molecule 102, the sequence numbers of monomers are not consecutive and very large.

Following optimization of the reaction conditions, only a few byproducts were present in the reaction mixture. However, the large variation in solubility and crystallinity due to the drastic differences in molecular structure render purification very challenging. After a single purification cycle, some products needed further purification due to low purity or sample weight. At this point, we created purification encodings (Figure 3c), which comprised sample property encoding and further action encoding. Taking the sample property encoding as an example, the width of the encoding corresponded to the number of sample properties, while bits represented properties that were present in a material, similarly to one-hot encoding.^23^ The purification encodings, on the other hand, can be adjusted according to the actual requirements. As a matter of fact, this is beneficial for an upgrade to a fully automatic purification. In the current context, attempts to optimize the isolated yields were kept moderate. Instead, we focus on obtaining products with suitable purity and sample weight as quickly as possible, which is typical in most medical chemistry screening campaigns.^22^ Therefore, we focused on the following properties: purity, weight of crystals recrystallized from the filtered solution (Product-C), and weight of products obtained from the filter cake (Product-F). This was meant to determine how to proceed from here on. For example, [0,1,0] represents a product that features low purity (<80%) after purification and moderate weight (>10 mg). But it is free of any filter cake. In other words, the product has high solubility but low crystallinity. Then, the product was recrystallized again, but with more antisolvent. The corresponding encoding is [0,0,1,0,0] (Table S4). All sample properties, together with an analysis of the corresponding factors, are listed in Table S4. Photographs of the purification process are shown in Figures S7–S9.

### HT Characterization Studies

**Figure 4:**
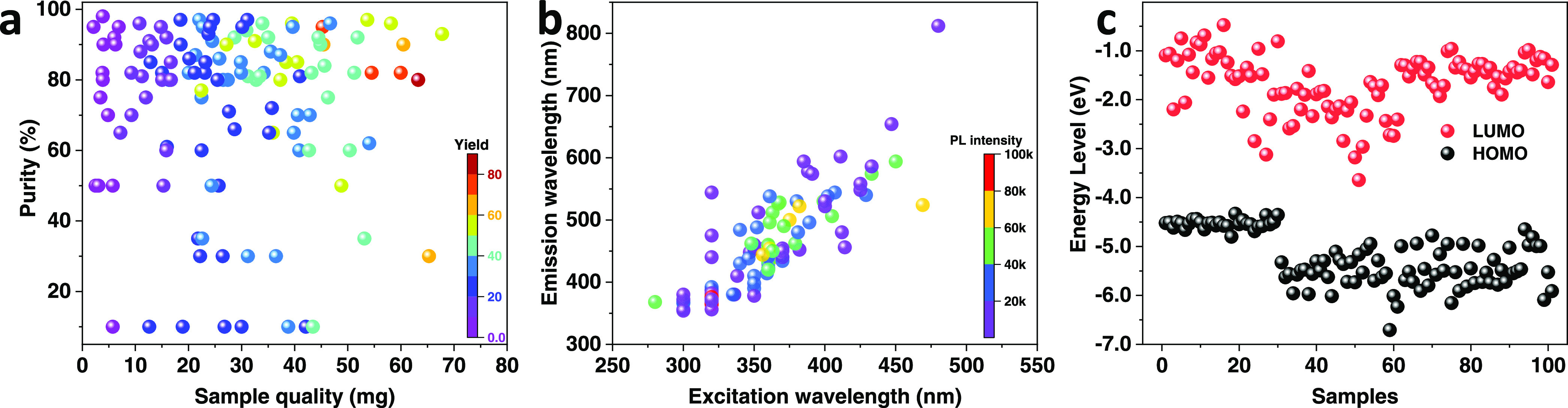
Properties of the synthesized compounds: (a) Sample weight, yield, and purity; (b) UV–vis absorption and PL; and (c) HOMO and LUMO based on DFT calculation.

All samples were characterized by ^1^H NMR, ultraviolet–visible (UV–vis) absorption, and PL spectroscopy. Some of the products were also characterized with matrix-assisted laser desorption/ionization time of flight mass spectrometry (MALDI-TOF MS) and CV to confirm the purity of the products and the value of highest occupied molecular orbital (HOMO) based on density functional theory (DFT) calculation, respectively. Figure 4 summarizes all products’ sample weight, yield, and purity. Among 125 reactions, we obtained 111 samples in more than 10 mg and 90 samples with a purity of more than 70%, sufficient to meet the requirements for functional discovery assays. In Figure 4b, several molecule solutions were diluted owing to the too high UV–vis absorbance (>1) or PL value (exceeding the machine detection threshold). The PL intensity–concentration curve of those molecules can be seen in Figures S11 and S12.

One advantage of our HT synthesis is being able to generate a big material library quickly. Combining our HT characterization equipment, including the TECAN system with absorption and PL spectroscopy,^24,25^ next to an automated spin-coating station, we gathered all relevant data in a matter of several days. Figure 4 illustrates that the variations in absorption, PL, and energy levels are rather large. Such an asset is tremendous as it allows selecting from any library depending on the specific needs. In addition, building a material library opens the ways and means to discover materials with unexpected properties. We take, for example, **A31B3** with a simple chemical structure. It exhibits a dual emission (Figure S13), which violates Kasha’s rule.^26^ In other words, in **A31B3**, photons may be emitted from higher-lying excited states, not just the lowest excited state. Most reported EX-De materials are, however, either nanoparticles or metal complexes.^27−29^ Our material library provides a compelling strategy to construct purely organic dual-emission materials. Of great importance is the fact that all data are obtained under the same conditions. This fact facilitates widely applicable structure–property relationships.

### Batch-to-Batch Reproducibility of the Platform

**Figure 5:**
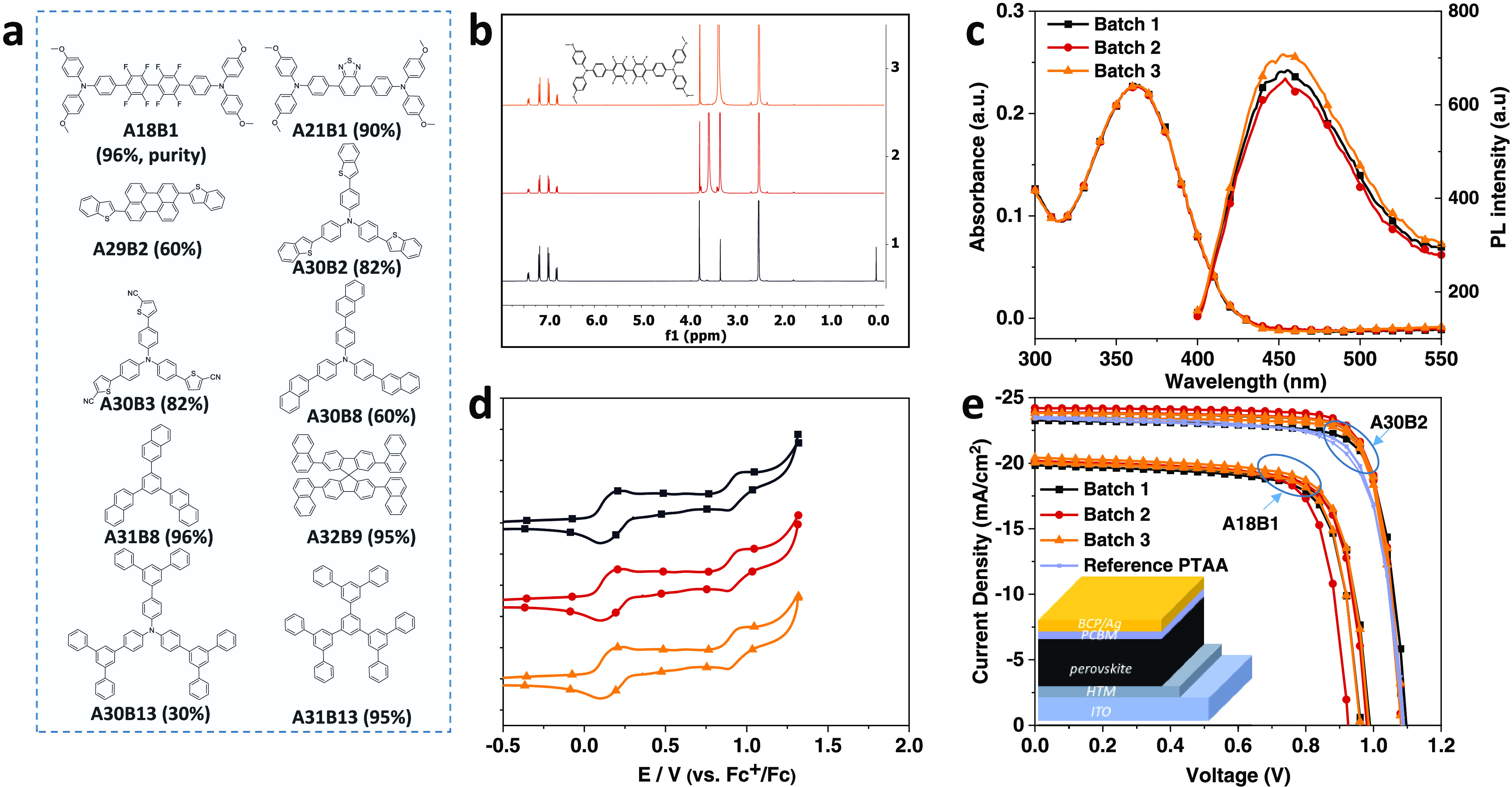
Characterization of batch-to-batch repeatability. (a) Chemical structures of 10 molecules with different purities selected for batch-to-batch repeatability characterization. Characterization of **A18B1** in three batches. (b) 1H NMR, (c) UV–vis and PL spectra (in DMF), (d) cyclic voltammetry, and (e) performance of **A18B1** and **A30B2** in perovskite solar cells.

For our synthesis method, some of the products are inevitably of low purity (<80%). They may contain reaction products, self-coupling products, partial substitutions, or other byproducts. Therefore, batch-to-batch reproducibility is critical. Reproducibility was verified by selecting and repeatedly synthesizing 10 representative products (Figure 5a). Emphasis was placed on different purities before comparing their properties. We considered **A18B1** as an example to prove the repeatability. Figure 5b–e shows the spectra of three different batches of **A18B1**. ^1^H NMR shows almost the same peaks in different batches. Only subtle differences were noted with respect to the solvent peak (3.5 ppm, MeOH) in the second batch. MALDI-TOF MS and thin layer chromatography further confirm the same purity and components in each batch (Figures S17–S38). Even for samples of low purity (<80%), TLCs reveal the same spots regardless of the respective batch. In other words, each batch contains the same byproducts or impurities. When organic semiconductors are applied in devices, trace impurities may cause significant changes in energy levels, PL quenching, etc. As such, the device performance might vary largely. Those changes are believed to be the main reason for semiconductor device degradation. Importantly, we are talking about impurities that might not be detectable by NMR or MALDI-TOF MS. Therefore, those materials in different batches were also characterized by absorption, PL, and CV, which show negligible alterations (Figure 5c,d). Data for the other nine materials are collected in Figures S17–S42. In order to understand the electronic quality and reproducibility of the molecules with respect to the electronic quality, we have decided to manually test various batches of two selected molecules as the hole transporting layer in perovskite solar cells (Figure 5e). Among them, the devices based on **A18B1** have moderate performance, while **A30B2** shows excellent performance, with an open-circuit voltage (*V*_oc_) of 1.08 V, a short-circuit current density (*J*_sc_) of 24.19 mA/cm^2^, a fill factor (FF) of 0.81, and PCE of 21.04%, even surpassing the commonly used poly(triarylamine) (PTAA). What is more noteworthy is their repeatability. Three batches of **A30B2** have almost consistent device performance, including *J*_sc_, *V*_oc_, and FF, while the performance of **A18B1** devices only fluctuated slightly in the second batch, which may be caused by the remaining small amount of solvent (Figure 5b). This greatly demonstrates the usability of our materials and the reliability of our HT synthesis platform.

The ideal situation is to develop a complete workflow that will couple automatic synthesis with automatic device processing that provides feedback loops. Such feedback loops will recommend new structures based on device results, and allow for a closed optimization of the material to the target criteria of the solar cell. However, such a workflow is far beyond the current paper. Here, we only developed the first part of the workflow, HT synthesis, which is the foundation for the entire process to proceed.

## Conclusions

In summary, we present a highly reliable semiautomatic platform to synthesize and characterize solution-processable small-molecule semiconductors. Within several weeks, it can build a material library containing 125 conjugated small molecules and establish their optical and electronic properties, encompassing absorption, PL, and electrochemistry property, both in theory and experiment. This will accelerate not only the establishment of structure–property relationships based on big data obtained under the same conditions but also the discovery of novel molecules. As the material library becomes available to scientists in various fields, more quantitative structure–property relationships and materials with unexpected properties will be constructed and discovered.

## Supporting Information Available

Synthesis and characterization details; computational details; all synthesized chemical structures; ^1^H NMR, MALDI-TOF MS, cyclic voltammetry, UV–vis data; and figures of high-throughput setups (PDF)
Summary of the data of UV–vis absorption and PL (Table 6) (XLSX)
Summary of sample properties (Table 5) (XLSX)
Commercial monomer A library (Table 7) (XLSX)
Commercial monomer B library (Table 8) (XLSX)
Theoretical properties of computational prescreened molecules (Table 9) (XLSX)
Theoretical properties of synthesized molecules (Table 10) (XLSX)
Recrystallization (Video 1) (MP4)
