# Supplementary material for: Integrated System Built for Small-Molecule Semiconductors via High-Throughput Approaches
Source: J Am Chem Soc. 2023 Jul 19;145(30):16517–25. doi: 10.1021/jacs.3c03271 (PMC10401720; doi:10.1021/jacs.3c03271)
Supplement: Supplementary file 1 — ja3c03271_si_001.pdf [file ja3c03271_si_001.pdf]

Supplementary Materials for

An integrated system built for small-molecule semiconductors via high-throughput approaches

Jianchang Wu[1,2], Jiyun Zhang[1,2], Manman Hu[3], Patrick Reiser[4], Luca Torresi[4], Pascal Friederich[4,5], Leopold Lahn[2,6], Olga Kasian[2,6], Dirk M. Guldi[7], M. Eugenia Pérez-Ojeda[8], Anastasia Barabash[1,2], Juan S. Rocha-Ortiz[2], Yicheng Zhao[1,2,9], Zhiqiang Xie[2], Junsheng Luo[2,9], Yunuo Wang[2], Sang Il Seok[3], Jens A. Hauch[1,2], and Christoph J. Brabec[1,2]**

[1]Forschungszentrum Jülich GmbH, Helmholtz-Institute Erlangen-Nürnberg (HI-ERN), Immerwahrstraße 2, 91058 Erlangen, Germany

[2]Friedrich-Alexander-University Erlangen-Nuremberg (FAU), Faculty of Engineering, Department of Material Science, Materials for Electronics and Energy Technology (i-MEET), Martensstrasse 7, 91058 Erlangen, Germany

[3]Department of Energy Engineering, School of Energy and Chemical Engineering, Ulsan National Institute of Science and Technology (UNIST), 50 UNIST-gil, Eonyang-eup, Ulju-gun, Ulsan 44919, Korea

[4]Institute of Nanotechnology, Karlsruhe Institute of Technology (KIT), Hermann-von-Helmholtz-Platz 1, 76344 Eggenstein-Leopoldshafen, Germany

[5]Institute of Theoretical Informatics, Karlsruhe Institute of Technology (KIT), Ham Fasanengarten 5, 76131 Karlsruhe, Germany

[6]Helmholtz-Zentrum Berlin GmbH, Helmholtz Institut Erlangen-Nürnberg, Cauerstraße 1, 91058 Erlangen, Germany

[7]Department of Chemistry and Pharmacy & Interdisciplinary Center of Molecular Materials (ICMM), Friedrich-Alexander-Universität Erlangen-Nürnberg (FAU), Erlangen 91058, Germany

[8]Department of Chemistry and Pharmacy, Friedrich-AlexanderUniversity Erlangen-Nuremberg (FAU), Nikolaus-Fiebiger-Straße 10, 91058 Erlangen, Germany

[9]University of Electronic Science and Technology of China, School of Electronic Science and Engineering, State Key Laboratory of Electronic Thin Films and Integrated Devices, 611731 Chengdu, P. R. China.

Corresponding Authors

E-mail: jianchang.wu@fau.de; christoph.brabec@fau.de

Table of Content

1. Materials and Methods……………………………………………………………………………………..S3

2. Reaction optimization based on reference materials Re1 and Re2……………………….……...S5

3. High-throughput syntheses and purification……………………………………………………...S12

4. High throughput setups and corresponding data…………………………………………………...S28

5. Batch-to-batch reproducibility of the platform………………………………………….……..…..S32

6. NMR of all synthesized molecules………………………………………………………………...S58

7. References……………………………………………………………………………………………..S185

1. Materials and Methods

1.1. Materials

Reagents and solvents for organic synthesis were purchased from commercial suppliers (Fluorochem, Sigma-Aldrich, BLD pharm, TCI Europe, Apollo, Alfa Aesar) and used with no further purification unless otherwise noted. Thin layer chromatography (TLC) plates were purchased from Sigma-Aldrich.

Chemicals for perovskite solar cells: Formamidinium iodide (FAI) and methylamonium bromide (MABr) were purchased from Greatcell Solar Materials. lead iodide (PbI_2) and Lead bromide ($PbBr_2$) were purchased from TCI chemicals. Cesium iodide (CsI), *N,N*-Dimethylformamide (DMF), isopropyl alcohol, dimethyl sulfoxide (DMSO), and chlorobenzene were purchased from Sigma-Aldrich.

1.2. General Experimental Information

The amount of theoretical products is 0.2 mmol for most reactions, except for some products with too large or too small molecular weight. We set the weights of theoretical products around 100 mg. The stock solution-1 dissolving $Pd(OAc)_2$ (0.01 mmol) and SPhos (0.02 mmol) in dioxane, stock solution-2 of 3 M K_3PO_4 in H_2O, were firstly prepared and degassed with N_2. Then monomer A (0.2 mmol, 1.0 equiv.) and monomer B (0.8 mmol, 4.0 equiv.) were added into vials and sealed under the inert atmosphere. 3 ml stock solution-1 and 0.5 ml stock solution-2 were injected into vials with a syringe through the septum. Before the start of the heating program in the microwave reactor, we set a pre-stirring for 5 min to dissolve the starting materials. Then the reaction temperature was raised to 90 °C and kept for 30 min. Here, we set the power of the microwave limit not to exceed 100 W. The reaction mixtures were cooled to room temperature. Aqueous layers were removed manually with pipettes. The remaining organic layers were transferred into the SEP tube of the vacuum manifold. After filtering, the SEP was rinsed 5 times with 2 ml THF. The solvents were removed by a sample concentrator, and the residues were purified by recrystallization with optimized mixed solvent and recrystallization again if needed by encoding.

Computational screening. The workflow of reaction, structure, and electronic property prediction was set up using FireWorks[(1)]. The reaction product from the coupling of boronic acid with brominated compounds has been generated using RDKit[(2)]. Subsequently, a conformer search was carried out using CREST[(3)], starting from an initial geometry guess of RDKit, which was

optimized with a semiempirical tight-binding model in GNF-xTB[4]. DFT single-point energies were calculated for the optimized conformers with the TURBOMOLE[5] package using the def2-SVP basis set and the B3LYP functional.

^{1}H NMR spectra were recorded on a Brucker Avance III 400 MHz (400 and 377 MHz for ^{1}H and ^{19}F NMR, respectively). Chemical shifts were reported as δ values (ppm) with tetramethylsilane (TMS) as the internal standard. The splitting patterns are designated as follows: singlet (s), doublet (d), triplet (t), and multiplet (m). MALDI TOF HRMS were recorded on a Bruker UltrafleXtreme TOF/TOF, trans-2-[3-(4-tert-butylphenyl)-2-methyl-propenylidene]malonitrile (DCTB) and 2,5-Dihydroxybenzoic acid (DHB) were used as matrix. UV-vis absorption spectra and PL were performed by TECAN with a Microplate Reader infinite 200 Pro. Conductivity measurements were performed by measuring I-V characteristics between two ITO electrodes using a Keithley 2401 source meter. For the conductivity measurement, solutions of materials concentrations of 5 mg/ml in chlorobenzene were prepared. The solutions were spin-coated onto patterned ITO substrates by robot at 500 rpm for 3 min. The conductivity (σ) was calculated by the following equation $\sigma = W/(RLD)$, where L was the channel length with 5 mm, W was the channel width 233 µm, D was the thickness of film with around 50 nm measured by Stylus Profilers, and R was the film resistance derived from the slope of the I-V curves.

HT Electrochemical characterization. A scanning flow cell (SFC) is used to investigate the electrochemical behavior of the large number of molecules present on the material library. Each sample on the library serves as working electrode, of which the geometric surface area is defined by the circular port of the SFC (about 0.01 cm^2). Mounted upstream in the inlet duct of the SFC, a graphite rod is used as counter electrode. A conventional Ag/AgCl/3M KCl reference electrode (Metrohm, Germany) is connected via Luggin capillary to the outlet duct downstream the SFC. All SFC components, gas flow and the potentiostat (Gamry Reference 600, USA) are controlled by a homemade LabVIEW software (National Instruments, USA). An Ar saturated phosphate buffer solution (5.2 mM KH_2PO_4 and 8.2 mM Na_2HPO_4 solution), prepared each day before the measurements by dissolution of KH_2PO_4 and Na_2HPO_4 salts in ultrapure water (18 mΩ cm, TOC <3 ppb), is used as electrolyte for all electrochemical measurements. The protocol for electrochemical testing comprises an initial sweep of potential from 0-1.8 $V_{Ag/AgCl}$ and subsequently 3 potential cycles from -0.5-1.8 $V_{Ag/AgCl}$ at a scan rate of 100 mV s^{-1}. Normal cyclic voltammetry (CV) test was obtained in a tetrabutylammonium hexafluorophosphate (TBAPF6, 0.1 mol L^{-1}) supported CH_2Cl_2 solution at room temperature using a Zahner Zennium Pro electrochemical workstation operated at a scanning

rate of 100 mV s^{-1}. A Pt wire (φ = 1.0 mm) embedded in Teflon column was used as the working electrode, and a Pt sheet and Ag/AgCl electrodes were served as the counter and reference electrodes, respectively.

Device fabrication. Perovskite solar cell devices having the structure of ITO/HTM/perovskite/PCBM/BCP/Ag were fabricated, where the Indium tin oxide (ITO) was the bottom layer. The process for fabricating the optoelectronic devices is summarized as follows. ITO glasses were cleaned with detergent, deionized water, and acetone and sonicated with isopropanol in an ultrasonic bath for 30 minutes. After that, ITO glasses were treated in a UV cleaner for 20 minutes. HTM (2 mg/ml in chlorobenzene) was spin-coated on the ITO substrate at 5000 rpm for 30s and then annealed at 100 °C for 10 min.

For perovskite solution, $PbBr_2$ (1.5M) and PbI_2 (1.5M) are dissolved in a mixture of anhydrous Dimethylformamide (DMF): Dimethyl sulfoxide (DMSO) (4:1 volume ratio) and added to formamidinium iodide (1.09:1 molar ratio) and methylammonium bromide (1.09:1 molar ratio) powders respectively, to obtain $MAPbBr_3$ and $FAPbI_3$ solutions with a final concentration of 1.24 M. These two solutions are then mixed in a 17:83 volume ratio. Finally, the cesium cation is added from a 1.5 M CsI solution in DMSO in a 5:95 volume ratio. The perovskite solution is spin-coated on top of the HTM layer using the following program: 1000rpm for 10s, 5000 rpm for 30 s. After 25 s, 250 µl of Chlorobenzene is dropped on the spinning substrate. Then the perovskite film is annealed at 100°C for 10 min, and 120°C for 10 min. PCBM solution (20 mg/ml in chlorobenzene) is spin-coated on top of perovskite at 2000rpm for 30s, then annealed at 80°C for 10 min. BCP solution (0.5 mg/ml in isopropanol) is spin-coated on top of PCBM at 5000rpm for 30s, annealed 80°C for 5 min. 100 nm thick Ag layer was thermally evaporated under a vacuum of 8×10^{-6} mbar at a rate of ~0.1 nm/s to finish the device fabrication. The device active area was 0.08 cm^{-2}.

2. Reaction optimization based on reference materials Re1 and Re2.

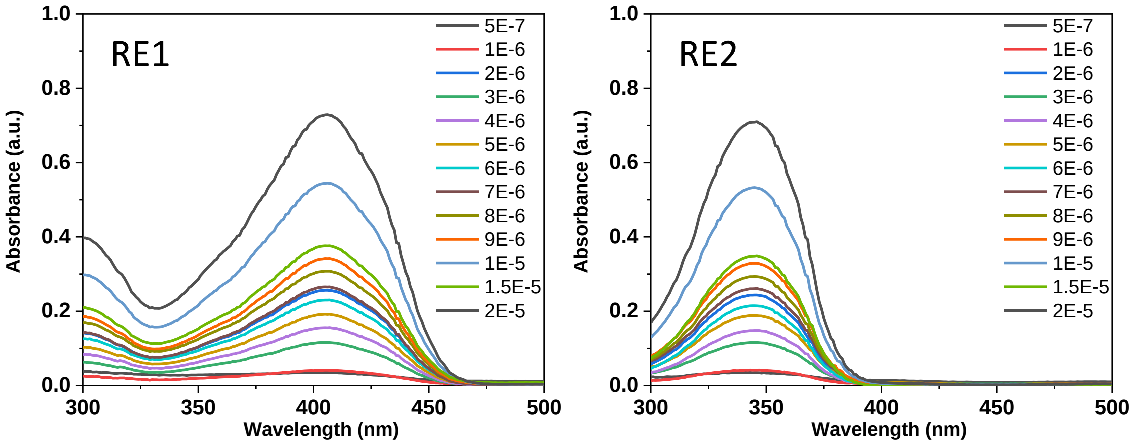

Supplementary Figure 1. UV-vis absorption spectra of RE1 and RE2 in DMF with concentration around 10^{-7}-10^{-5} mol·L^{-1}.

Synthesis of RE1 based on (4-(bis(4-methoxyphenyl)amino)phenyl)boronic acid

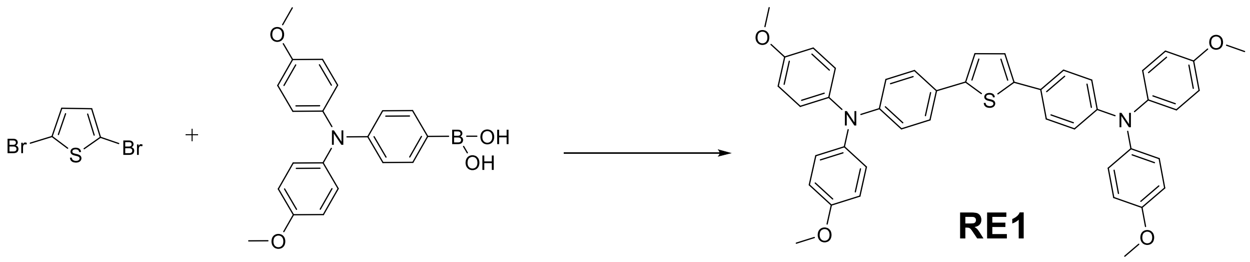

Supplementary Table 1. Optimization microwave-assisted experiments conditions for reference 1.

Reaction	Boric acid compound equiv.[a]	Solvent	Temp[b]/°C	Time/min	Base[c]	Catalyst[d]	Yield
1	1.2	THF	80	30	K_2CO_3	$Pd(PPh_3)_4$	65%
2	1.5	THF	80	30	K_2CO_3	$Pd(PPh_3)_4$	70%
3	2	THF	80	30	K_2CO_3	$Pd(PPh_3)_4$	80%
4	3	THF	80	30	K_2CO_3	$Pd(PPh_3)_4$	78%
5	4	THF	80	30	K_2CO_3	$Pd(PPh_3)_4$	79%
6	2	THF	60	30	K_2CO_3	$Pd(PPh_3)_4$	50%
7	2	THF	70	30	K_2CO_3	$Pd(PPh_3)_4$	65%
8	2	THF	80	30	K_2CO_3	$Pd(PPh_3)_4$	80%
9	2	THF	90	30	K_2CO_3	$Pd(PPh_3)_4$	95%
10	2	THF	100	30	K_2CO_3	$Pd(PPh_3)_4$	95%
11	2	THF	110	30	K_2CO_3	$Pd(PPh_3)_4$	90%
12	2	Toluene[e]	80	30	K_2CO_3	$Pd(PPh_3)_4$	70%
13	2	Toluene[e]	90	30	K_2CO_3	$Pd(PPh_3)_4$	69%
14	2	Toluene[e]	100	30	K_2CO_3	$Pd(PPh_3)_4$	75%
15	2	Toluene[e]	110	30	K_2CO_3	$Pd(PPh_3)_4$	72%
16	2	Toluene[e]	120	30	K_2CO_3	$Pd(PPh_3)_4$	75%
17	2	Toluene[e]	130	30	K_2CO_3	$Pd(PPh_3)_4$	70%

[a] the equivalent of MIDA to dibromo compounds; [b] temperature ramp: 30°C/min, the power of the microwave was limited not to exceed 100W; [c] K_3PO_4 3M in H_2O, K_2CO_3 2M in H_2O; [d] $Pd(OAC)_2$ was used with SPhos as ligand; [e] contained 1.5 vol.% Starks' catalyst.

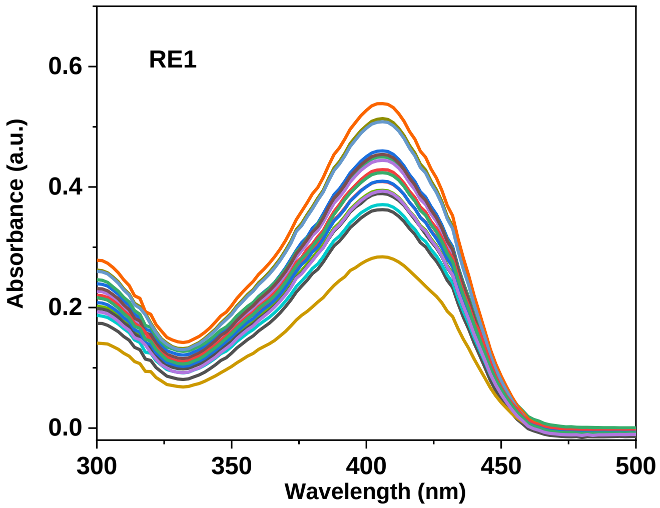

Supplementary Figure 2. UV-vis absorption of diluted reaction solution at different conditions in Supplementary Table 1. The reaction solution was diluted 3000 times, where the concentration of product (100% yield) was $1.5x10^{-5}$ M.

Solvents screening of recrystallization for RE1 and RE2.

First, mother liquors with a concentration of 50 mg/ml in THF were prepared. Then, 0.2ml mother liquors were added into vials. After the solvents were removed by heating, the good solvent and anti-solvent were added into the vials to dissolve the materials. In the initial solvent screening, we used 1ml mixed solvent (good solvent: anti-solvent= 5:1, in volume) to dissolve the RE1 and RE2, respectively. The mixed solvent that could not dissolve the materials would be excluded. After preliminary screening, several solvents, THF and Chloroform were chosen for good solvent, while methanol (MeOH) and hexane as anti-solvent. Meanwhile, in this progress we found when the ratio was 1:3 (good solvent: anti-solvent), most of mixed solvent could not dissolve the RE1 and RE2. Therefore, in the next part, we set the ration of good solvent to anti-solvent from 3:1 to 1:3.

Supplementary Table 2. Recrystallization yields of RE1 with different mixed solvents

Yield(%)	3:1	2:1	1:1	1:2	1:3
THF:MeOH	0	22	52	60	x
THF:Hexane	10	50	80	81	x
CH_2Cl_2:MeOH	0	0	31	55	60
CH_2Cl_2:Hexane	0	0	0	10	25
Toluene:MeOH	0	0	30	31	x
Toluene:Hexane	0	10	20	41	x

x: not completely dissolved

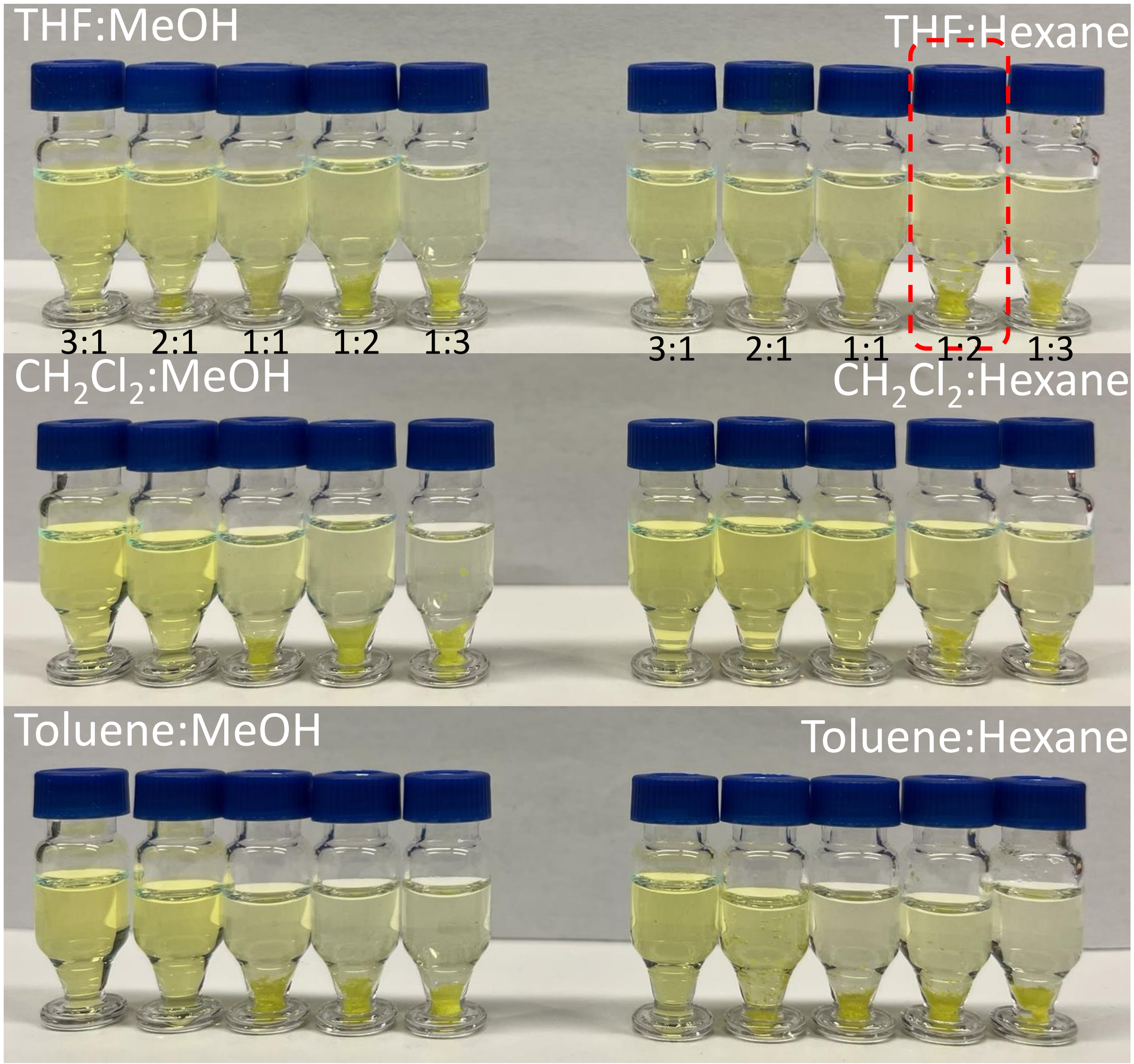

Supplementary Figure 3. Recrystallization of RE1 with different mixed solvents.

Supplementary Table 3. Recrystallization yields of RE2 recrystallization with different mixed solvents.

Yield(%)	3:1	2:1	1:1	1:2	1:3
THF:MeOH	0	0	25	92	x
THF:Hexane	0	0	0	62	x
$CHCl_3$:MeOH	0	0	60	85	x
$CHCl_3$:Hexane	0	0	0	0	80

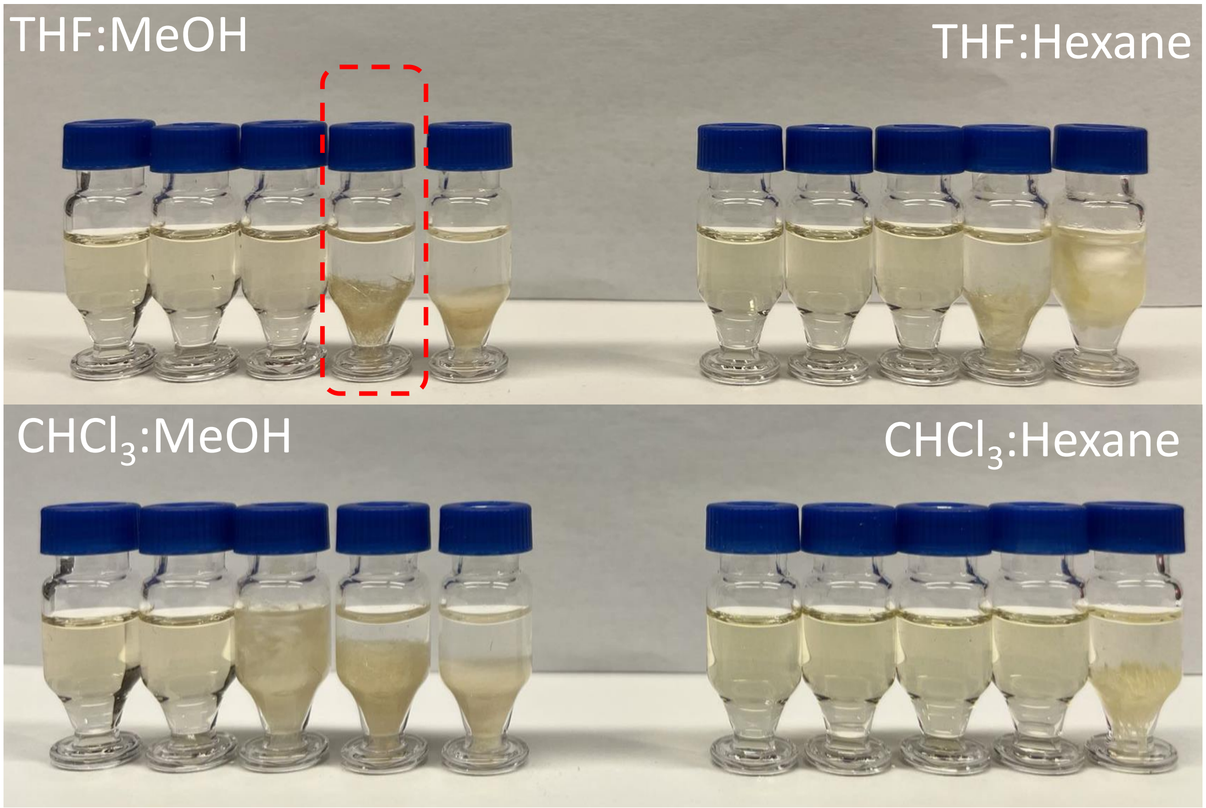

Supplementary Figure 4. Recrystallization of RE2 with different mixed solvents.

3. High-throughput syntheses and purification.

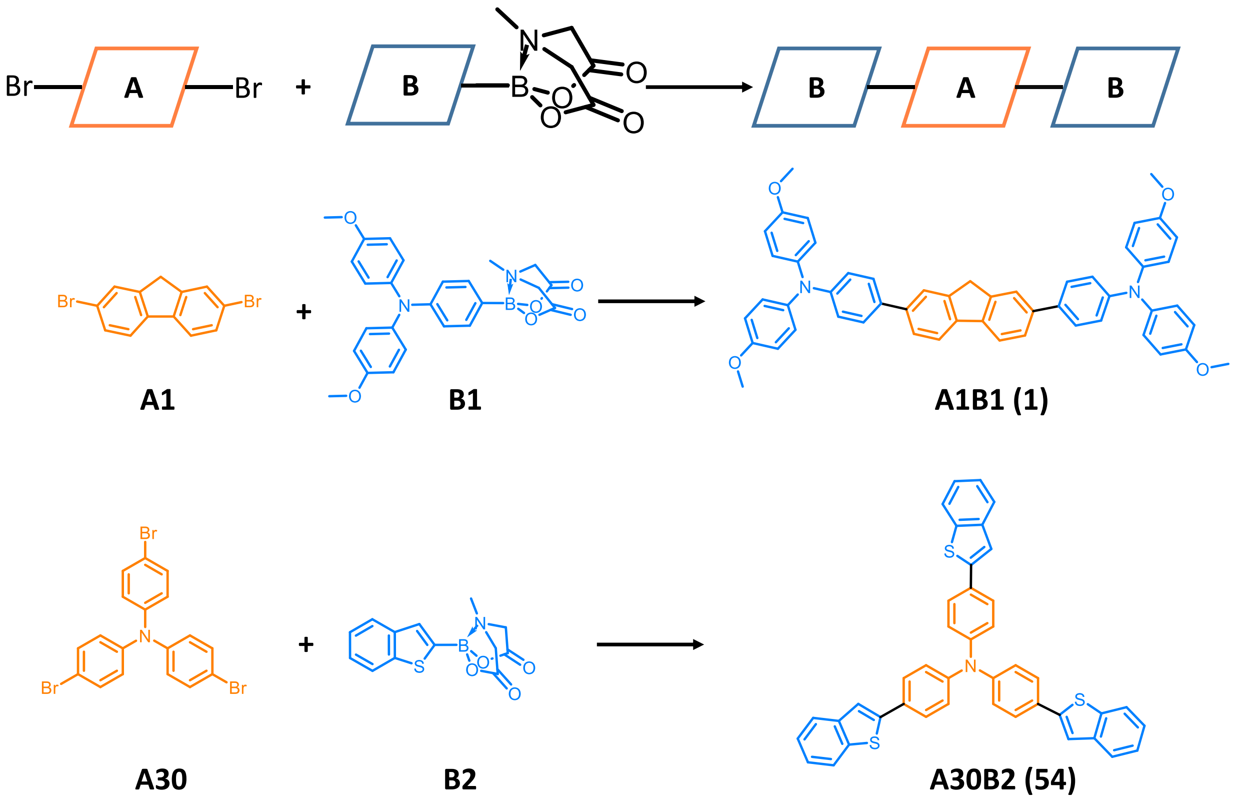

Supplementary Figure 5. Schematic diagram of synthesizing all molecules and the naming rules.

We name the molecules by combing the serial number of two reactants. In addition, to we also describe the molecules with Arabic numbers to simplify the experimental recording. For example, the sample 54 is A30B2.

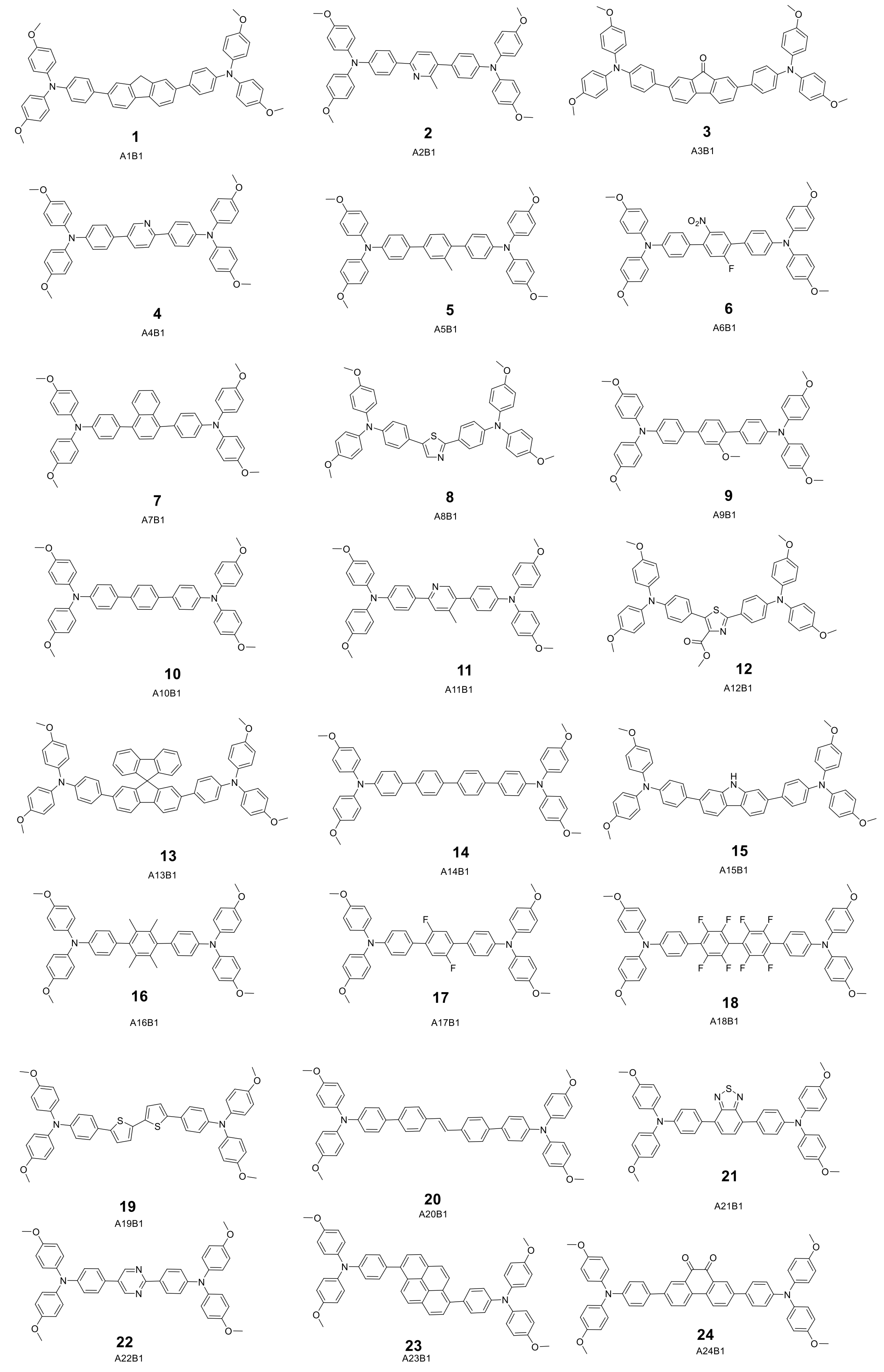

Supplementary Figure 6. Chemical structures of synthesized small molecules.

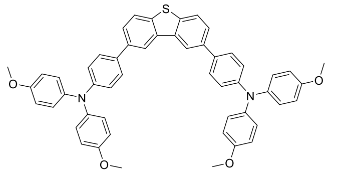

25

A25B1

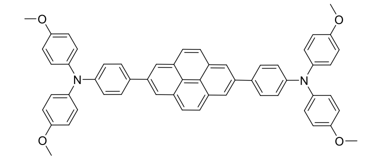

26

A26B1

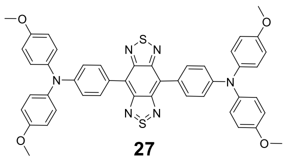

27

A27B1

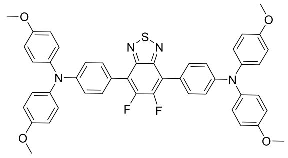

28

A28B1

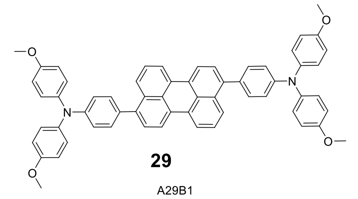

29

A29B1

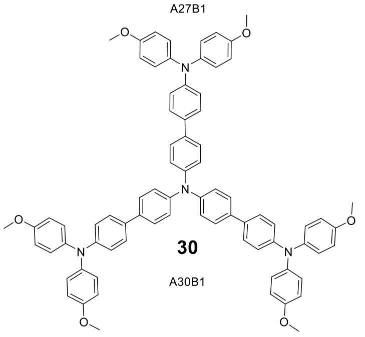

30

A30B1

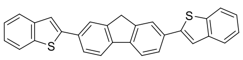

31

A1B2

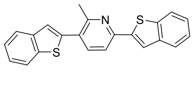

32

A2B2

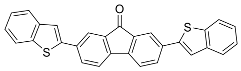

33

A3B2

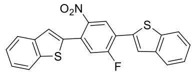

34

A6B2

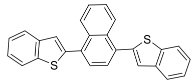

35

A7B2

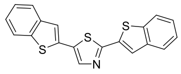

36

A8B2

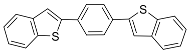

37

A10B2

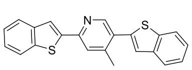

38

A11B2

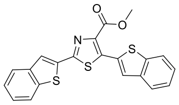

39

A12B2

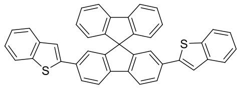

40

A13B2

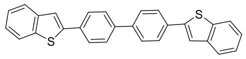

41

A14B2

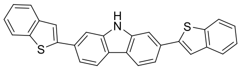

42

A15B2

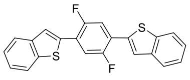

43

A17B2

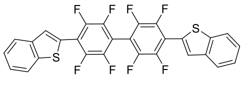

44

A18B2

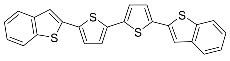

45

A19B2

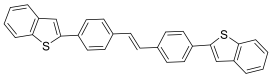

46

A20B2

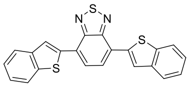

47

A21B2

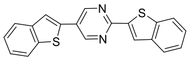

48

A22B2

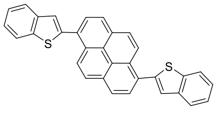

49

A23B2

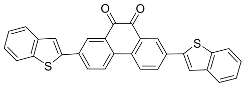

50

A24B2

Supplementary Figure 6. Chemical structures of synthesized small molecules.

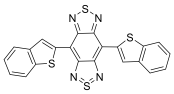

51
A27B2

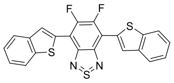

52
A28B2

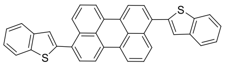

53
A29B2

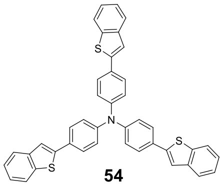

54
A30B2

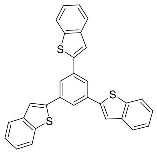

55
A31B2

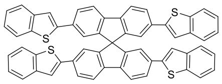

56
A32B2

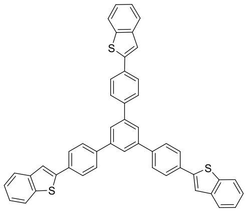

57
A33B2

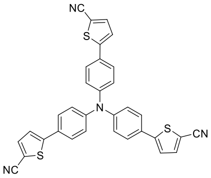

58
A30B3

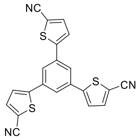

59
A31B3

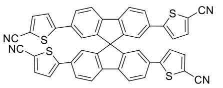

60
A32B3

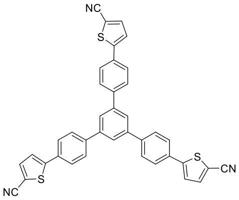

61
A33B3

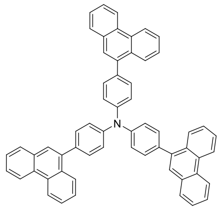

62
A30B4

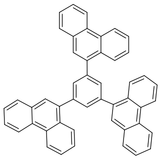

63
A31B4

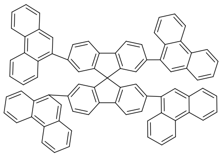

64
A32B4

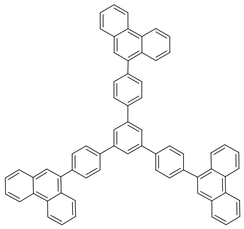

65
A33B4

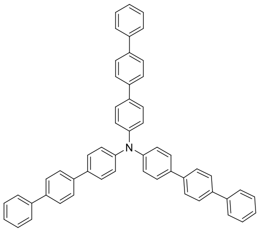

66
A30B5

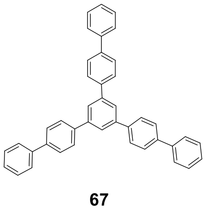

67
A31B5

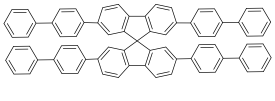

68
A32B5

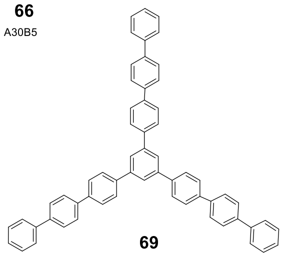

69
A33B5

Supplementary Figure 6. Chemical structures of synthesized small molecules.

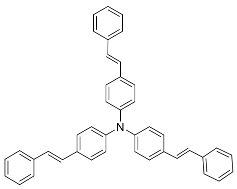

70
A30B6

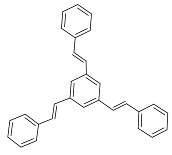

71
A31B6

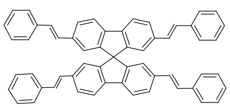

72
A32B6

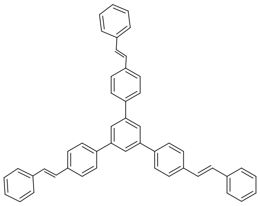

73
A33B6

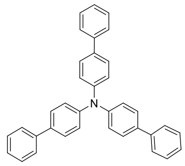

74
A30B7

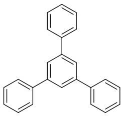

75
A31B7

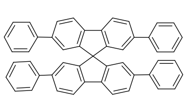

76
A32B7

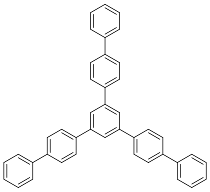

77
A33B7

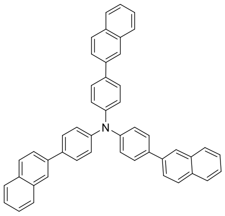

78
A30B8

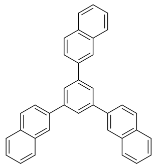

79
A31B8

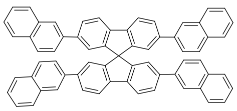

80
A32B8

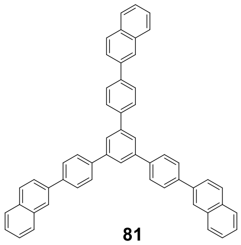

81
A33B8

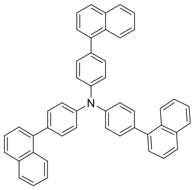

82
A30B8

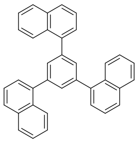

83
A31B9

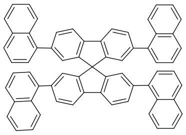

84
A32B9

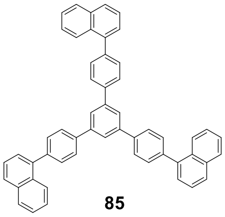

85
A33B9

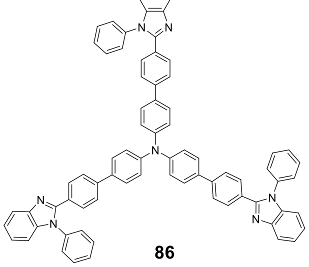

86
A30B10

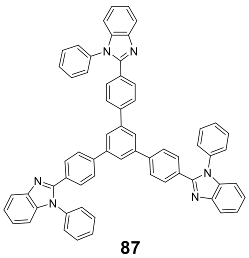

87
A31B10

Supplementary Figure 6. Chemical structures of synthesized small molecules.

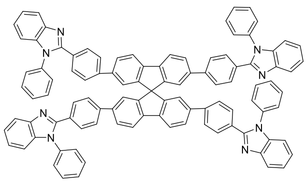

88
A32B10

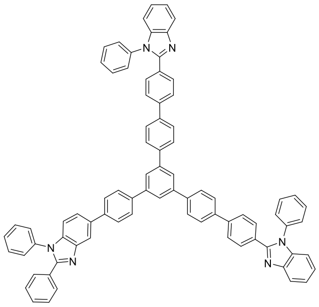

89
A33B10

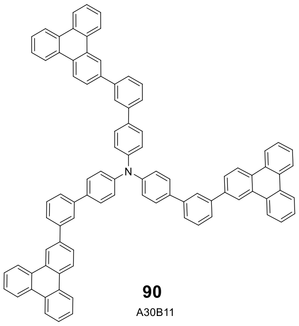

90
A30B11

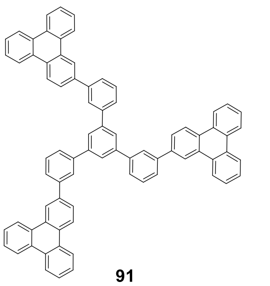

91
A31B11

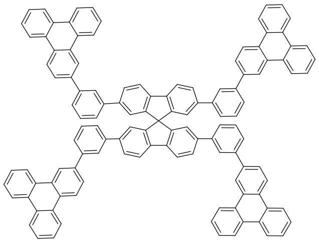

92
A32B11

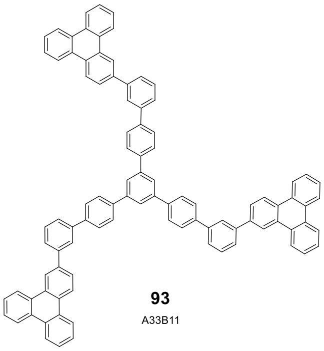

93
A33B11

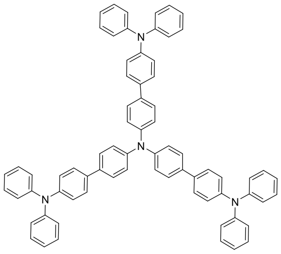

94
A30B12

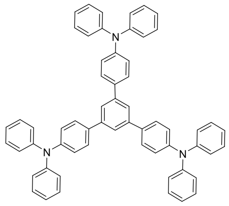

95
A31B12

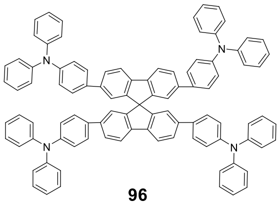

96
A32B12

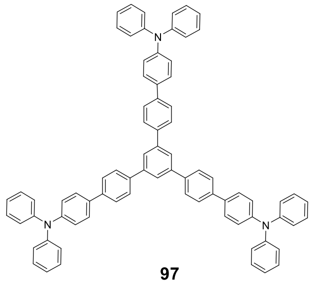

97
A33B12

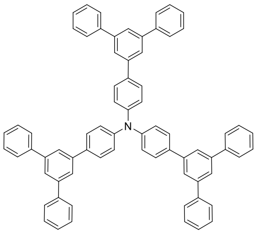

98
A30B13

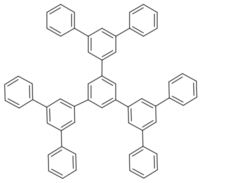

99
A31B13

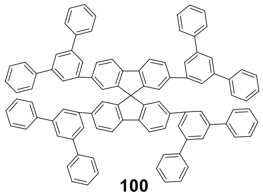

100
A32B13

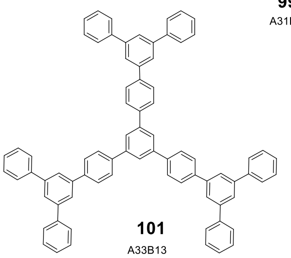

101
A33B13

Supplementary Figure 6. Chemical structures of synthesized small molecules.

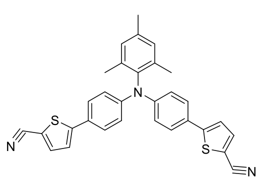

102
A1087B3

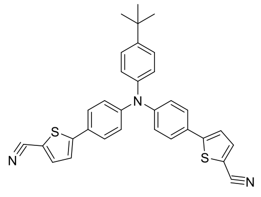

103
A1090B3

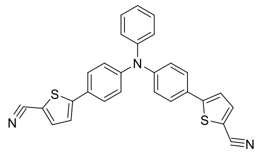

104
A1106B3

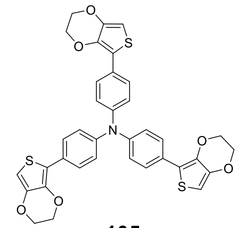

105
A30B770

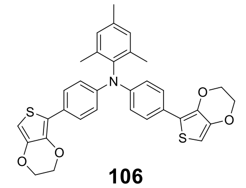

106
A1087B770

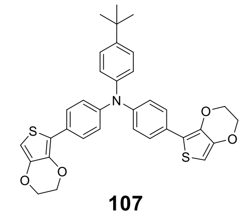

107
A1090B770

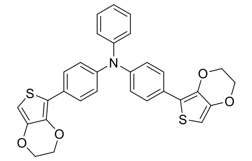

108
A1106B770

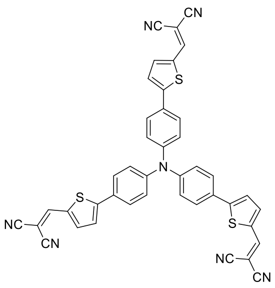

109
A30B772

Supplementary Figure 6. Chemical structures of synthesized small molecules.

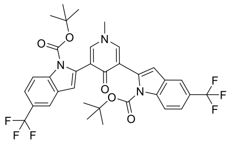

110
A525B386

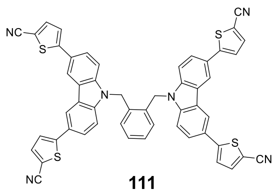

111
A1058B3

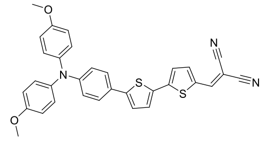

112
A1104B1

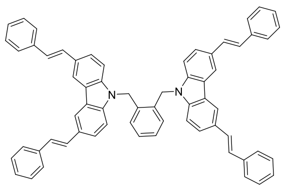

113
A1058B6

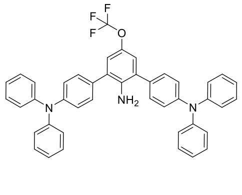

114
A51B12

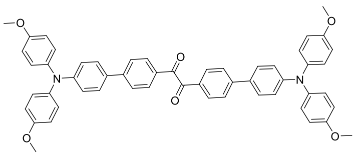

115
A1021B1

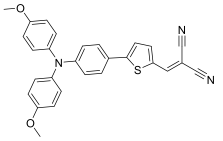

116
A1103B1

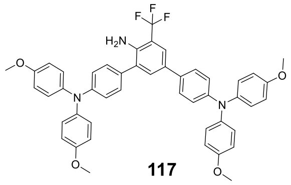

117
A52B1

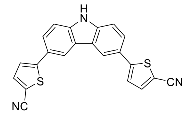

118
A359B3

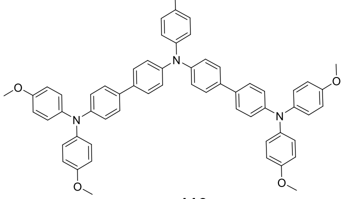

119
A1090B1

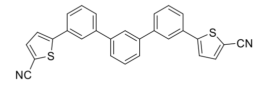

120
A1047B3

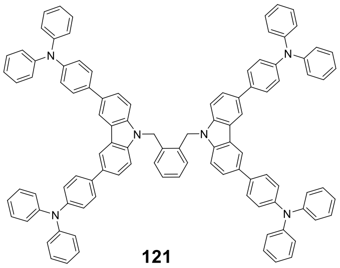

121
A1058B12

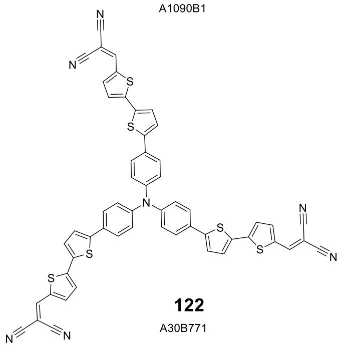

122
A30B771

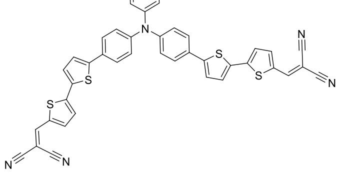

123
A1106B771

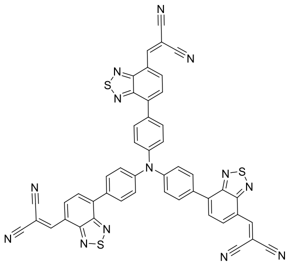

124
A30B773

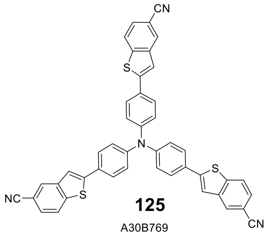

125
A30B769

Supplementary Figure 6. Chemical structures of synthesized small molecules.

Supplementary Table 4. Description of the further purification based on sample properties.

Sample properties					Possible reasons				Recrystallization again		
Entry	Encoding	Purity (80%)	Product-C (>10mg)	Products-F (>50mg)		Encoding	collect sample, then stop	stop	with more anti-solvent (1:5)	with more good solvent (5:1)	with pure THF
1	[1,0,0]	1	0	0	high solubility, low crystallinity	[0,0,1,0,0]	0	0	1	0	0
2	[1,0,1]	1	0	1	low solubility	[1,0,0,0,0]	1	0	0	0	0
3	[1,1,0]	1	1	0		[1,0,0,0,0]	1	0	0	0	0
4	[1,1,1]	1	1	1		[1,0,0,0,0]	1	0	0		
5	[0,0,0]	0	0	0	almost no reaction	[0,1,0,0,0]	0	1	0	0	0
6	[0,0,1]	0	0	1	low solubility	[0,0,0,1,0]	0	0	0	1	0
7	[0,1,0]	0	1	0	high solubility, low crystallinity	[0,0,1,0,0]		0	1	0	0
8	[0,1,1]	0	1	1	low solubility	[0,0,0,0,1]	0	0	0	0	1

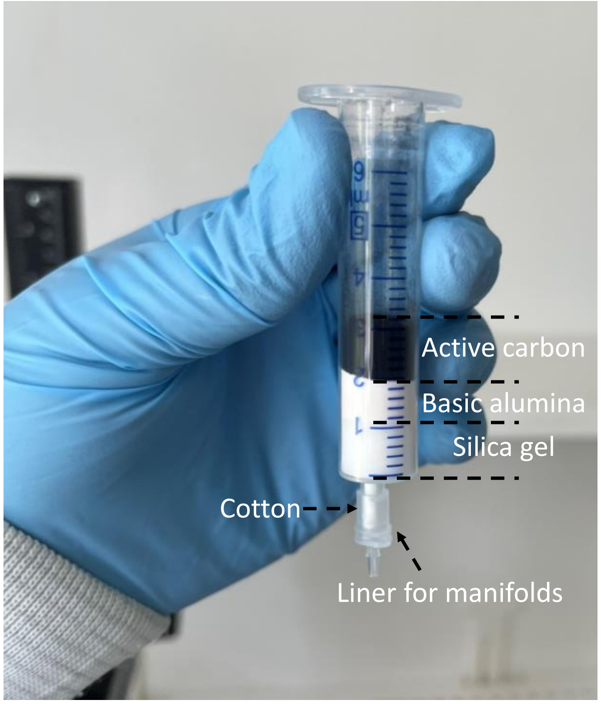

Supplementary Figure 7. Photograph of SEP for manifolds.

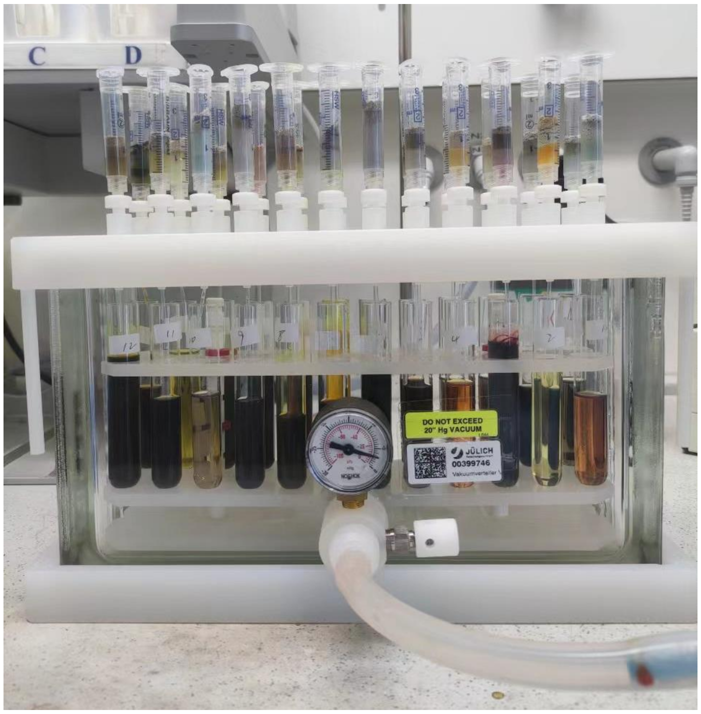

Supplementary Figure 8. Photograph of manifolds in operation (the corresponding is the step 2.1 filtration in Fig.1).

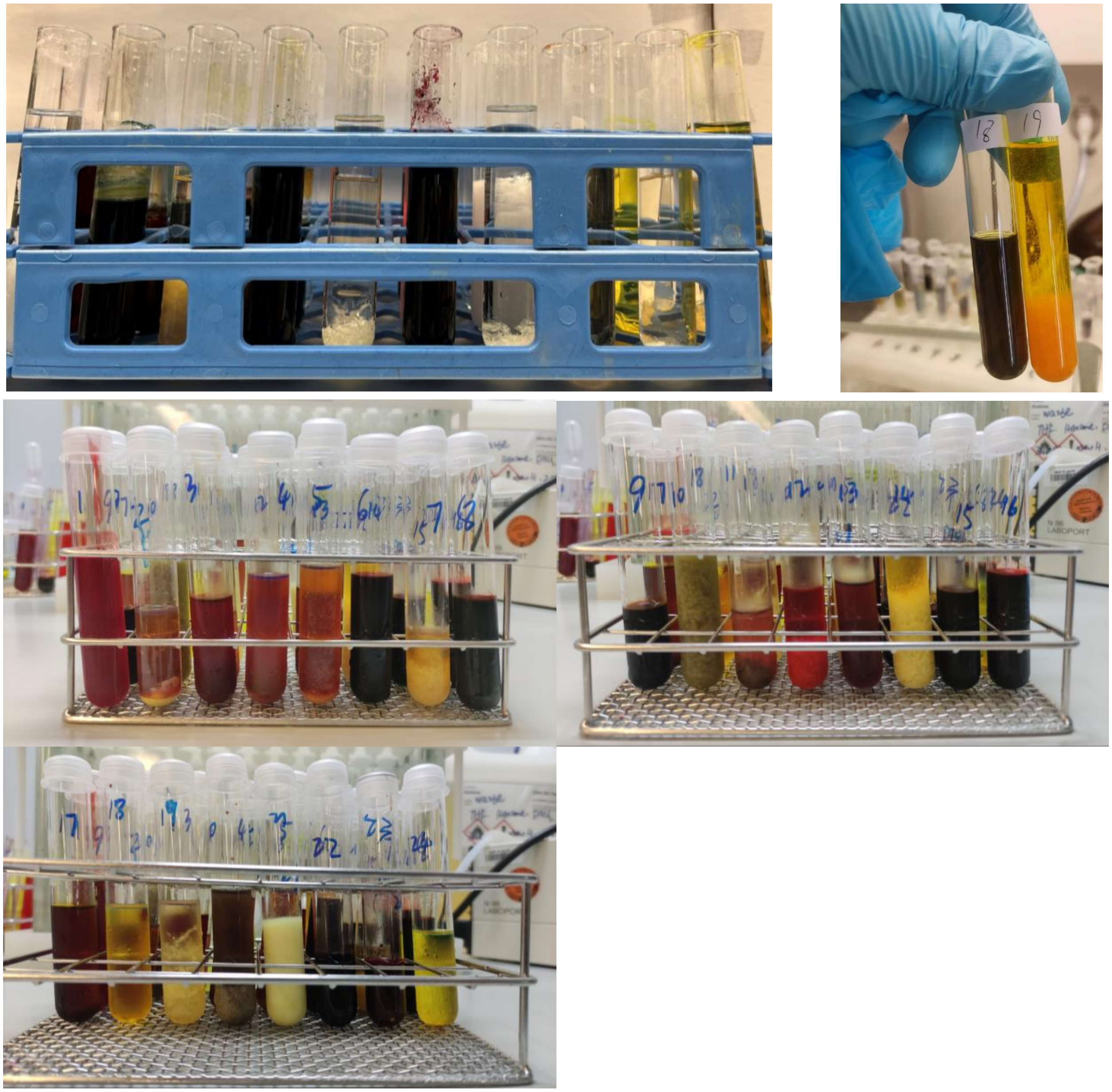

Supplementary Figure 9. Photograph of samples in recrystallization. Due to the dark color of some samples, it may be difficult to see the crystals in the solution. For example, there are 20 mg crystals in the solution for sample 18.

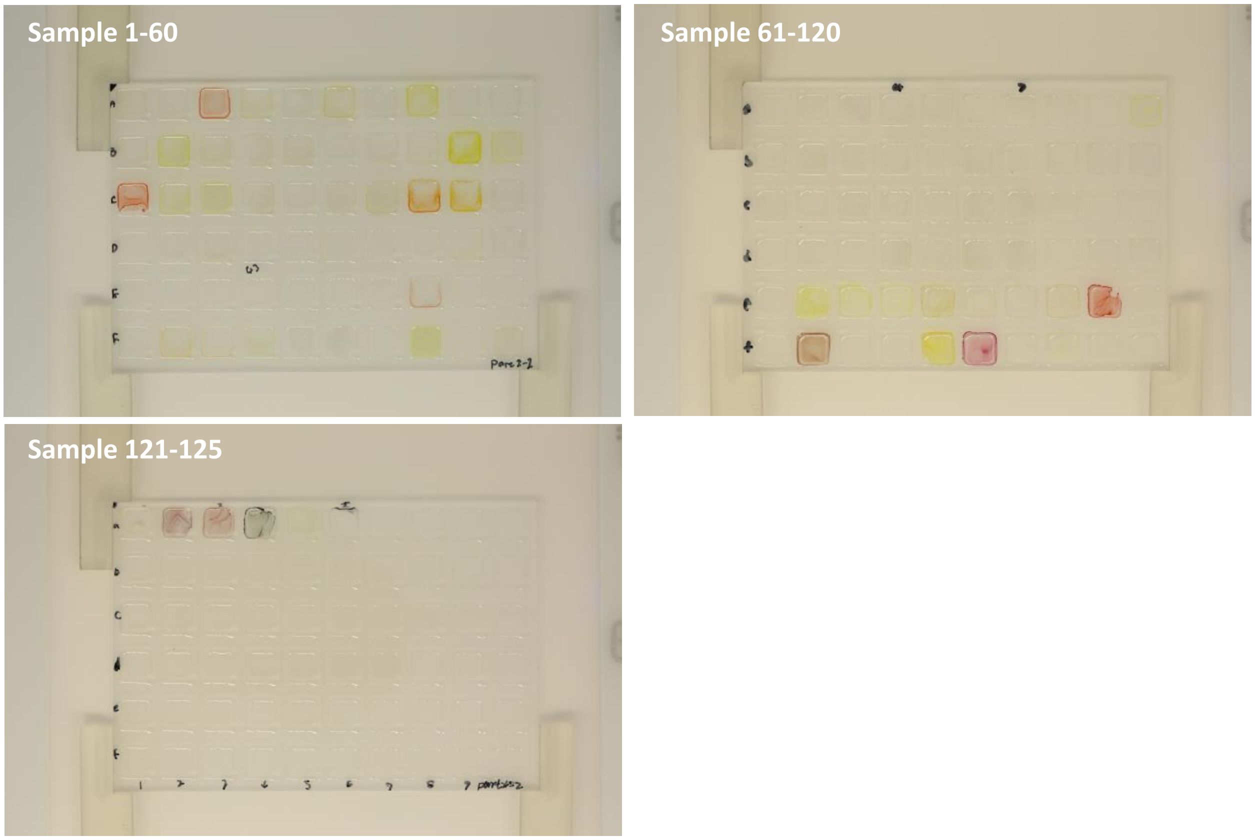

Supplementary Figure 10. Photograph of sample films via drop casting.

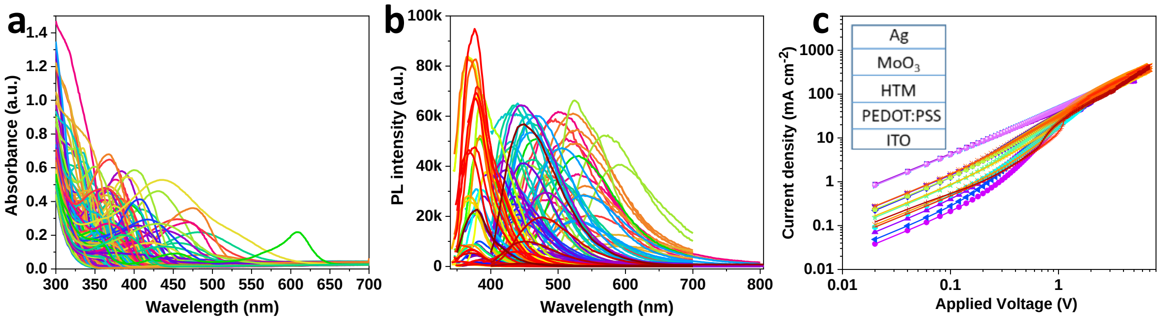

Supplementary Figure 11. UV-Vis absorption (a) and PL (b) of the synthesized materials in DMF. Concentrations are from 2.5 x 10^{-6} M to 1 x 10^{-5} M. c. Hole mobility of subsample (film).

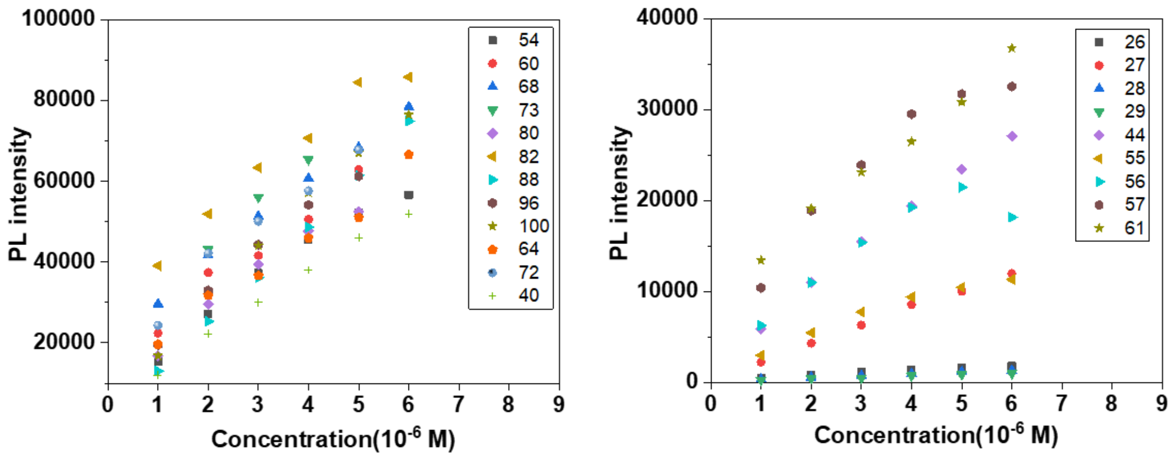

Supplementary Figure 12. PL of the materials in DMF with different concentrations.

Supplementary Table 5. Summary of sample properties, including purity weight, yield, and conductivity.

Supplementary Table 6. Summary of the data of UV-Vis absorption and PL.

Supplementary Table 7. Commercial Monomer A library.

Supplementary Table 8. Commercial Monomer B library.

Supplementary Table 9. Theoretical properties of computational pre-screened molecules.

Supplementary Table 10. Theoretical properties of synthesized molecules.

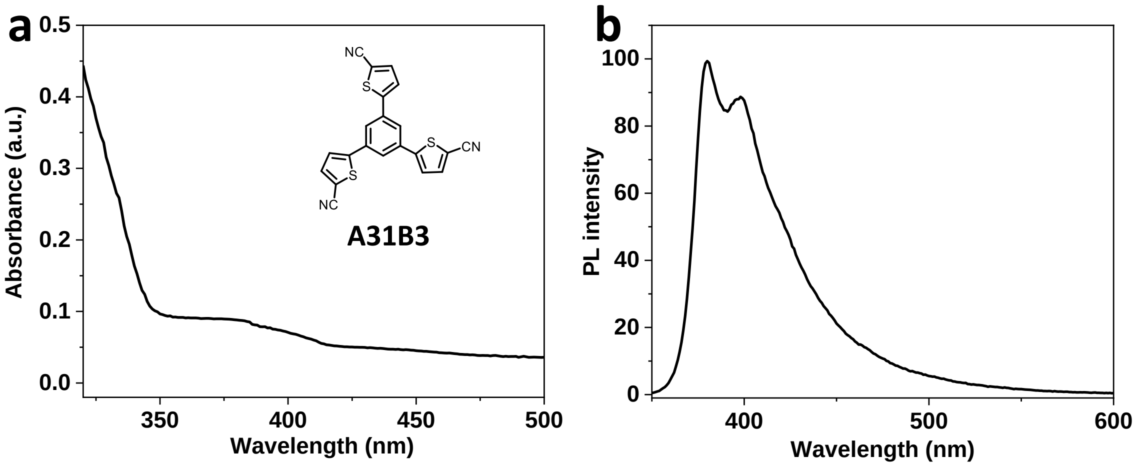

Supplementary Figure 13. UV-Vis absorption (a) and PL (b) of the A31B3 in DMF.

4. High throughput setups and corresponding data

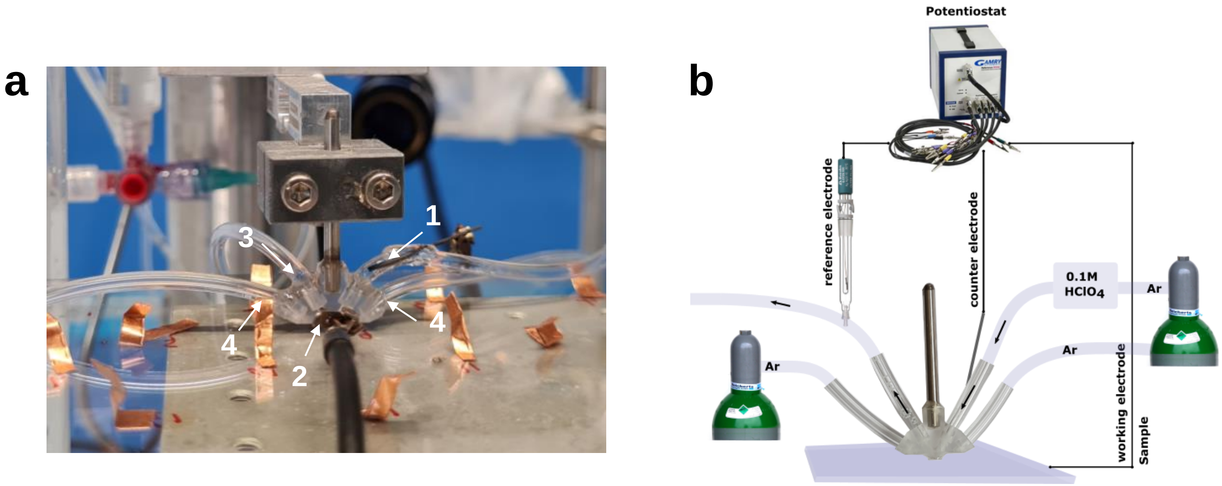

Supplementary Figure 14. Setup for high throughput CV test. Scanning flow cell (SFC) setup for electrochemical characterization. a): Picture of the SFC, pressed onto one of the samples of the material library. A copper tape was attached to each sample to ensure good electrical contact. Ar saturated electrolyte (5.2 mM KH_2PO_4 and 8.2 mM Na_2HPO_4 solution) is pumped through the inlet duct of the SFC (1), where a graphite rod is used as counter electrode. The electrolyte flows through the opening of the SFC (A=0.01 cm^2) via the sample surface (2) serving as working electrode, towards the outlet duct (3). The compartment with the reference electrode (Ag/AgCl/3M KCl) is connected to the outlet duct via Luggin capillary. During the whole measurement, Ar is streamed over the measured surface through gas channels at each side of the SFC (4). b): Schematic drawing of the SFC setup.

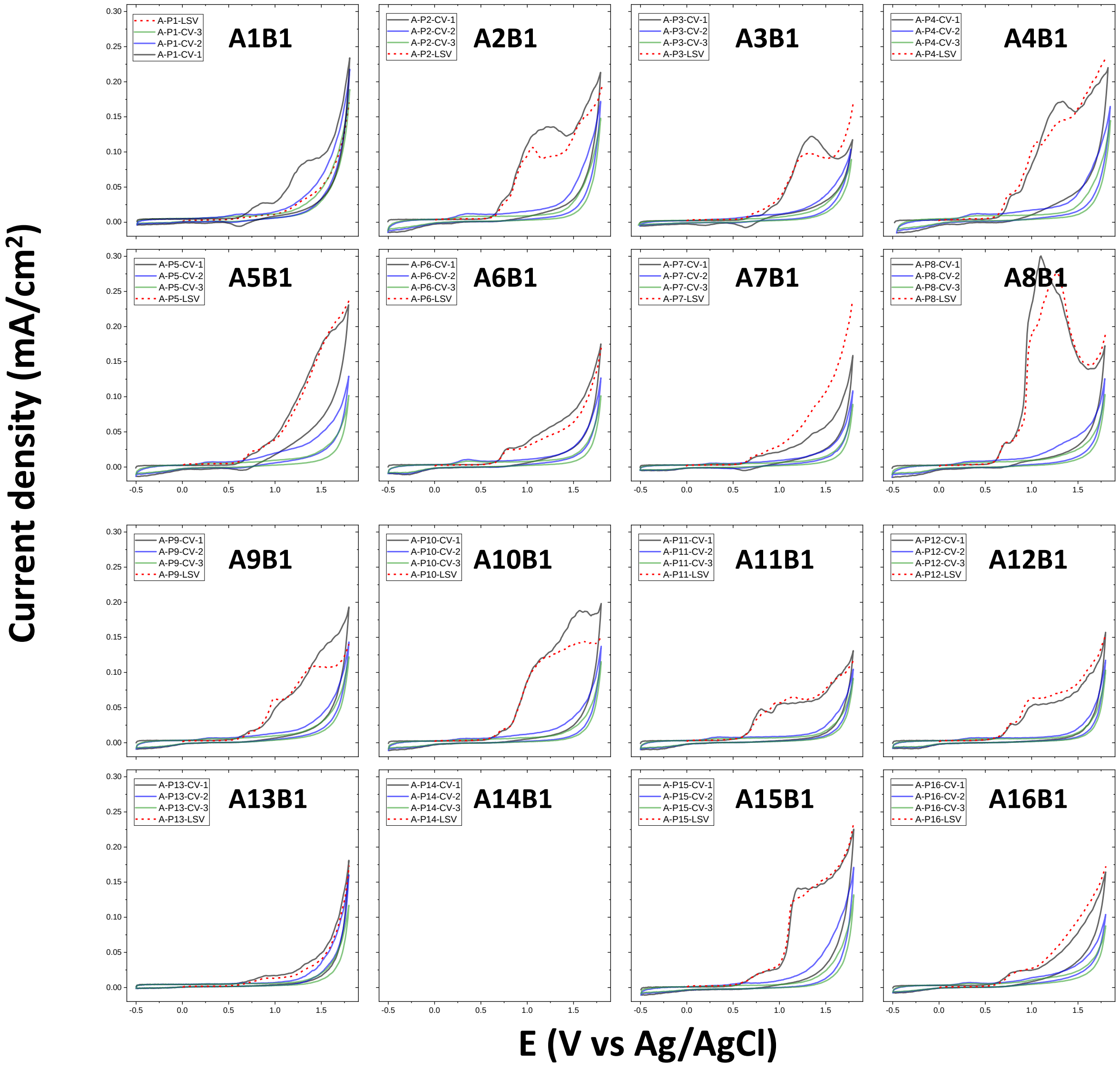

Supplementary Figure 15. Electrochemical data from Scanning flow cell (SFC) setup.

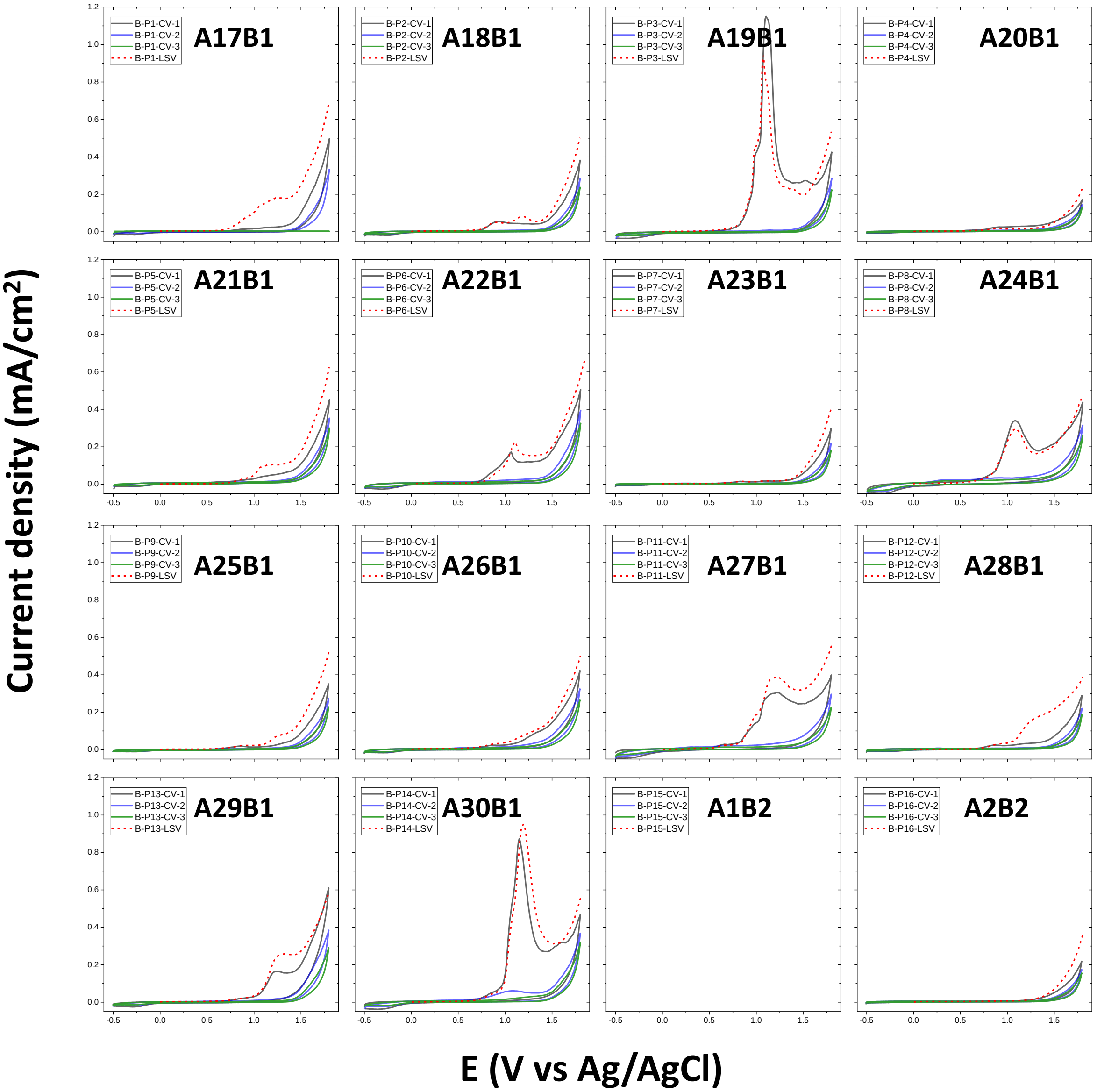

Supplementary Figure 15. Electrochemical data from Scanning flow cell (SFC) setup.

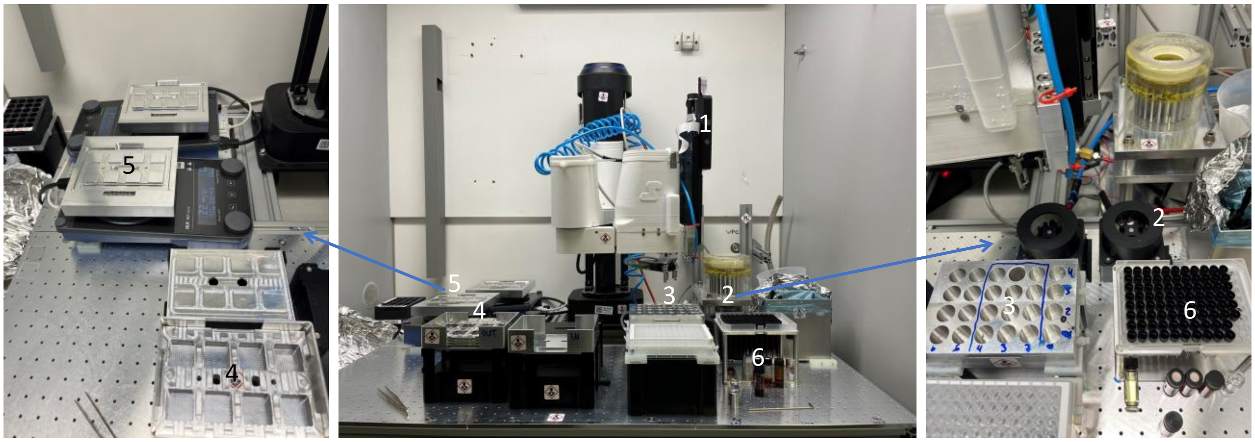

Supplementary Figure 16. Photograph of robotic spin-coater platform. 1. Robotic arm; 2. Spin-coater; 3. Stock solutions; 4. Stock substrates; 5. Hot plate; 6. Stock pipette tips.

5. Batch-to-batch reproducibility of the platform

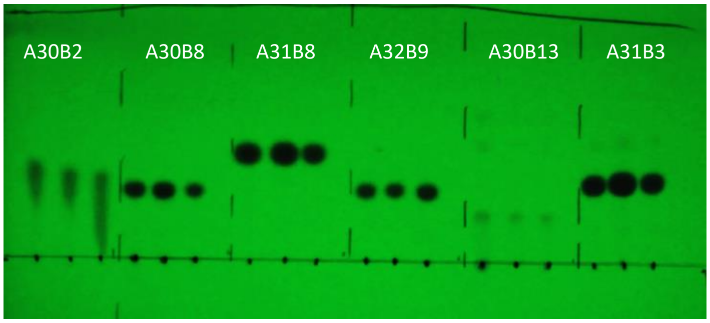

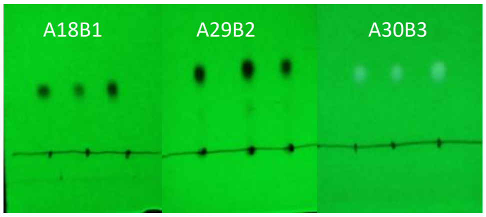

Supplementary Figure 17. Characterization of batch-to-batch repeatability. TLC of selected molecules in three batches.

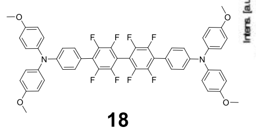

18
Batch 1

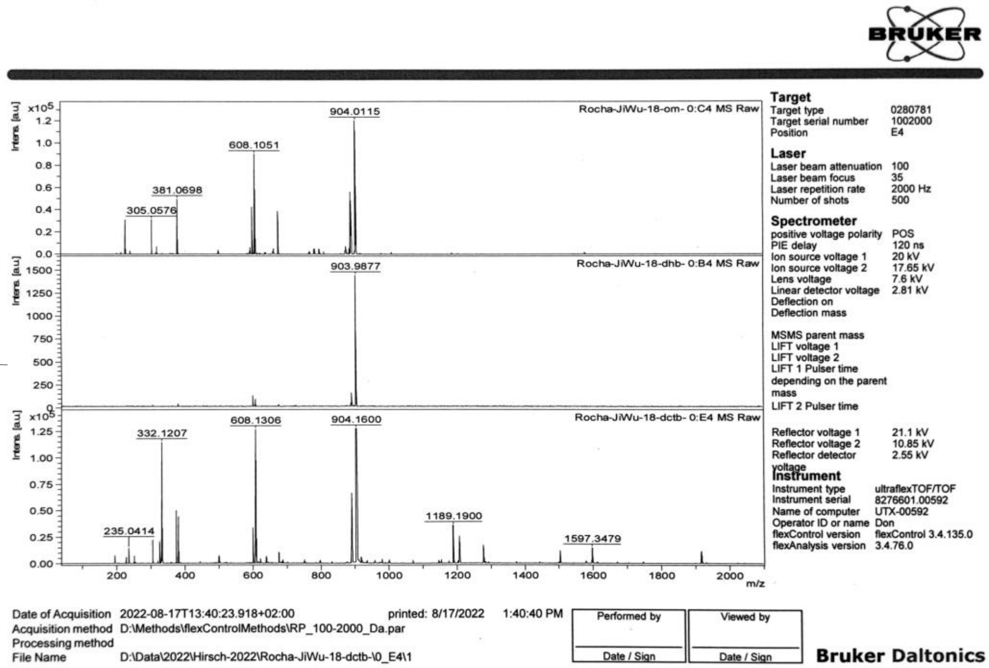

Supplementary Figure 18. HRMS of sample 18 (A18B1, Batch 1).

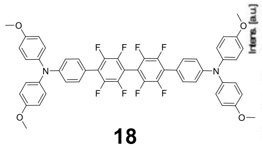

18
Batch 2

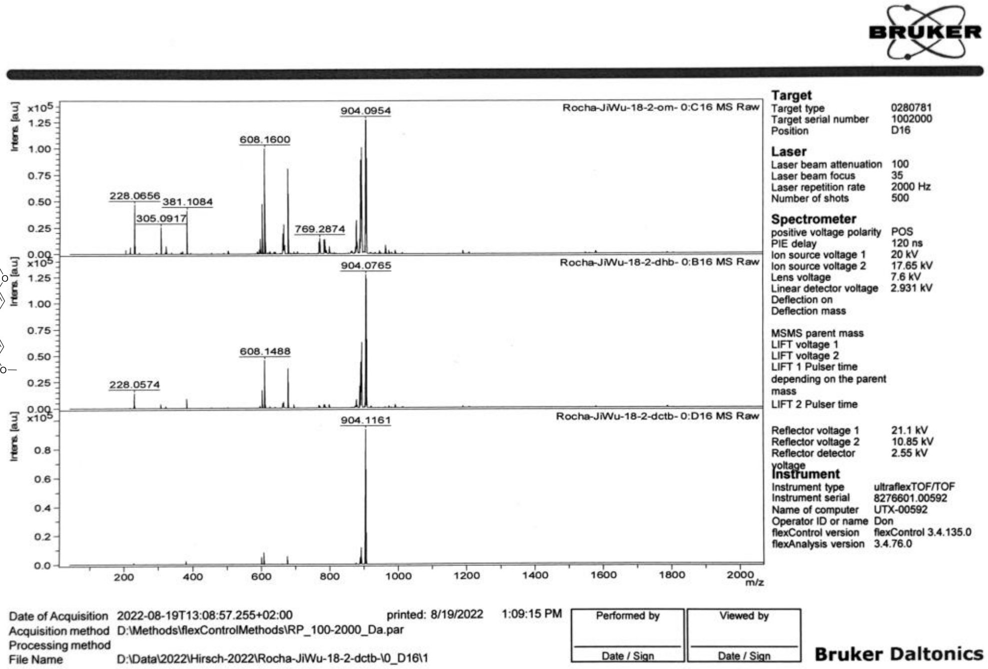

Supplementary Figure 19. HRMS of sample 18 (A18B1, Batch 2).

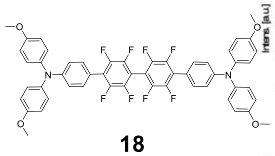

18
Batch 3

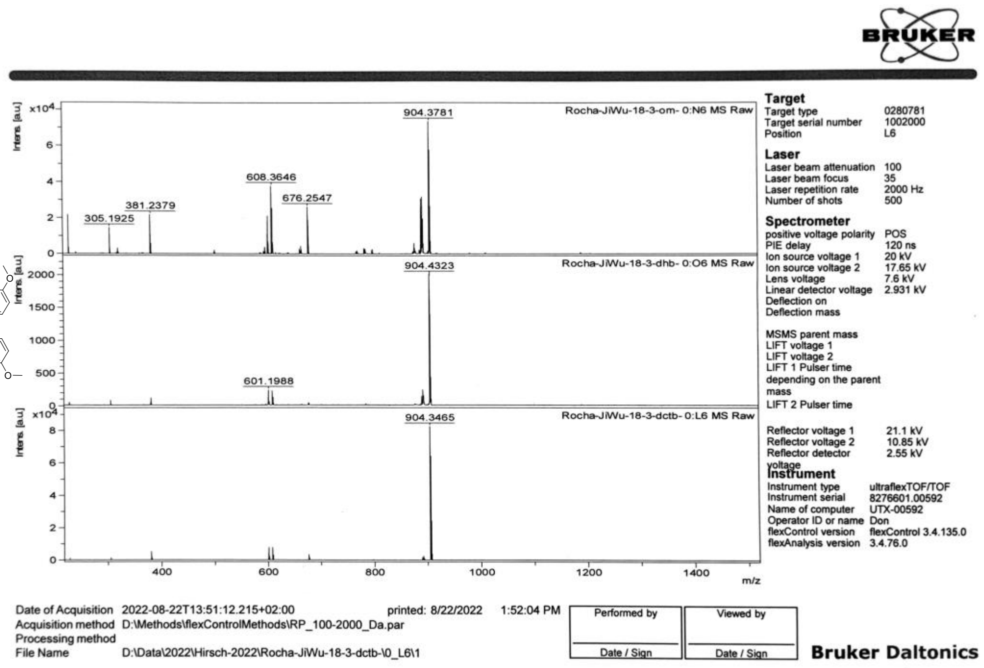

Supplementary Figure 20. HRMS of sample 18 (A18B1, Batch 3).

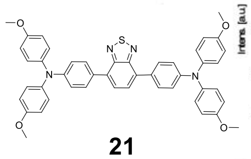

21
Batch 1

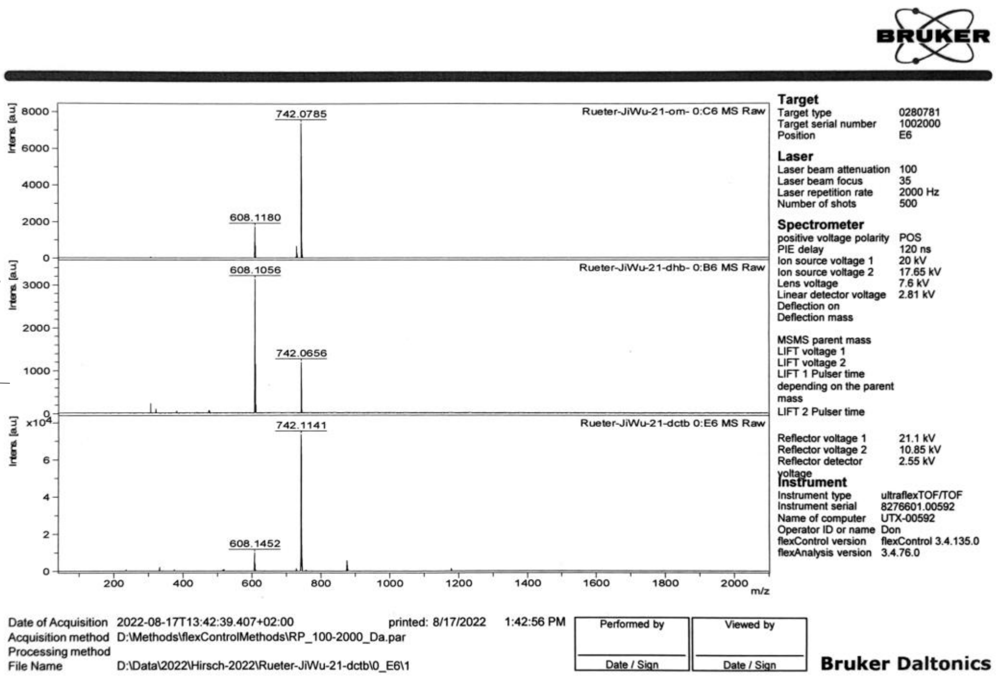

Supplementary Figure 21. HRMS of sample 21 (A21B1, Batch 1).

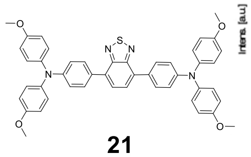

21
Batch 2

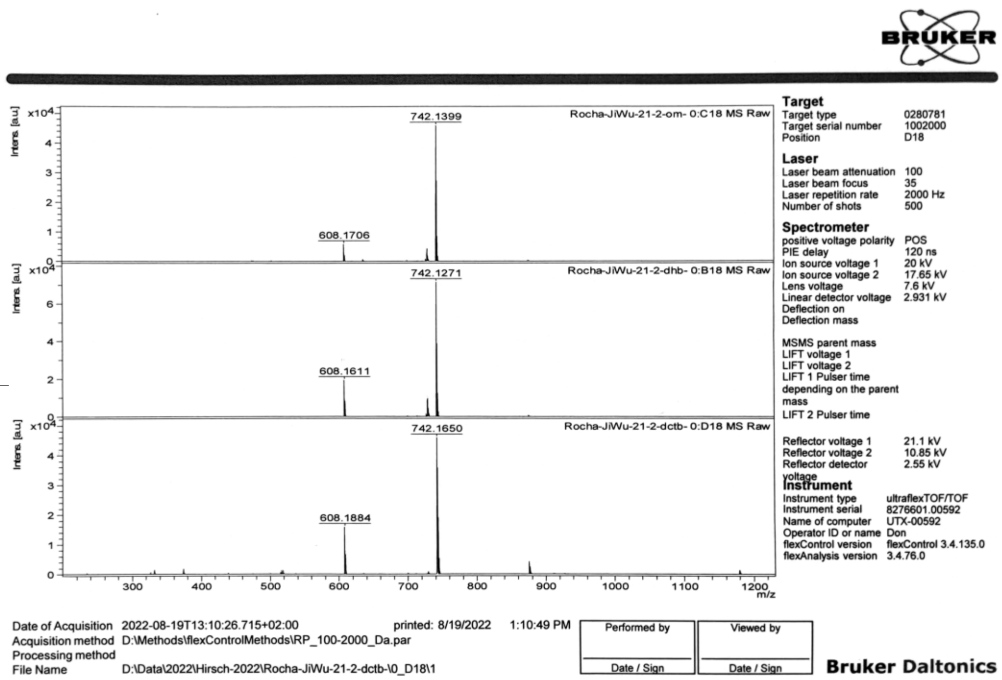

Supplementary Figure 22. HRMS of sample 21 (A21B1, Batch 2).

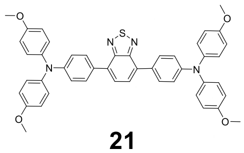

21
Batch 3

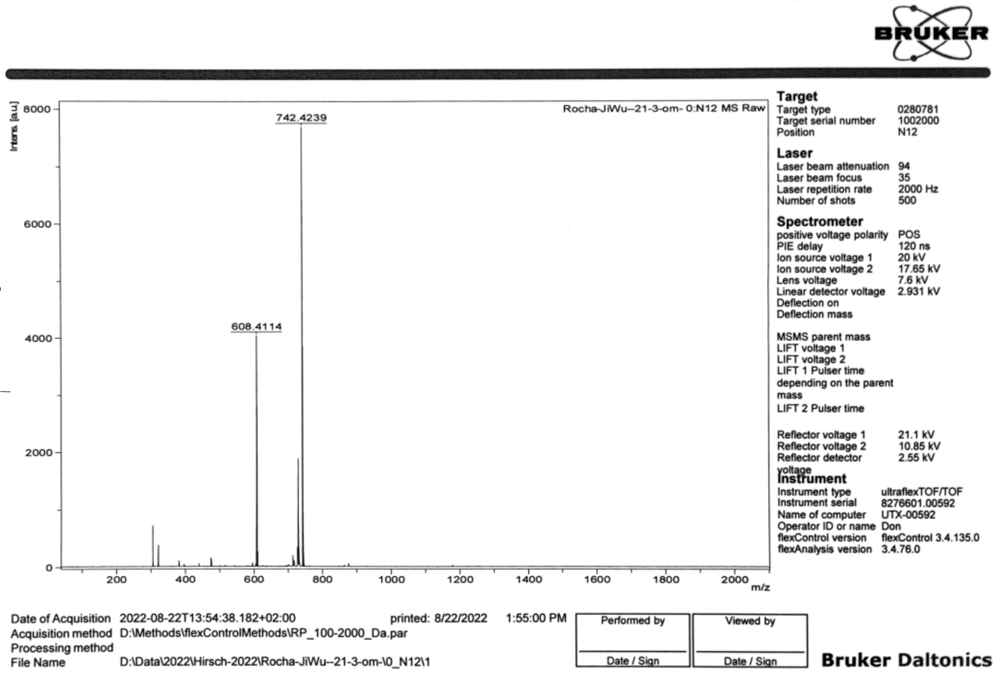

Supplementary Figure 23. HRMS of sample 21 (A21B1, Batch 3).

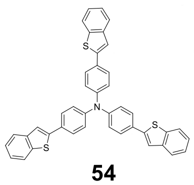

54
Batch 1

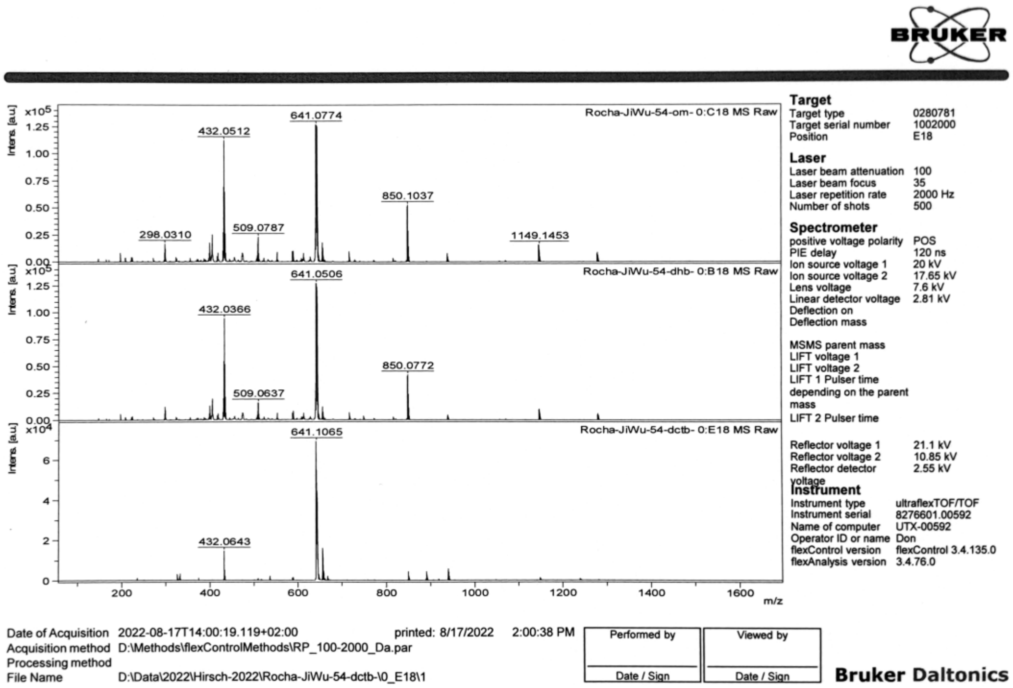

Supplementary Figure 24. HRMS of sample 54 (A30B2, Batch 1).

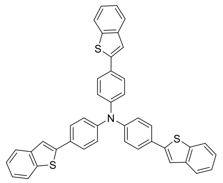

54
Batch 2

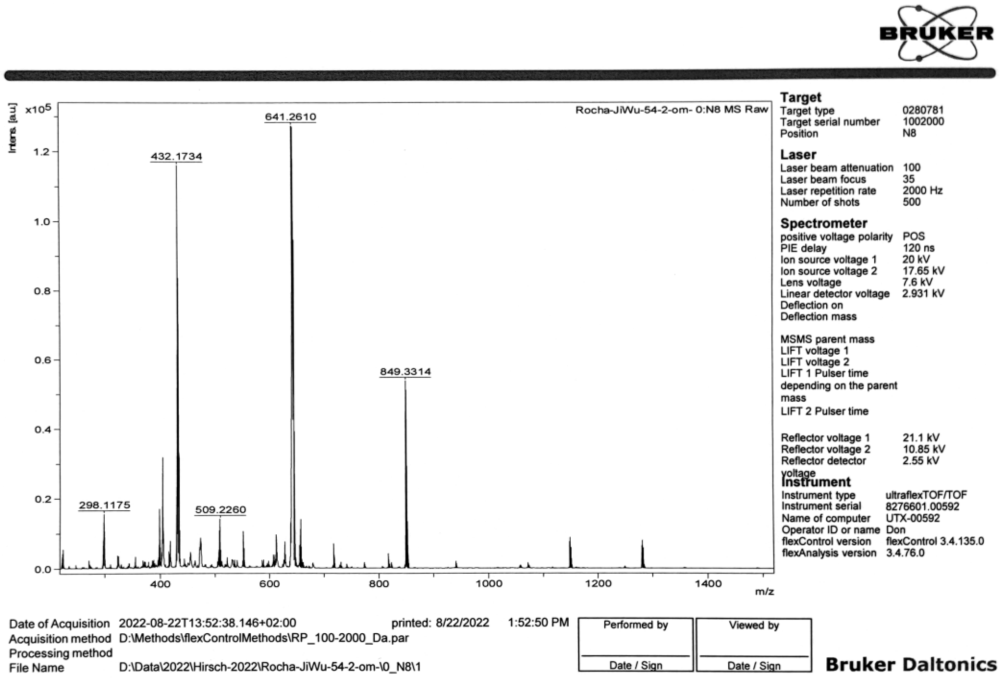

Supplementary Figure 25. HRMS of sample 54 (A30B2, Batch 2).

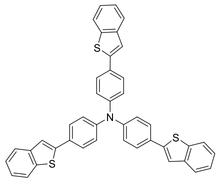

54
Batch 3

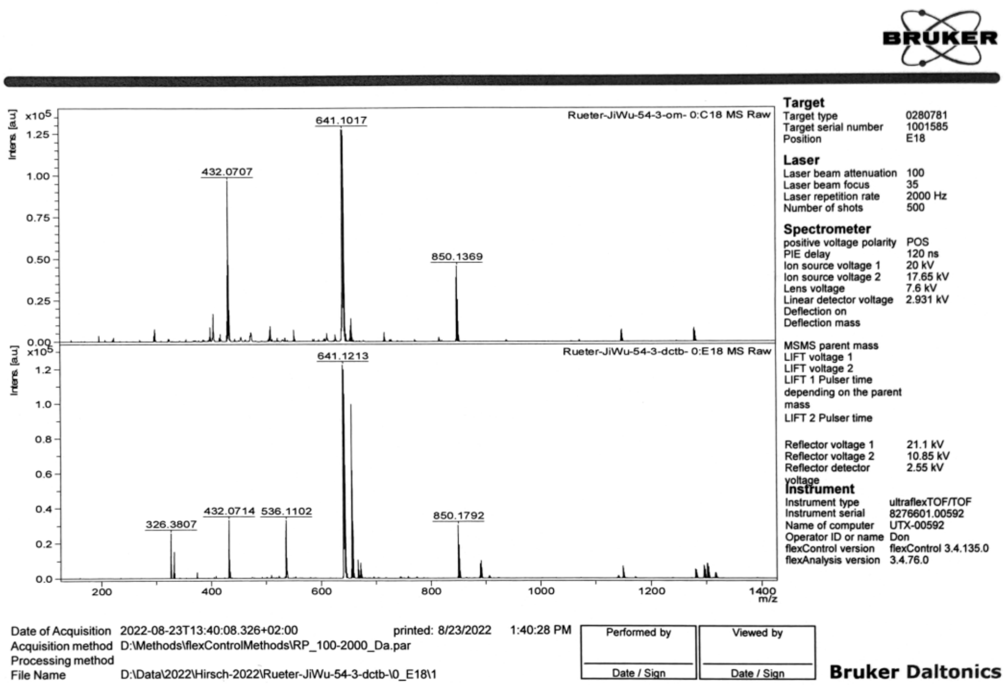

Supplementary Figure 26. HRMS of sample 54 (A30B2, Batch 3).

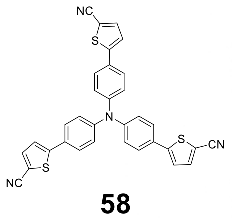

58
Batch 1

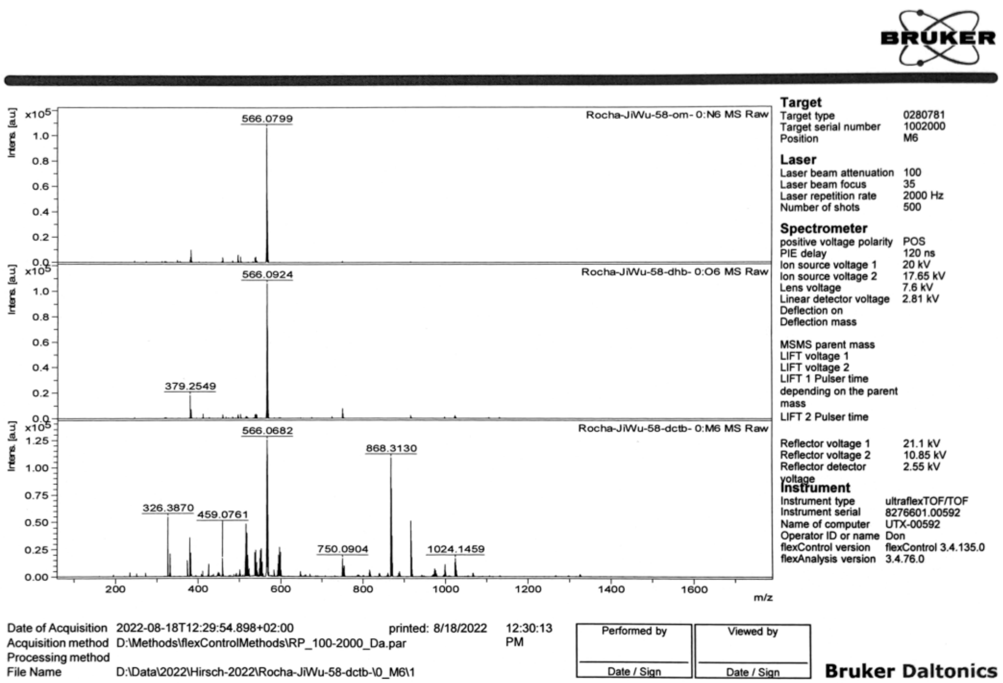

Supplementary Figure 27. HRMS of sample 58 (A30B3, Batch 1).

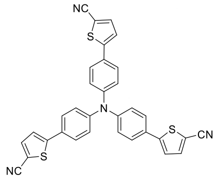

58
Batch 2

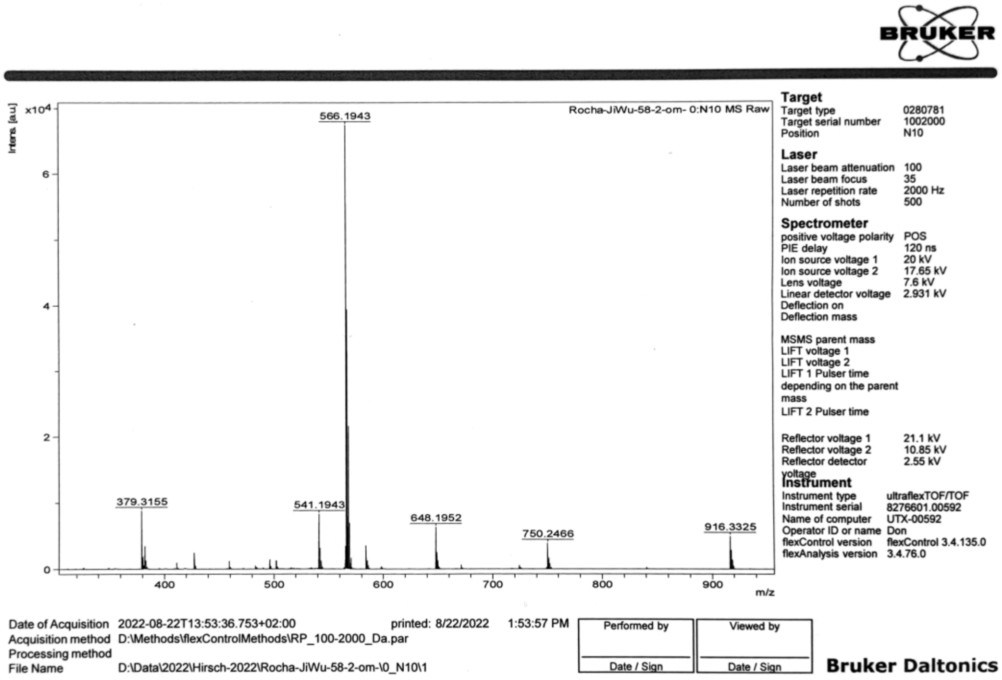

Supplementary Figure 28. HRMS of sample 58 (A30B3, Batch 2).

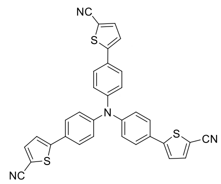

58
Batch 3

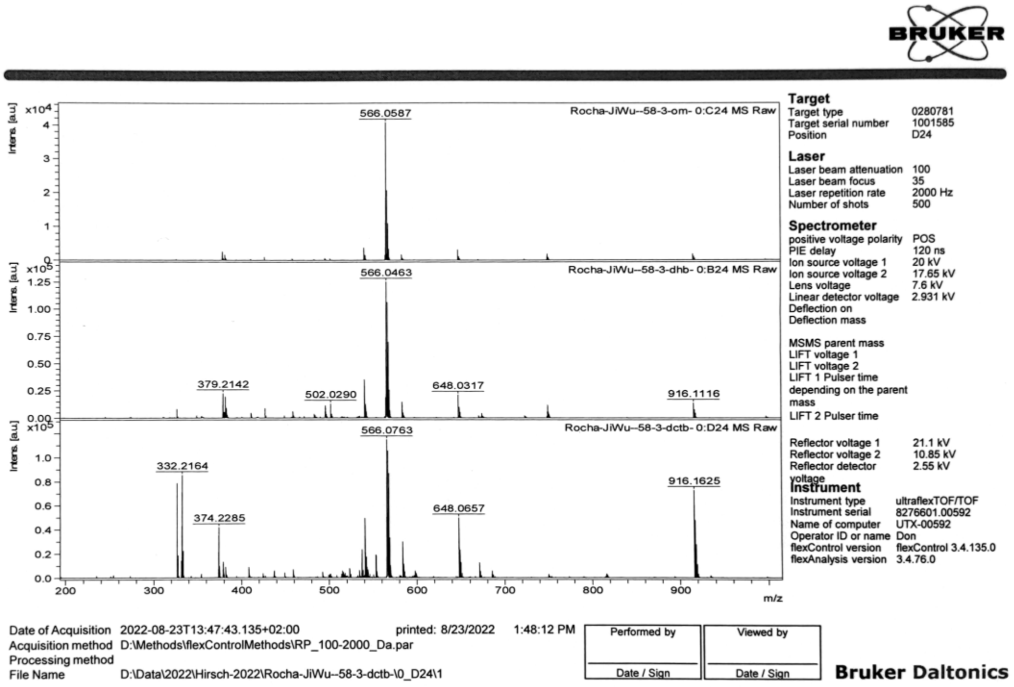

Supplementary Figure 29. HRMS of sample 58 (A30B3, Batch 3).

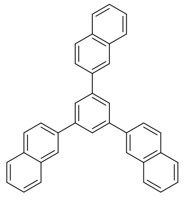

79
Batch 1

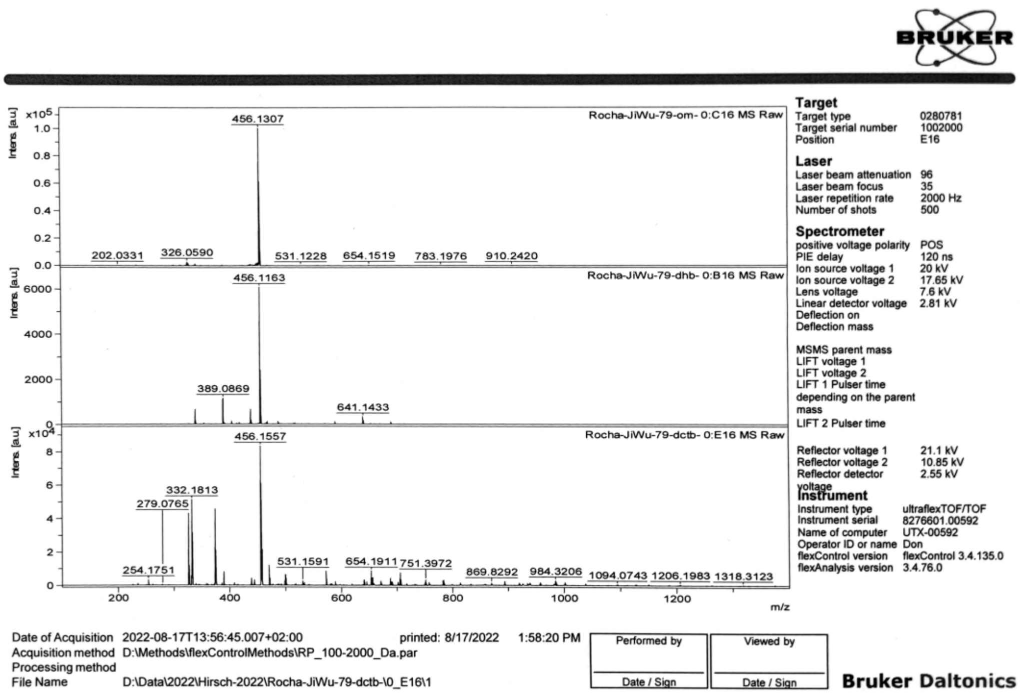

Supplementary Figure 30. HRMS of sample 79 (A31B8, Batch 1).

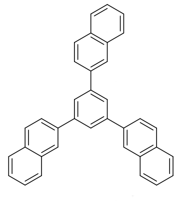

79
Batch 2

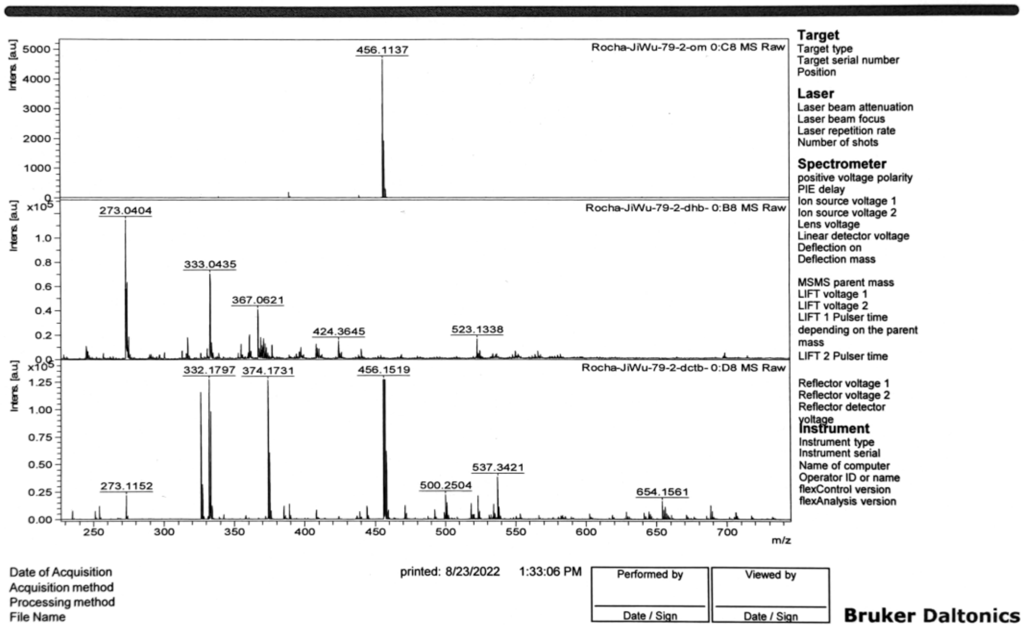

Supplementary Figure 31. HRMS of sample 79 (A31B8, Batch 2).

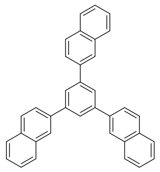

79
Batch 3

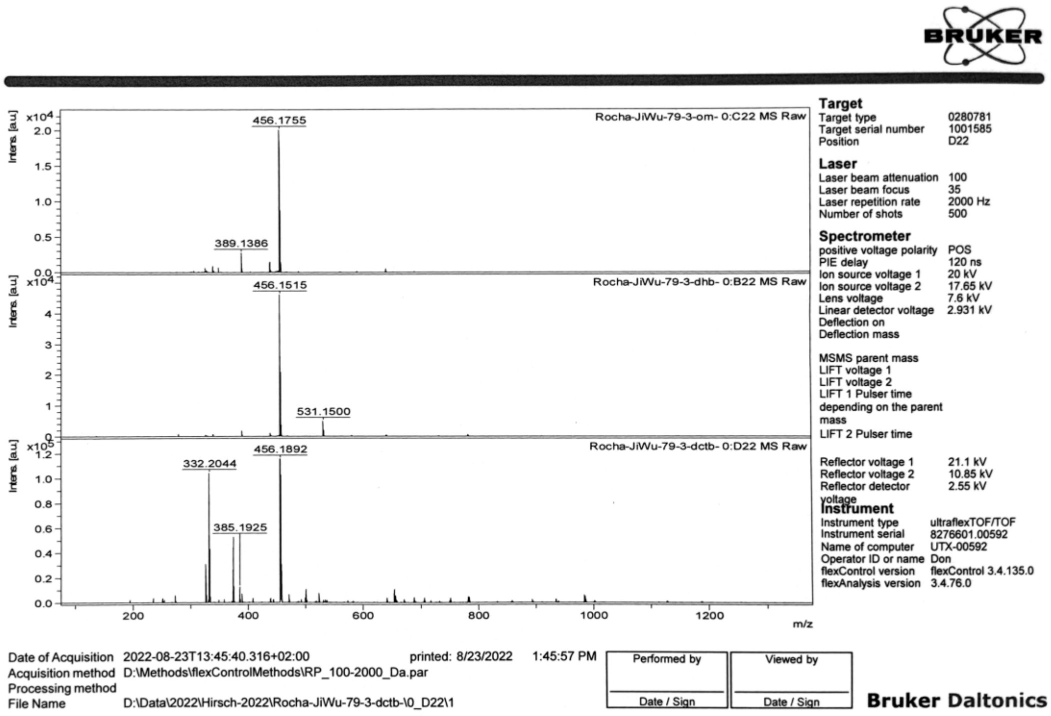

Supplementary Figure 32. HRMS of sample 79 (A31B8, Batch 3).

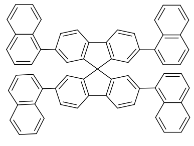

84
Batch 1

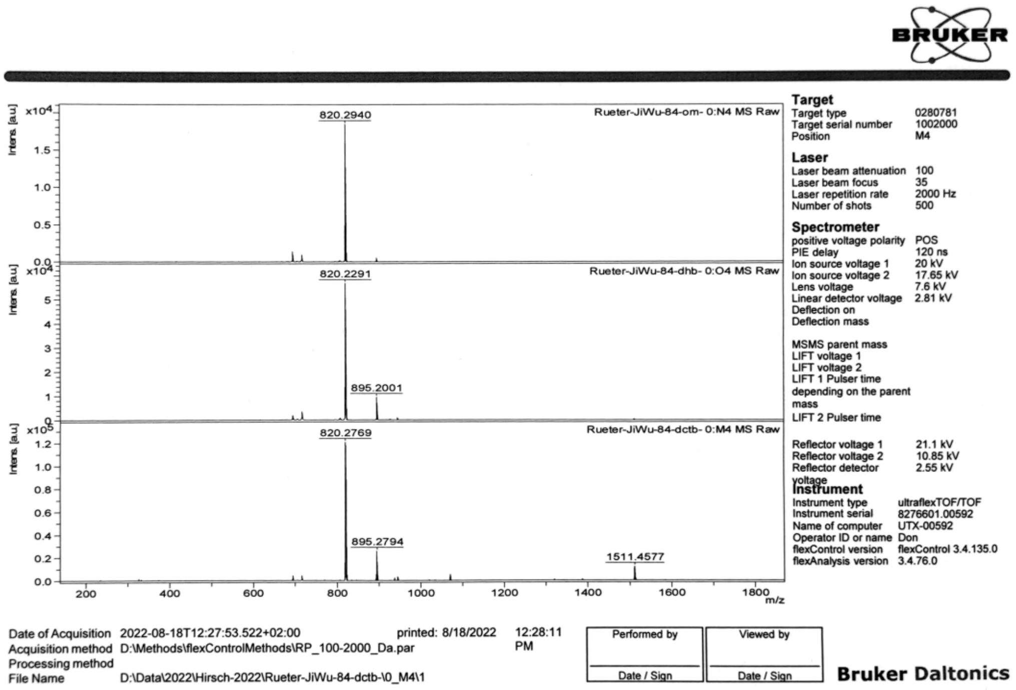

Supplementary Figure 33. HRMS of sample 84 (A32B9, Batch 1).

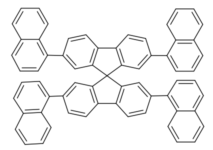

84
Batch 2

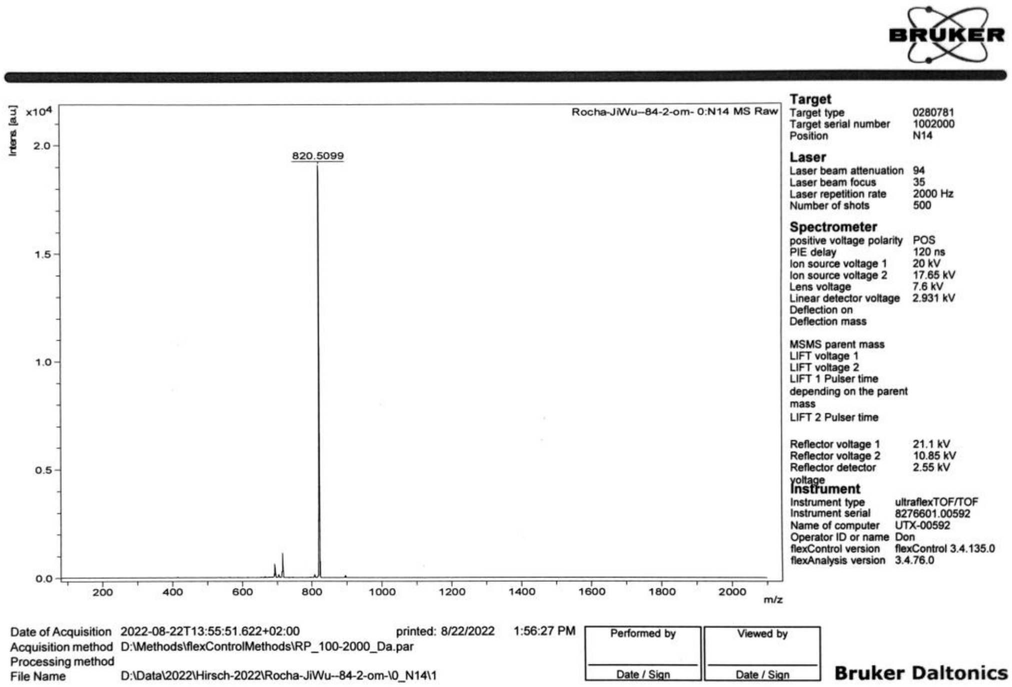

Supplementary Figure 34. HRMS of sample 84 (A32B9, Batch 2).

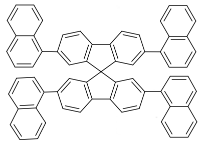

84
Batch 3

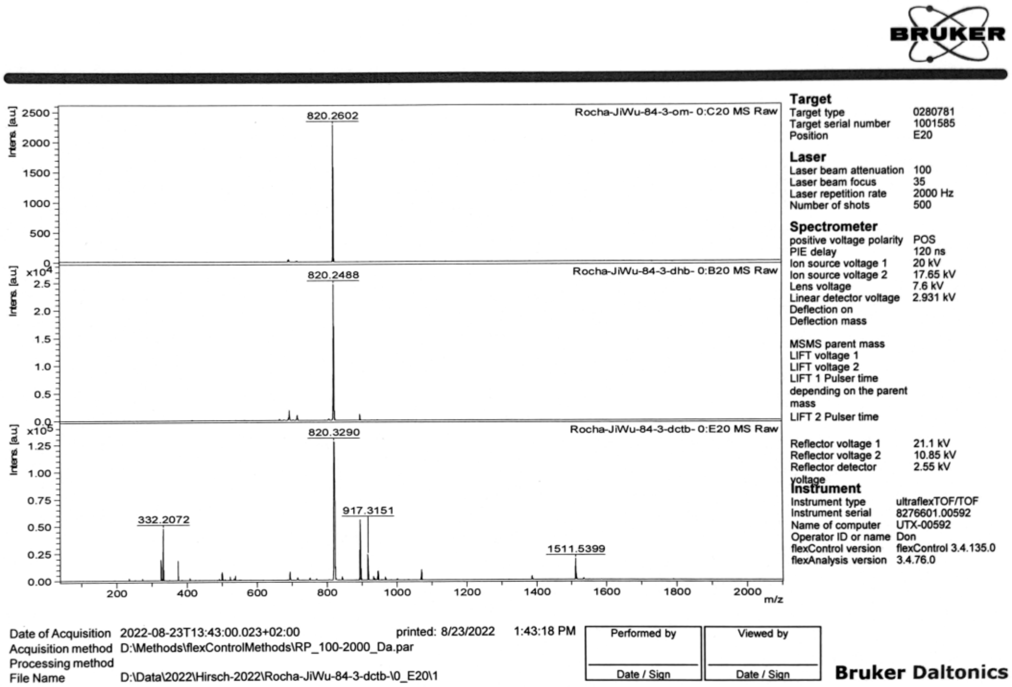

Supplementary Figure 35. HRMS of sample 84 (A32B9, Batch 3).

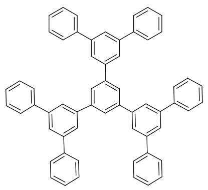

99
Batch 1

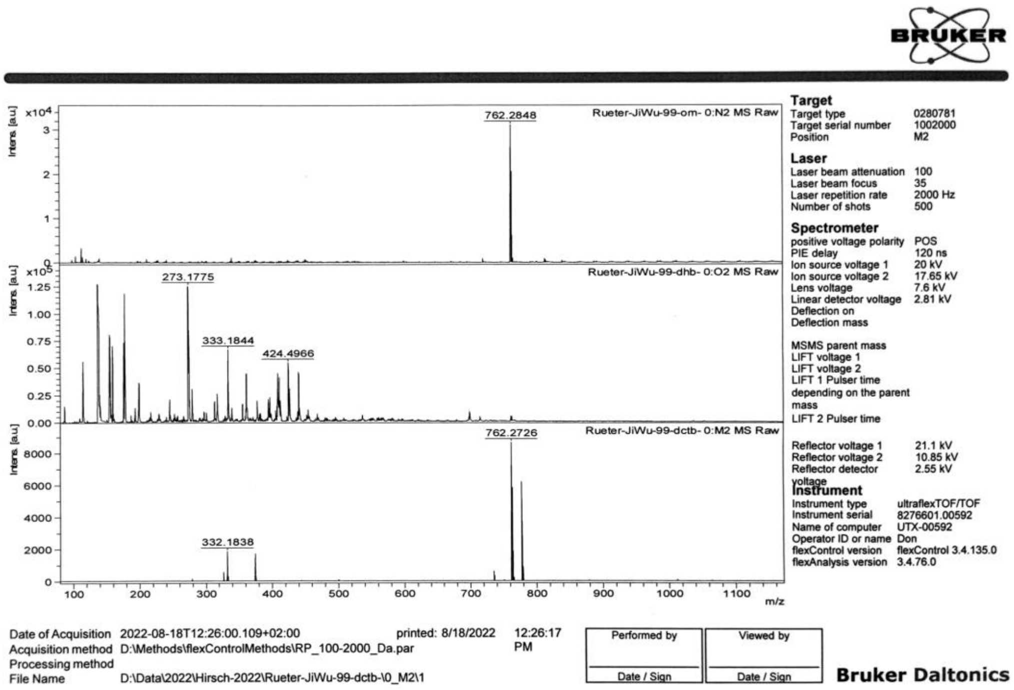

Supplementary Figure 36. HRMS of sample 99 (A31B13, Batch 1).

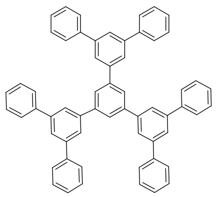

99
Batch 2

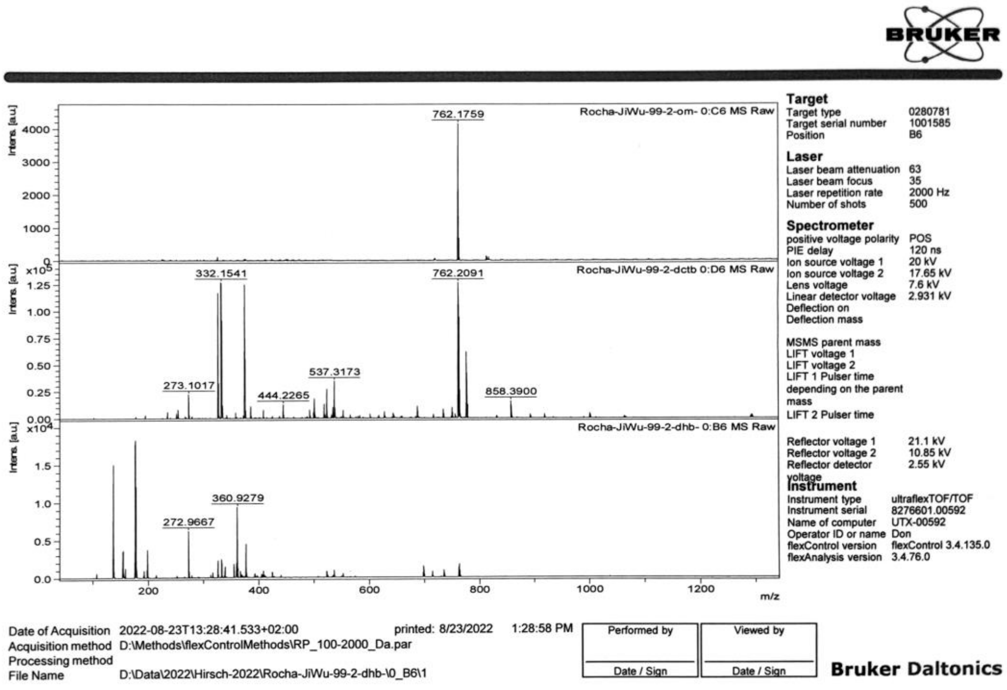

Supplementary Figure 37. HRMS of sample 99 (A31B13, Batch 2).

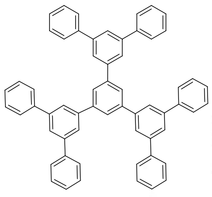

99
Batch 3

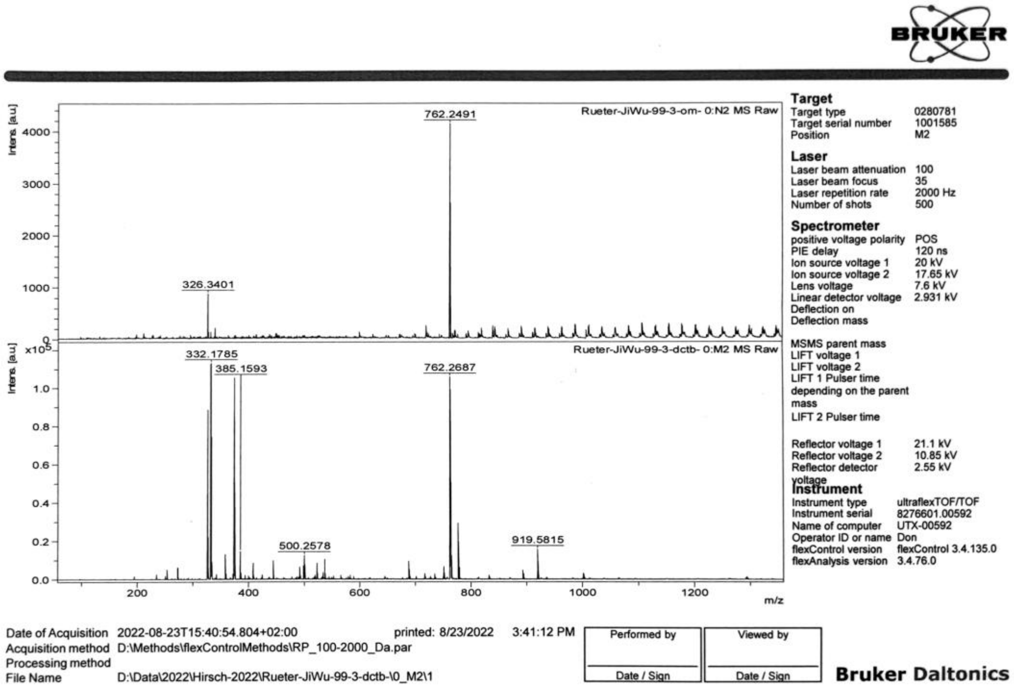

Supplementary Figure 38. HRMS of sample 99 (A31B13, Batch 3).

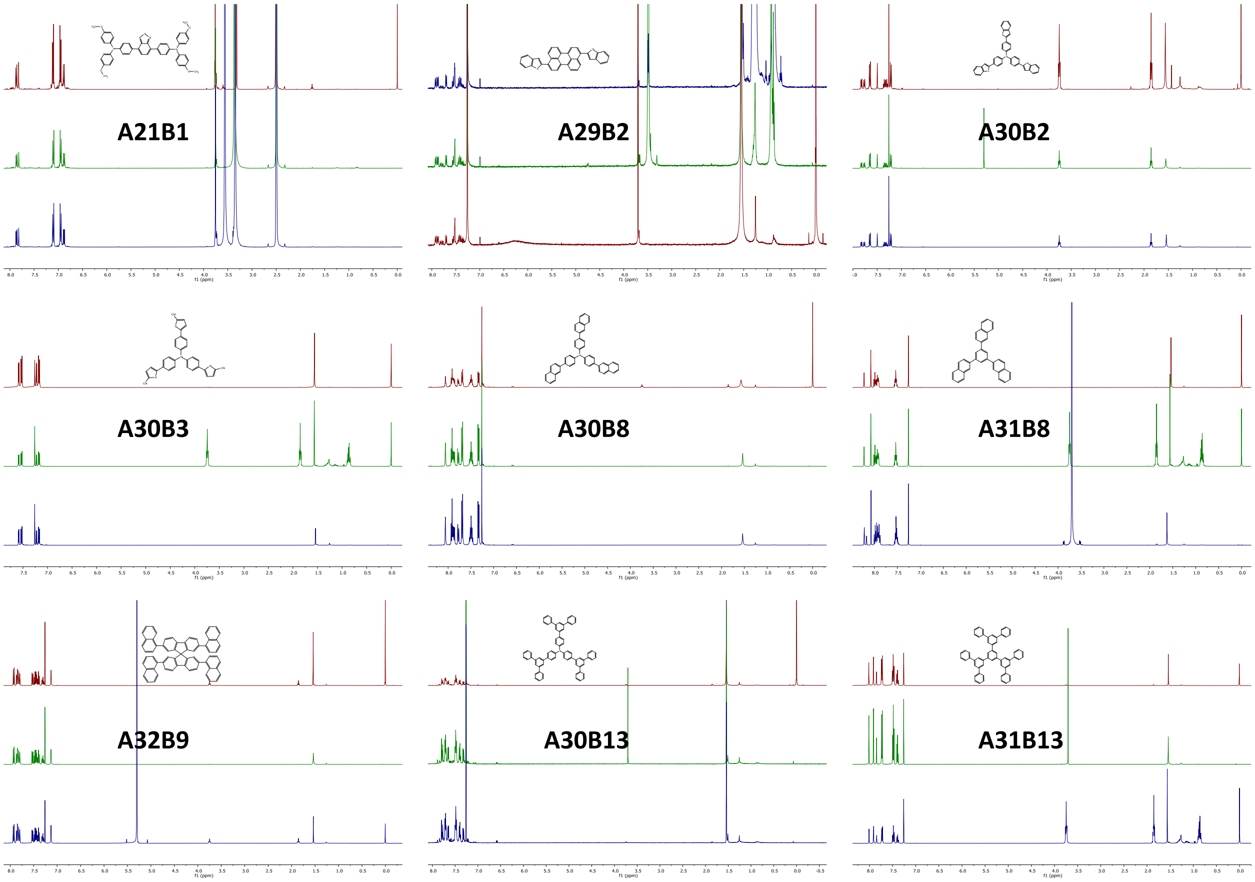

Supplementary Figure 39. Characterization of batch-to-batch repeatability. ^{1}H NMR of selected molecules in three batches.

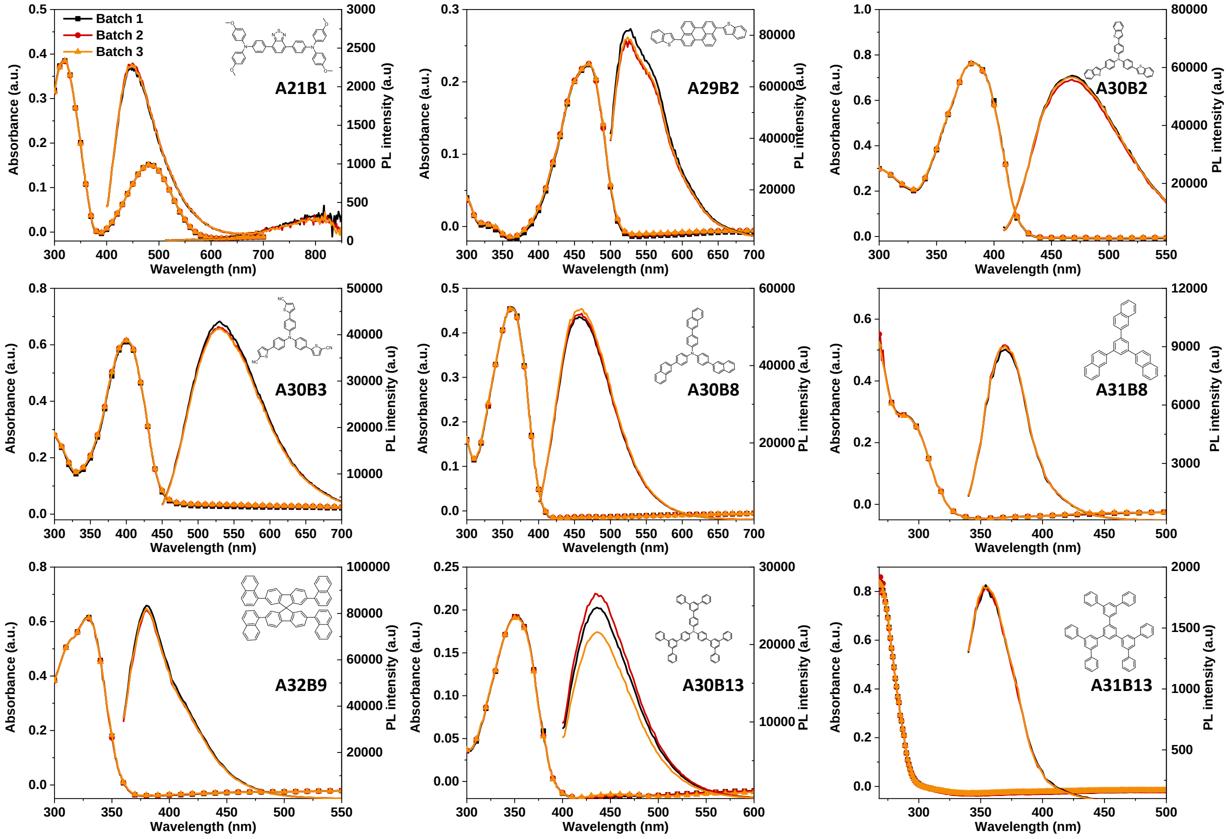

Supplementary Figure 40. Characterization of batch-to-batch repeatability. UV-Vis absorption and PL of selected molecules in three batches.

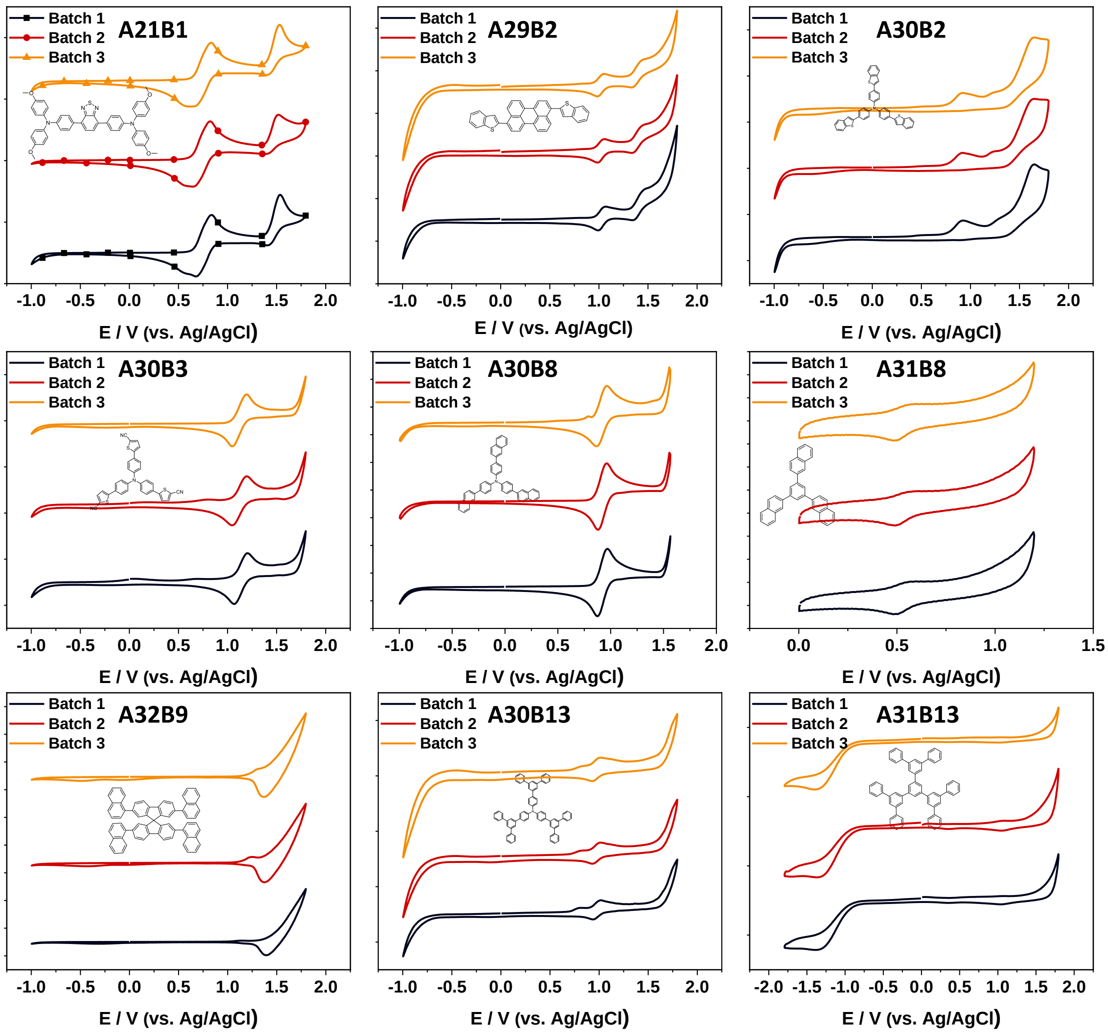

Supplementary Figure 41. Characterization of batch-to-batch repeatability. CV of selected molecules in three batches.

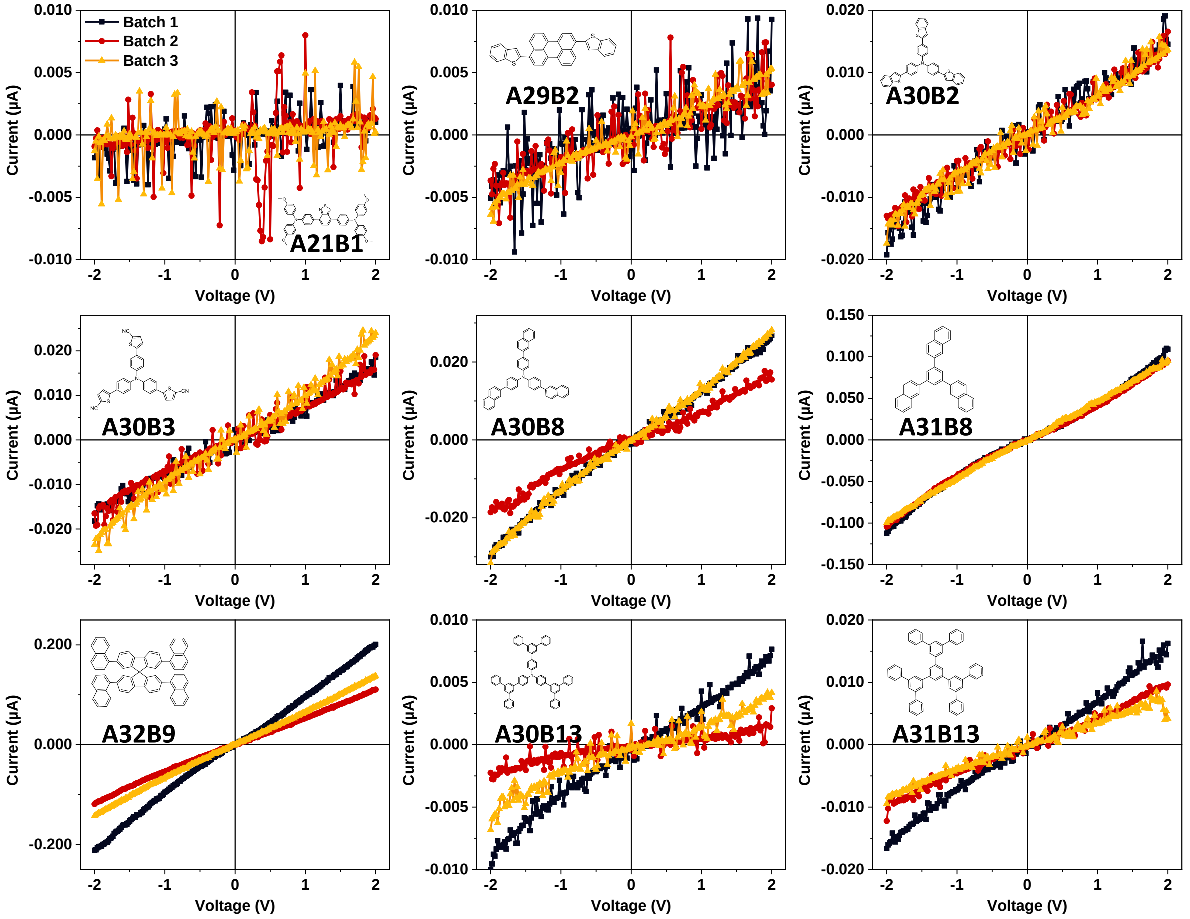

Supplementary Figure 42. Characterization of batch-to-batch repeatability. Conductivity test of selected molecules in three batches.

6. NMR of all synthesized molecules

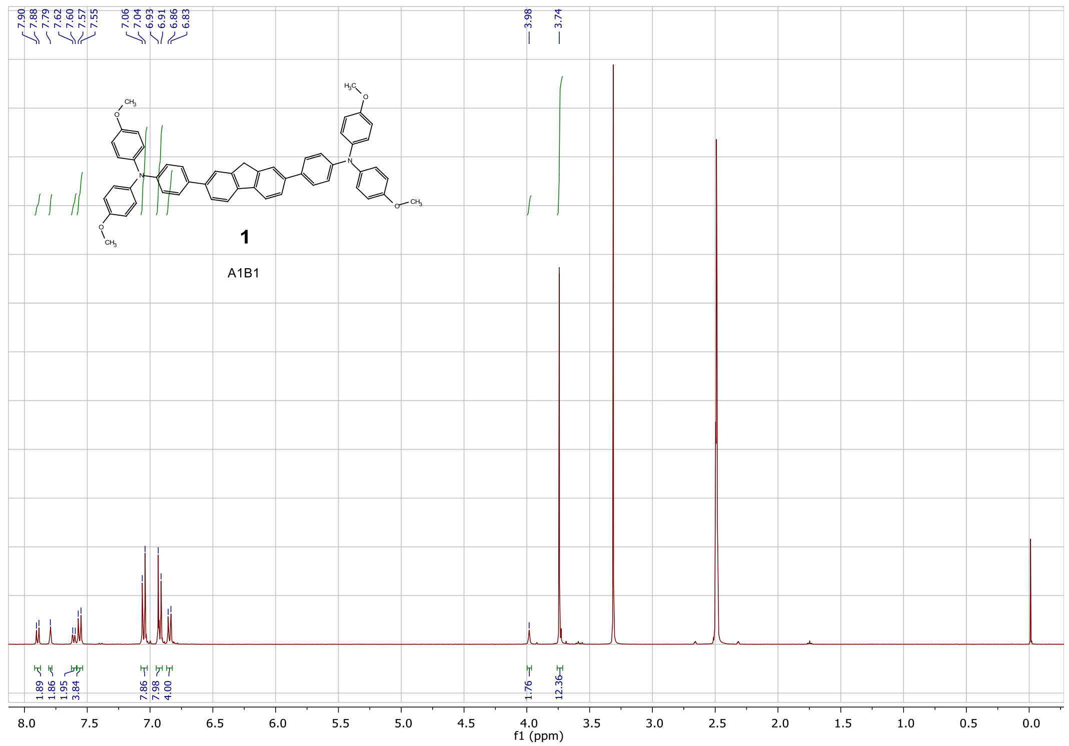

Supplementary Figure 43. ^{1}H NMR of **compound 1 (A1B1)** in d_6-DMSO.

^{1}H NMR (400 MHz, DMSO-d_6) δ 7.89 (d, *J* = 8.0 Hz, 2H), 7.79 (s, 2H), 7.61 (d, *J* = 8.1 Hz, 2H), 7.56 (d, *J* = 8.9 Hz, 4H), 7.05 (d, *J* = 9.0 Hz, 8H), 6.92 (d, *J* = 9.1 Hz, 8H), 6.84 (d, *J* = 8.8 Hz, 4H), 3.98 (s, 2H), 3.74 (s, 12H).

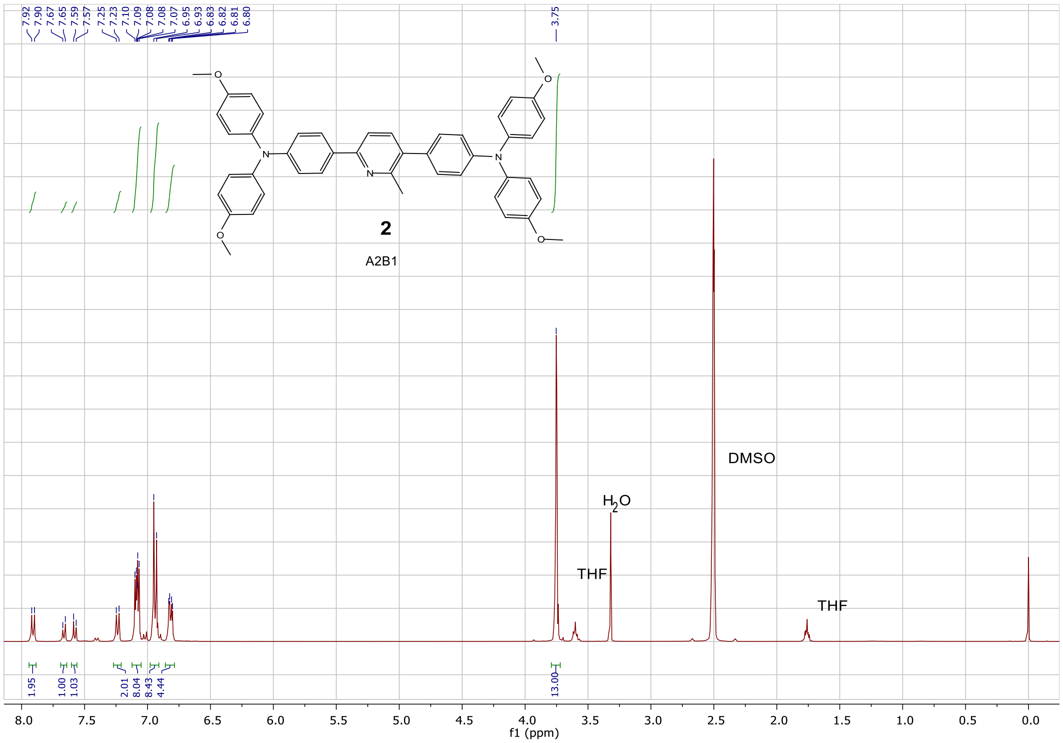

Supplementary Figure 44. ^{1}H NMR of **compound 2 (A2B1)** in d_6-DMSO.

^{1}H NMR (400 MHz, DMSO-d_6) δ 7.91 (d, *J* = 8.8 Hz, 2H), 7.66 (d, *J* = 8.1 Hz, 1H), 7.58 (d, *J* = 8.1 Hz, 1H), 7.24 (d, *J* = 8.6 Hz, 2H), 7.12 – 7.05 (m, 8H), 6.94 (d, *J* = 8.9 Hz, 8H), 6.86 – 6.79 (m, 4H), 3.75 (s, 13H).

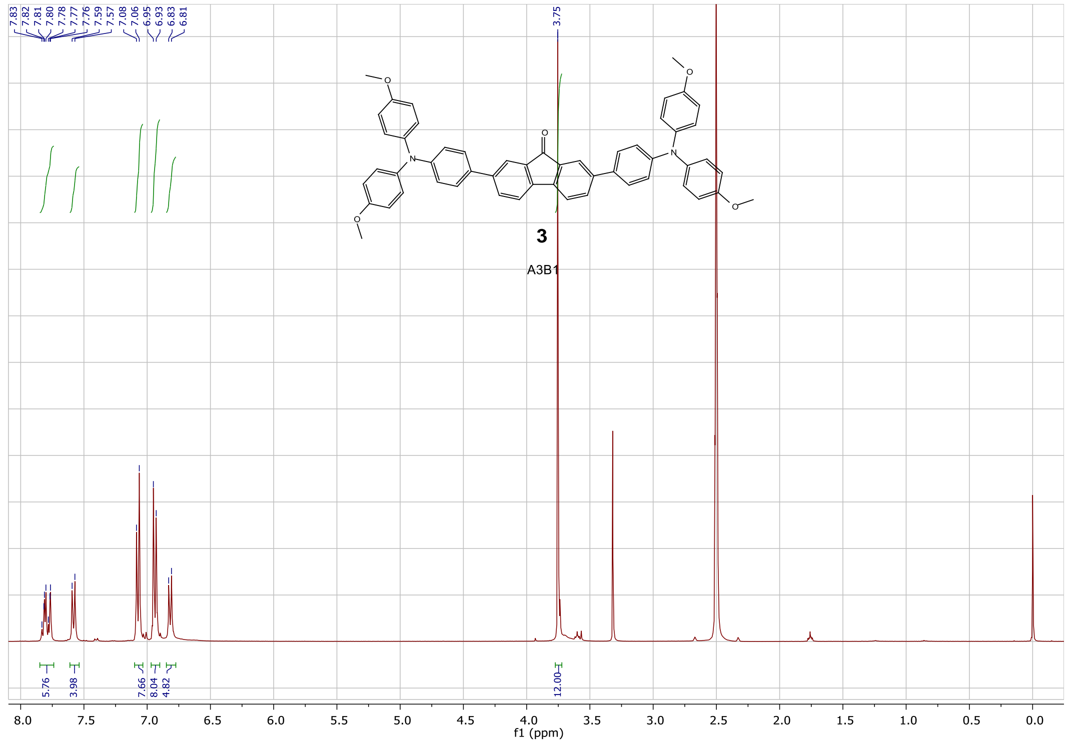

Supplementary Figure 45. ^{1}H NMR of **compound 3 (A3B1)** in d_6-DMSO.

^{1}H NMR (400 MHz, DMSO-d_6) δ 7.85 – 7.74 (m, 6H), 7.58 (d, *J* = 8.8 Hz, 4H), 7.07 (d, *J* = 8.9 Hz, 8H), 6.94 (d, *J* = 9.0 Hz, 8H), 6.82 (d, *J* = 8.8 Hz, 5H), 3.75 (s, 12H).

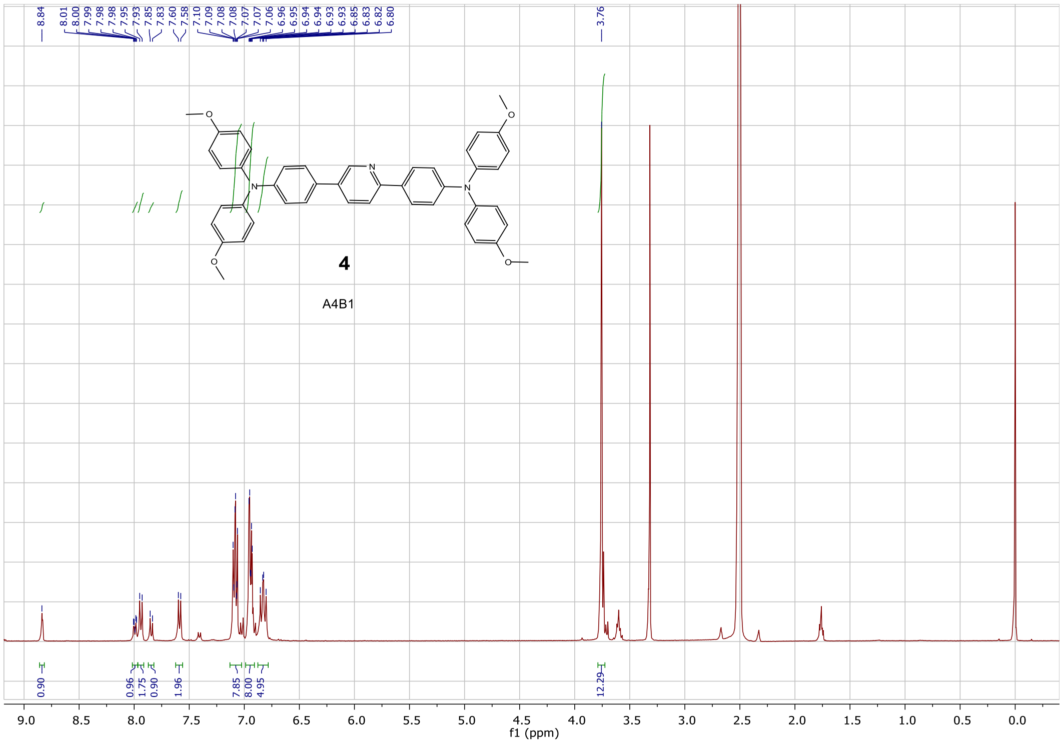

Supplementary Figure 46. ^{1}H NMR of **compound 4 (A4B1)** in d_6-DMSO.

^{1}H NMR (400 MHz, DMSO-d_6) δ 8.84 (s, 1H), 8.02 – 7.97 (m, 1H), 7.94 (d, J = 8.9 Hz, 2H), 7.84 (d, J = 8.4 Hz, 1H), 7.59 (d, J = 8.8 Hz, 2H), 7.13 – 7.02 (m, 8H), 6.99 – 6.91 (m, 8H), 6.88 – 6.78 (m, 5H), 3.76 (s, 12H).

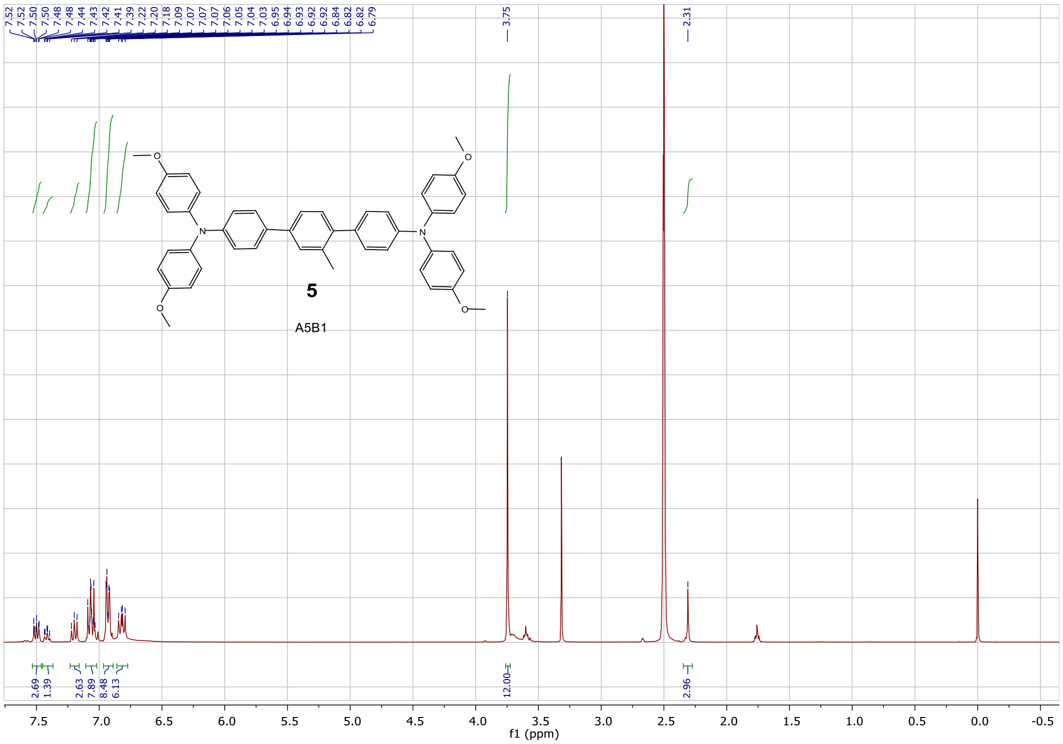

Supplementary Figure 47. ^{1}H NMR of **compound 5 (A5B1)** in d_6-DMSO.

^{1}H NMR (400 MHz, DMSO-d_6) δ 7.53 – 7.46 (m, 3H), 7.45 – 7.37 (m, 1H), 7.23 – 7.16 (m, 3H), 7.11 – 7.02 (m, 8H), 6.97 – 6.89 (m, 8H), 6.82 (dd, J = 11.9, 8.7 Hz, 6H), 3.75 (s, 13H), 2.31 (s, 3H).

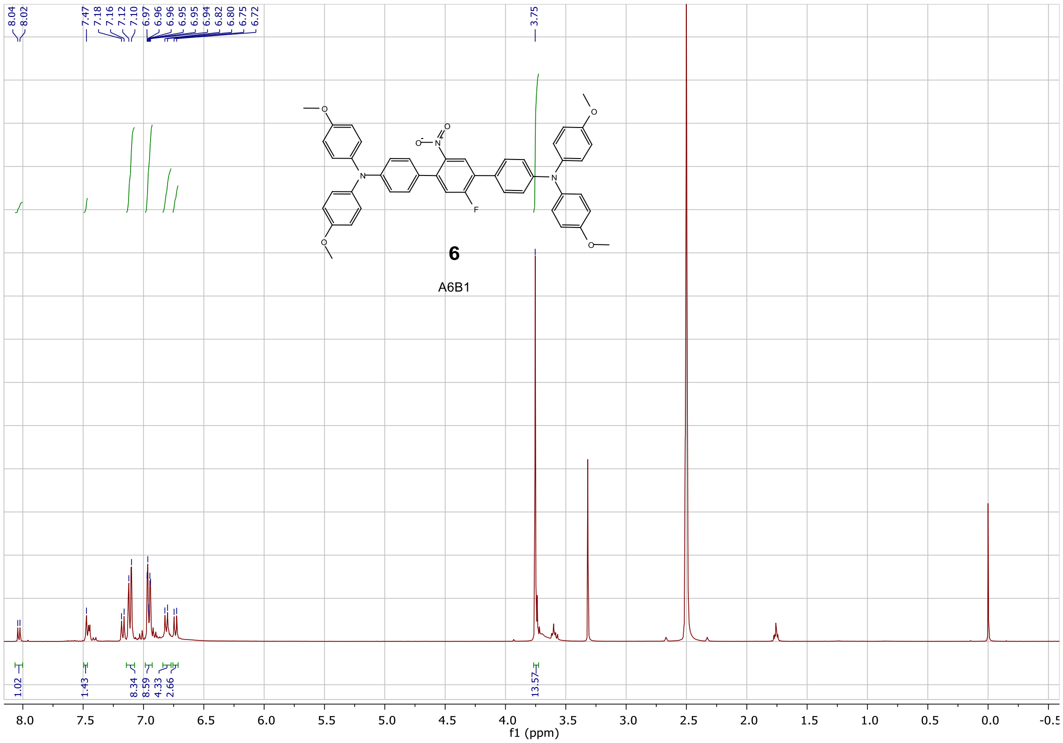

Supplementary Figure 48. ^{1}H NMR of **compound 6 (A6B1)** in d_6-DMSO.

^{1}H NMR (400 MHz, DMSO-d_6) δ 8.03 (d, J = 7.3 Hz, 1H), 7.47 (s, 1H), 7.11 (d, J = 8.8 Hz, 8H), 6.98 – 6.93 (m, 9H), 6.81 (d, J = 8.8 Hz, 4H), 6.74 (d, J = 8.7 Hz, 3H), 3.75 (s, 14H).

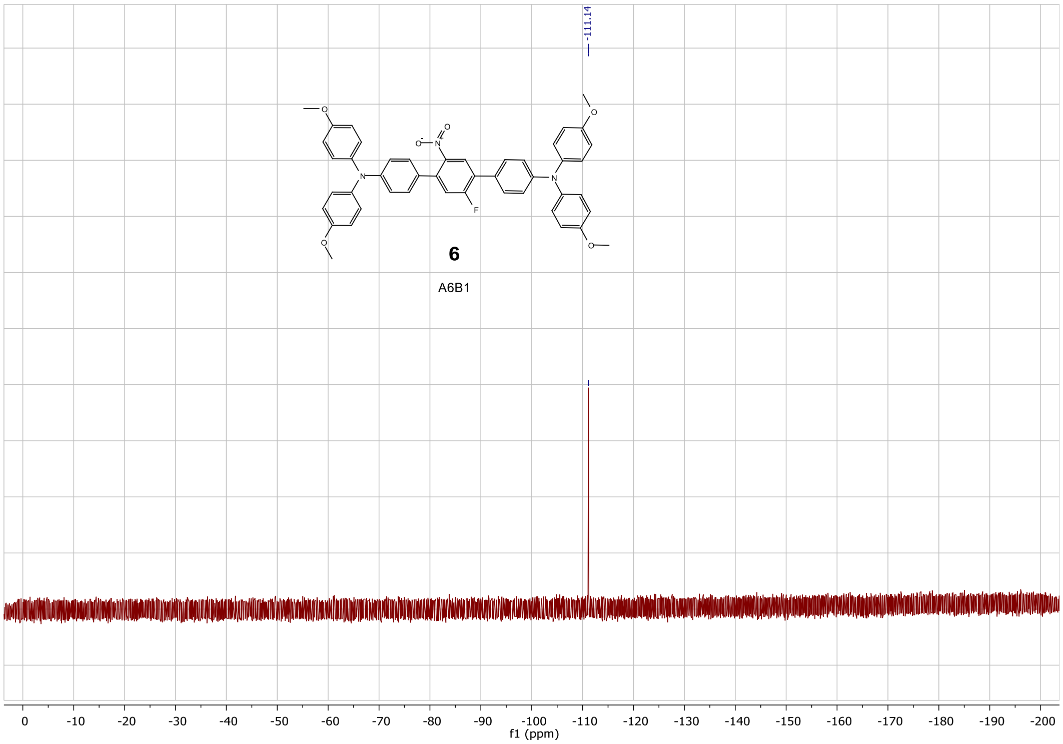

Supplementary Figure 49. ^{19}F NMR of **compound 6 (A6B1)** in *d6*-DMSO.

^{19}F NMR (377 MHz, DMSO-d_6) δ -111.14.

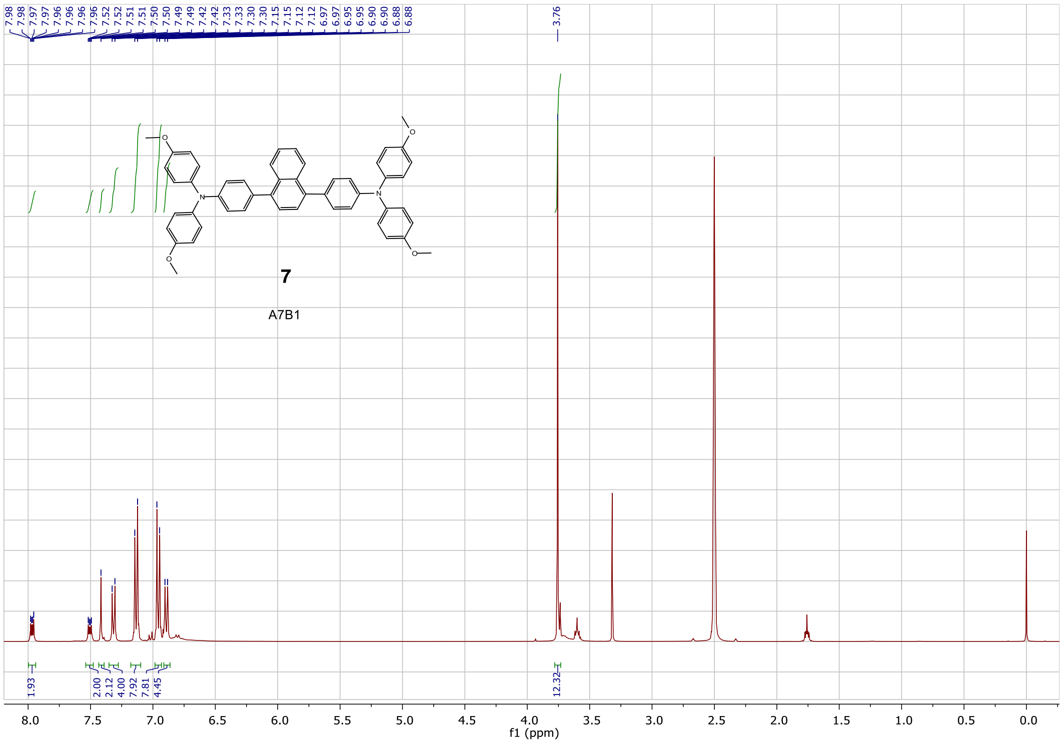

Supplementary Figure 50. ^{1}H NMR of **compound 7 (A7B1)** in d_6-DMSO.

^{1}H NMR (400 MHz, DMSO-d_6) δ 7.97 (dd, *J* = 6.5, 3.4 Hz, 2H), 7.51 (dd, *J* = 6.5, 3.4 Hz, 2H), 7.42 (s, 2H), 7.32 (d, *J* = 8.6 Hz, 4H), 7.13 (d, *J* = 8.9 Hz, 8H), 6.96 (d, *J* = 9.0 Hz, 8H), 6.89 (d, *J* = 8.6 Hz, 4H), 3.76 (s, 15H).

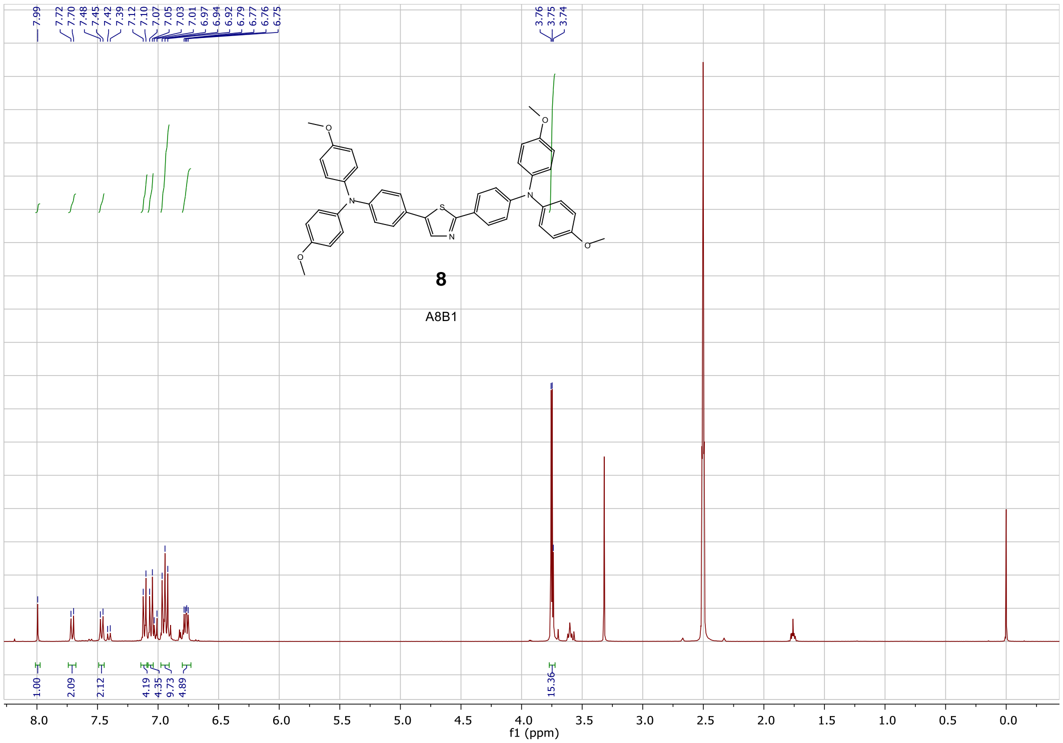

Supplementary Figure 51. ^{1}H NMR of **compound 8 (A8B1)** in d_6-DMSO.

^{1}H NMR (400 MHz, DMSO-d_6) δ 7.99 (s, 1H), 7.71 (d, J = 8.8 Hz, 2H), 7.47 (d, J = 8.7 Hz, 2H), 7.11 (d, J = 8.9 Hz, 4H), 7.06 (d, J = 8.9 Hz, 4H), 6.98 – 6.91 (m, 10H), 6.77 (dd, J = 8.8, 4.8 Hz, 5H), 3.77 – 3.72 (m, 15H).

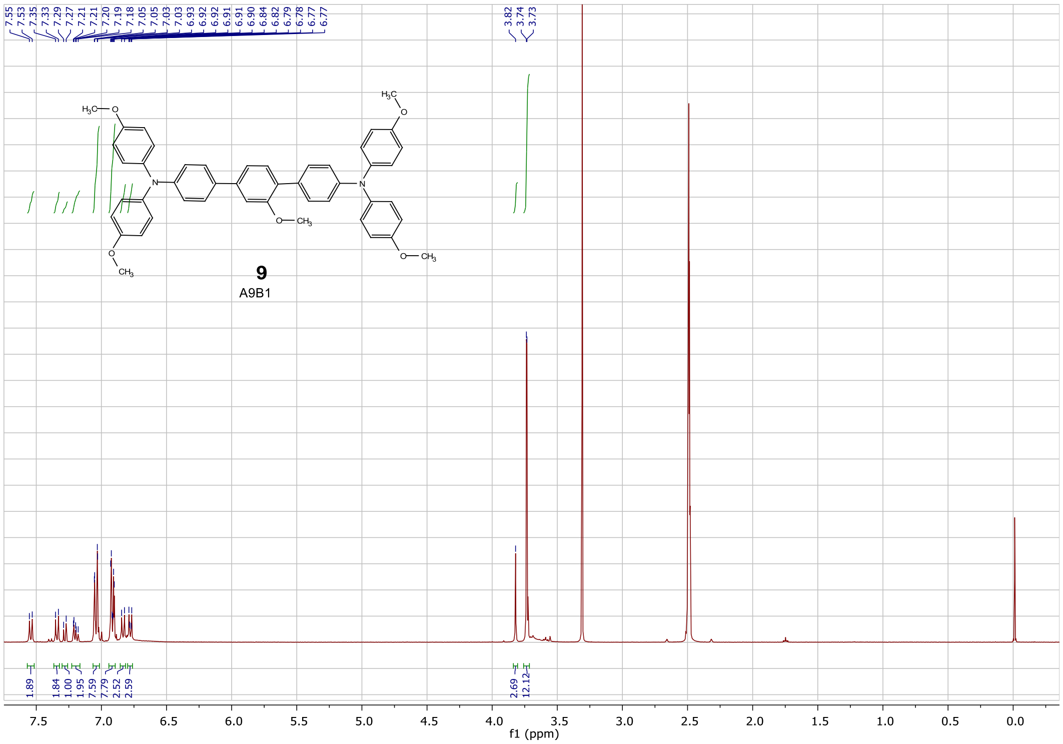

Supplementary Figure 52. ^{1}H NMR of **compound 9 (A9B1)** in d_6-DMSO.

^{1}H NMR (400 MHz, DMSO-d_6) δ 7.54 (d, J = 8.8 Hz, 2H), 7.34 (d, J = 8.8 Hz, 2H), 7.28 (d, J = 7.8 Hz, 1H), 7.23 – 7.16 (m, 2H), 7.04 (d, J = 8.2 Hz, 8H), 6.94 – 6.89 (m, 8H), 6.83 (d, J = 8.8 Hz, 3H), 6.78 (d, J = 8.8 Hz, 3H), 3.82 (s, 3H), 3.74 (d, J = 1.6 Hz, 12H).

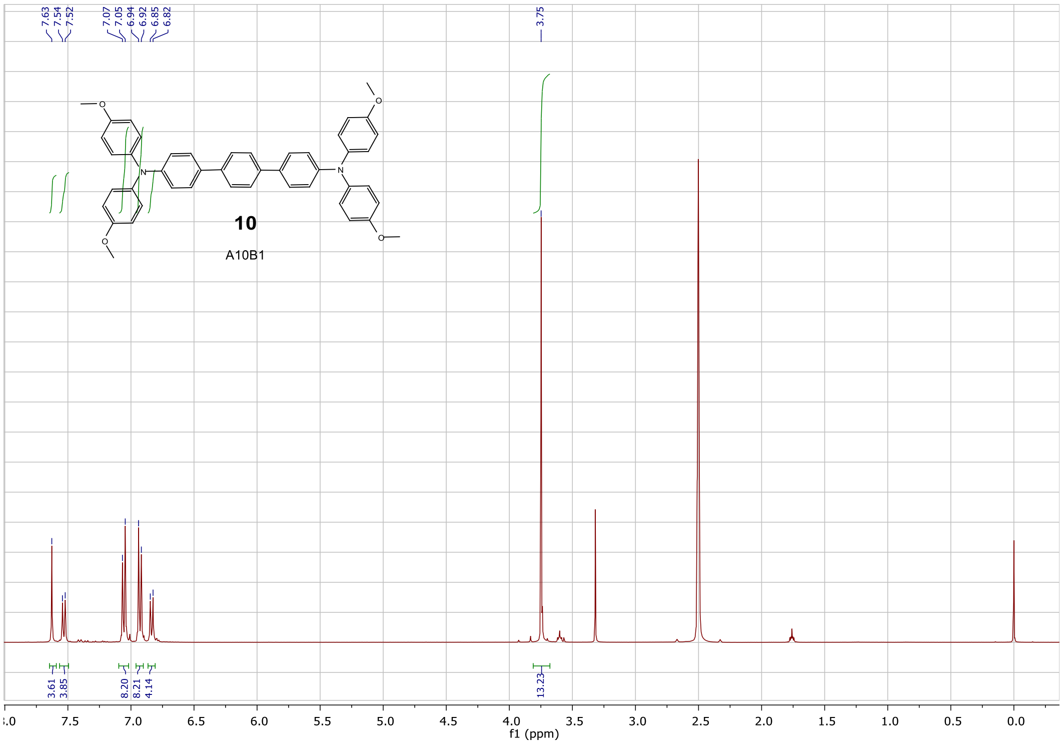

Supplementary Figure 53. ^{1}H NMR of **compound 10 (A10B1)** in *d*$_6$-DMSO.

^{1}H NMR (400 MHz, DMSO-*d*$_6$) δ 7.63 (s, 4H), 7.53 (d, *J* = 8.8 Hz, 4H), 7.06 (d, *J* = 8.9 Hz, 8H), 6.93 (d, *J* = 8.9 Hz, 8H), 6.84 (d, *J* = 8.8 Hz, 4H), 3.75 (s, 13H).

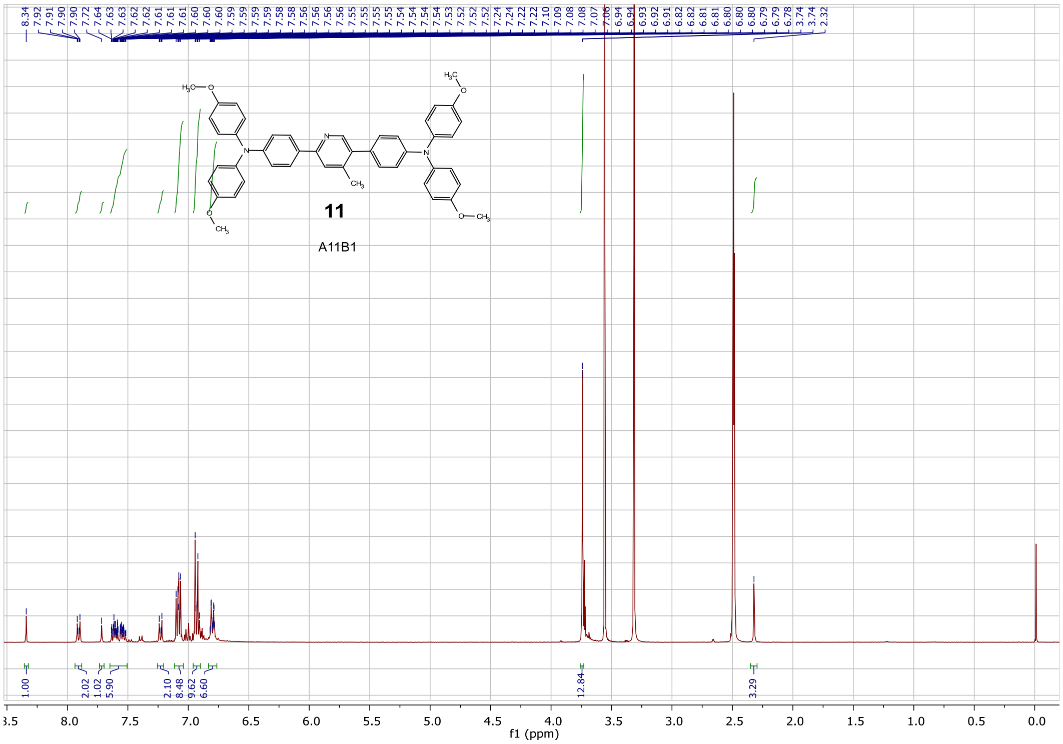

Supplementary Figure 54. ^{1}H NMR of **compound 11 (A11B1)** in d_6-DMSO.

^{1}H NMR (400 MHz, DMSO-d_6) δ 8.34 (s, 1H), 7.94 – 7.88 (m, 2H), 7.72 (s, 1H), 7.65 – 7.51 (m, 6H), 7.26 – 7.20 (m, 2H), 7.11 – 7.04 (m, 8H), 6.96 – 6.90 (m, 10H), 6.83 – 6.76 (m, 7H), 3.74 (d, J = 1.7 Hz, 13H), 2.32 (s, 3H).

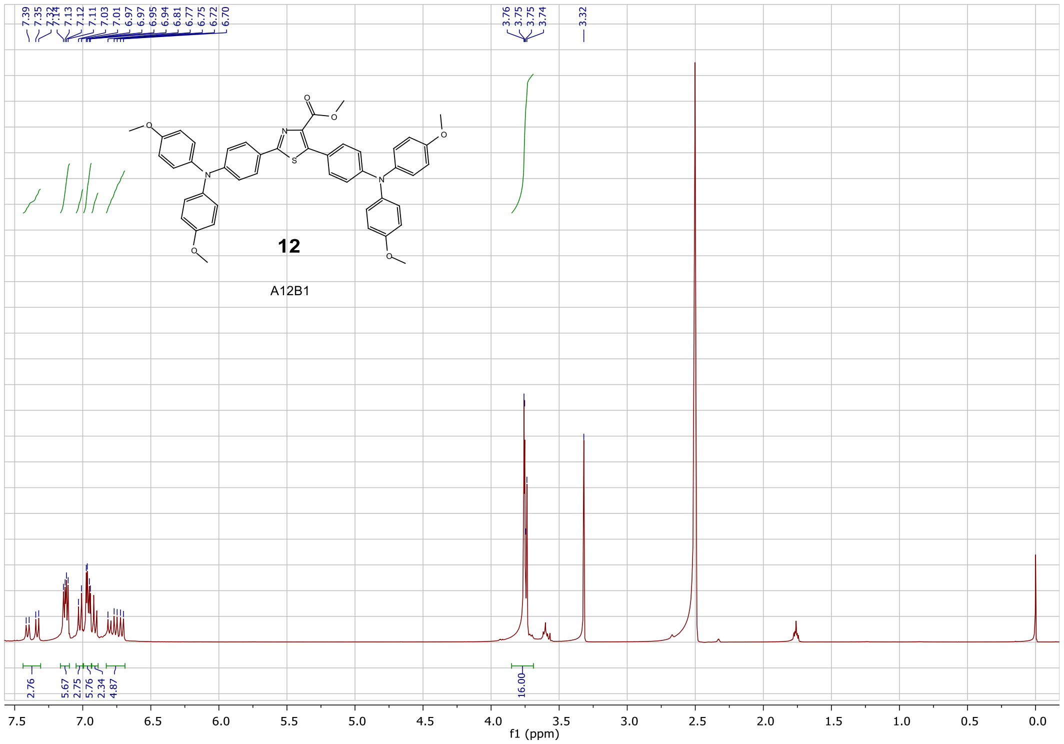

Supplementary Figure 55. ^{1}H NMR of **compound 12 (A12B1)** in d_6-DMSO.

^{1}H NMR (400 MHz, DMSO-d_6) δ 7.44 – 7.31 (m, 3H), 7.16 – 7.10 (m, 6H), 7.02 (d, J = 8.9 Hz, 3H), 6.99 – 6.94 (m, 6H), 6.93 – 6.89 (m, 2H), 6.83 – 6.69 (m, 5H), 3.85 – 3.69 (m, 16H).

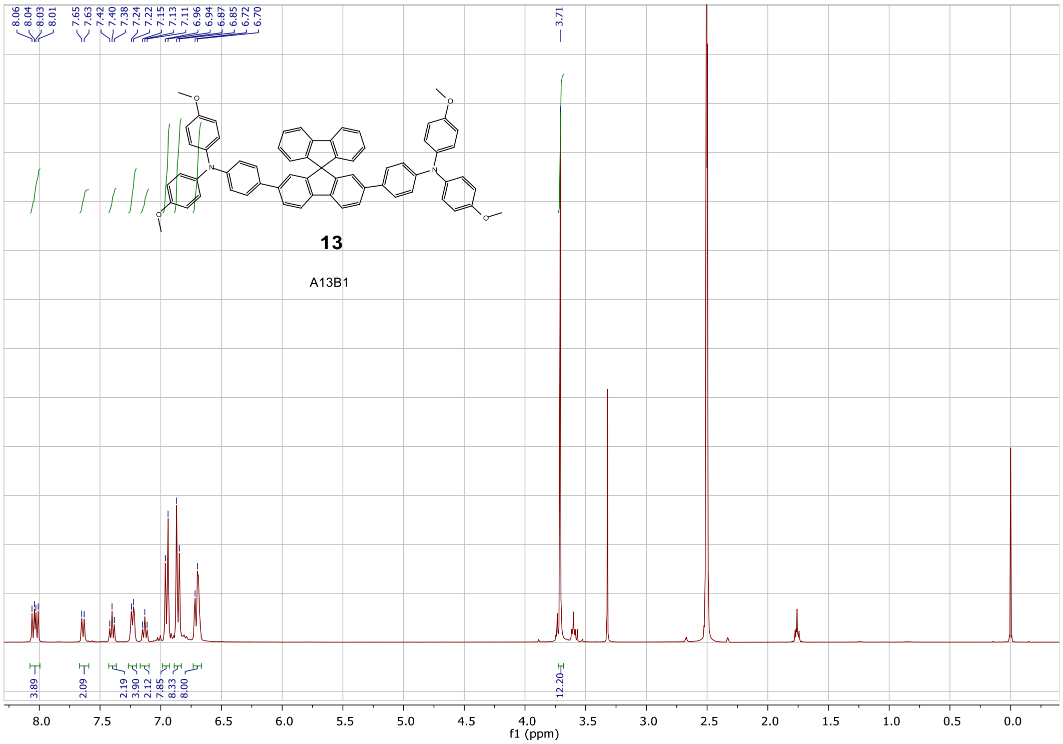

Supplementary Figure 56. ^{1}H NMR of **compound 13 (A13B1)** in d_6-DMSO.

^{1}H NMR (400 MHz, DMSO-d_6) δ 8.03 (dd, *J* = 12.6, 7.8 Hz, 4H), 7.64 (d, *J* = 8.1 Hz, 2H), 7.40 (t, *J* = 7.5 Hz, 2H), 7.23 (d, *J* = 6.9 Hz, 4H), 7.13 (t, *J* = 7.5 Hz, 2H), 6.95 (d, *J* = 8.8 Hz, 8H), 6.86 (d, *J* = 8.8 Hz, 8H), 6.71 (d, *J* = 8.5 Hz, 8H), 3.71 (s, 12H).

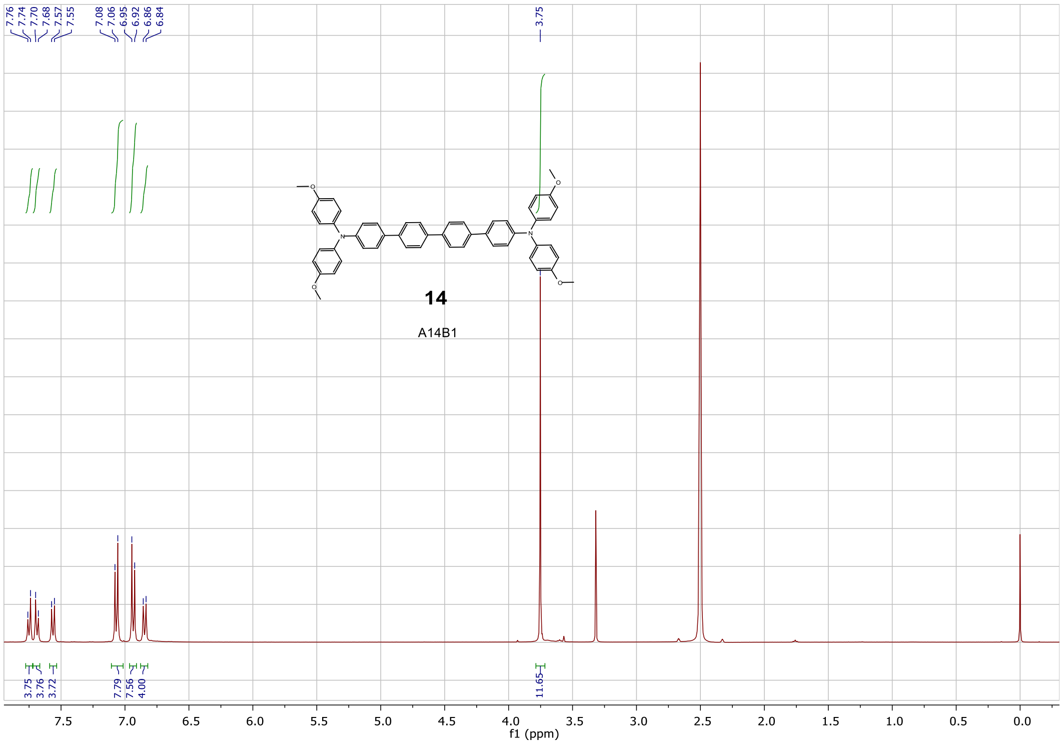

Supplementary Figure 57. ^{1}H NMR of **compound 14 (A14B1)** in d_6-DMSO.

^{1}H NMR (400 MHz, DMSO-d_6) δ 7.75 (d, *J* = 8.4 Hz, 4H), 7.69 (d, *J* = 8.5 Hz, 4H), 7.56 (d, *J* = 8.8 Hz, 4H), 7.07 (d, *J* = 9.0 Hz, 8H), 6.94 (d, *J* = 9.0 Hz, 8H), 6.85 (d, *J* = 8.8 Hz, 4H), 3.75 (s, 12H).

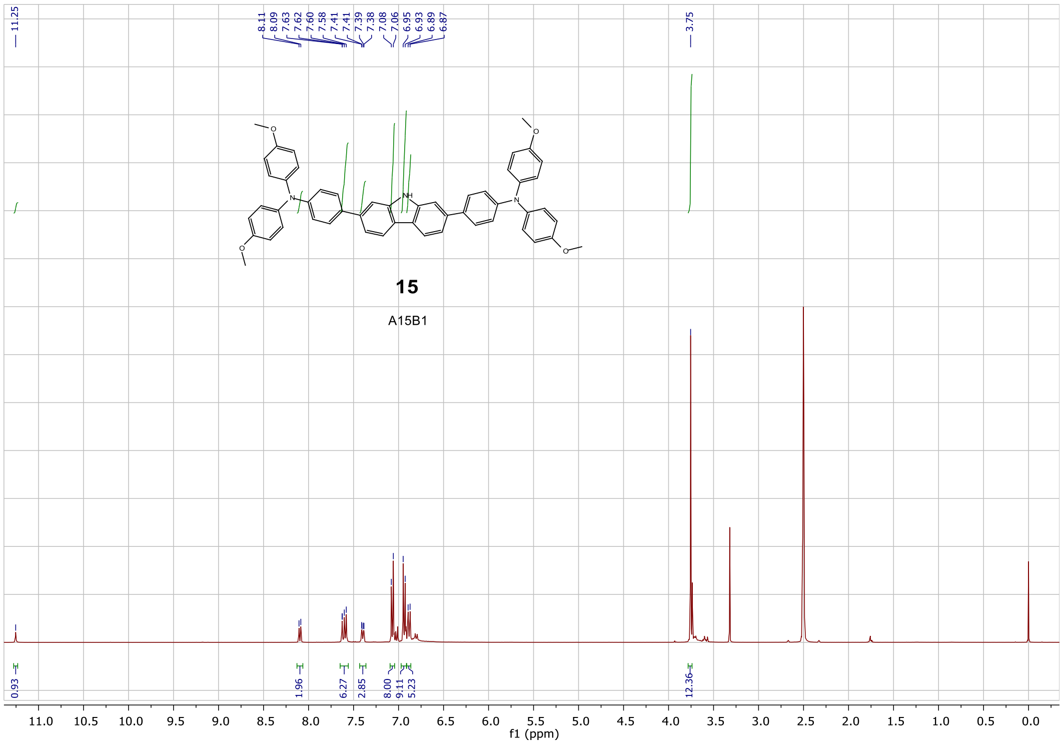

Supplementary Figure 58. ^{1}H NMR of **compound 15 (A15B1)** in d_6-DMSO.

^{1}H NMR (400 MHz, DMSO-d_6) δ 11.25 (s, 1H), 8.10 (d, J = 8.1 Hz, 2H), 7.65 – 7.56 (m, 6H), 7.40 (dd, J = 8.3, 1.6 Hz, 3H), 7.07 (d, J = 8.9 Hz, 8H), 6.94 (d, J = 9.0 Hz, 9H), 6.88 (d, J = 8.7 Hz, 5H), 3.75 (s, 12H).

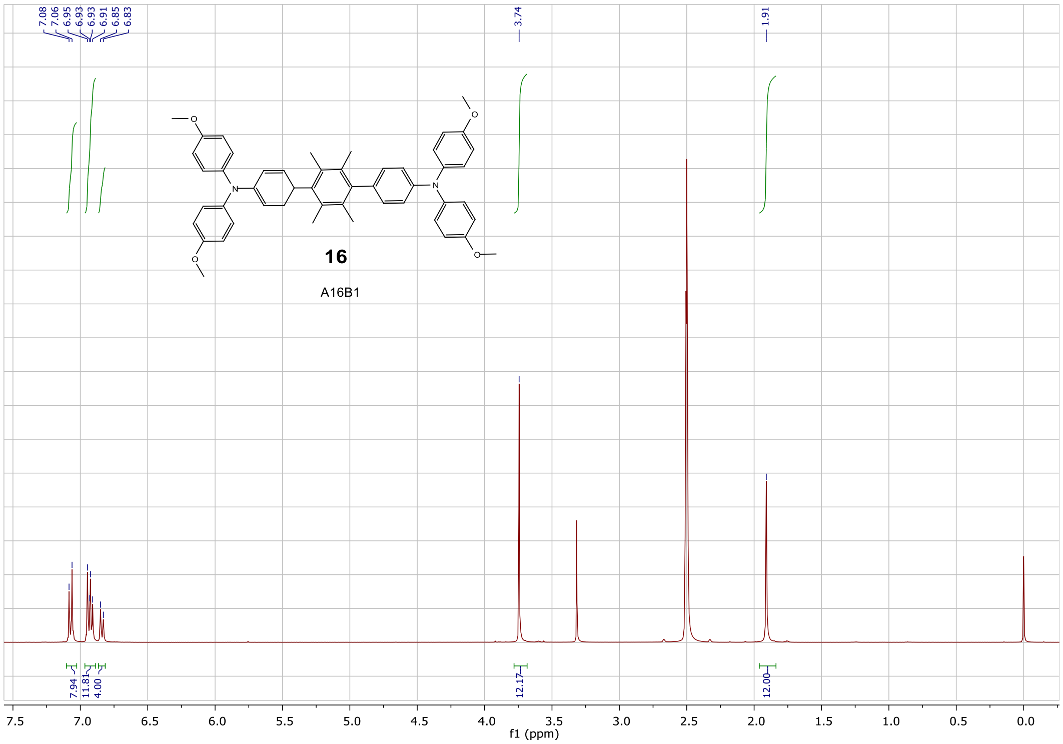

Supplementary Figure 59. ^{1}H NMR of **compound 16 (A16B1)** in d_6-DMSO.

^{1}H NMR (400 MHz, DMSO-d_6) δ 7.07 (d, J = 8.9 Hz, 8H), 6.97 – 6.89 (m, 12H), 6.84 (d, J = 8.6 Hz, 4H), 3.74 (s, 12H), 1.91 (s, 12H).

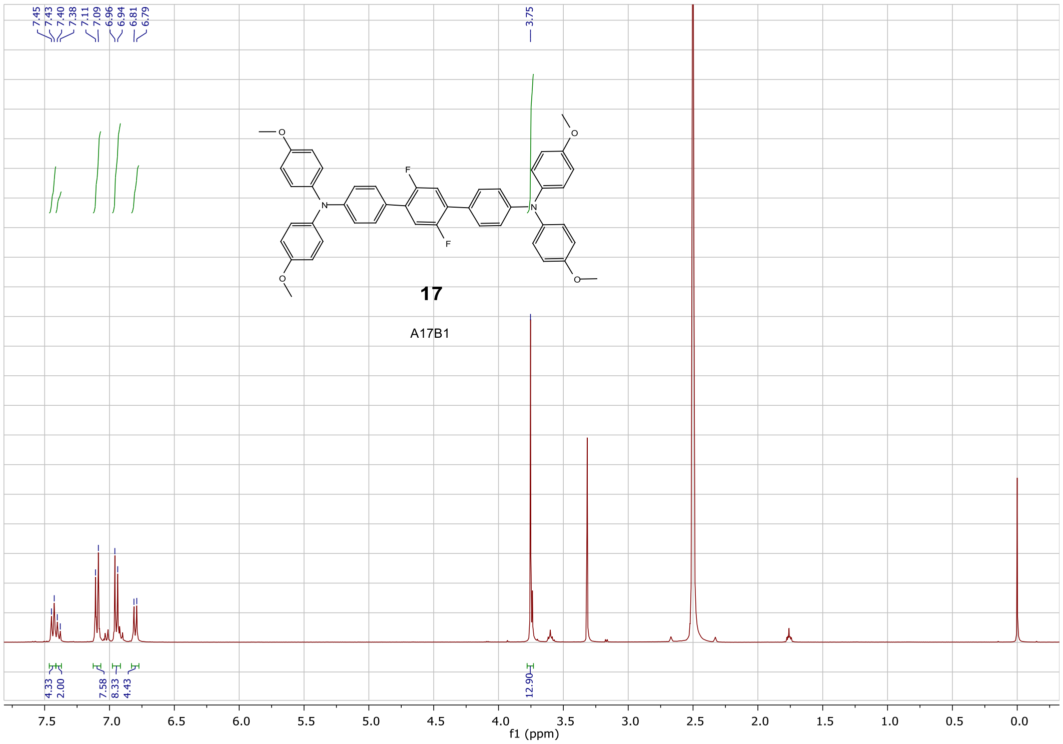

Supplementary Figure 60. ^{1}H NMR of **compound 17 (A17B1)** in d_6-DMSO.

^{1}H NMR (400 MHz, DMSO-d_6) δ 7.44 (d, J = 8.6 Hz, 4H), 7.39 (d, J = 9.3 Hz, 2H), 7.10 (d, J = 8.9 Hz, 8H), 6.95 (d, J = 8.9 Hz, 8H), 6.80 (d, J = 8.8 Hz, 4H), 3.75 (s, 13H).

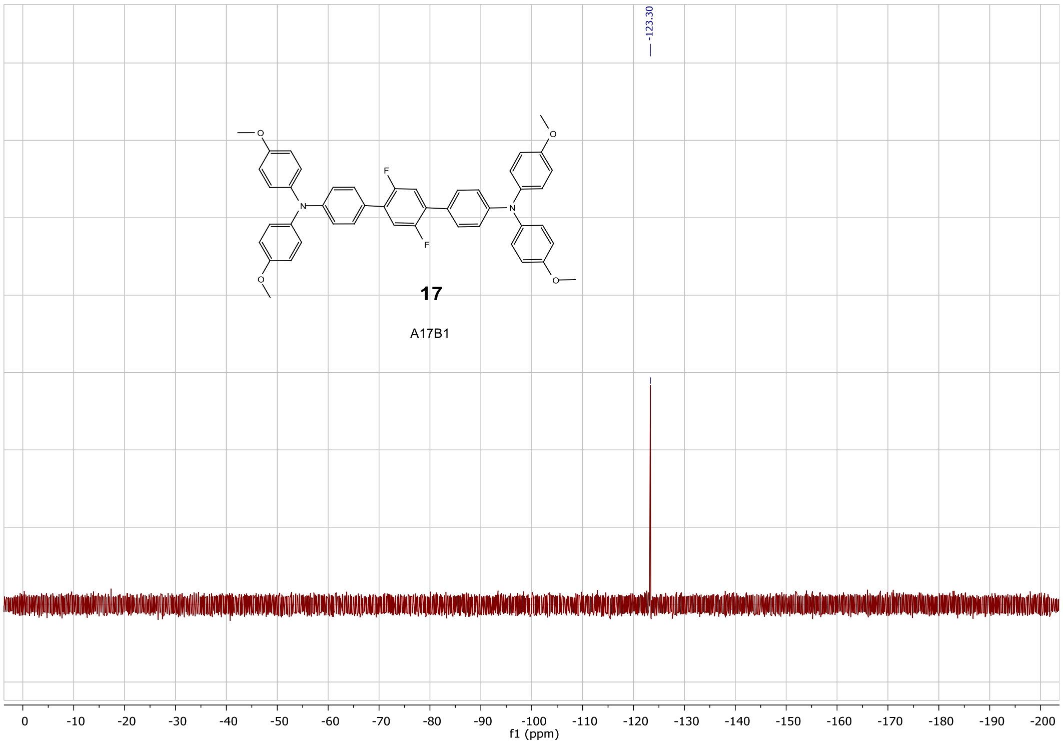

Supplementary Figure 61. ^{19}F NMR of **compound 17 (A17B1)** in *d6*-DMSO.

^{19}F NMR (377 MHz, DMSO-d_6) δ -123.30.

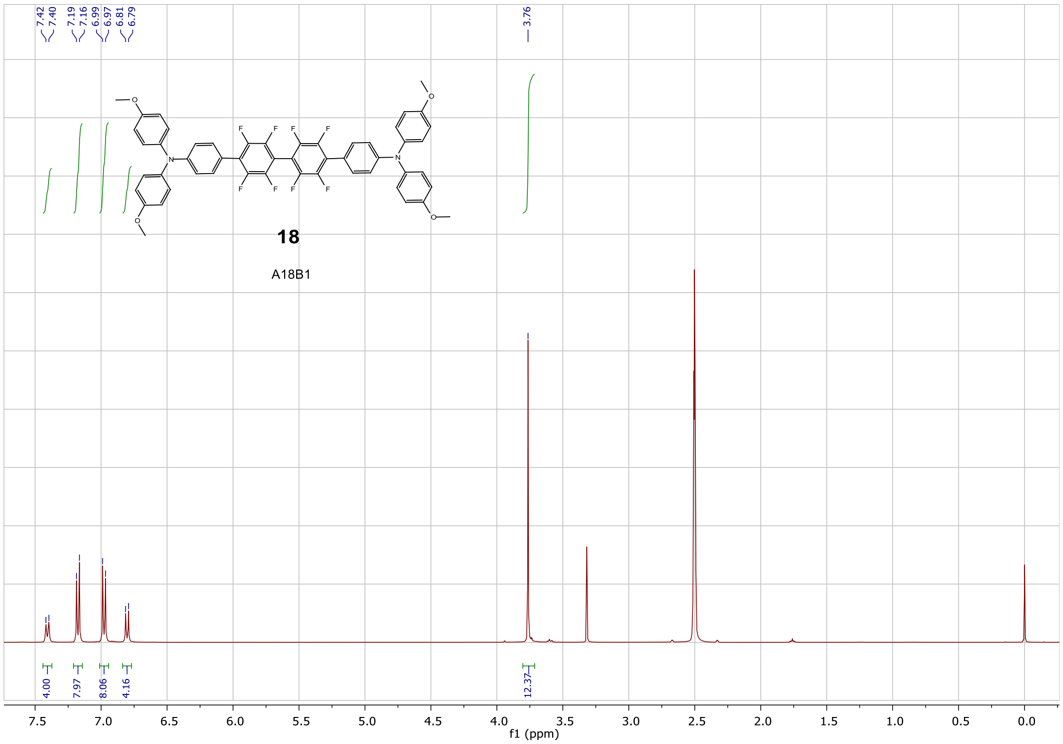

Supplementary Figure 62. ^{1}H NMR of **compound 18 (A18B1)** in *d*$_6$-DMSO.

^{1}H NMR (400 MHz, DMSO-*d*$_6$) δ 7.41 (d, *J* = 9.1 Hz, 4H), 7.18 (d, *J* = 8.9 Hz, 8H), 6.98 (d, *J* = 9.0 Hz, 8H), 6.80 (d, *J* = 8.9 Hz, 4H), 3.76 (s, 12H).

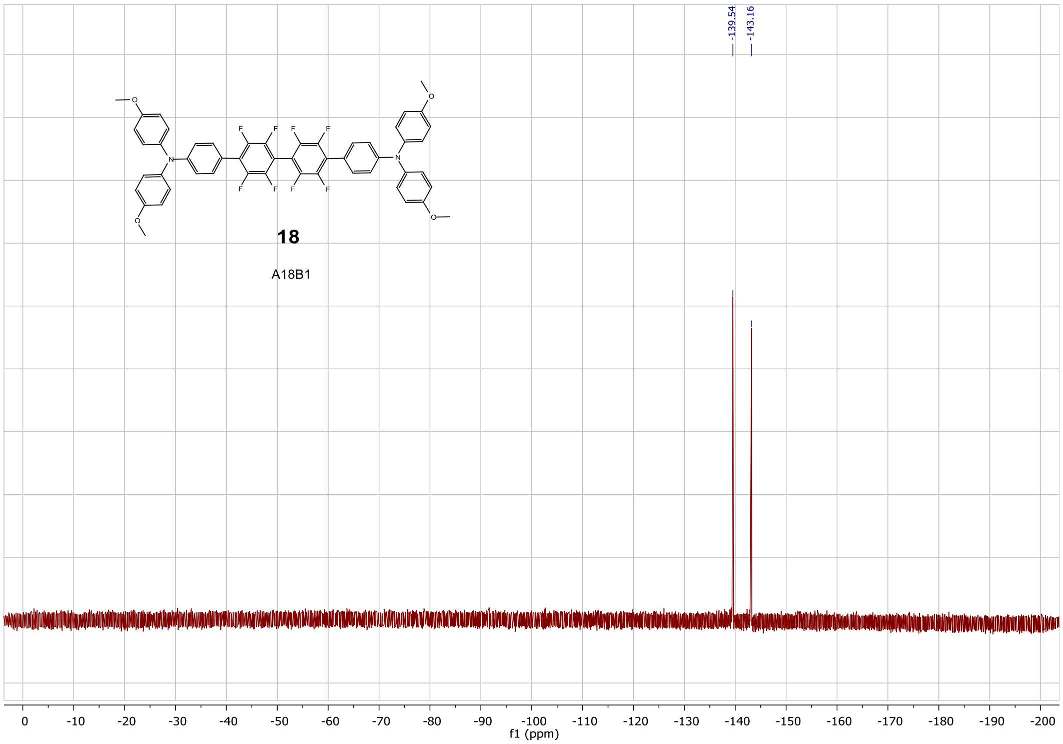

Supplementary Figure 63. ^{19}F NMR of **compound 18 (A18B1)** in d_6-DMSO.

^{19}F NMR (377 MHz, DMSO-d_6) δ -139.54, -143.16.

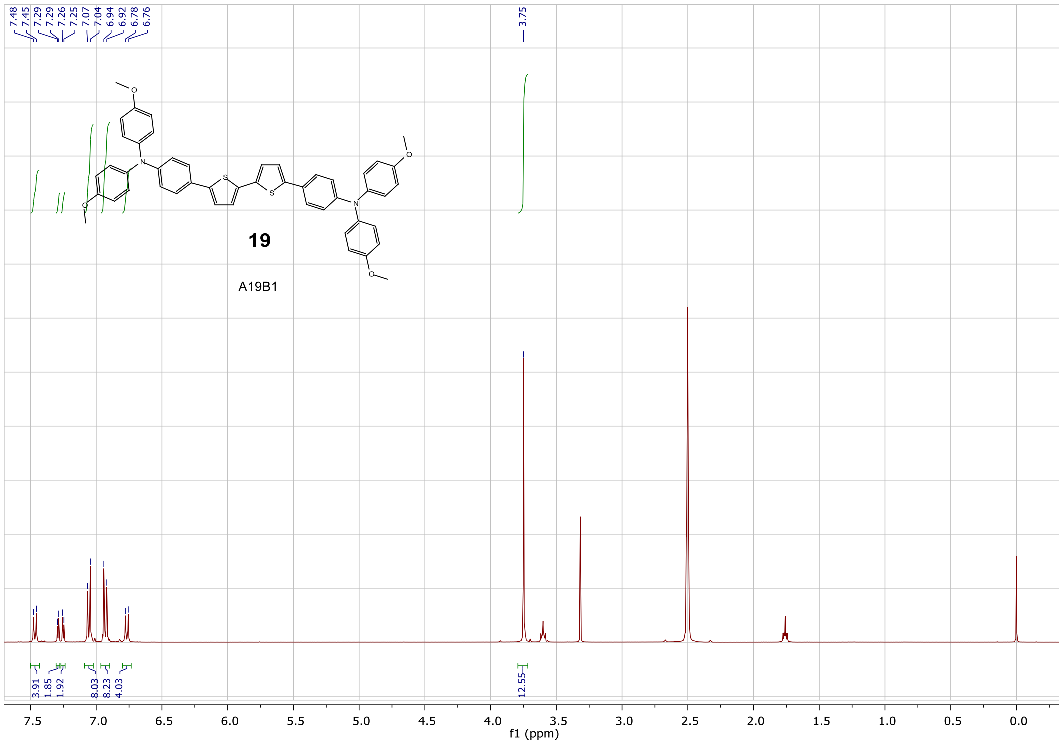

Supplementary Figure 64. ^{1}H NMR of **compound 19 (A19B1)** in d_6-DMSO.

^{1}H NMR (400 MHz, DMSO-d_6) δ 7.47 (d, J = 8.8 Hz, 4H), 7.29 (d, J = 3.8 Hz, 2H), 7.25 (d, J = 3.8 Hz, 2H), 7.06 (d, J = 8.9 Hz, 8H), 6.93 (d, J = 9.0 Hz, 8H), 6.77 (d, J = 8.9 Hz, 4H), 3.75 (s, 13H).

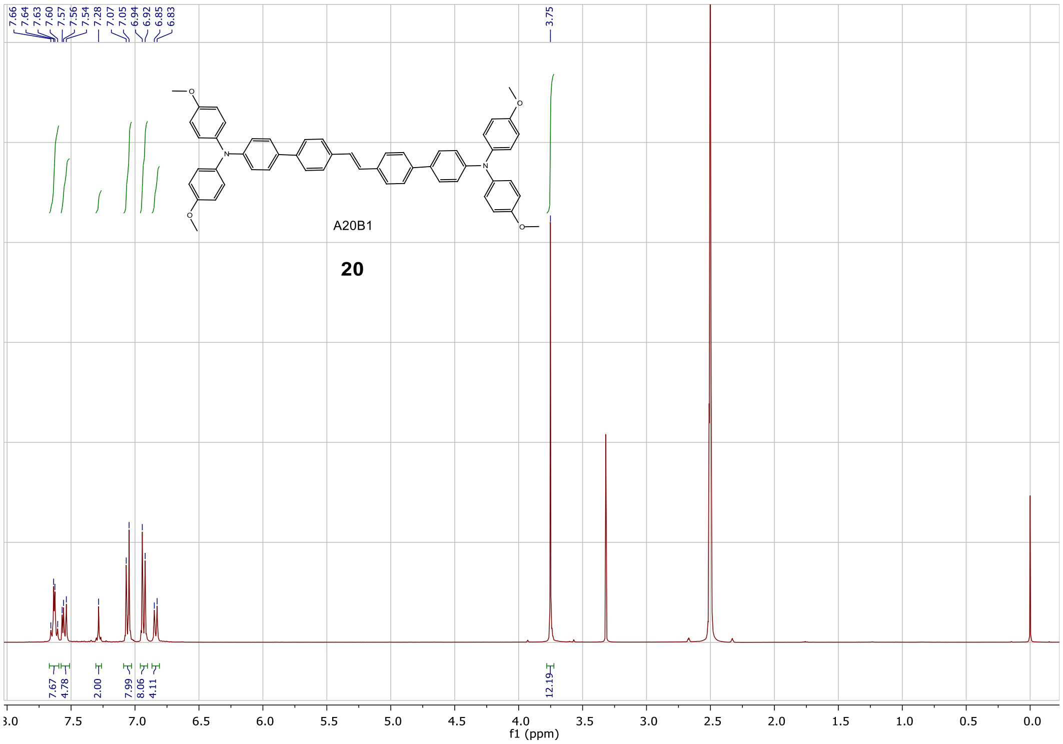

Supplementary Figure 65. ^{1}H NMR of **compound 20 (A20B1)** in d_6-DMSO.

^{1}H NMR (400 MHz, DMSO-d_6) δ 7.63 (d, J = 3.9 Hz, 8H), 7.58 – 7.53 (m, 5H), 7.28 (s, 2H), 7.06 (d, J = 8.9 Hz, 8H), 6.93 (d, J = 9.0 Hz, 8H), 6.84 (d, J = 8.8 Hz, 4H), 3.75 (s, 15H).

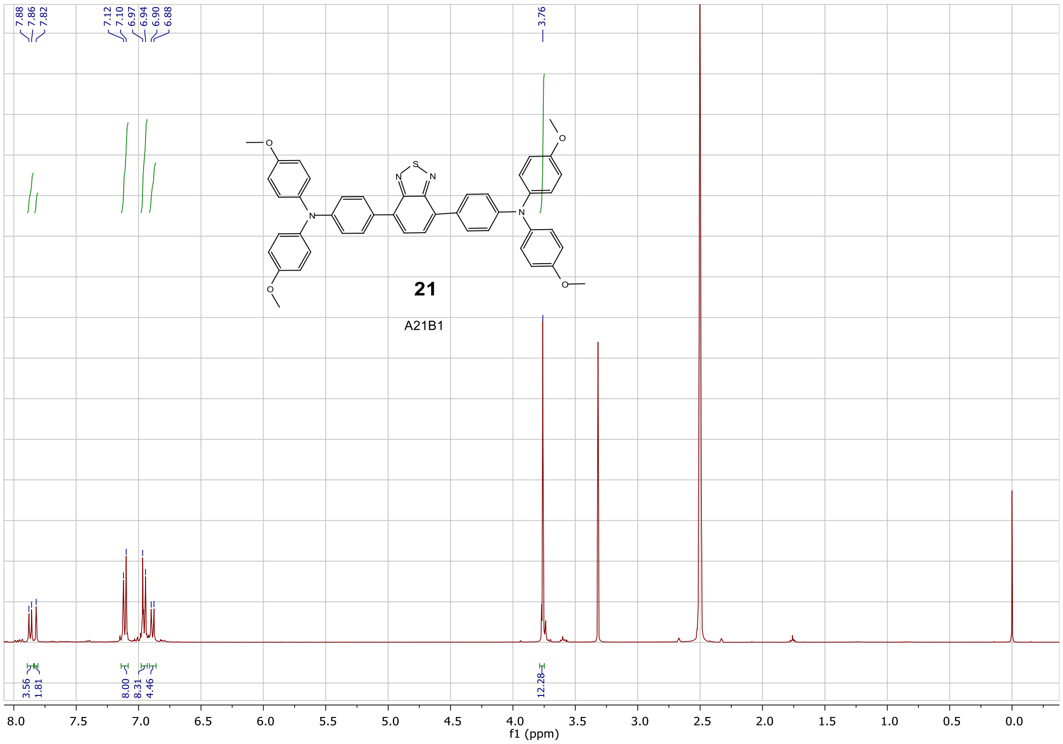

Supplementary Figure 66. ^{1}H NMR of **compound 21 (A21B1)** in d_6-DMSO.

^{1}H NMR (400 MHz, DMSO-d_6) δ 7.87 (d, J = 8.8 Hz, 4H), 7.82 (s, 2H), 7.11 (d, J = 8.9 Hz, 8H), 6.96 (d, J = 9.0 Hz, 8H), 6.89 (d, J = 8.8 Hz, 4H), 3.76 (s, 12H).

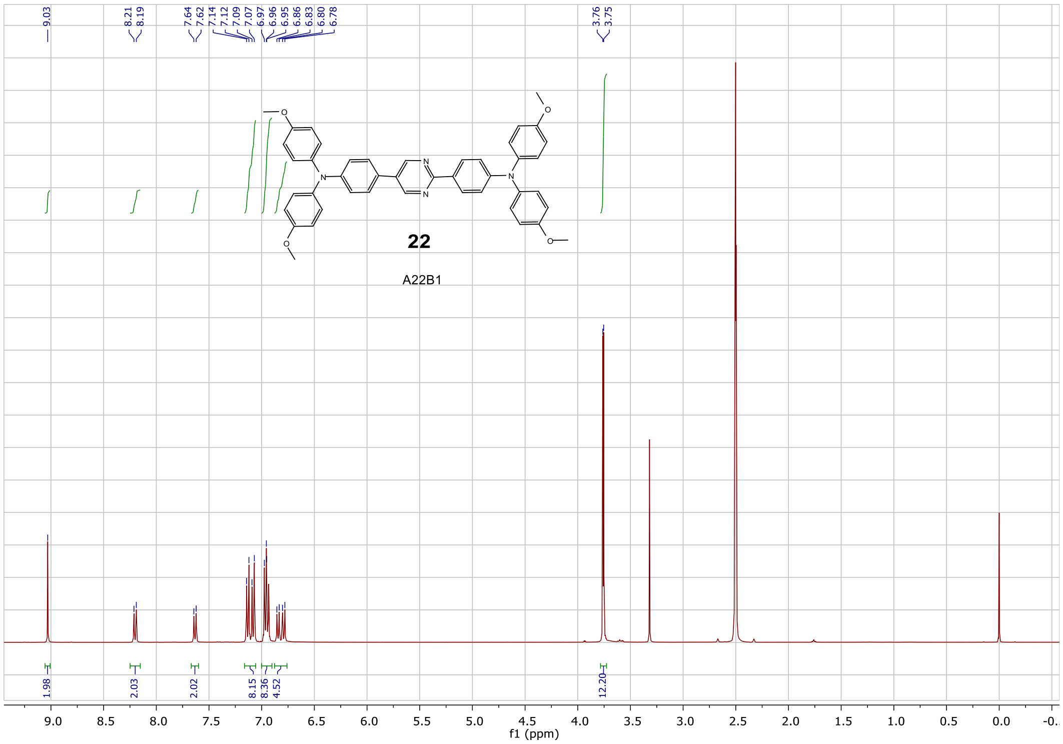

Supplementary Figure 67. ^{1}H NMR of **compound 22 (A22B1)** in *d6*-DMSO.

^{1}H NMR (400 MHz, DMSO-d_6) δ 9.03 (s, 2H), 8.20 (d, *J* = 9.0 Hz, 2H), 7.63 (d, *J* = 8.8 Hz, 2H), 7.11 (dd, *J* = 20.5, 8.9 Hz, 8H), 7.00 – 6.92 (m, 8H), 6.82 (dd, *J* = 21.4, 8.9 Hz, 4H), 3.76 (d, *J* = 3.1 Hz, 13H).

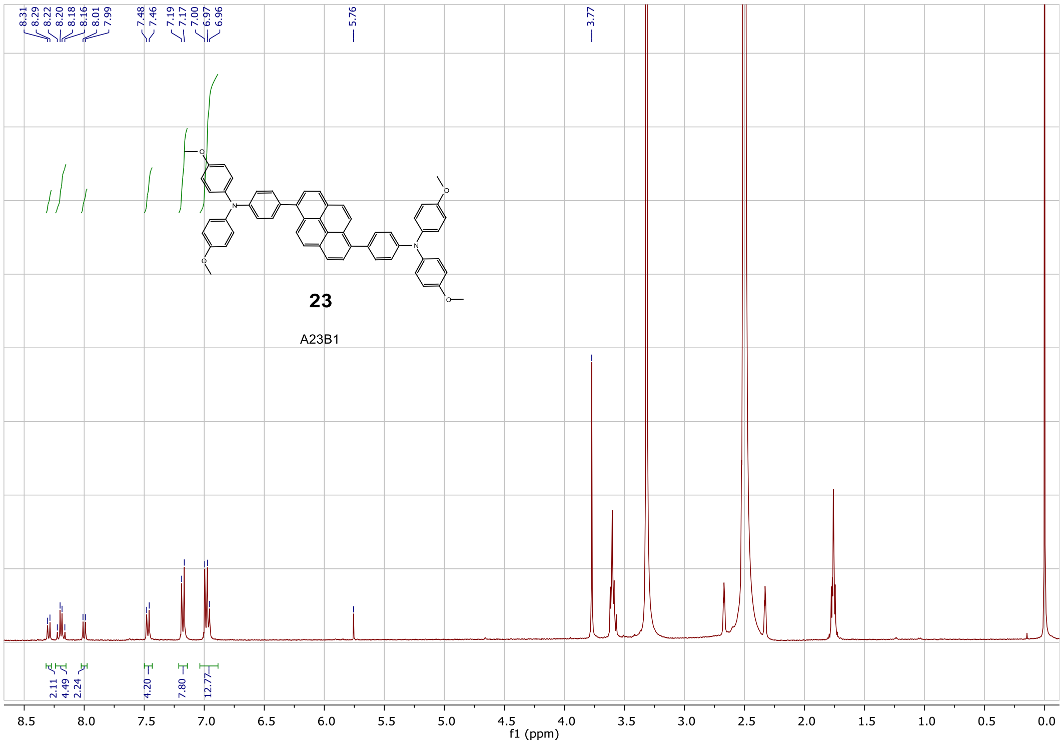

Supplementary Figure 68. ^{1}H NMR of **compound 23 (A23B1)** in d_6-DMSO.

^{1}H NMR (400 MHz, DMSO-d_6) δ 8.30 (d, J = 8.0 Hz, 2H), 8.24 – 8.15 (m, 4H), 8.00 (d, J = 7.9 Hz, 2H), 7.47 (d, J = 8.6 Hz, 4H), 7.18 (d, J = 8.9 Hz, 8H), 7.04 – 6.89 (m, 13H).

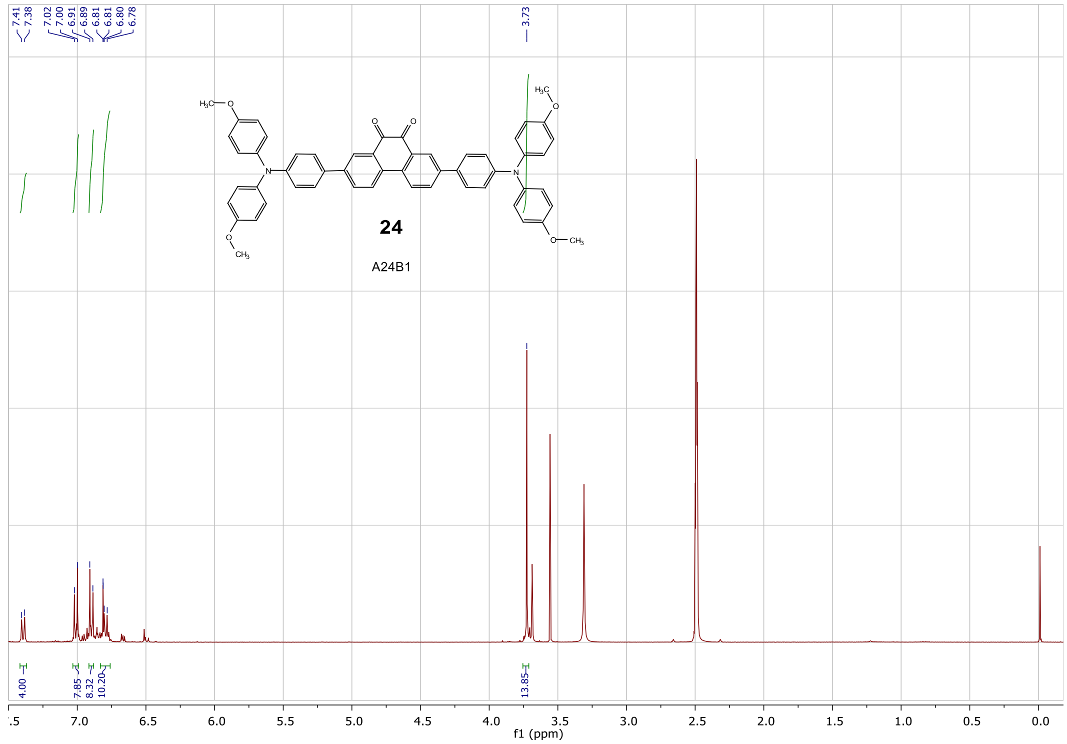

Supplementary Figure 69. ^{1}H NMR of **compound 24 (A24B1)** in d_6-DMSO.

^{1}H NMR (400 MHz, DMSO-d_6) δ 7.39 (d, J = 8.8 Hz, 4H), 7.01 (d, J = 9.0 Hz, 8H), 6.90 (d, J = 9.0 Hz, 8H), 6.83 – 6.76 (m, 10H), 3.73 (s, 14H).

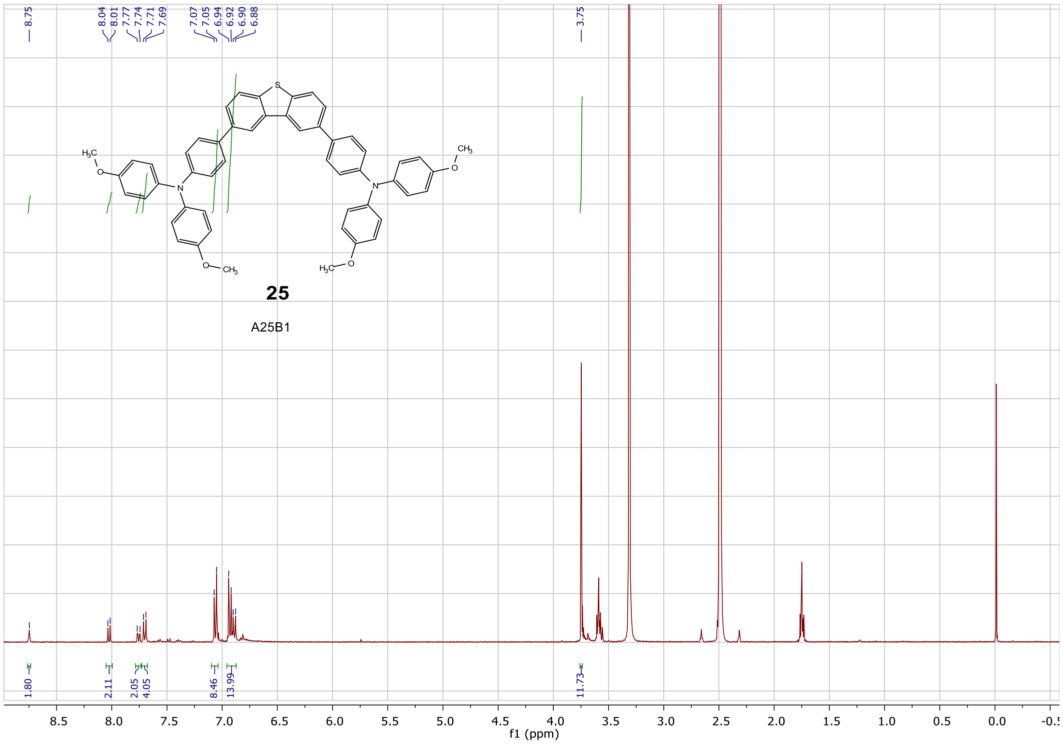

Supplementary Figure 70. ^{1}H NMR of **compound 25 (A25B1)** in d_6-DMSO.

^{1}H NMR (400 MHz, DMSO-d_6) δ 8.75 (s, 2H), 8.02 (d, *J* = 8.4 Hz, 2H), 7.76 (d, *J* = 10.2 Hz, 2H), 7.70 (d, *J* = 8.8 Hz, 4H), 7.06 (d, *J* = 9.0 Hz, 8H), 6.96 – 6.87 (m, 14H), 3.75 (s, 12H).

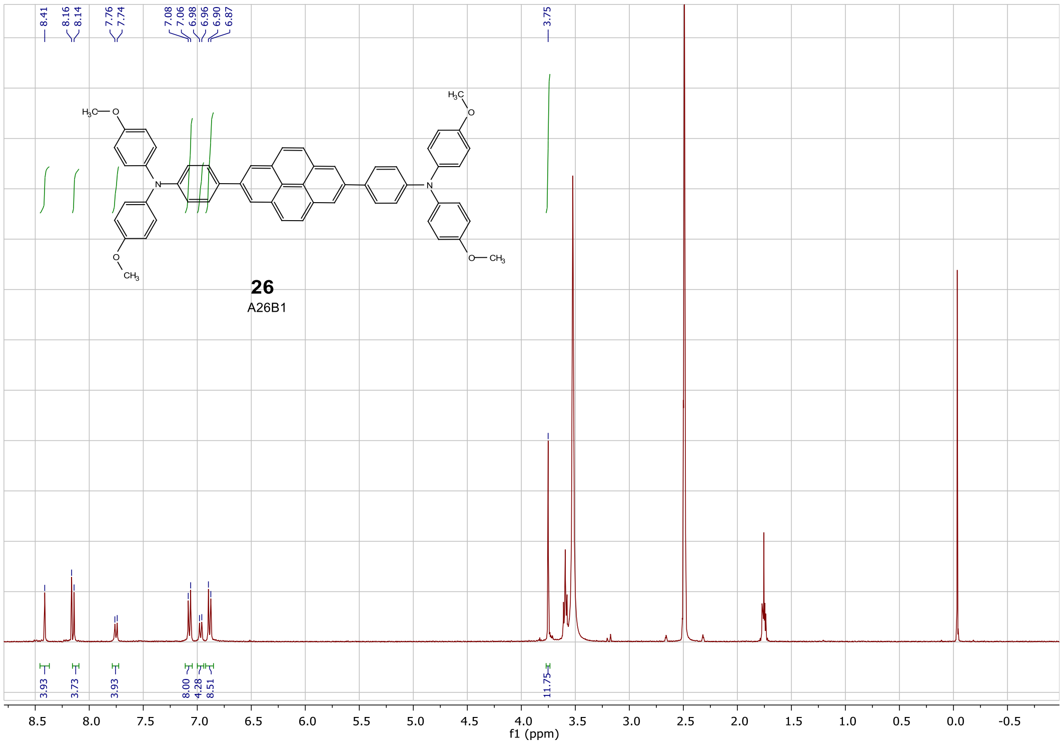

Supplementary Figure 71. ^{1}H NMR of **compound 26 (A26B1)** in d_6-DMSO.

^{1}H NMR (400 MHz, DMSO-d_6) δ 8.41 (s, 4H), 8.14 (s, 4H), 7.75 (d, J = 8.7 Hz, 4H), 7.07 (d, J = 9.0 Hz, 8H), 6.97 (d, J = 8.7 Hz, 4H), 6.89 (d, J = 9.0 Hz, 9H), 3.75 (s, 12H).

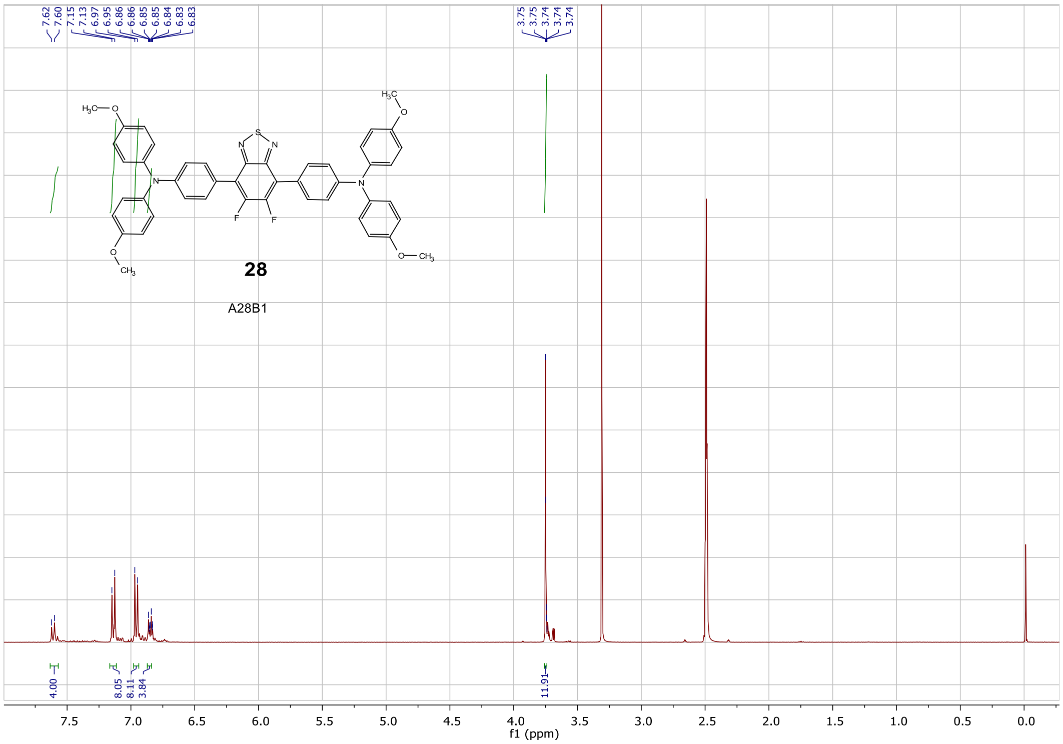

Supplementary Figure 72. ^{1}H NMR of **compound 28 (A28B1)** in d_6-DMSO.

^{1}H NMR (400 MHz, DMSO-d_6) δ 7.61 (d, J = 8.9 Hz, 4H), 7.14 (d, J = 9.0 Hz, 8H), 6.96 (d, J = 9.0 Hz, 8H), 6.85 (d, J = 8.9 Hz, 4H), 3.75 (d, J = 0.7 Hz, 12H).

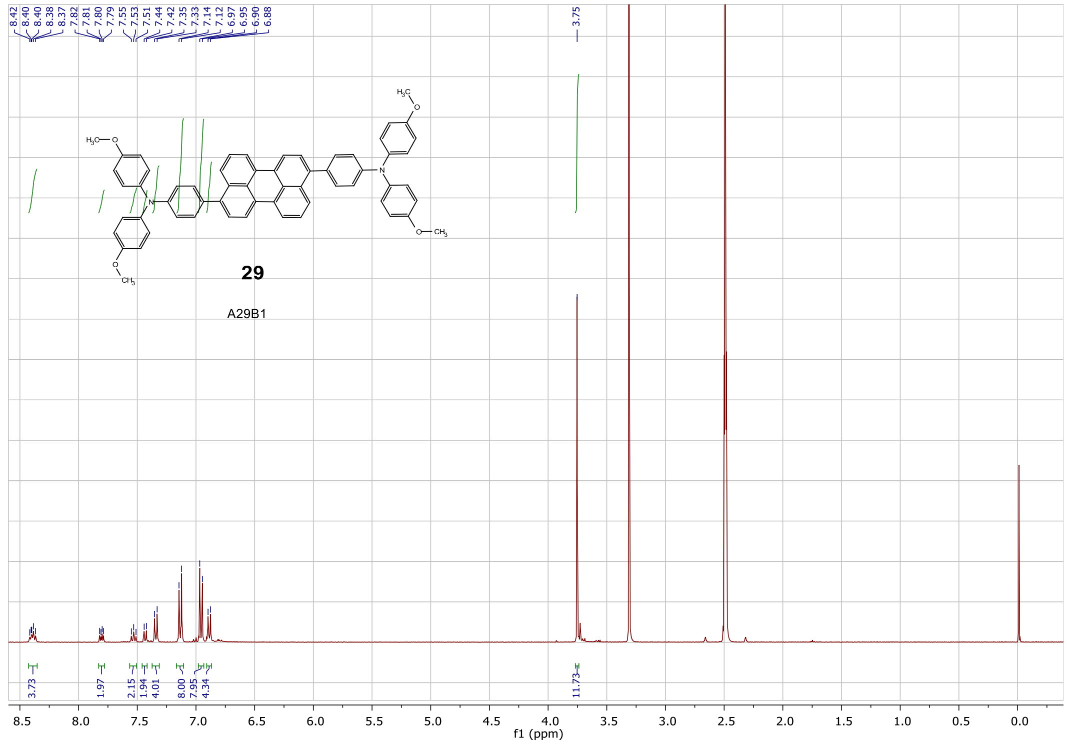

Supplementary Figure 73. ^{1}H NMR of **compound 29 (A29B1)** in *d*$_6$-DMSO.

^{1}H NMR (400 MHz, DMSO-*d*$_6$) δ 8.43 – 8.35 (m, 4H), 7.83 – 7.78 (m, 2H), 7.53 (t, *J* = 8.0 Hz, 2H), 7.43 (d, *J* = 7.7 Hz, 2H), 7.34 (d, *J* = 8.7 Hz, 4H), 7.13 (d, *J* = 8.9 Hz, 8H), 6.96 (d, *J* = 9.0 Hz, 8H), 6.89 (d, *J* = 8.7 Hz, 4H), 3.75 (s, 12H).

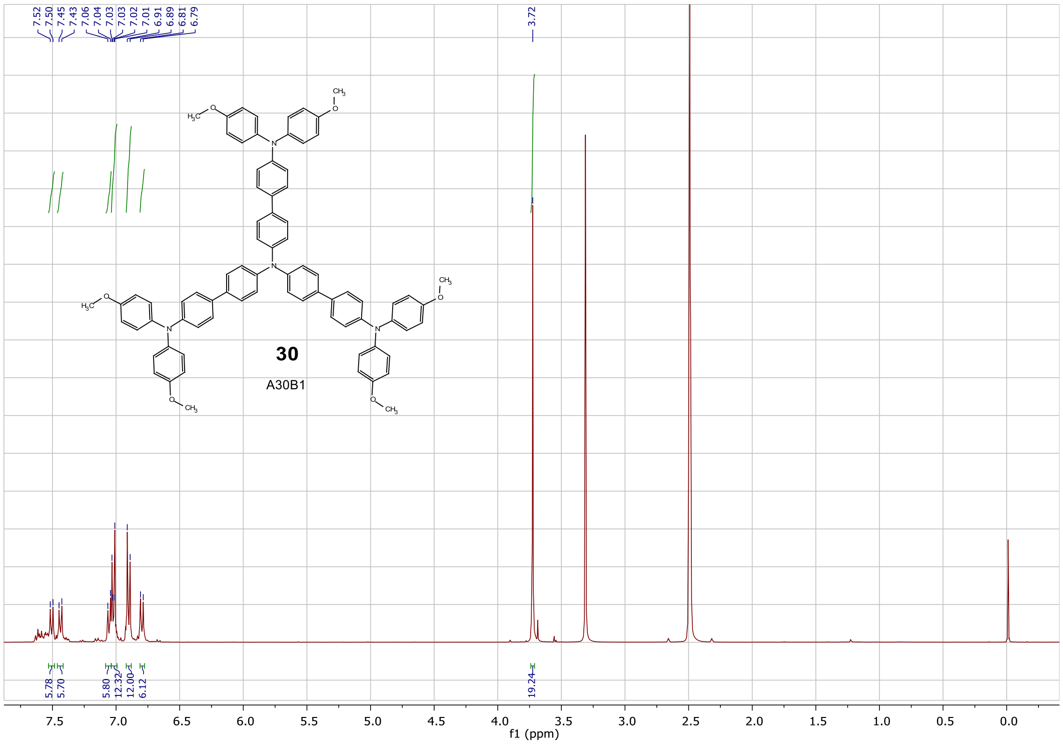

Supplementary Figure 74. ^{1}H NMR of **compound 30 (A30B1)** in d_6-DMSO.

^{1}H NMR (400 MHz, DMSO-d_6) δ 7.51 (d, J = 8.6 Hz, 6H), 7.44 (d, J = 8.8 Hz, 6H), 7.05 (d, J = 8.6 Hz, 6H), 7.04 – 6.99 (m, 12H), 6.90 (d, J = 9.0 Hz, 12H), 6.80 (d, J = 8.7 Hz, 6H), 3.72 (s, 19H).

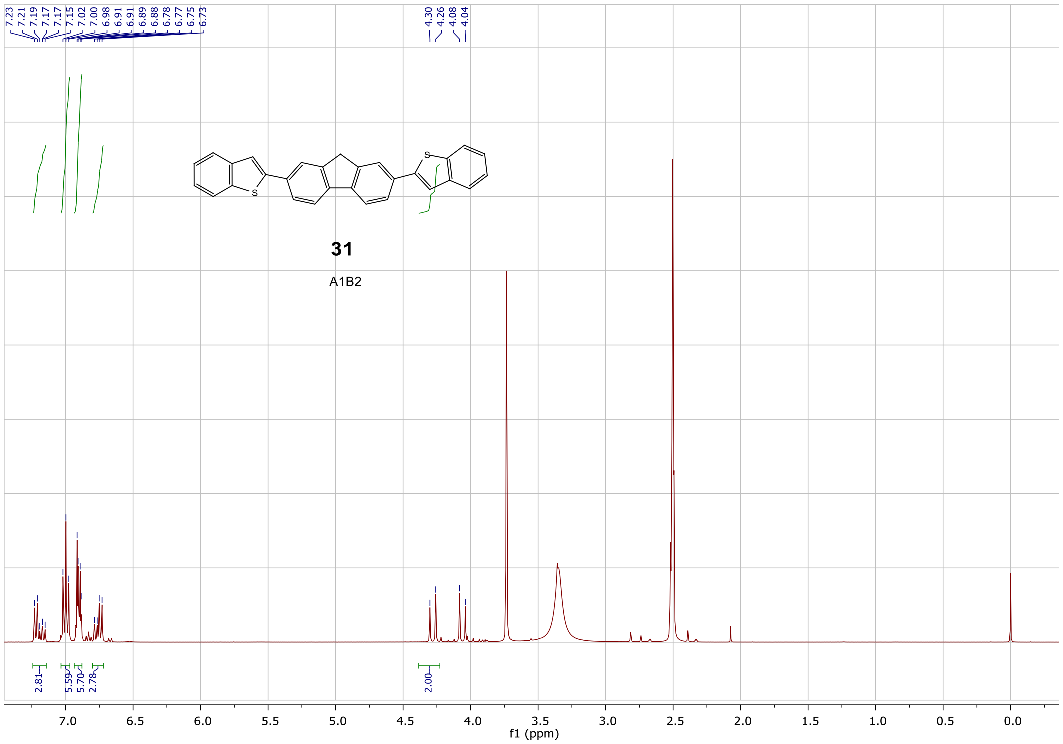

Supplementary Figure 75. ^{1}H NMR of **compound 31 (A1B2)** in d_6-DMSO.

^{1}H NMR (400 MHz, DMSO-d_6) δ 7.24 – 7.15 (m, 3H), 7.00 (t, J = 8.7 Hz, 6H), 6.94 – 6.86 (m, 6H), 6.81 – 6.71 (m, 3H), 4.28 (d, J = 17.1 Hz, 2H).

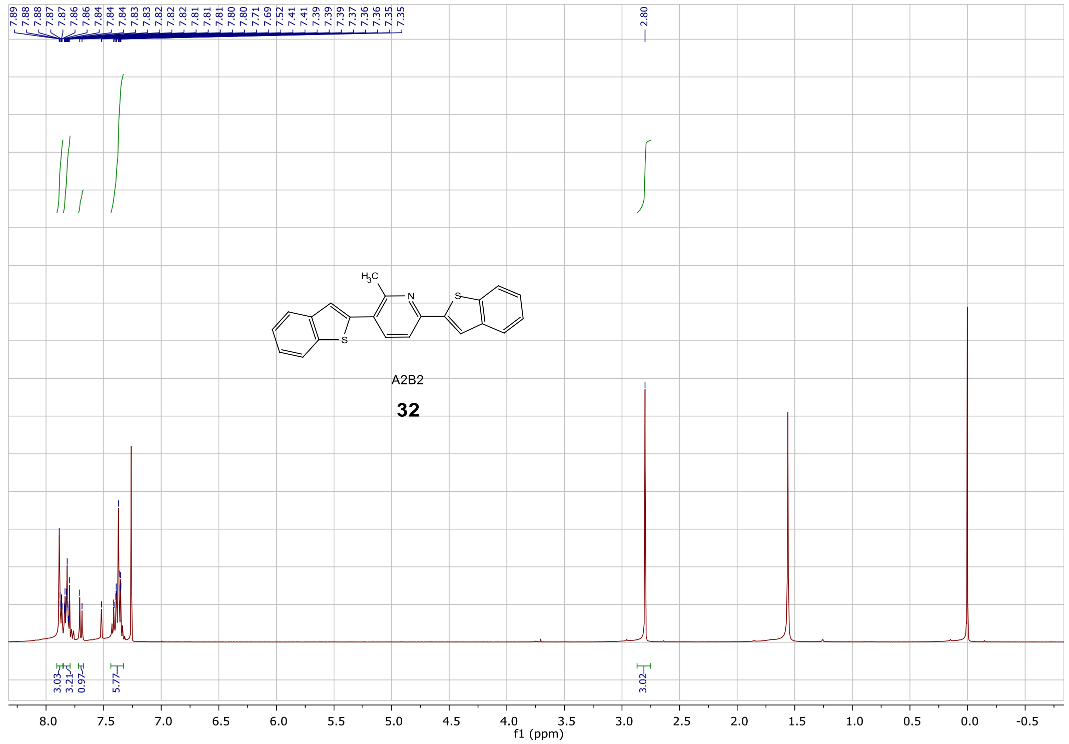

Supplementary Figure 76. ^{1}H NMR of **compound 32 (A2B2)** in $CDCl_3$.

^{1}H NMR (400 MHz, Chloroform-*d*) δ 7.91 – 7.85 (m, 3H), 7.85 – 7.79 (m, 3H), 7.70 (d, *J* = 8.0 Hz, 1H), 7.44 – 7.33 (m, 6H), 2.80 (s, 3H).

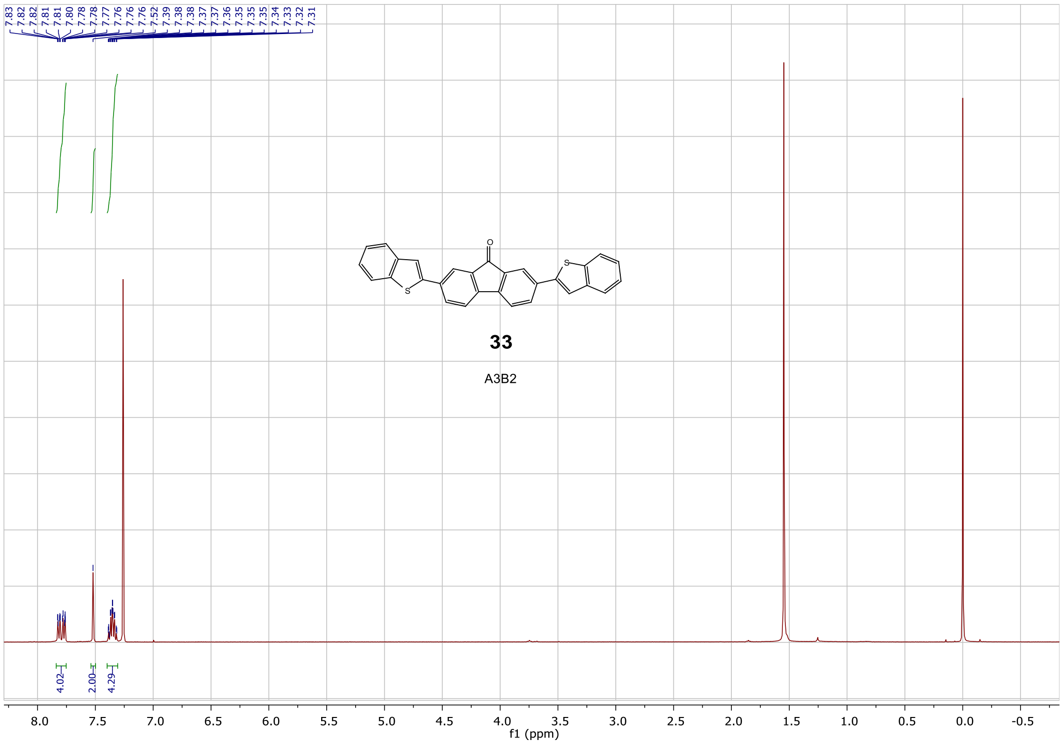

Supplementary Figure 77. ^{1}H NMR of **compound 33 (A3B2)** in $CDCl_3$.

^{1}H NMR (400 MHz, Chloroform-d) δ 7.84 – 7.75 (m, 4H), 7.52 (s, 2H), 7.40 – 7.31 (m, 4H).

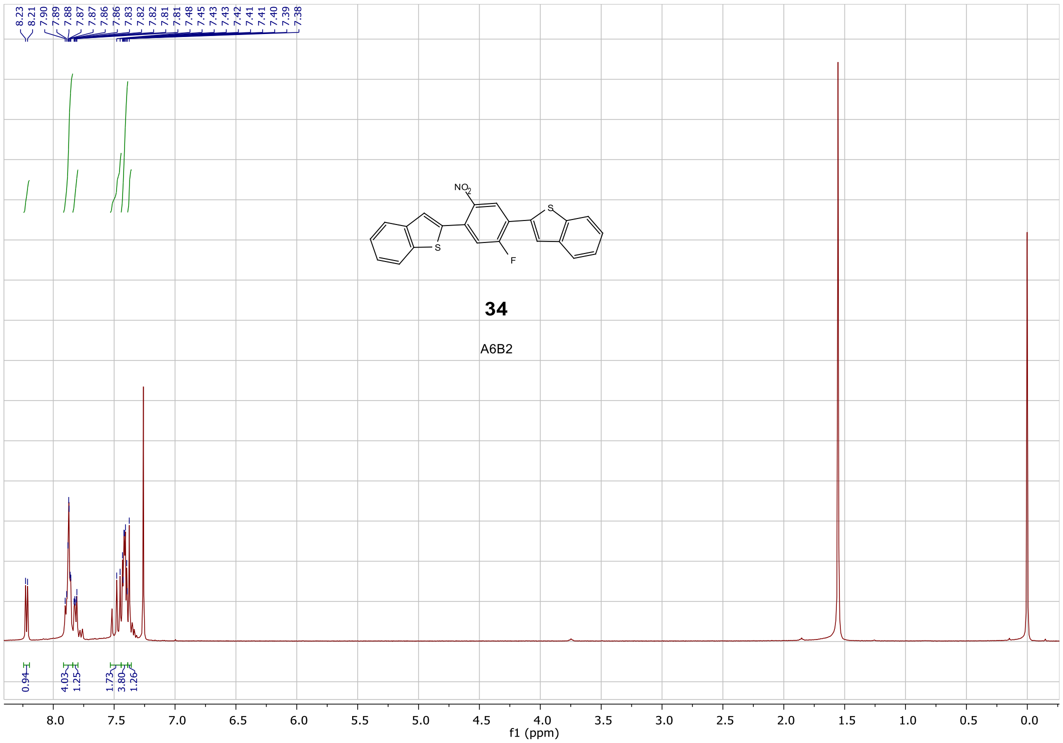

Supplementary Figure 78. ^{1}H NMR of **compound 34 (A6B2)** in $CDCl_3$.

^{1}H NMR (400 MHz, Chloroform-*d*) δ 8.22 (d, *J* = 6.8 Hz, 1H), 7.92 – 7.84 (m, 4H), 7.84 – 7.80 (m, 1H), 7.53 – 7.44 (m, 2H), 7.44 – 7.39 (m, 4H), 7.38 (s, 1H).

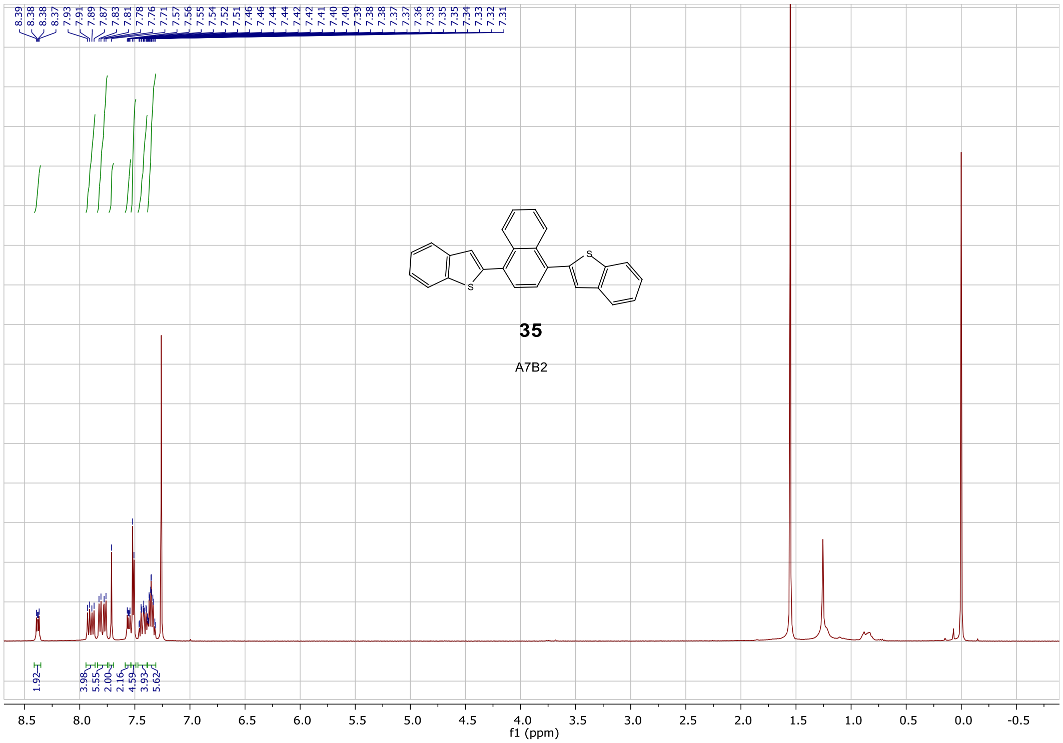

Supplementary Figure 79. ^{1}H NMR of **compound 35 (A7B2)** in $CDCl_3$.

^{1}H NMR (400 MHz, Chloroform-*d*) δ 8.41 – 8.35 (m, 2H), 7.94 – 7.86 (m, 4H), 7.84 – 7.75 (m, 6H), 7.71 (s, 2H), 7.59 – 7.54 (m, 2H), 7.51 (d, *J* = 4.8 Hz, 5H), 7.47 – 7.39 (m, 4H), 7.38 – 7.31 (m, 6H).

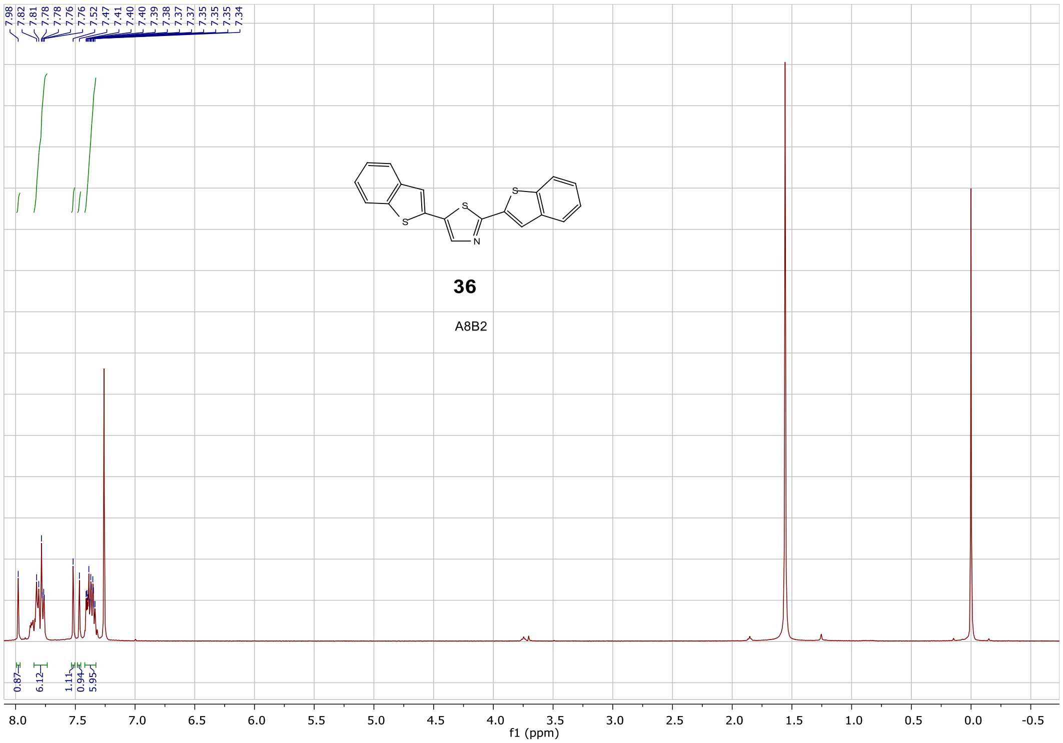

Supplementary Figure 80. ^{1}H NMR of **compound 36 (A8B2)** in $CDCl_3$.

^{1}H NMR (400 MHz, Chloroform-*d*) δ 7.98 (s, 1H), 7.85 – 7.74 (m, 6H), 7.52 (s, 1H), 7.47 (s, 1H), 7.42 – 7.33 (m, 5H).

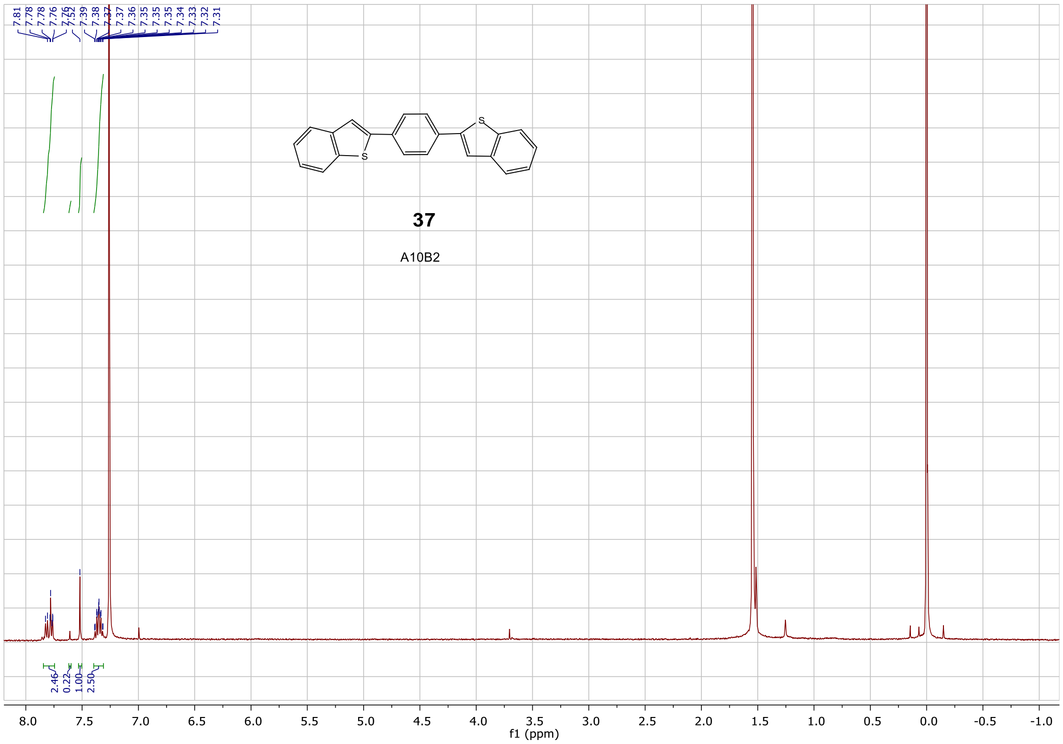

Supplementary Figure 81. ^{1}H NMR of **compound 37 (A10B2)** in $CDCl_3$.

^{1}H NMR (400 MHz, Chloroform-*d*) δ 7.84 – 7.74 (m, 2H), 7.52 (s, 1H), 7.40 – 7.31 (m, 2H).

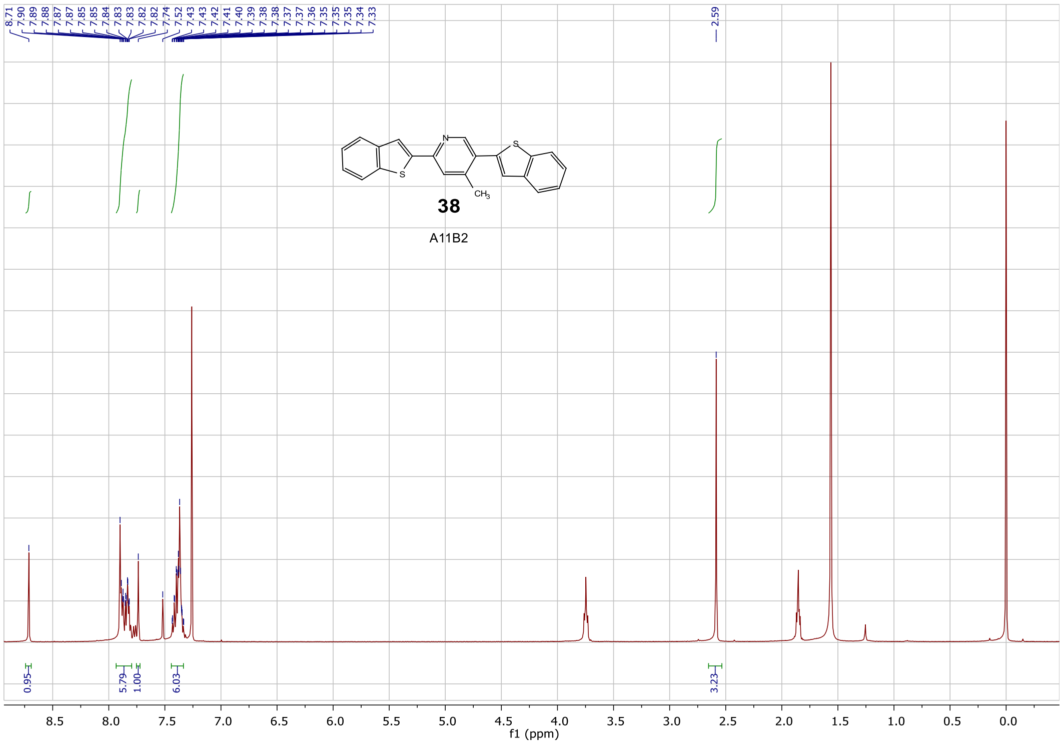

Supplementary Figure 82. ^{1}H NMR of **compound 38 (A11B2)** in $CDCl_3$.

^{1}H NMR (400 MHz, Chloroform-*d*) δ 8.71 (s, 1H), 7.93 – 7.80 (m, 6H), 7.74 (s, 1H), 7.44 – 7.33 (m, 6H), 2.59 (s, 3H).

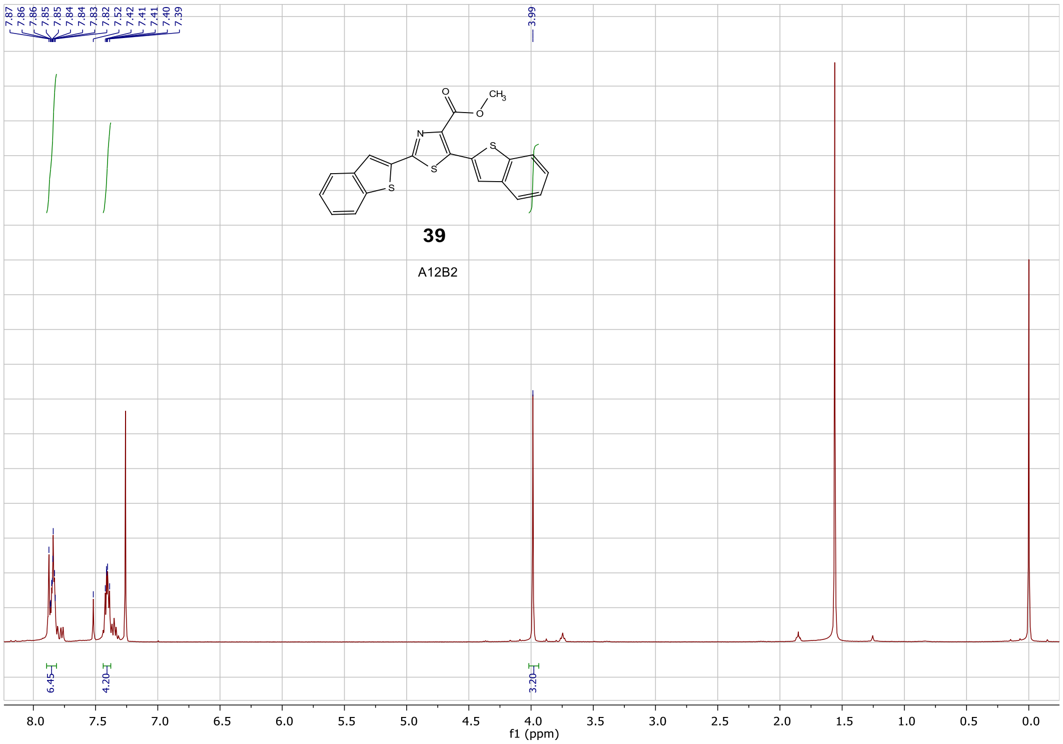

Supplementary Figure 83. ^{1}H NMR of **compound 39 (A12B2)** in $CDCl_3$.

^{1}H NMR (400 MHz, Chloroform-*d*) δ 7.89 – 7.81 (m, 6H), 7.44 – 7.38 (m, 4H), 3.99 (s, 3H).

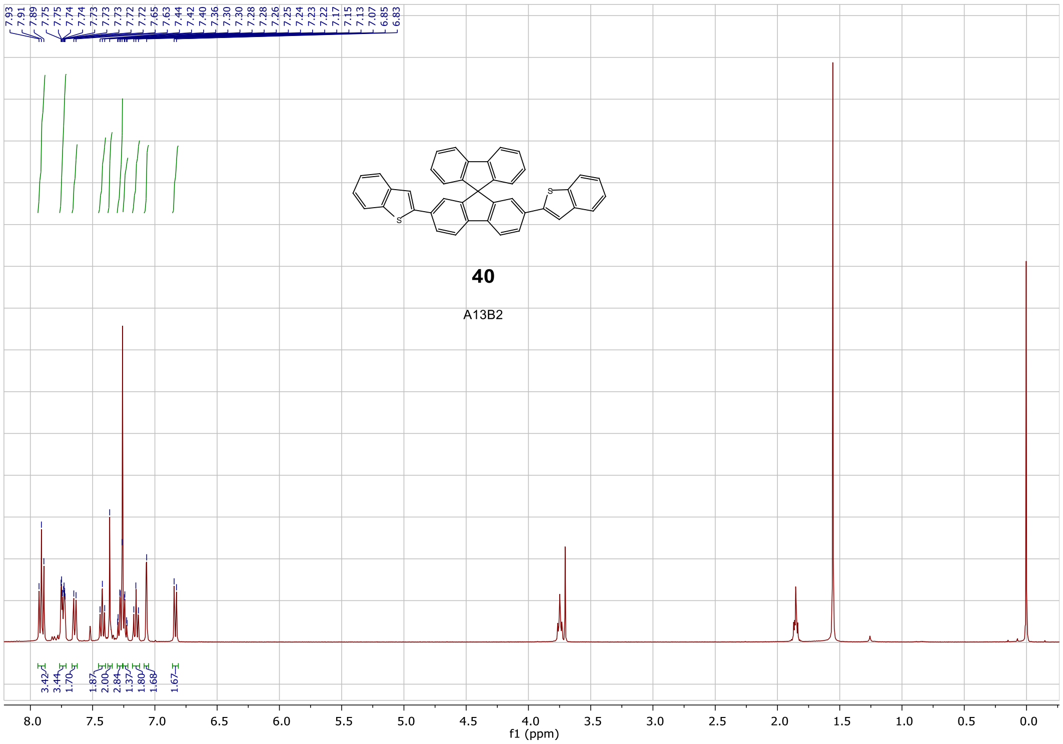

Supplementary Figure 84. ^{1}H NMR of **compound 40 (A13B2)** in $CDCl_3$.

^{1}H NMR (400 MHz, Chloroform-*d*) δ 7.91 (t, *J* = 7.8 Hz, 3H), 7.76 – 7.71 (m, 3H), 7.64 (d, *J* = 7.2 Hz, 2H), 7.42 (t, *J* = 7.5 Hz, 2H), 7.36 (s, 2H), 7.30 – 7.26 (m, 3H), 7.26 – 7.22 (m, 1H), 7.18 – 7.12 (m, 2H), 7.07 (s, 2H), 6.84 (d, *J* = 7.6 Hz, 2H).

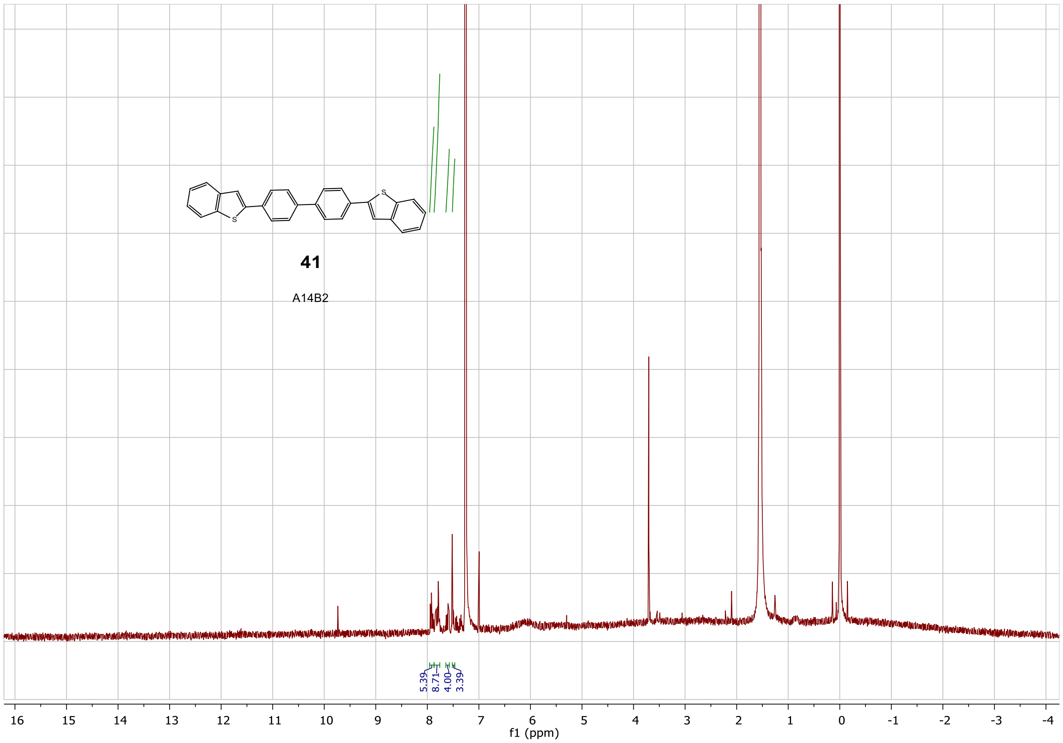

Supplementary Figure 85. ^{1}H NMR of **compound 41 (A14B2)** in $CDCl_3$.

^{1}H NMR (400 MHz, Chloroform-*d*) δ 7.96 – 7.87 (m, 5H), 7.87 – 7.76 (m, 9H), 7.61 (m, 4H), 7.51 – 7.47 (m, 3H).

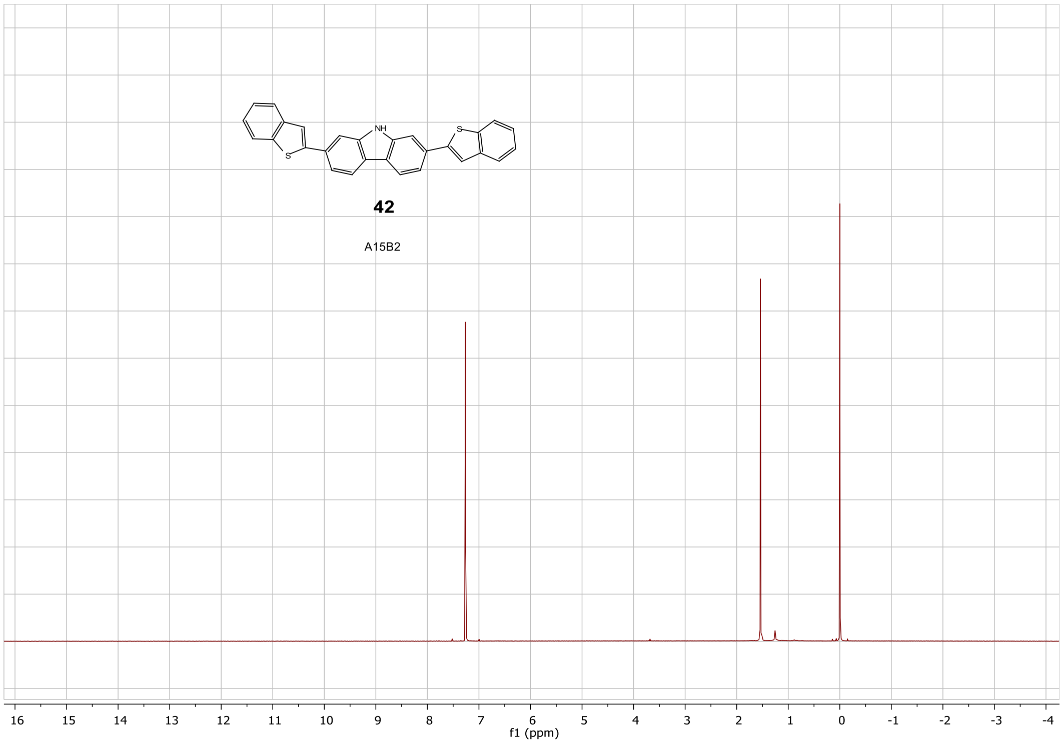

Supplementary Figure 86. ^{1}H NMR of **compound 42 (A15B2)** in $CDCl_3$.

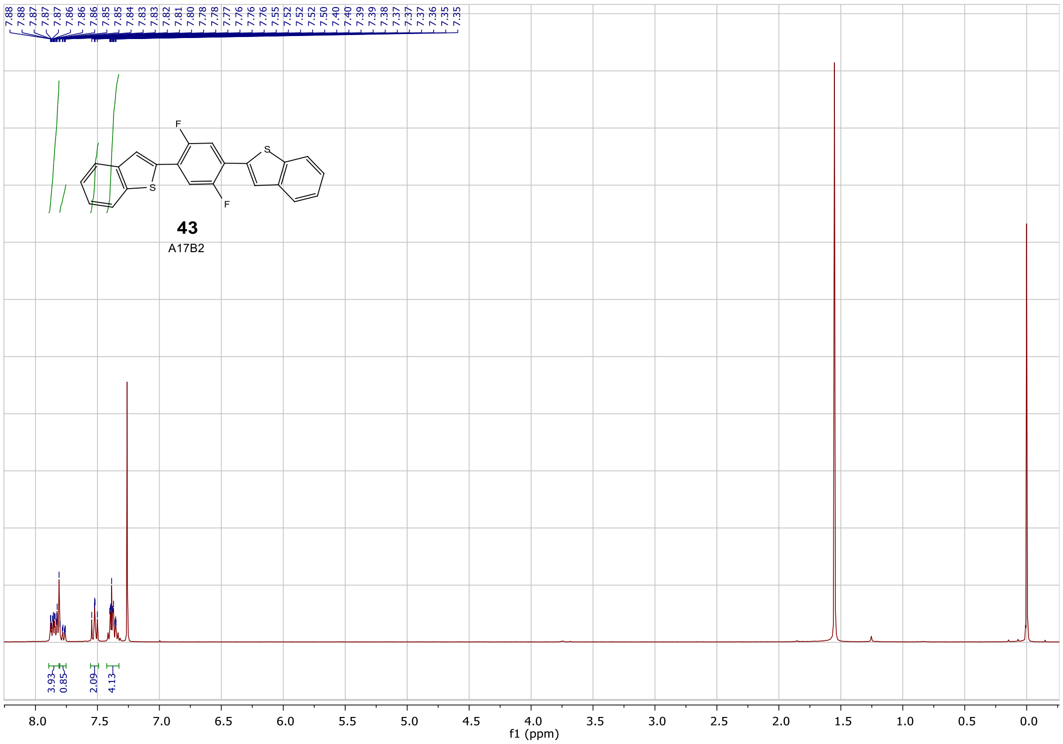

Supplementary Figure 87. ^{1}H NMR of **compound 43 (A17B2)** in $CDCl_3$.

^{1}H NMR (400 MHz, Chloroform-*d*) δ 7.89 – 7.81 (m, 4H), 7.80 – 7.75 (m, 2H), 7.56 – 7.49 (m, 2H), 7.42 – 7.33 (m, 4H).

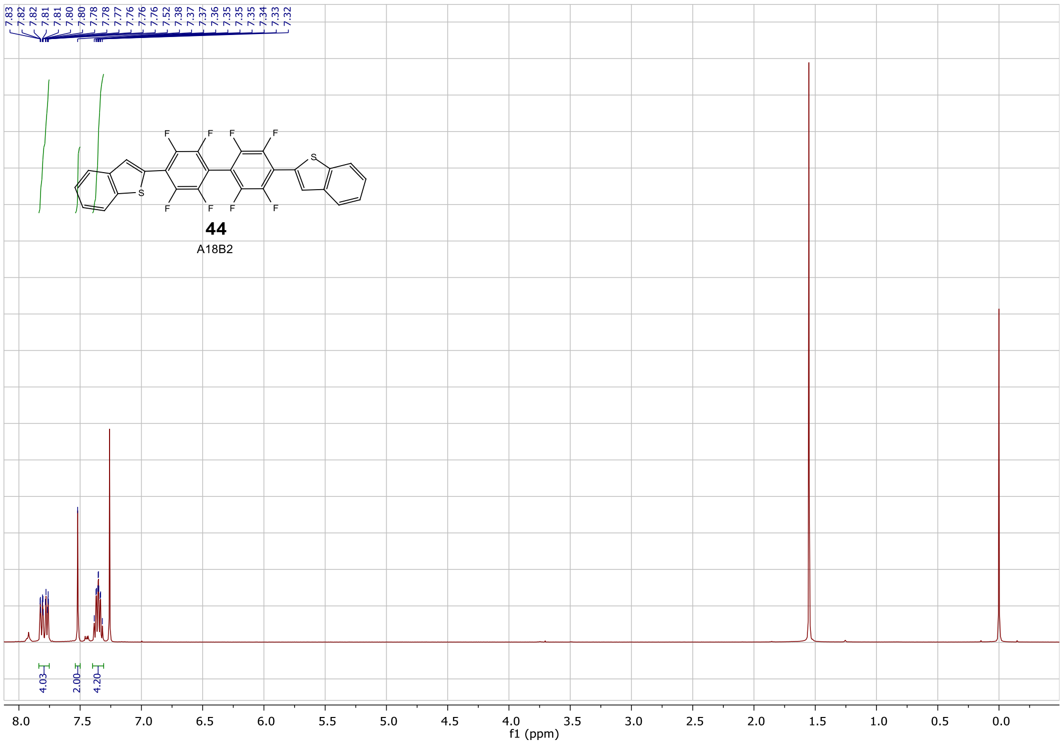

Supplementary Figure 88. ^{1}H NMR of **compound 44 (A18B2)** in $CDCl_3$.

^{1}H NMR (400 MHz, Chloroform-*d*) δ 7.84 – 7.75 (m, 4H), 7.52 (s, 2H), 7.40 – 7.31 (m, 4H).

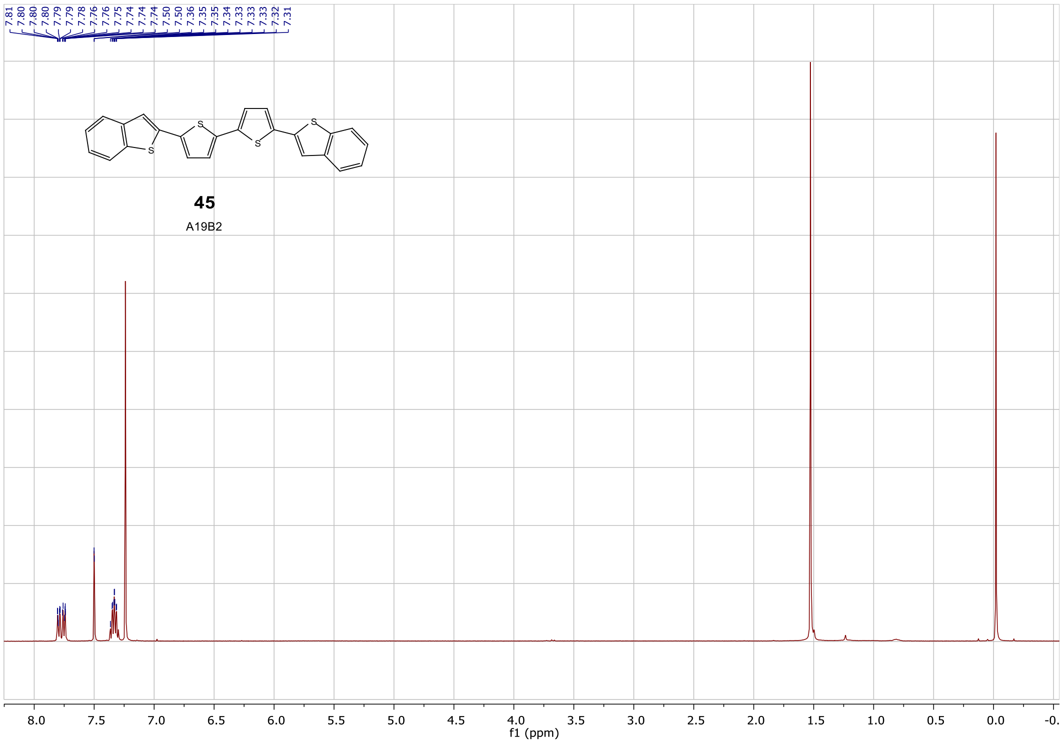

Supplementary Figure 89. ^{1}H NMR of **compound 45 (A19B2)** in $CDCl_3$.

^{1}H NMR (400 MHz, Chloroform-*d*) δ 7.83 – 7.72 (m, 2H), 7.50 (d, *J* = 0.8 Hz, 1H), 7.37 – 7.29 (m, 2H).

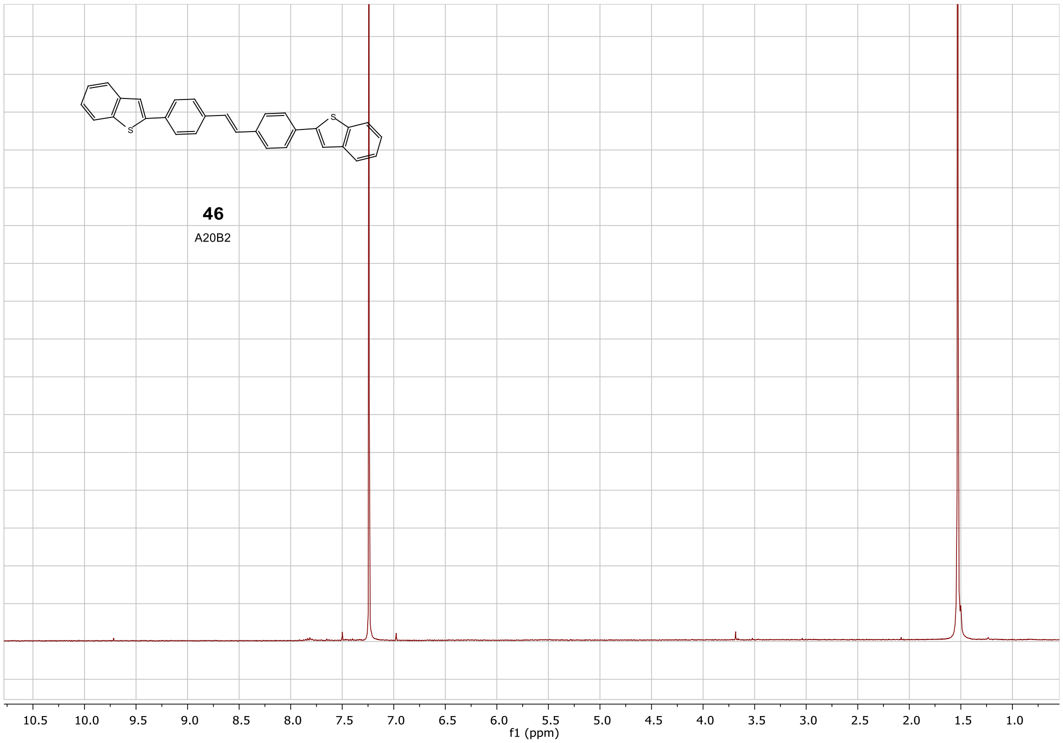

Supplementary Figure 90. ^{1}H NMR of **compound 46 (A20B2)** in $CDCl_3$.

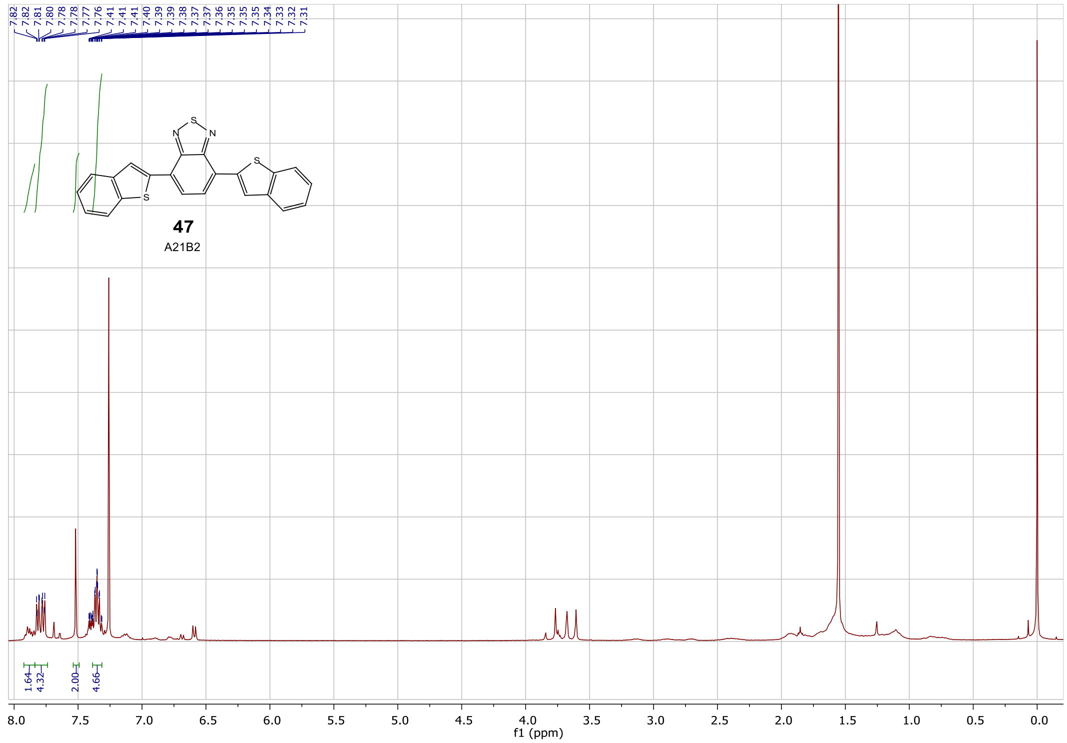

Supplementary Figure 91. ^{1}H NMR of **compound 47 (A21B2)** in $CDCl_3$.

^{1}H NMR (400 MHz, Chloroform-*d*) δ 7.92 – 7.84 (m, 2H), 7.79 (m, 4H), 7.52 (s, 2H), 7.39 – 7.31 (m, 5H).

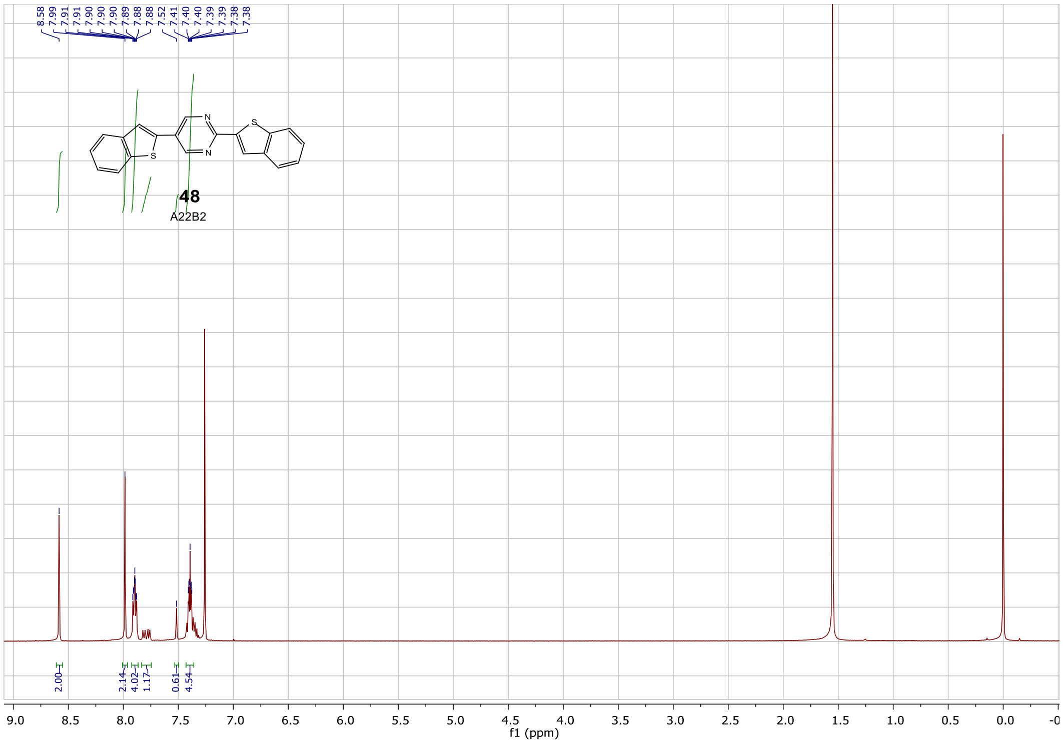

Supplementary Figure 92. ^{1}H NMR of **compound 48 (A22B2)** in $CDCl_3$.

^{1}H NMR (400 MHz, Chloroform-*d*) δ 8.58 (s, 2H), 7.99 (s, 2H), 7.92 – 7.87 (m, 4H), 7.83 – 7.75 (m, 1H), 7.52 (s, 1H), 7.43 – 7.36 (m, 5H).

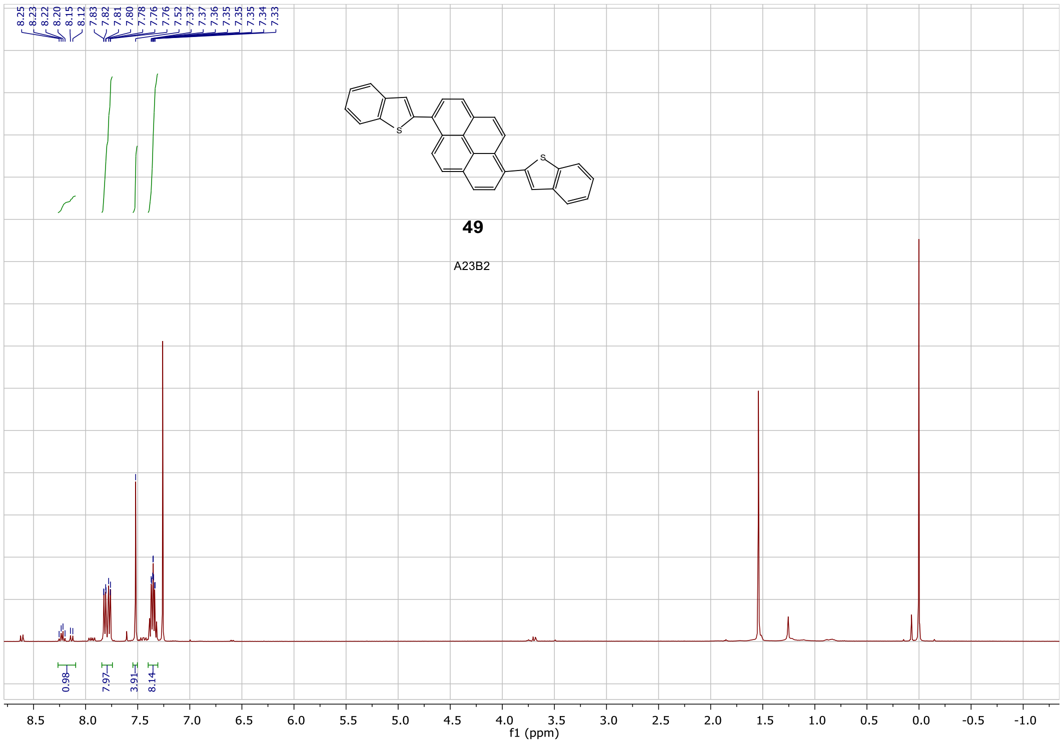

Supplementary Figure 93. ^{1}H NMR of **compound 49 (A23B2)** in $CDCl_3$.

^{1}H NMR (400 MHz, Chloroform-*d*) δ 8.27 – 8.10 (m, 1H), 7.84 – 7.74 (m, 8H), 7.52 (s, 4H), 7.40 – 7.31 (m, 8H).

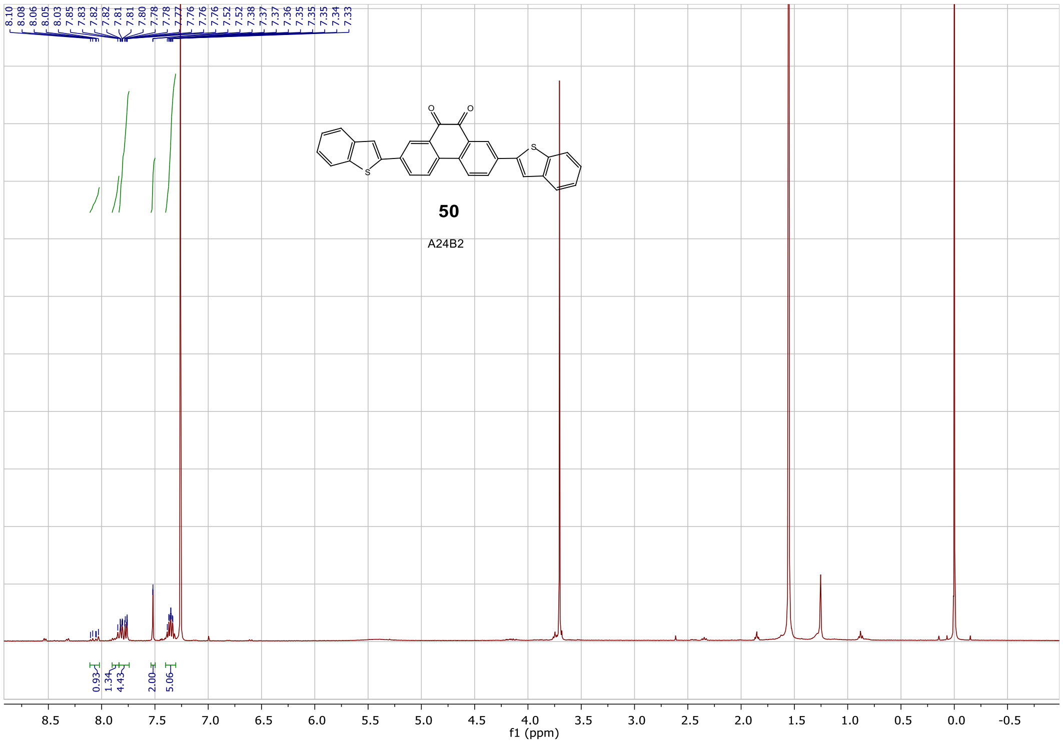

Supplementary Figure 94. ^1H NMR of **compound 50 (A24B2)** in $CDCl_3$.

^1H NMR (400 MHz, Chloroform-*d*) δ 8.11 – 8.02 (m, 1H), 7.85 (s, 1H), 7.84 – 7.74 (m, 4H), 7.52 (s, 2H), 7.40 – 7.30 (m, 5H).

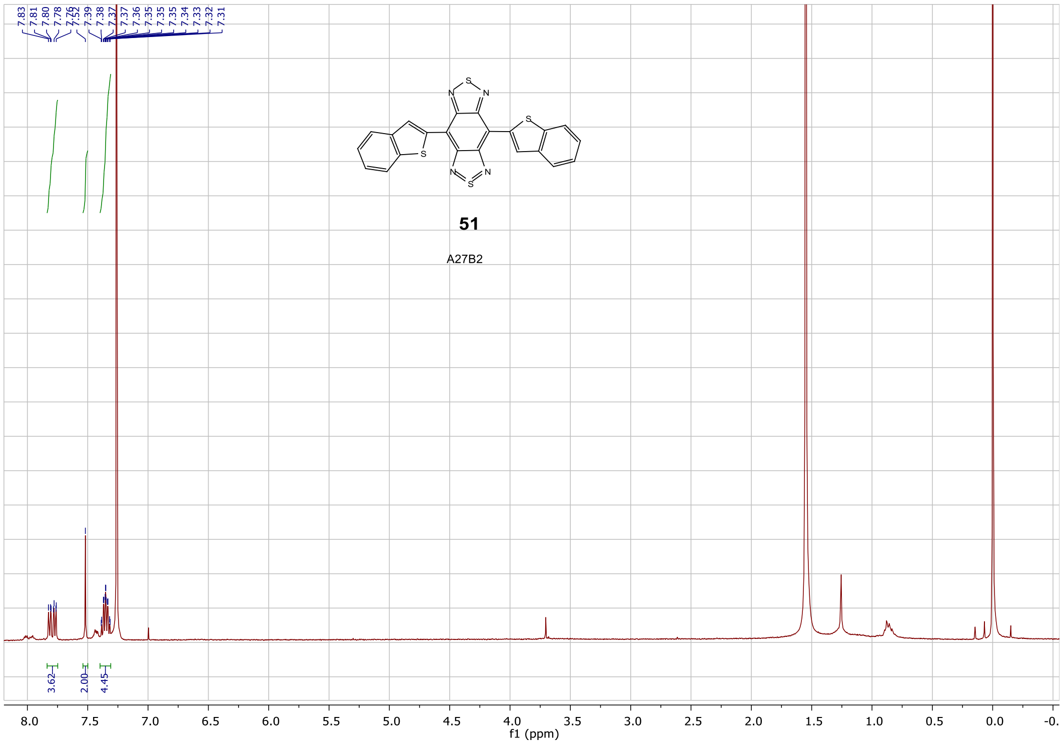

Supplementary Figure 95. ^{1}H NMR of **compound 51 (A27B2)** in $CDCl_3$.

^{1}H NMR (400 MHz, Chloroform-*d*) δ 7.84 – 7.75 (m, 4H), 7.52 (s, 2H), 7.40 – 7.31 (m, 4H).

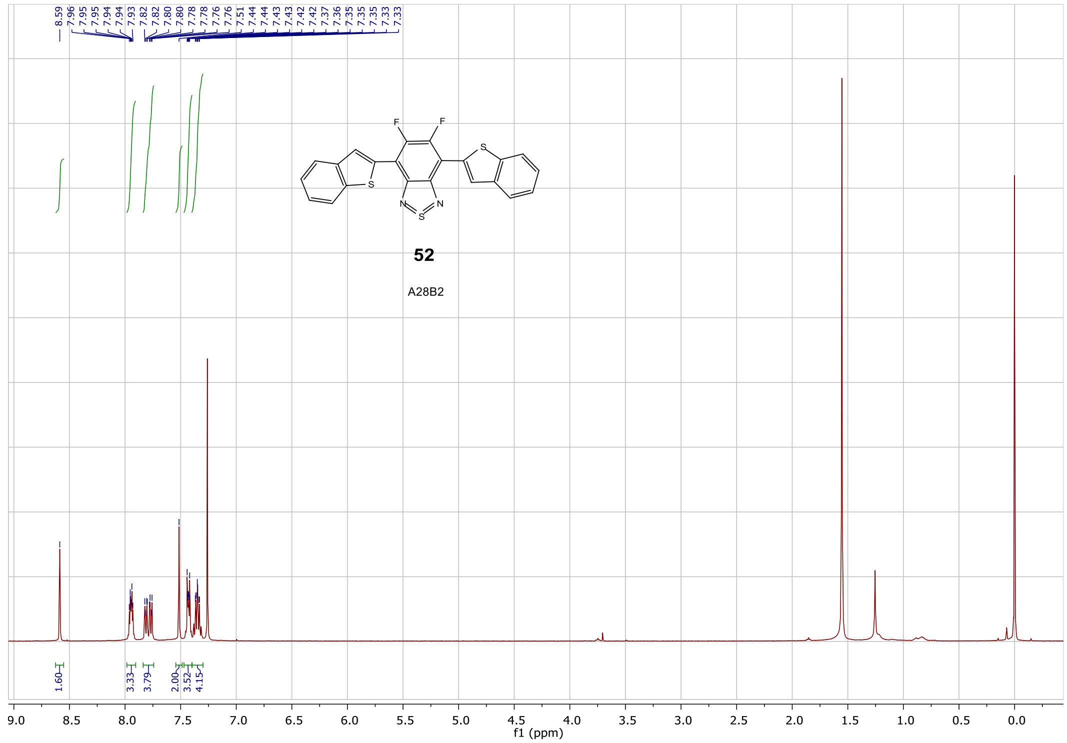

Supplementary Figure 96. ^{1}H NMR of **compound 52 (A28B2)** in $CDCl_3$.

^{1}H NMR (400 MHz, Chloroform-*d*) δ 8.59 (s, 2H), 7.98 – 7.90 (m, 3H), 7.84 – 7.74 (m, 4H), 7.51 (s, 2H), 7.47 – 7.40 (m, 4H), 7.40 – 7.30 (m, 4H).

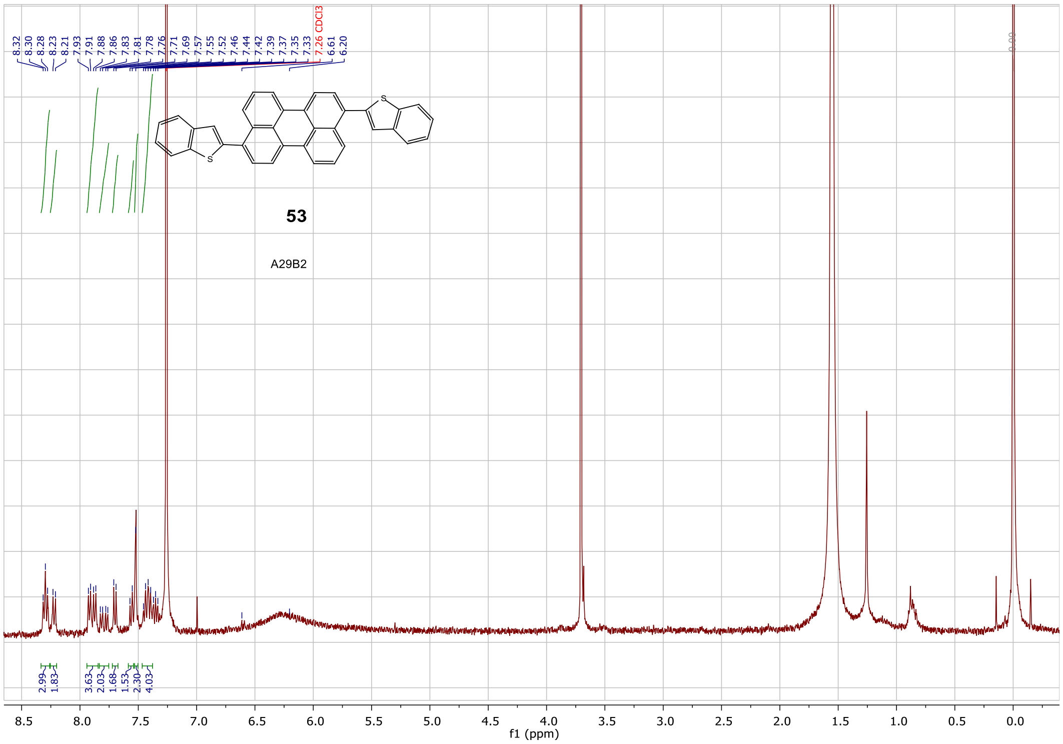

Supplementary Figure 97. ^{1}H NMR of **compound 53 (A29B2)** in $CDCl_3$.

^{1}H NMR (400 MHz, Chloroform-*d*) δ 8.30 (t, *J* = 7.7 Hz, 3H), 8.22 (d, *J* = 8.5 Hz, 2H), 7.94 – 7.84 (m, 4H), 7.83 – 7.75 (m, 2H), 7.70 (d, *J* = 7.8 Hz, 2H), 7.56 (d, *J* = 8.0 Hz, 2H), 7.52 (s, 3H), 7.47 – 7.38 (m, 4H).

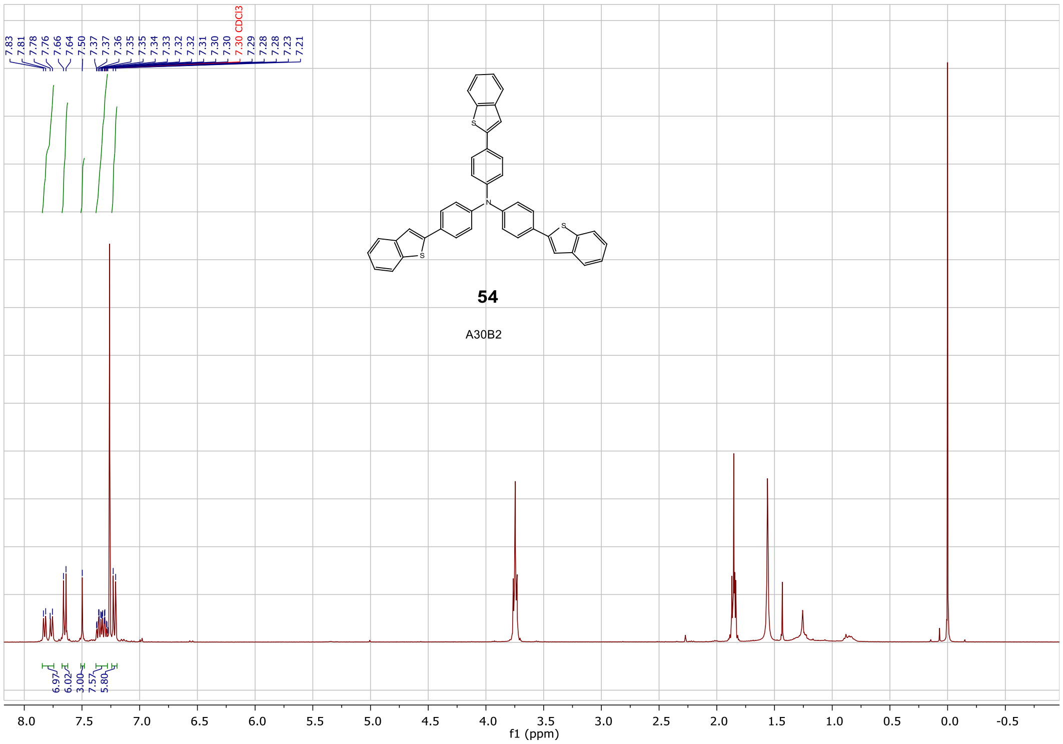

Supplementary Figure 98. ^{1}H NMR of **compound 54 (A30B2)** in $CDCl_3$.

^{1}H NMR (400 MHz, Chloroform-*d*) δ 7.84 – 7.74 (m, 6H), 7.65 (d, *J* = 8.7 Hz, 6H), 7.50 (s, 3H), 7.38 – 7.28 (m, 6H), 7.22 (d, *J* = 8.7 Hz, 6H).

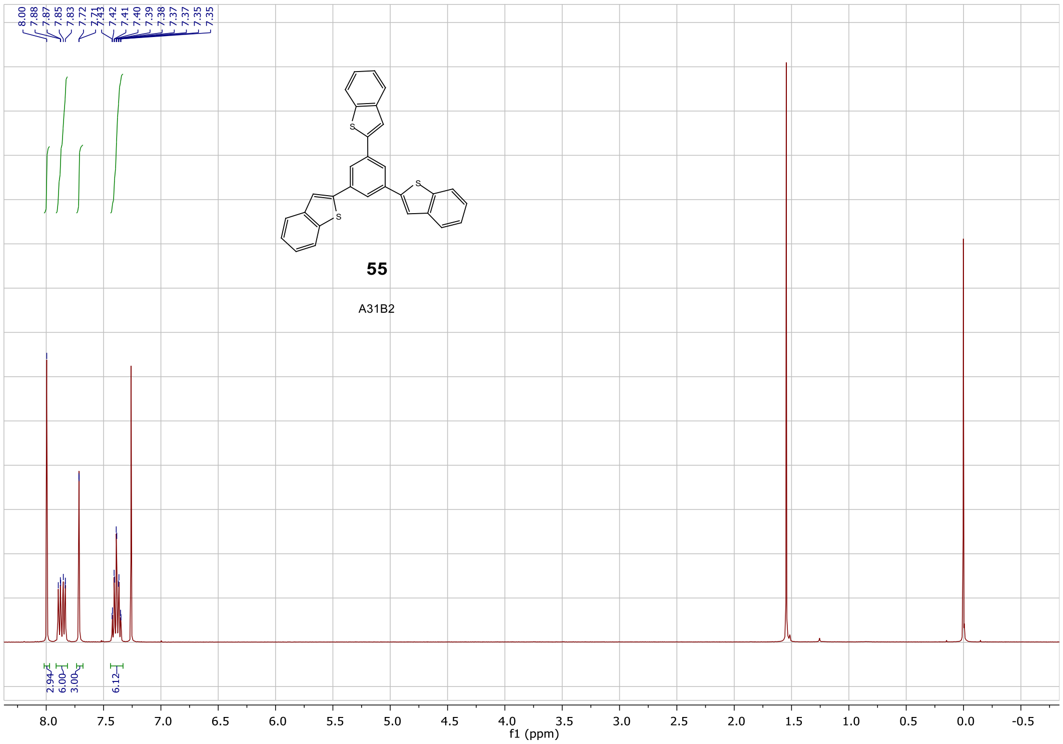

Supplementary Figure 99. ^{1}H NMR of **compound 55 (A31B2)** in $CDCl_3$.

^{1}H NMR (400 MHz, Chloroform-*d*) δ 8.00 (s, 3H), 7.91 – 7.81 (m, 6H), 7.71 (s, 3H), 7.44 – 7.33 (m, 6H).

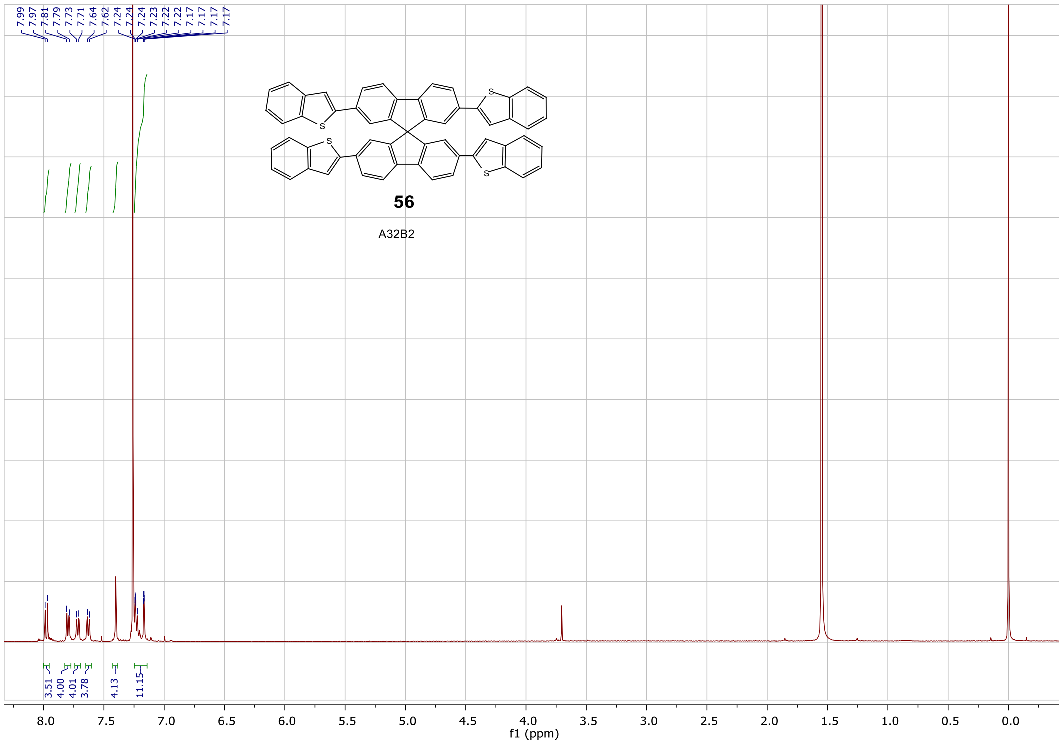

Supplementary Figure 100. ^{1}H NMR of **compound 56 (A32B2)** in $CDCl_3$.

^{1}H NMR (400 MHz, Chloroform-*d*) δ 7.98 (d, *J* = 8.1 Hz, 4H), 7.80 (d, *J* = 9.7 Hz, 4H), 7.72 (d, *J* = 7.1 Hz, 4H), 7.63 (d, *J* = 7.1 Hz, 4H), 7.25 – 7.14 (m, 11H).

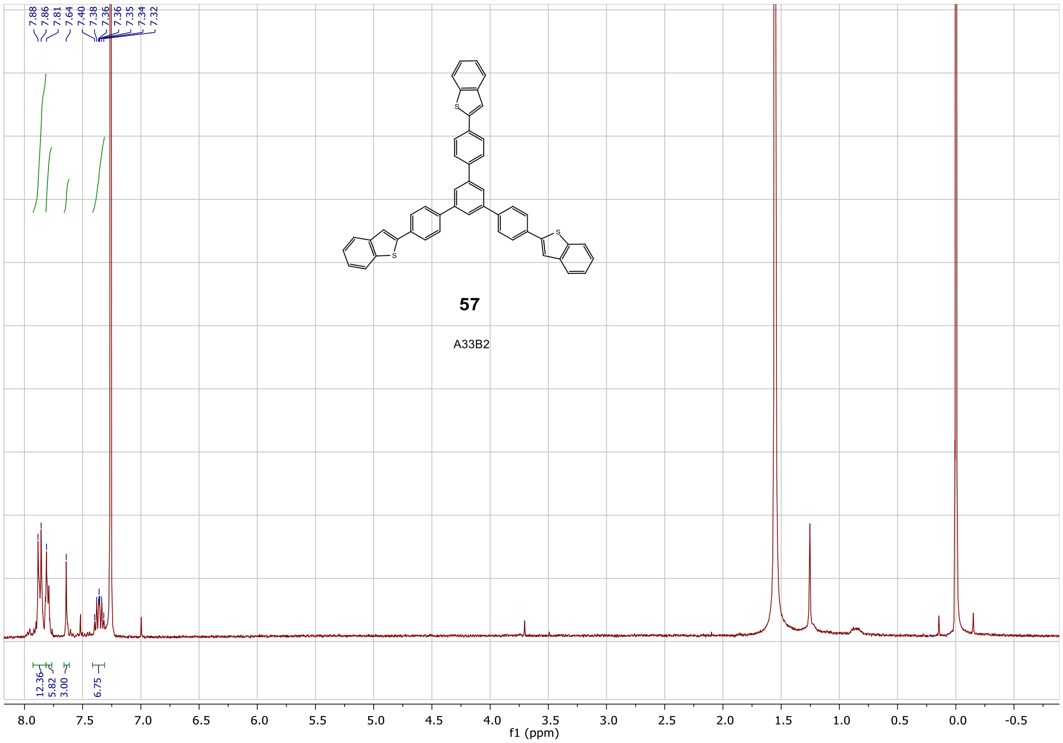

Supplementary Figure 101. ^{1}H NMR of **compound 57 (A33B2)** in $CDCl_3$.

^{1}H NMR (400 MHz, Chloroform-*d*) δ 7.90 – 7.83 (m, 12H), 7.81 (s, 6H), 7.64 (s, 3H), 7.42 – 7.31 (m, 6H).

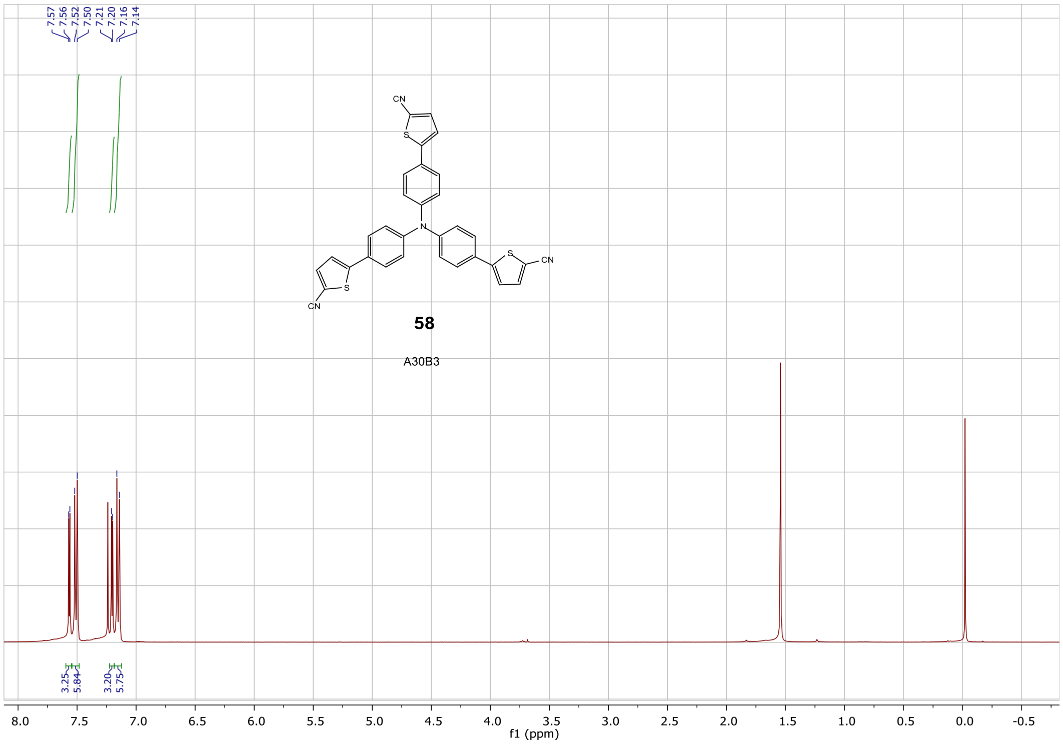

Supplementary Figure 102. ^1H NMR of **compound 58 (A30B3)** in $CDCl_3$.

^1H NMR (400 MHz, Chloroform-*d*) δ 7.57 (d, *J* = 3.9 Hz, 3H), 7.51 (d, *J* = 8.7 Hz, 6H), 7.20 (d, *J* = 4.0 Hz, 3H), 7.15 (d, *J* = 8.7 Hz, 6H).

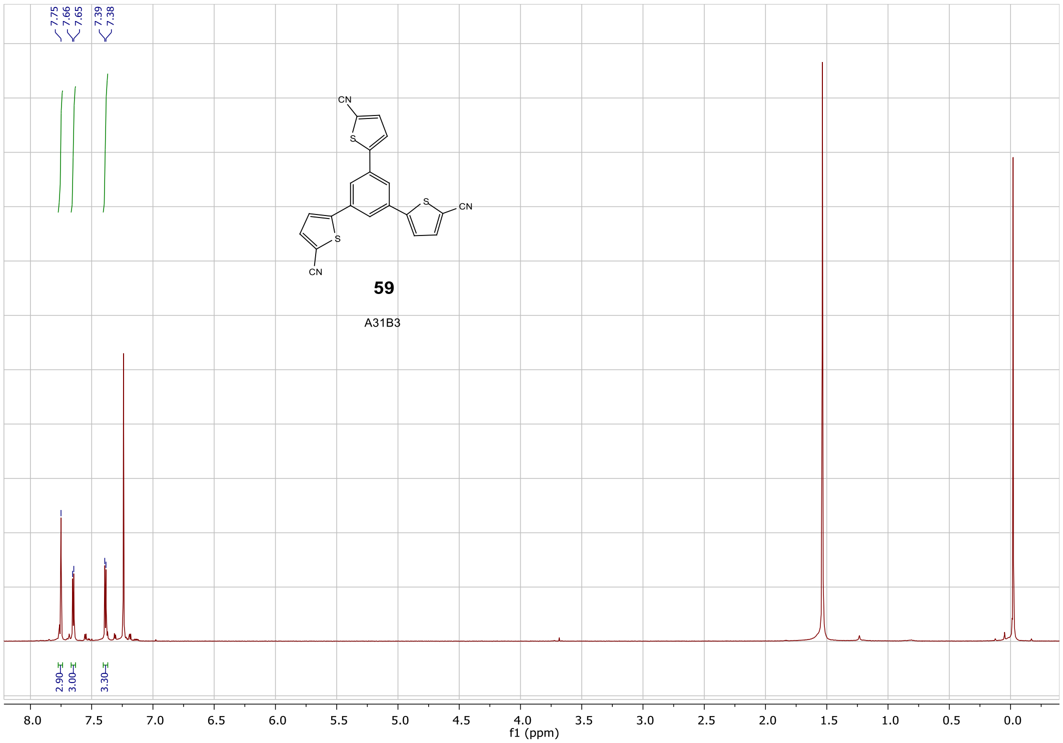

Supplementary Figure 103. ^{1}H NMR of **compound 59 (A31B3)** in $CDCl_3$.

^{1}H NMR (400 MHz, Chloroform-*d*) δ 7.75 (s, 3H), 7.65 (d, *J* = 3.9 Hz, 3H), 7.39 (d, *J* = 3.9 Hz, 3H).

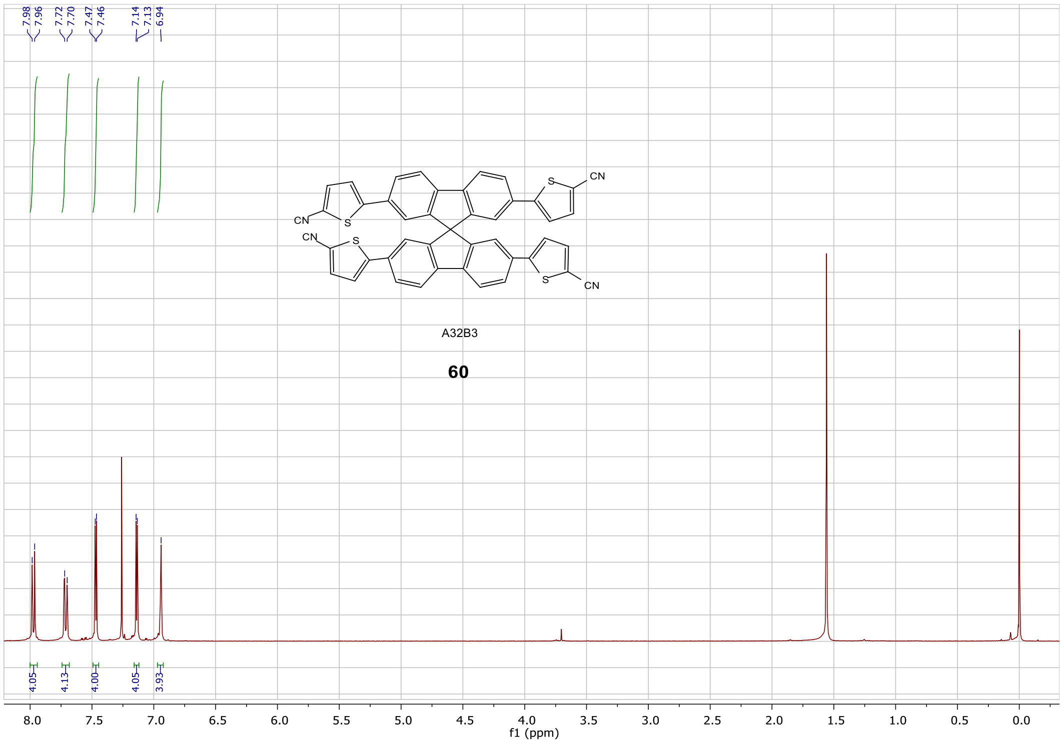

Supplementary Figure 104. ^1H NMR of **compound 60 (A32B3)** in $CDCl_3$.

^1H NMR (400 MHz, Chloroform-*d*) δ 7.97 (d, *J* = 8.0 Hz, 4H), 7.71 (d, *J* = 8.0 Hz, 4H), 7.47 (d, *J* = 4.0 Hz, 4H), 7.14 (d, *J* = 4.0 Hz, 4H), 6.94 (s, 4H).

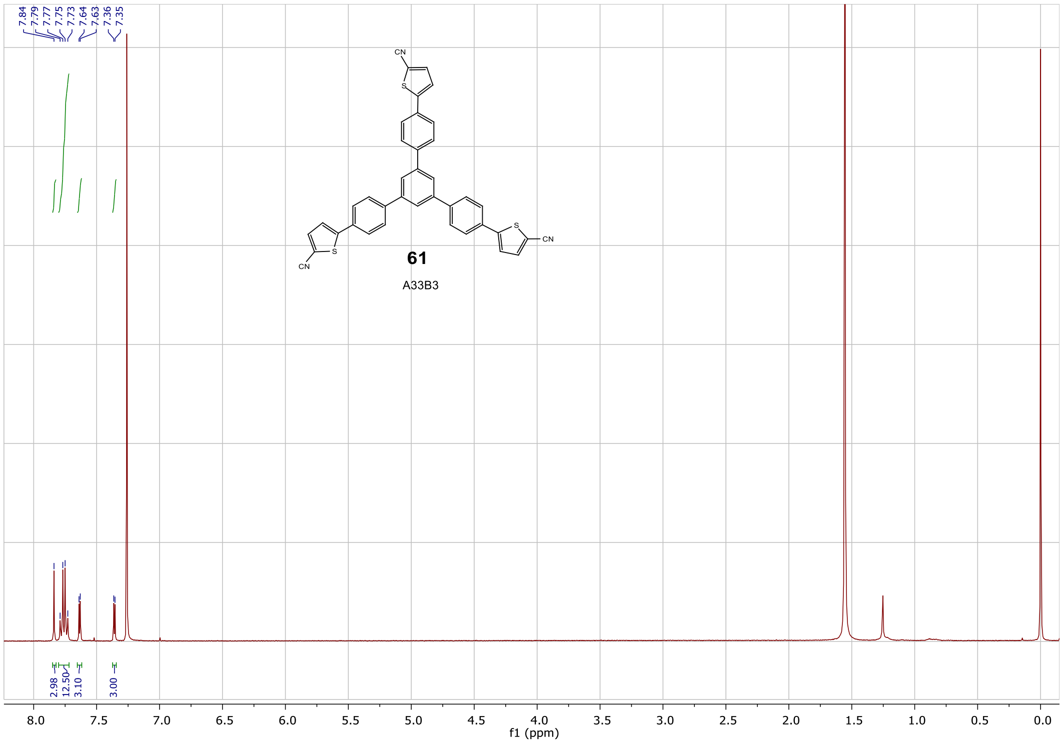

Supplementary Figure 105. ^{1}H NMR of **compound 61 (A33B3)** in $CDCl_3$.

^{1}H NMR (400 MHz, Chloroform-*d*) δ 7.84 (s, 3H), 7.76 (q, *J* = 8.5 Hz, 12H), 7.63 (d, *J* = 3.9 Hz, 3H), 7.36 (d, *J* = 3.9 Hz, 3H).

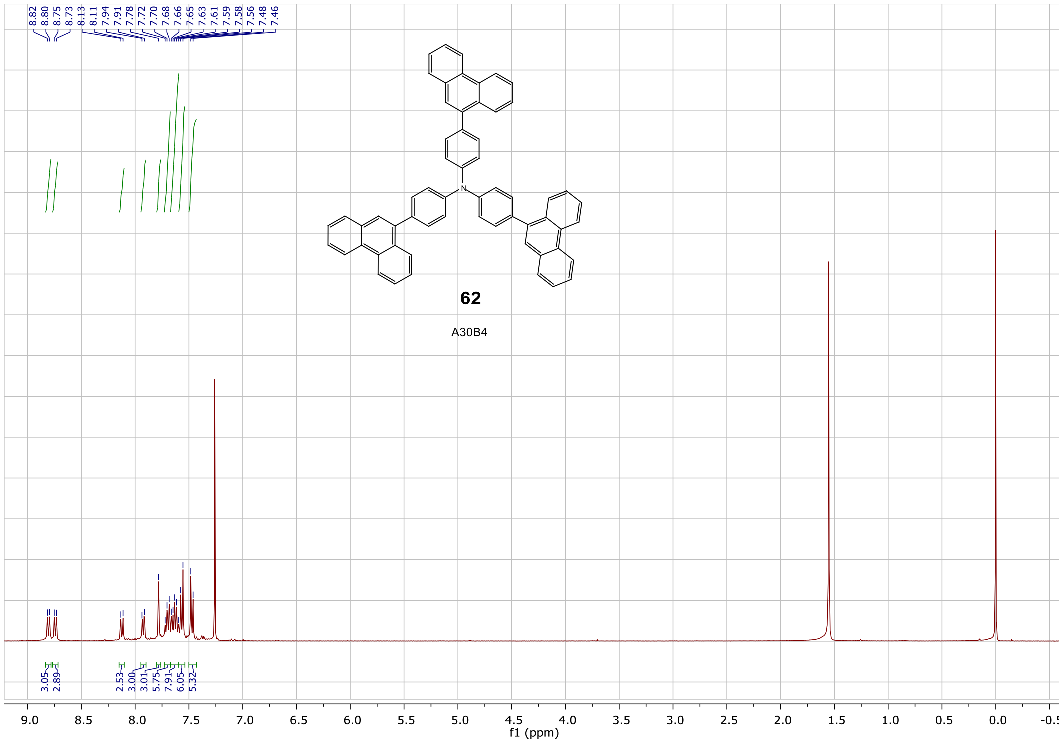

Supplementary Figure 106. ^1H NMR of **compound 62 (A30B4)** in $CDCl_3$.

^1H NMR (400 MHz, Chloroform-*d*) δ 8.81 (d, *J* = 8.0 Hz, 3H), 8.74 (d, *J* = 8.2 Hz, 3H), 8.12 (d, *J* = 8.2 Hz, 2H), 7.93 (d, *J* = 9.0 Hz, 3H), 7.78 (s, 3H), 7.73 – 7.67 (m, 6H), 7.67 – 7.59 (m, 8H), 7.57 (d, *J* = 8.5 Hz, 6H), 7.47 (d, *J* = 8.5 Hz, 5H).

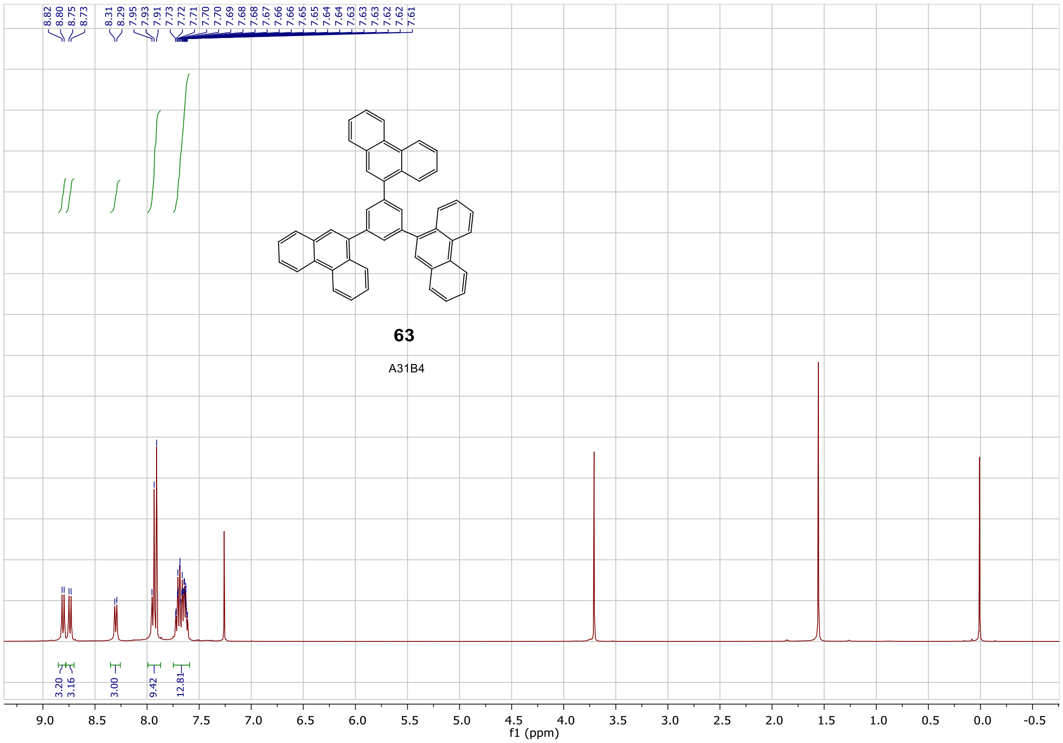

Supplementary Figure 107. ^{1}H NMR of **compound 63 (A31B4)** in $CDCl_3$.

^{1}H NMR (400 MHz, Chloroform-*d*) δ 8.81 (d, *J* = 7.9 Hz, 3H), 8.74 (d, *J* = 8.2 Hz, 3H), 8.30 (d, *J* = 7.9 Hz, 3H), 7.99 – 7.87 (m, 9H), 7.75 – 7.59 (m, 13H).

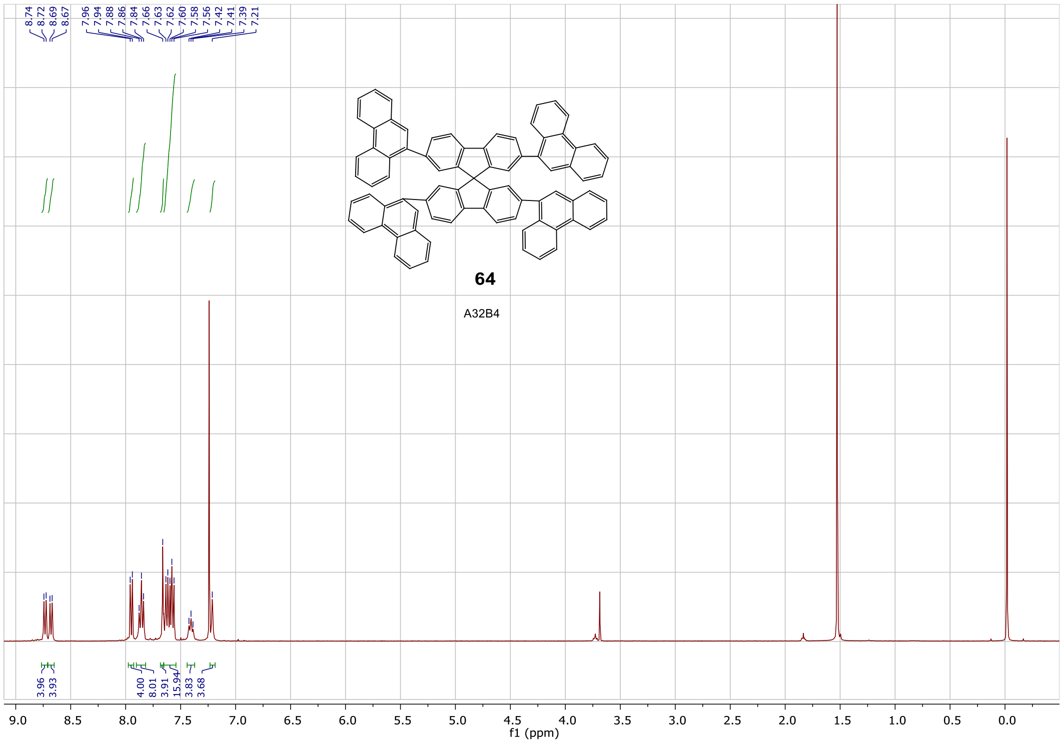

Supplementary Figure 108. ^{1}H NMR of **compound 64 (A32B4)** in $CDCl_3$.

^{1}H NMR (400 MHz, Chloroform-*d*) δ 8.73 (d, *J* = 8.2 Hz, 4H), 8.68 (d, *J* = 8.1 Hz, 4H), 7.95 (d, *J* = 7.8 Hz, 4H), 7.86 (t, *J* = 8.0 Hz, 8H), 7.66 (s, 4H), 7.65 – 7.54 (m, 16H), 7.41 (t, *J* = 7.3 Hz, 4H), 7.21 (s, 4H).

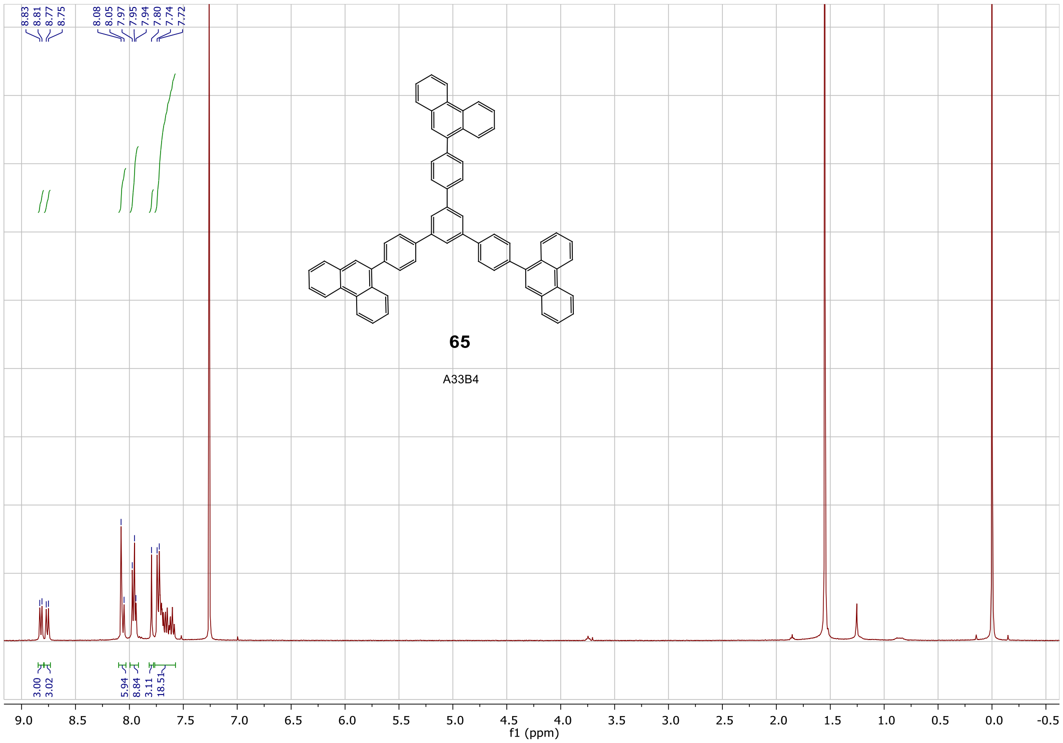

Supplementary Figure 109. ^{1}H NMR of **compound 65 (A33B4)** in $CDCl_3$.

^{1}H NMR (400 MHz, Chloroform-*d*) δ 8.82 (d, *J* = 8.2 Hz, 3H), 8.76 (d, *J* = 8.1 Hz, 3H), 8.06 (d, *J* = 11.0 Hz, 6H), 7.96 (t, *J* = 6.7 Hz, 9H), 7.80 (s, 3H), 7.76 – 7.57 (m, 19H).

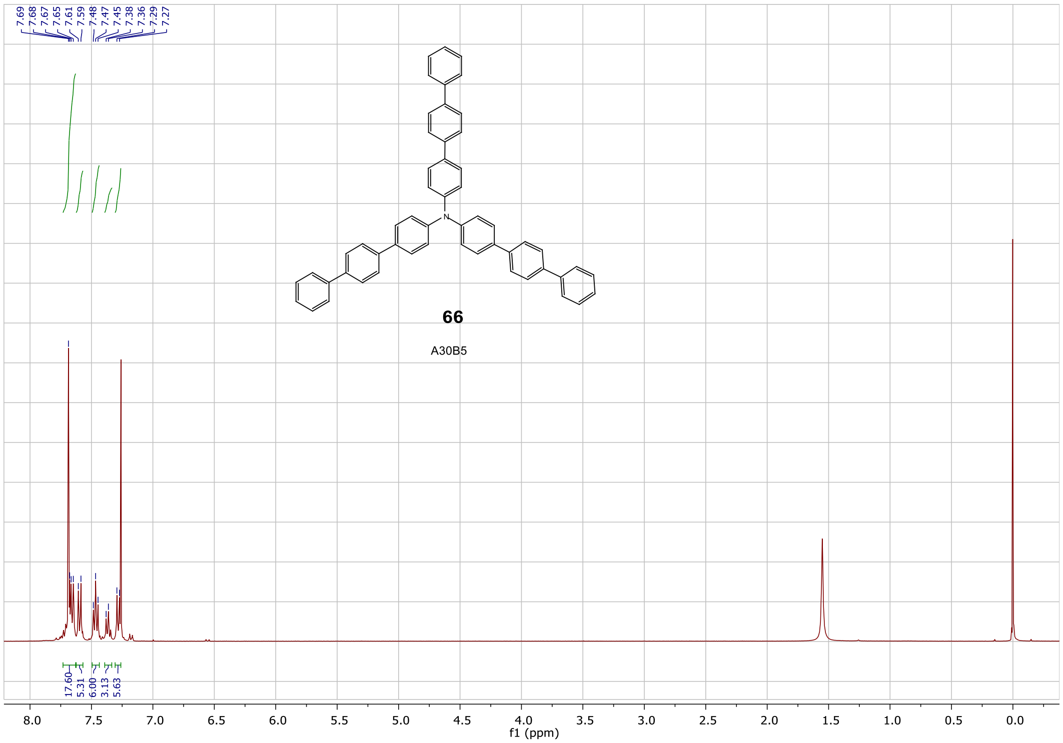

Supplementary Figure 110. ^{1}H NMR of **compound 66 (A30B5)** in $CDCl_3$.

^{1}H NMR (400 MHz, Chloroform-*d*) δ 7.73 – 7.63 (m, 18H), 7.60 (d, *J* = 8.6 Hz, 5H), 7.47 (t, *J* = 7.6 Hz, 6H), 7.36 (t, *J* = 7.4 Hz, 3H), 7.28 (d, *J* = 8.6 Hz, 6H).

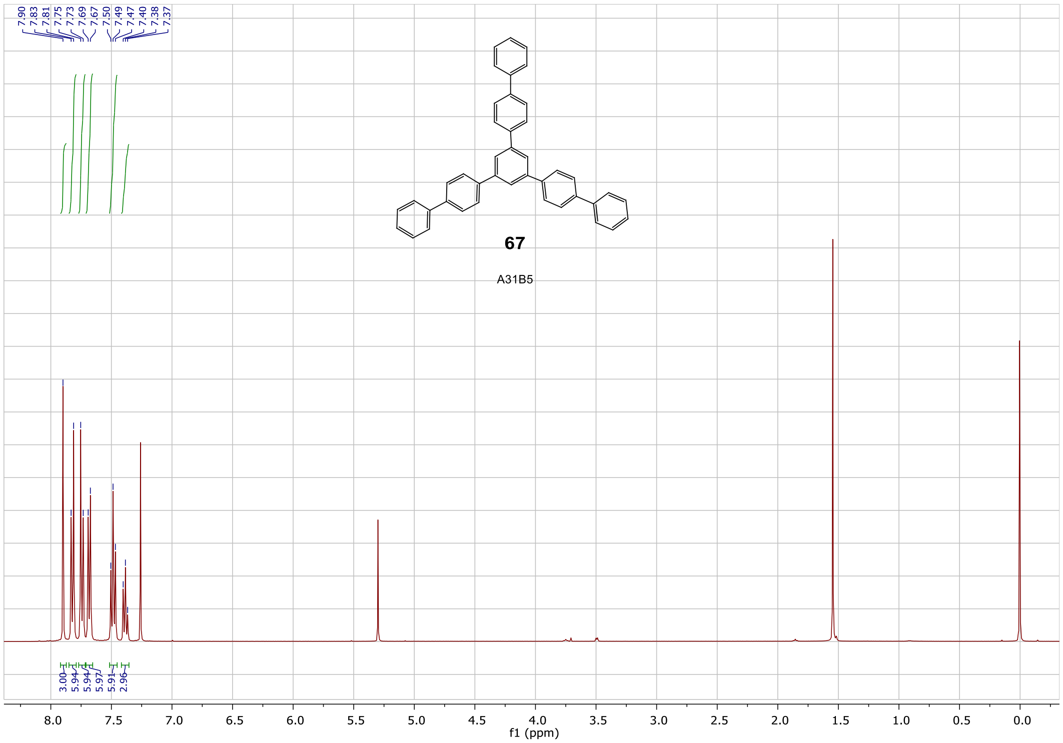

Supplementary Figure 111. ^{1}H NMR of **compound 67 (A31B5)** in $CDCl_3$.

^{1}H NMR (400 MHz, Chloroform-*d*) δ 7.90 (s, 3H), 7.82 (d, *J* = 8.3 Hz, 6H), 7.74 (d, *J* = 8.3 Hz, 6H), 7.68 (d, *J* = 7.2 Hz, 6H), 7.49 (t, *J* = 7.6 Hz, 6H), 7.38 (t, *J* = 7.4 Hz, 3H).

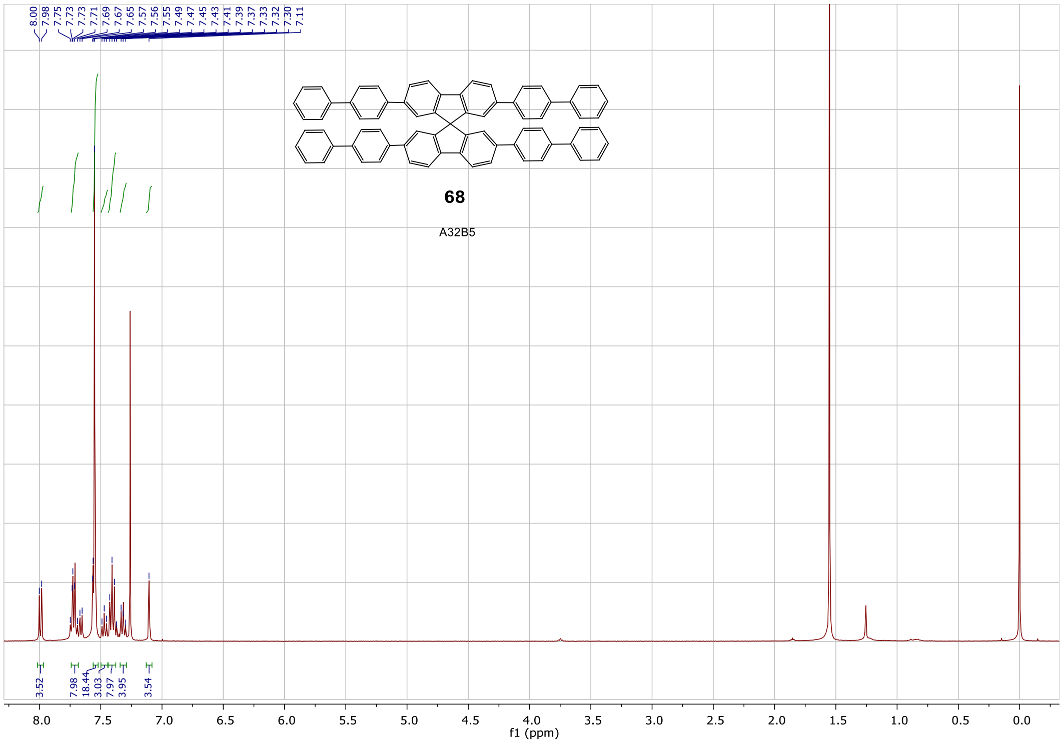

Supplementary Figure 112. ^{1}H NMR of **compound 68 (A32B5)** in $CDCl_3$.

^{1}H NMR (400 MHz, Chloroform-*d*) δ 7.99 (d, *J* = 8.0 Hz, 4H), 7.74 – 7.68 (m, 8H), 7.55 (s, 18H), 7.47 (t, *J* = 7.6 Hz, 3H), 7.41 (t, *J* = 7.6 Hz, 8H), 7.33 (s, 4H), 7.11 (s, 4H).

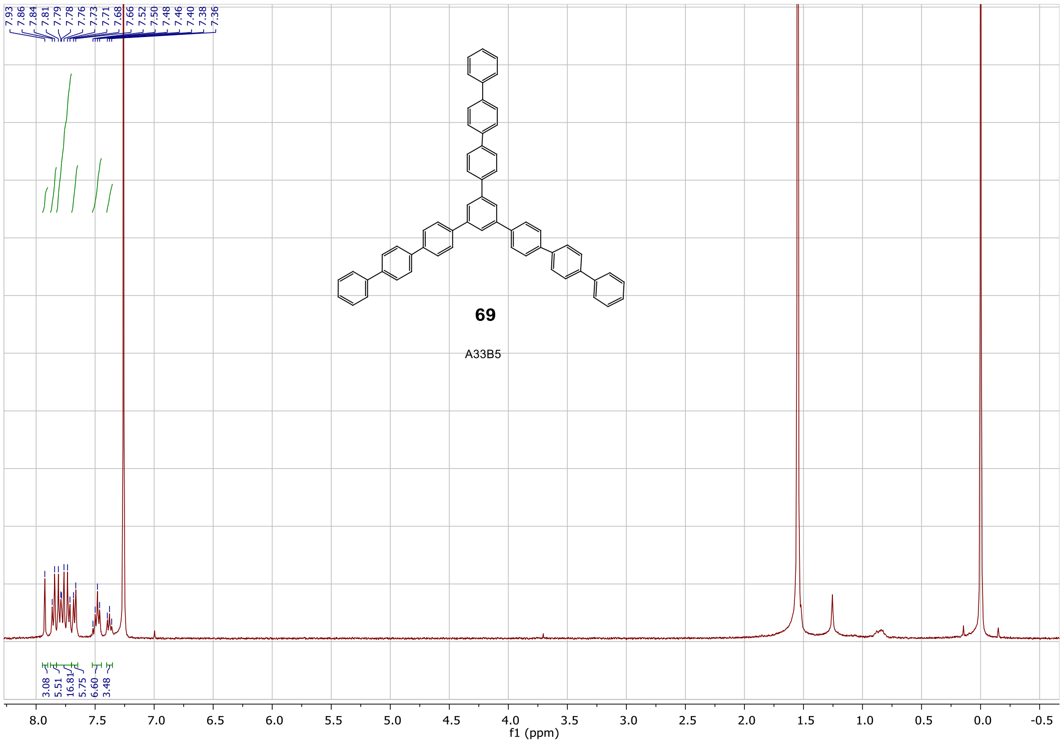

Supplementary Figure 113. ^{1}H NMR of **compound 69 (A33B5)** in $CDCl_3$.

^{1}H NMR (400 MHz, Chloroform-*d*) δ 7.93 (s, 3H), 7.85 (d, *J* = 8.2 Hz, 6H), 7.83 – 7.70 (m, 17H), 7.67 (d, *J* = 7.7 Hz, 6H), 7.48 (t, *J* = 7.5 Hz, 7H), 7.40 – 7.35 (m, 3H).

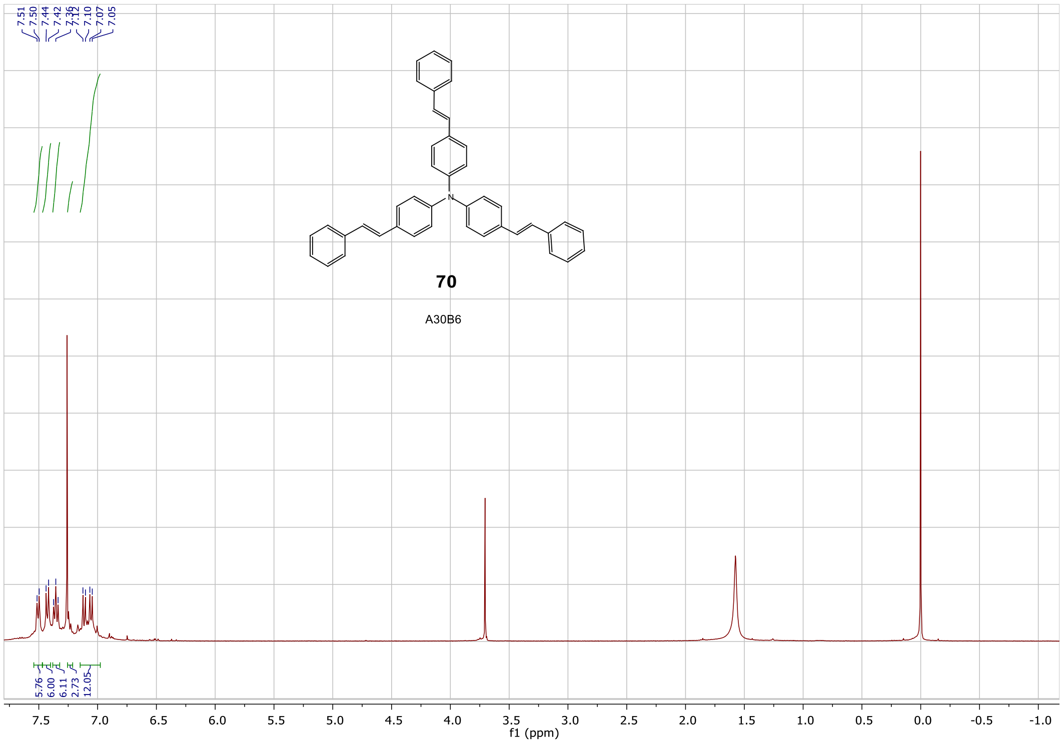

Supplementary Figure 114. ^{1}H NMR of **compound 70 (A30B6)** in $CDCl_3$.

^{1}H NMR (400 MHz, Chloroform-*d*) δ 7.51 (d, *J* = 7.3 Hz, 6H), 7.43 (d, *J* = 8.6 Hz, 6H), 7.36 (t, *J* = 7.6 Hz, 6H), 7.26 – 7.21 (m, 3H), 7.15 – 6.98 (m, 12H).

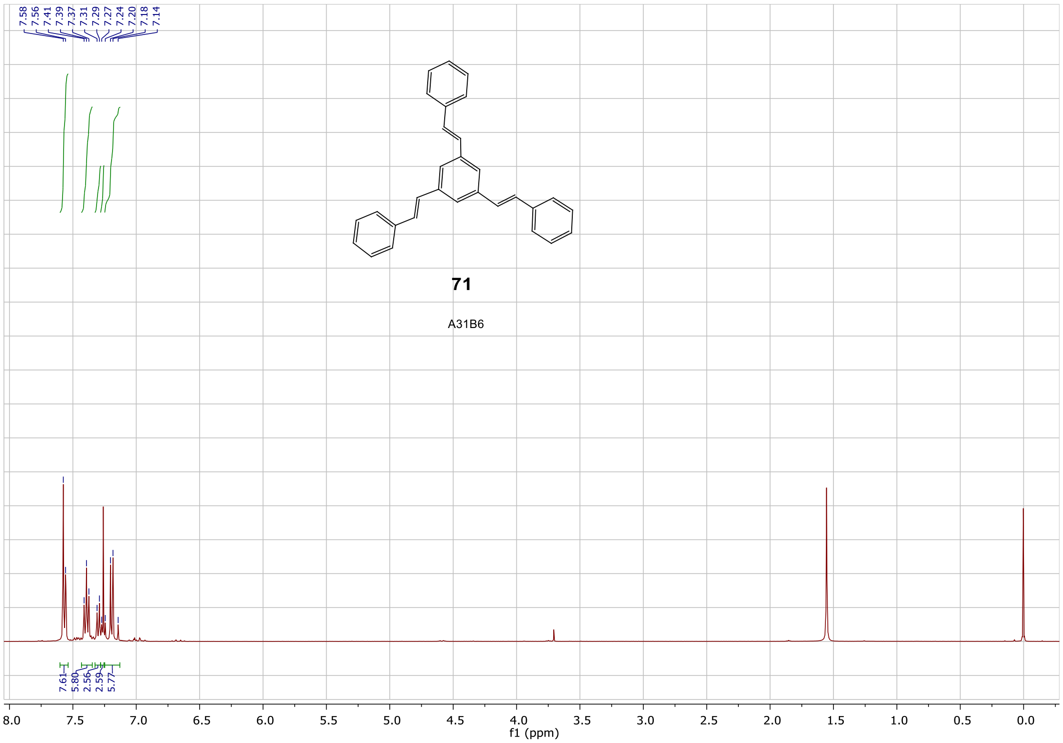

Supplementary Figure 115. ^1H NMR of **compound 71 (A31B6)** in $CDCl_3$.

^1H NMR (400 MHz, Chloroform-*d*) δ 7.57 (d, *J* = 7.1 Hz, 8H), 7.39 (t, *J* = 7.6 Hz, 6H), 7.30 (d, *J* = 7.4 Hz, 3H), 7.27 (s, 3H), 7.25 – 7.13 (m, 6H).

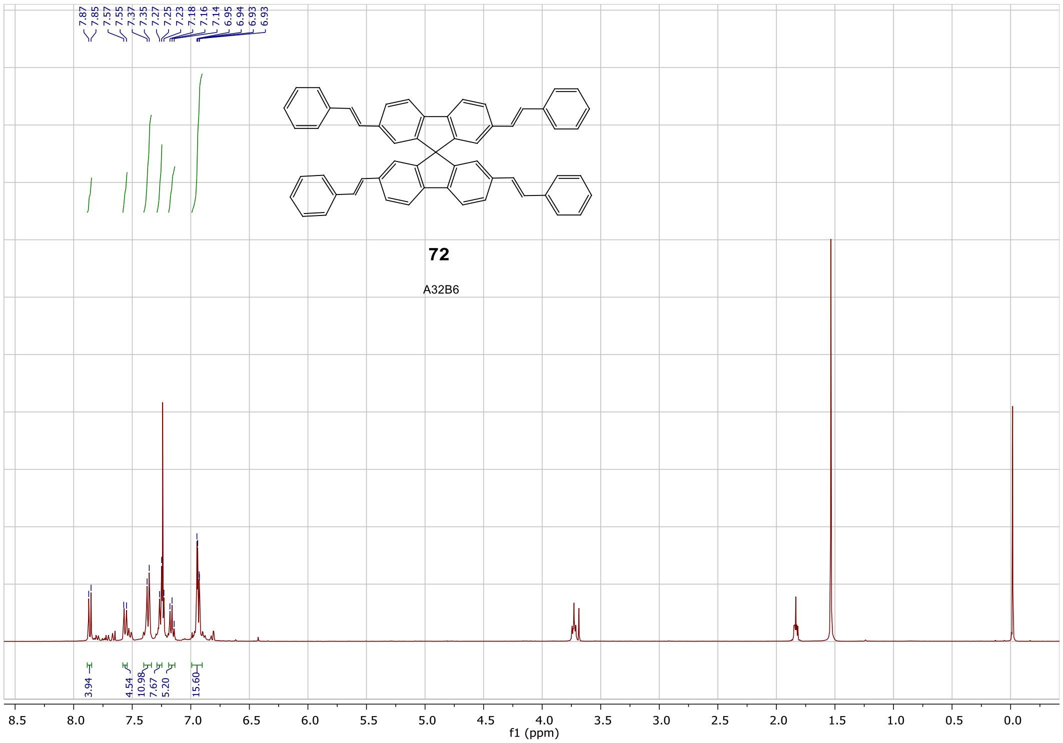

Supplementary Figure 116. ^{1}H NMR of **compound 72 (A32B6)** in $CDCl_3$.

^{1}H NMR (400 MHz, Chloroform-*d*) δ 7.86 (d, *J* = 8.0 Hz, 4H), 7.56 (d, *J* = 9.5 Hz, 5H), 7.36 (d, *J* = 7.3 Hz, 11H), 7.26 (d, *J* = 7.3 Hz, 8H), 7.16 (t, *J* = 7.3 Hz, 5H), 6.99 – 6.90 (m, 16H).

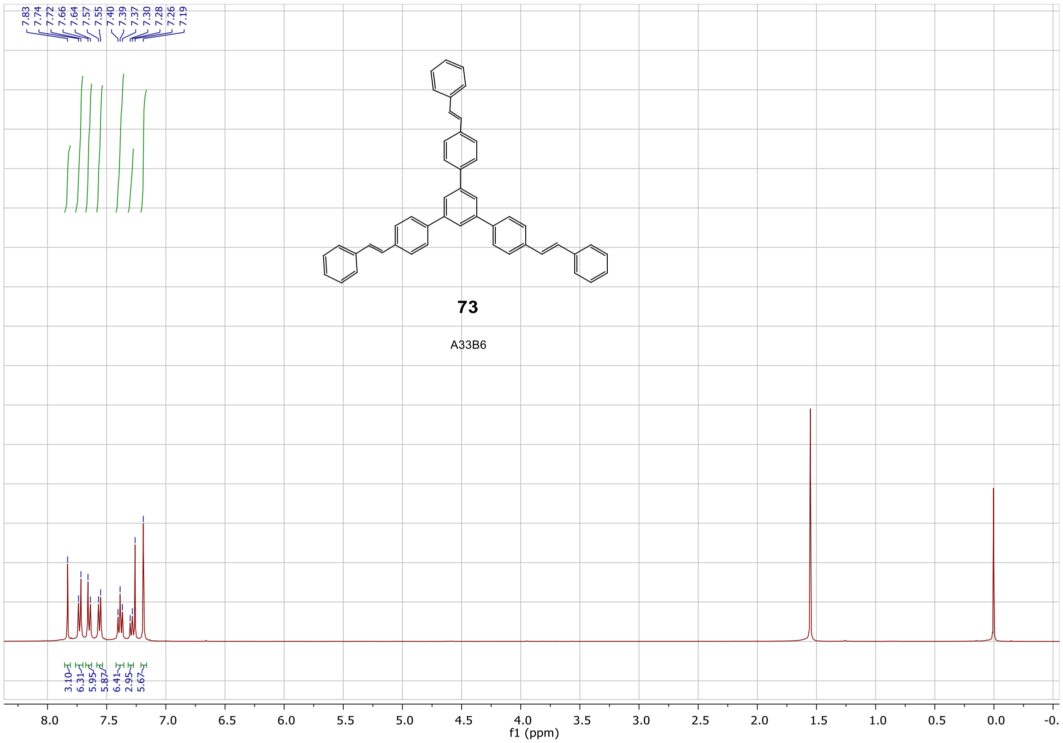

Supplementary Figure 117. ^{1}H NMR of **compound 73 (A33B6)** in $CDCl_3$.

^{1}H NMR (400 MHz, Chloroform-*d*) δ 7.83 (s, 3H), 7.73 (d, *J* = 8.3 Hz, 6H), 7.65 (d, *J* = 8.3 Hz, 6H), 7.56 (d, *J* = 7.3 Hz, 6H), 7.39 (t, *J* = 7.6 Hz, 6H), 7.29 (d, *J* = 7.3 Hz, 3H), 7.19 (s, 6H).

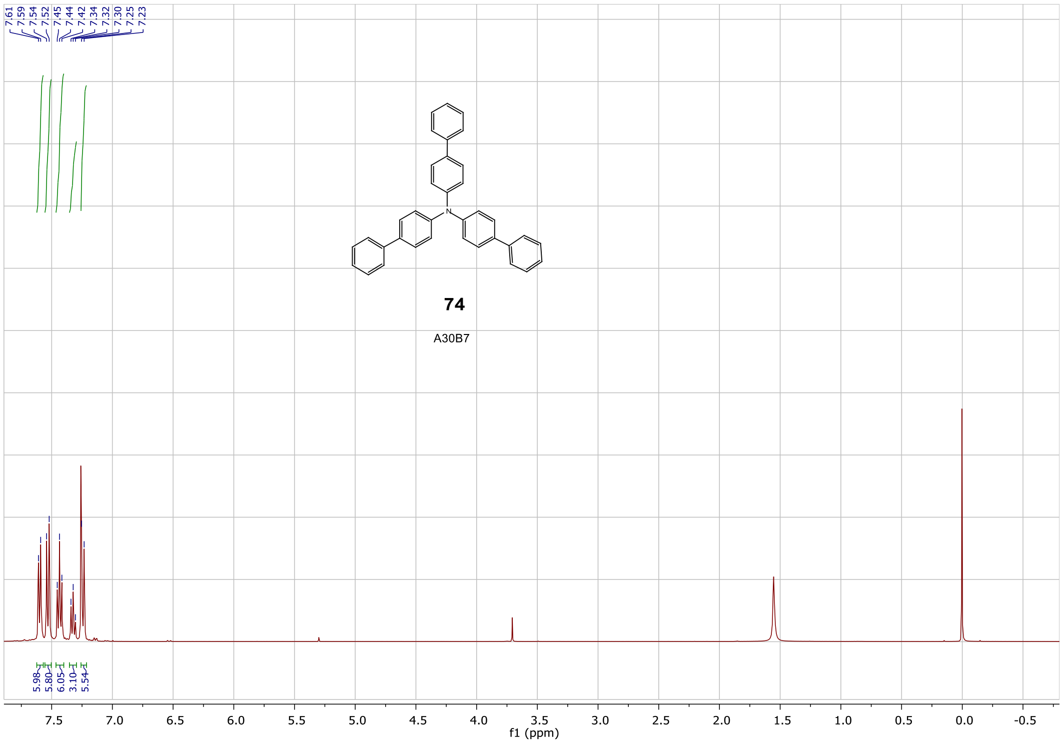

Supplementary Figure 118. ^{1}H NMR of **compound 74 (A30B7)** in $CDCl_3$.

^{1}H NMR (400 MHz, Chloroform-*d*) δ 7.60 (d, *J* = 7.2 Hz, 6H), 7.53 (d, *J* = 8.6 Hz, 6H), 7.44 (t, *J* = 7.6 Hz, 6H), 7.32 (t, *J* = 7.3 Hz, 3H), 7.24 (d, *J* = 8.6 Hz, 6H).

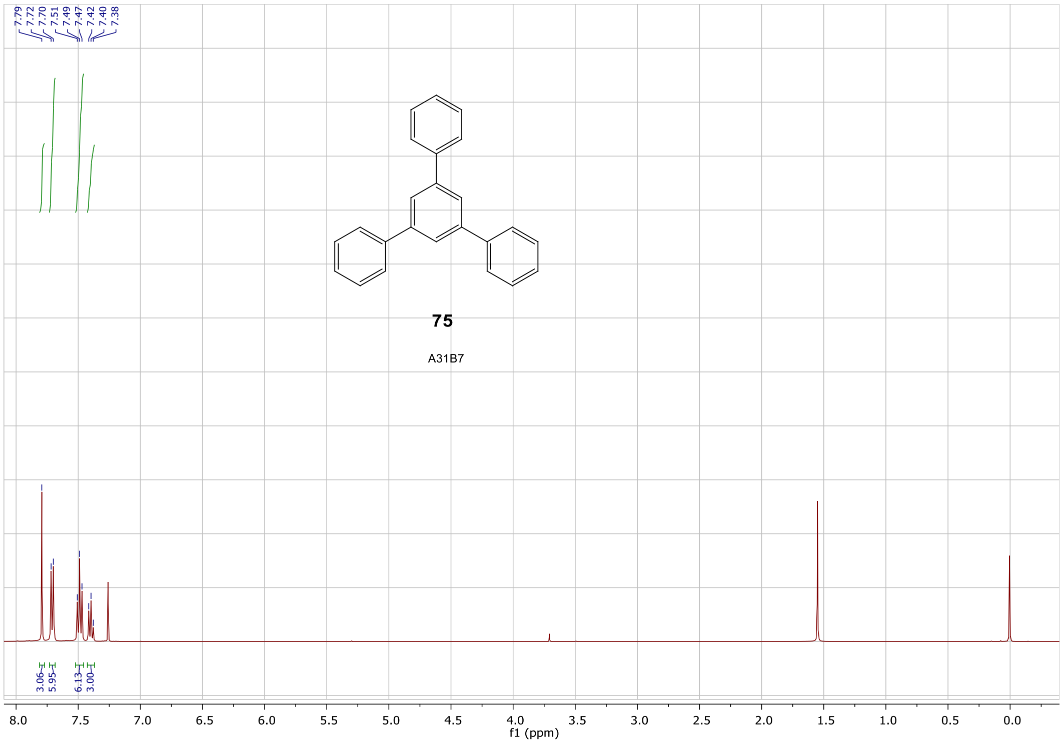

Supplementary Figure 119. ^{1}H NMR of **compound 75 (A31B7)** in $CDCl_3$.

^{1}H NMR (400 MHz, Chloroform-*d*) δ 7.79 (s, 3H), 7.71 (d, *J* = 7.1 Hz, 6H), 7.49 (t, *J* = 7.5 Hz, 6H), 7.40 (t, *J* = 7.4 Hz, 3H).

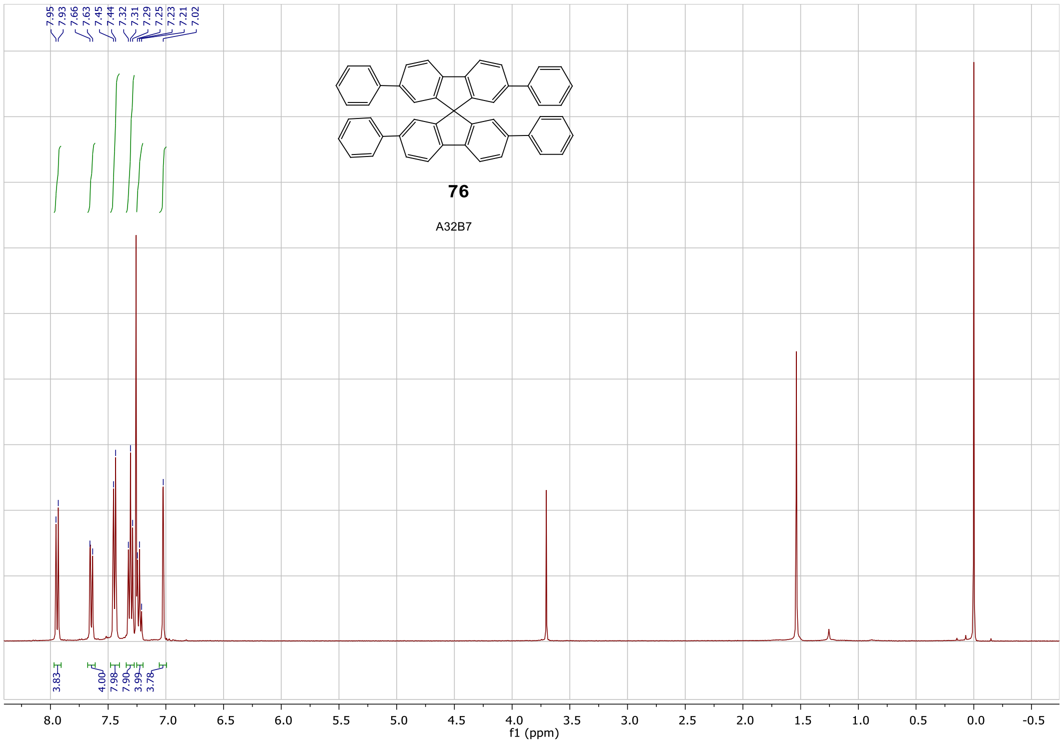

Supplementary Figure 120. ^{1}H NMR of **compound 76 (A32B7)** in $CDCl_3$.

^{1}H NMR (400 MHz, Chloroform-*d*) δ 7.94 (d, *J* = 7.9 Hz, 4H), 7.65 (d, *J* = 9.6 Hz, 4H), 7.44 (d, *J* = 7.2 Hz, 8H), 7.31 (t, *J* = 7.5 Hz, 8H), 7.23 (t, *J* = 7.3 Hz, 4H), 7.02 (s, 4H).

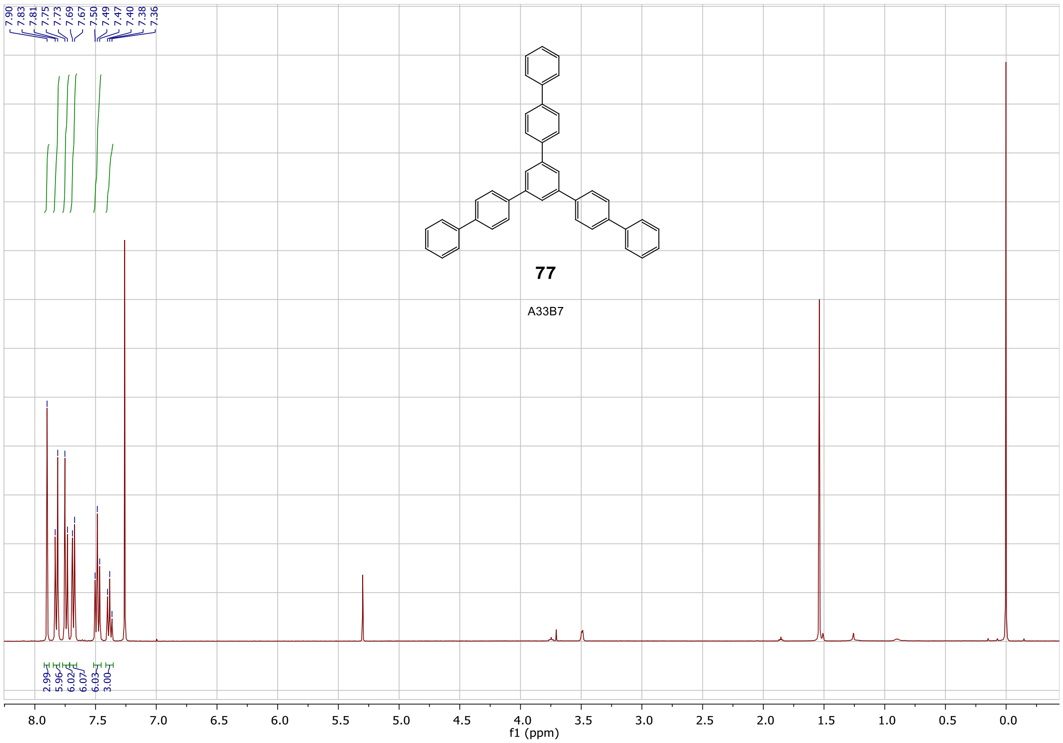

Supplementary Figure 121. ^{1}H NMR of **compound 77 (A33B7)** in $CDCl_3$.

^{1}H NMR (400 MHz, Chloroform-*d*) δ 7.90 (s, 3H), 7.82 (d, *J* = 8.4 Hz, 6H), 7.74 (d, *J* = 8.4 Hz, 6H), 7.68 (d, *J* = 7.1 Hz, 6H), 7.48 (t, *J* = 7.6 Hz, 6H), 7.38 (t, *J* = 7.4 Hz, 3H).

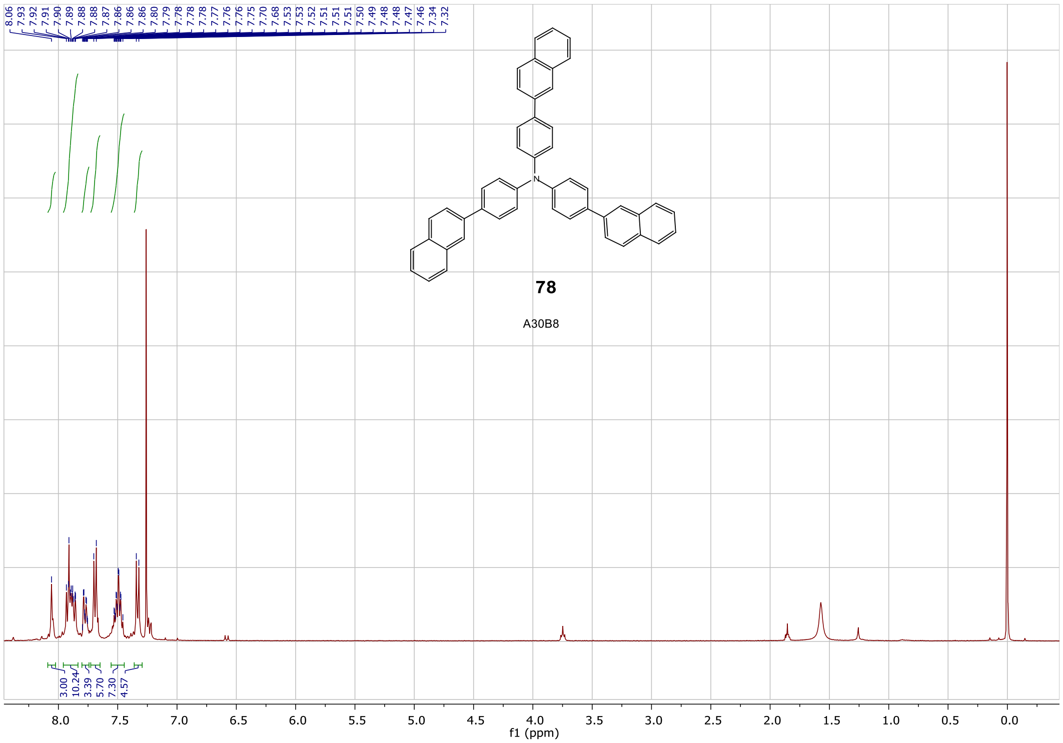

Supplementary Figure 122. ^1H NMR of **compound 78 (A30B8)** in $CDCl_3$.

^1H NMR (400 MHz, Chloroform-*d*) δ 8.06 (s, 3H), 7.96 – 7.83 (m, 10H), 7.80 – 7.74 (m, 3H), 7.69 (d, *J* = 8.5 Hz, 6H), 7.56 – 7.44 (m, 7H), 7.33 (d, *J* = 8.5 Hz, 5H).

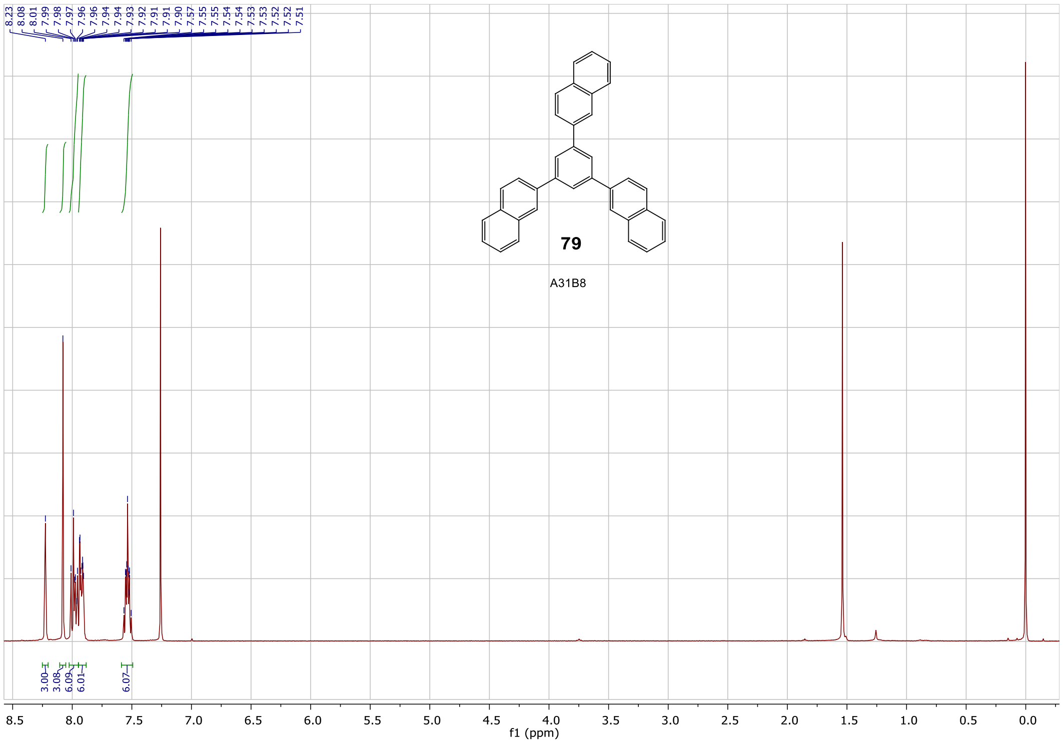

Supplementary Figure 123. ^{1}H NMR of **compound 79 (A31B8)** in $CDCl_3$.

^{1}H NMR (400 MHz, Chloroform-*d*) δ 8.23 (s, 3H), 8.08 (s, 3H), 8.03 – 7.95 (m, 6H), 7.95 – 7.88 (m, 6H), 7.59 – 7.49 (m, 6H).

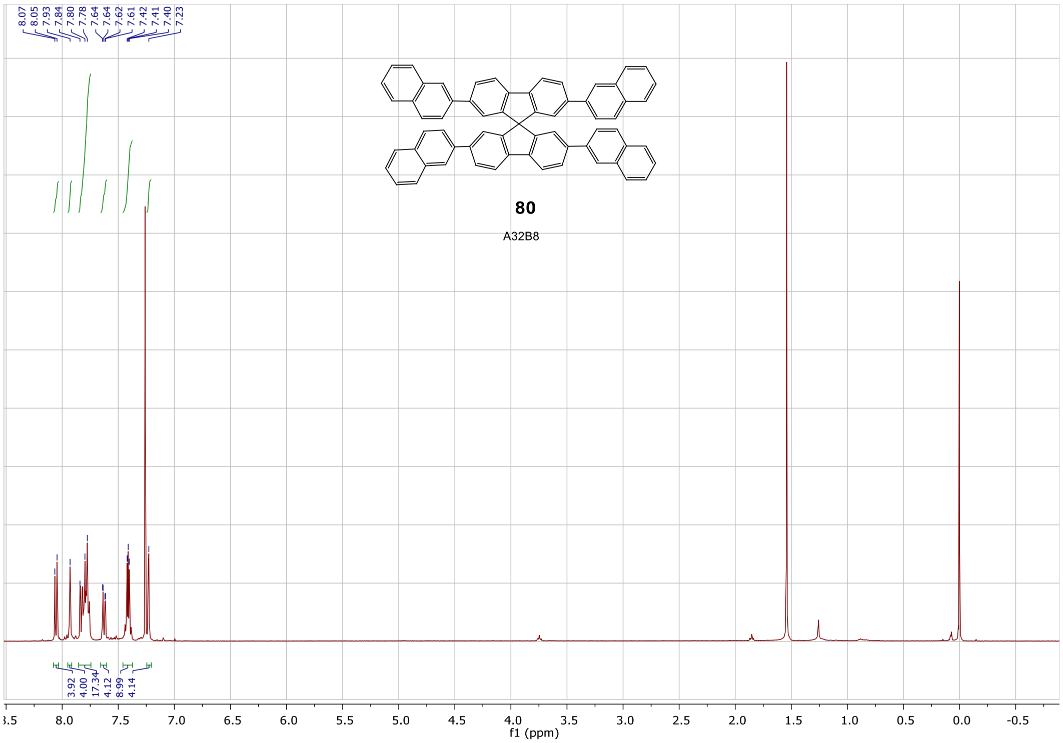

Supplementary Figure 124. ^{1}H NMR of **compound 80 (A32B8)** in $CDCl_3$.

^{1}H NMR (400 MHz, Chloroform-*d*) δ 8.06 (d, *J* = 8.0 Hz, 4H), 7.93 (s, 4H), 7.85 – 7.74 (m, 17H), 7.66 – 7.60 (m, 4H), 7.46 – 7.37 (m, 9H), 7.23 (s, 4H).

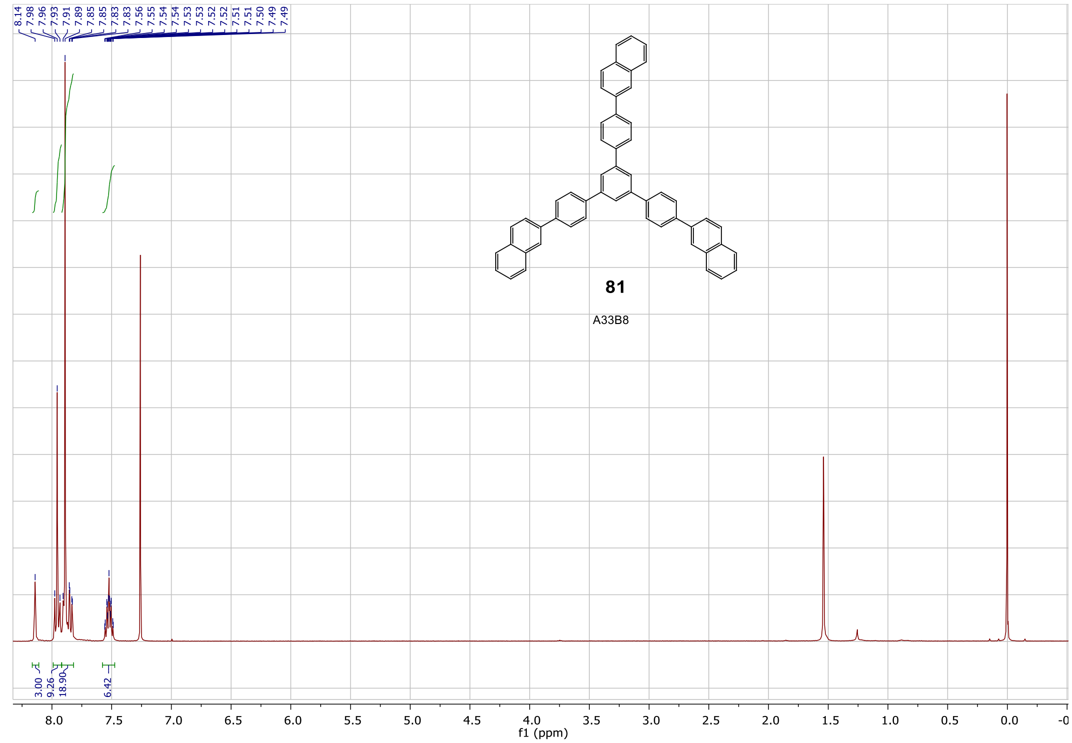

Supplementary Figure 125. ^{1}H NMR of **compound 81 (A33B8)** in $CDCl_3$.

^{1}H NMR (400 MHz, Chloroform-*d*) δ 8.14 (s, 3H), 7.99 – 7.92 (m, 9H), 7.92 – 7.82 (m, 19H), 7.58 – 7.47 (m, 6H).

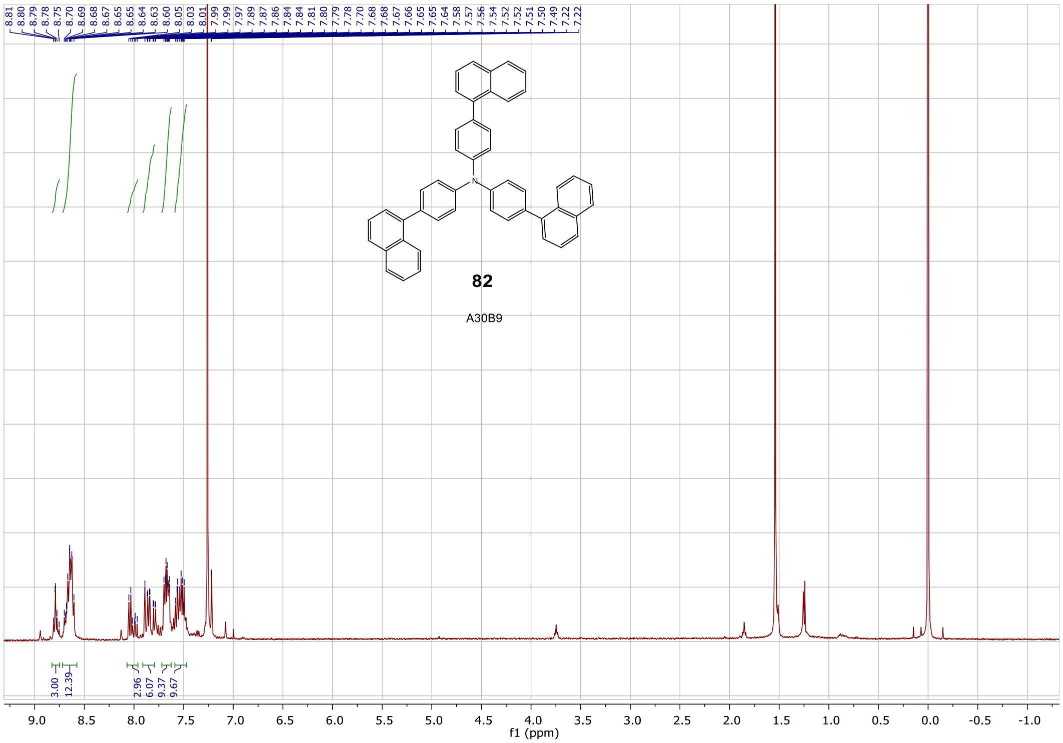

Supplementary Figure 126. ^{1}H NMR of **compound 82 (A30B9)** in $CDCl_3$.

^{1}H NMR (400 MHz, Chloroform-*d*) δ 8.83 – 8.75 (m, 3H), 8.72 – 8.57 (m, 12H), 8.07 – 7.96 (m, 3H), 7.91 – 7.79 (m, 6H), 7.72 – 7.62 (m, 9H), 7.59 – 7.47 (m, 10H).

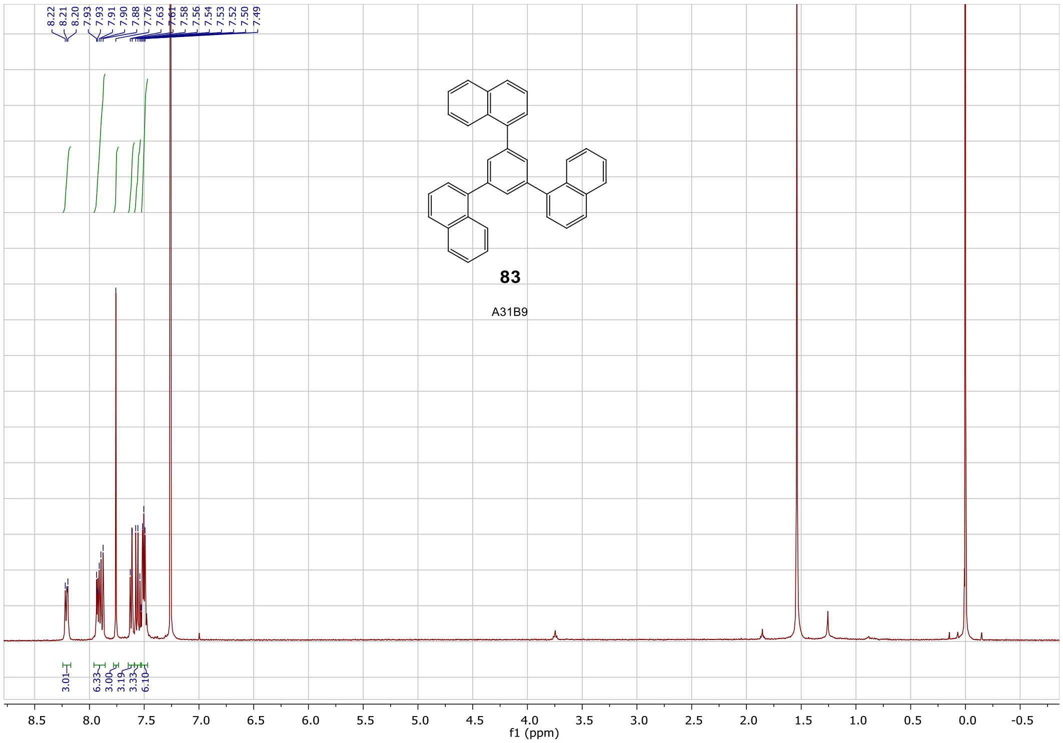

Supplementary Figure 127. ^{1}H NMR of **compound 83 (A31B9)** in $CDCl_3$.

^{1}H NMR (400 MHz, Chloroform-*d*) δ 8.24 – 8.17 (m, 3H), 7.96 – 7.86 (m, 6H), 7.76 (s, 3H), 7.62 (d, *J* = 7.1 Hz, 3H), 7.59 – 7.53 (m, 3H), 7.52 – 7.47 (m, 6H).

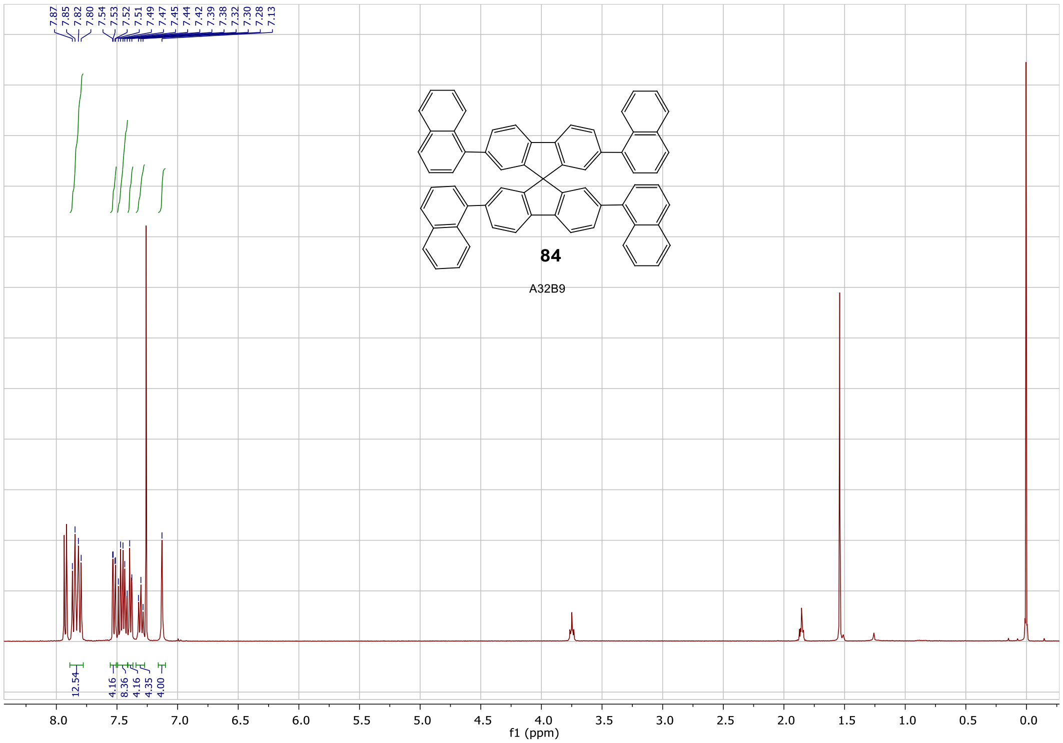

Supplementary Figure 128. ^{1}H NMR of **compound 84 (A32B9)** in $CDCl_3$.

^{1}H NMR (400 MHz, Chloroform-*d*) δ 7.83 (dd, *J* = 20.1, 9.0 Hz, 13H), 7.56 – 7.51 (m, 4H), 7.49 – 7.41 (m, 8H), 7.39 (d, *J* = 7.1 Hz, 4H), 7.30 (t, *J* = 7.6 Hz, 4H), 7.13 (s, 4H).

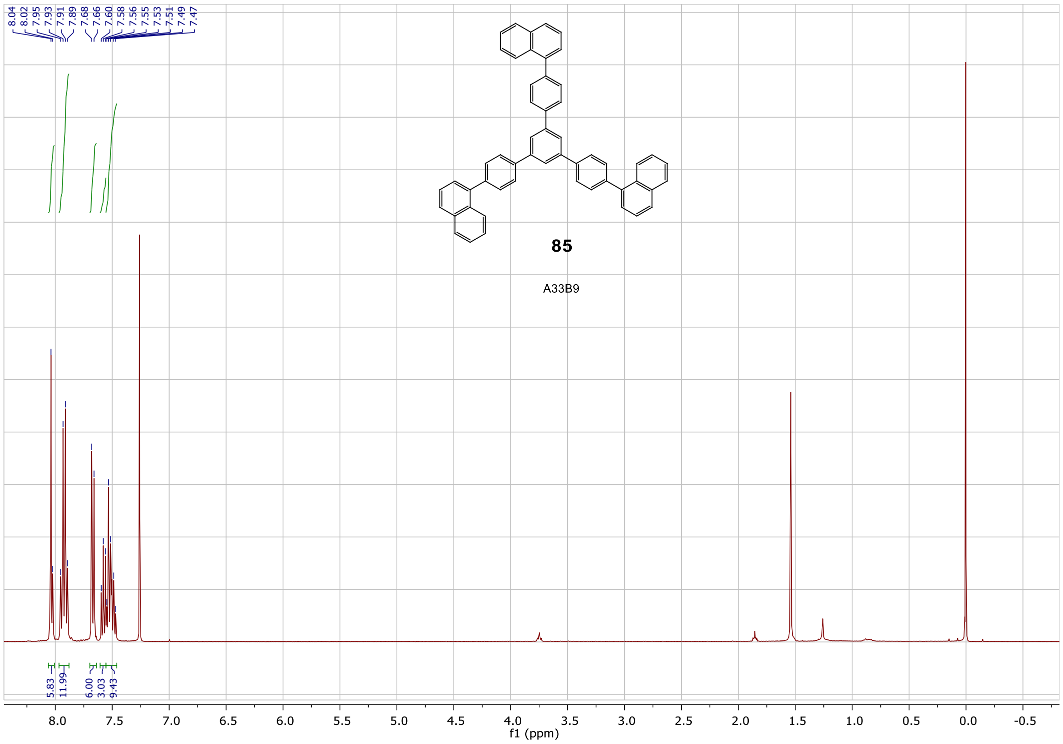

Supplementary Figure 129. ^{1}H NMR of **compound 85 (A33B9)** in $CDCl_3$.

^{1}H NMR (400 MHz, Chloroform-*d*) δ 8.03 (d, *J* = 5.8 Hz, 6H), 7.92 (q, *J* = 7.7, 7.0 Hz, 12H), 7.67 (d, *J* = 8.2 Hz, 6H), 7.61 – 7.56 (m, 3H), 7.56 – 7.46 (m, 9H).

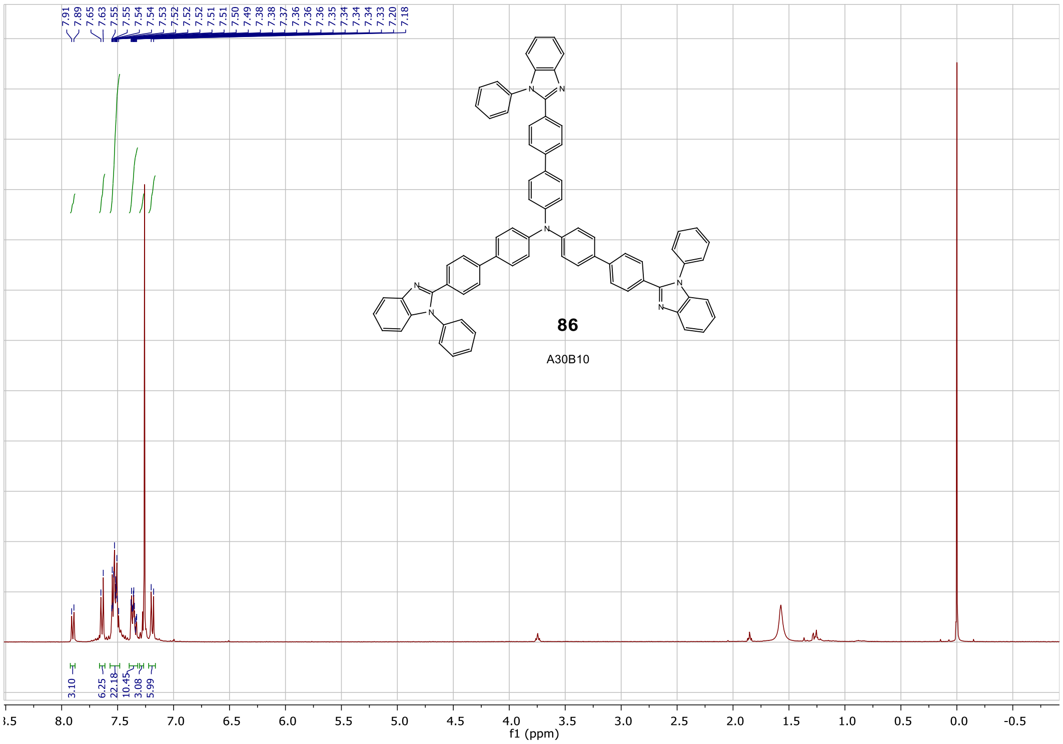

Supplementary Figure 130. ^{1}H NMR of **compound 86 (A30B10)** in $CDCl_3$.

^{1}H NMR (400 MHz, Chloroform-*d*) δ 7.90 (d, *J* = 8.0 Hz, 3H), 7.64 (d, *J* = 8.5 Hz, 6H), 7.57 – 7.48 (m, 22H), 7.40 – 7.32 (m, 10H), 7.30 – 7.27 (m, 3H), 7.19 (d, *J* = 8.7 Hz, 6H).

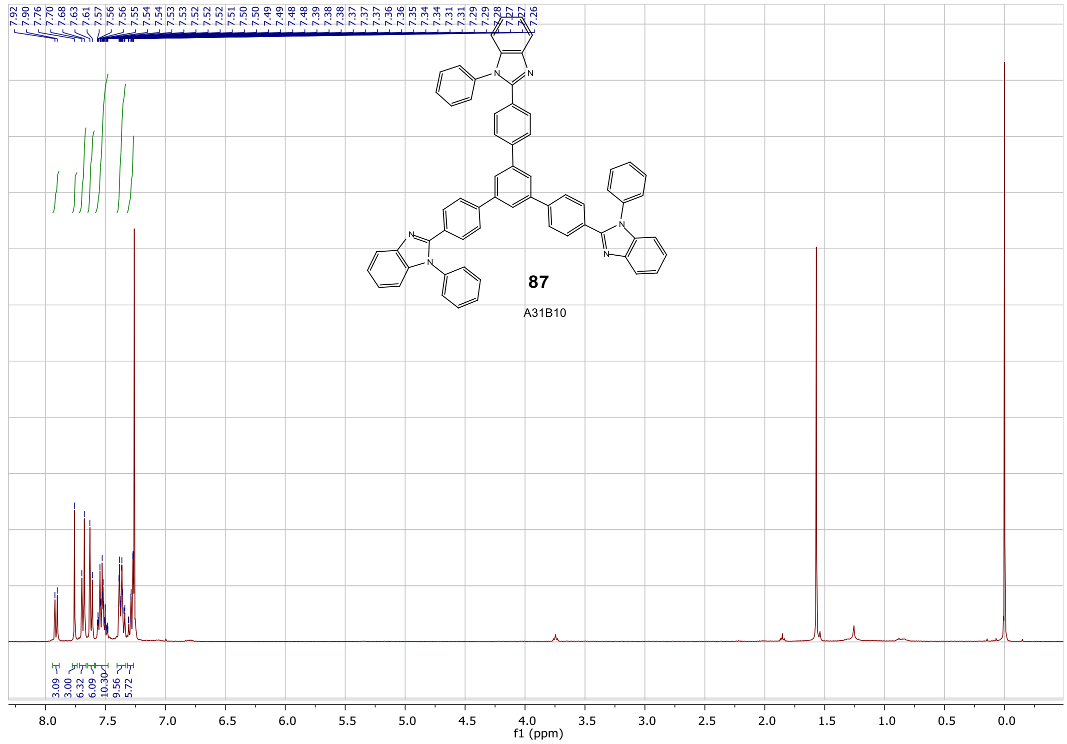

Supplementary Figure 131. ^{1}H NMR of **compound 87 (A31B10)** in $CDCl_3$.

^{1}H NMR (400 MHz, Chloroform-*d*) δ 7.91 (d, *J* = 8.0 Hz, 3H), 7.76 (s, 3H), 7.69 (d, *J* = 8.5 Hz, 6H), 7.62 (d, *J* = 8.5 Hz, 6H), 7.58 – 7.48 (m, 10H), 7.40 – 7.33 (m, 10H), 7.32 – 7.27 (m, 6H).

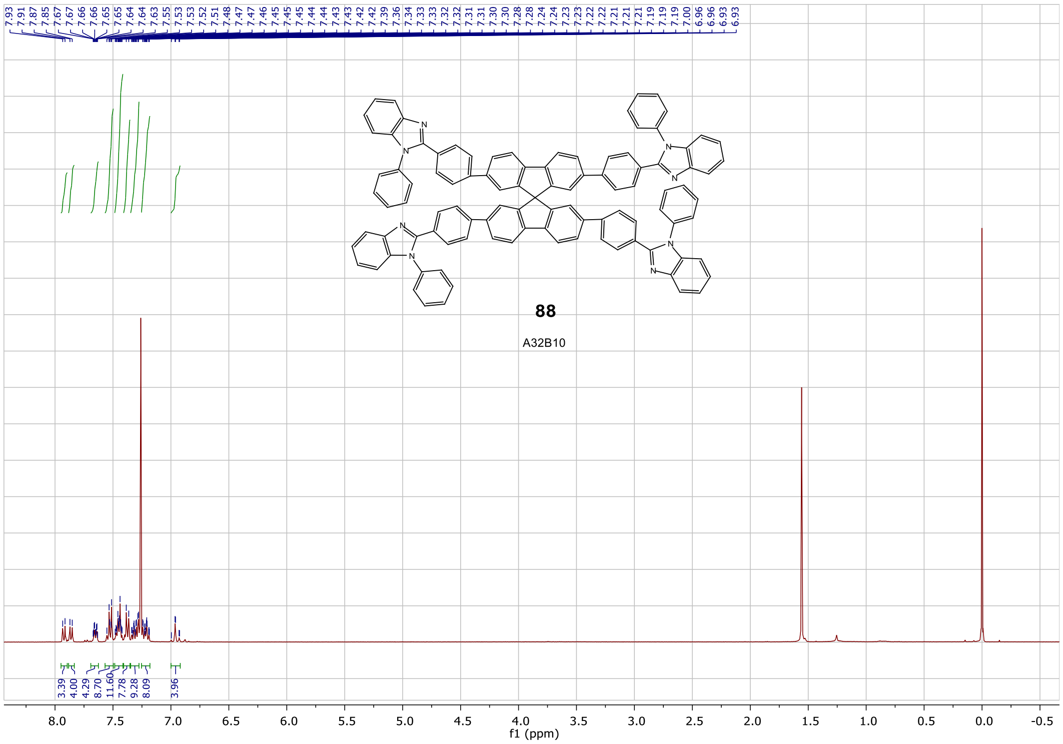

Supplementary Figure 132. ^{1}H NMR of **compound 88 (A32B10)** in $CDCl_3$.

^{1}H NMR (400 MHz, Chloroform-*d*) δ 7.92 (d, *J* = 8.0 Hz, 3H), 7.86 (d, *J* = 8.0 Hz, 4H), 7.69 – 7.63 (m, 4H), 7.57 – 7.50 (m, 9H), 7.48 – 7.41 (m, 12H), 7.37 (d, *J* = 8.6 Hz, 8H), 7.35 – 7.28 (m, 9H), 7.25 – 7.18 (m, 8H), 7.00 – 6.92 (m, 4H).

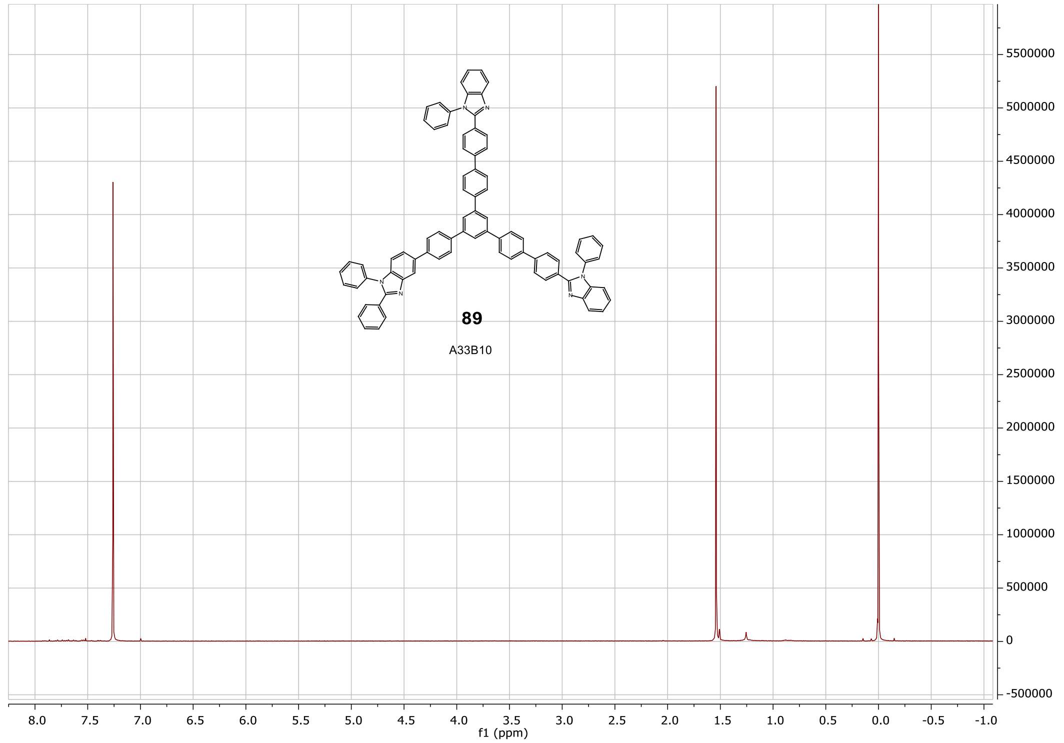

Supplementary Figure 133. ^{1}H NMR of **compound 89 (A33B10)** in $CDCl_3$.

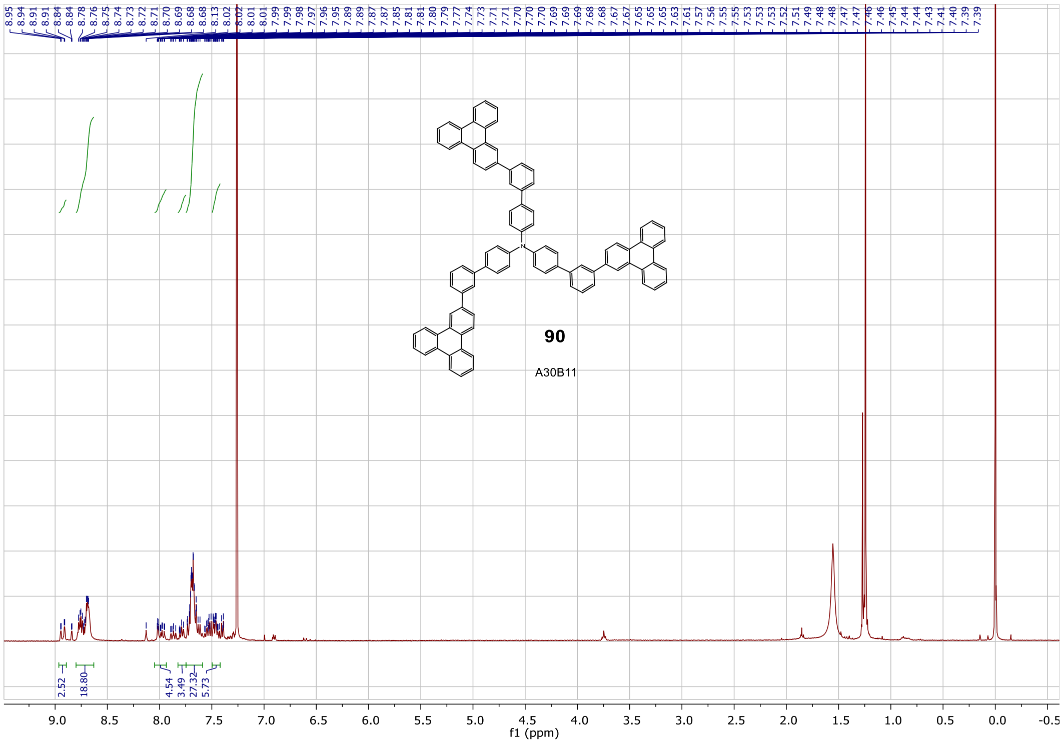

Supplementary Figure 134. ^{1}H NMR of **compound 90 (A30B11)** in $CDCl_3$.

^{1}H NMR (400 MHz, Chloroform-*d*) δ 8.93 (d, *J* = 13.7, 1.9 Hz, 3H), 8.80 – 8.63 (m, 19H), 8.05 – 7.94 (m, 5H), 7.82 – 7.75 (m, 3H), 7.75 – 7.59 (m, 27H), 7.50 – 7.42 (m, 6H).

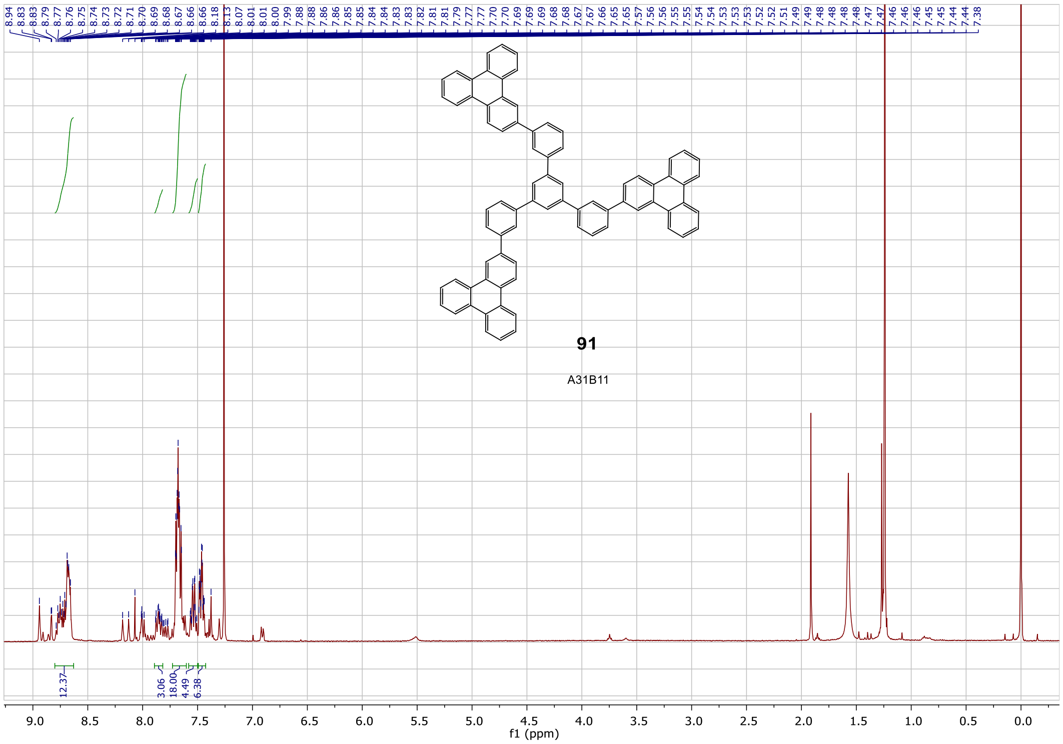

Supplementary Figure 135. ^{1}H NMR of **compound 91 (A31B11)** in $CDCl_3$.

^{1}H NMR (400 MHz, Chloroform-*d*) δ 8.80 – 8.63 (m, 12H), 7.89 – 7.82 (m, 3H), 7.73 – 7.60 (m, 18H), 7.58 – 7.50 (m, 4H), 7.49 – 7.43 (m, 6H).

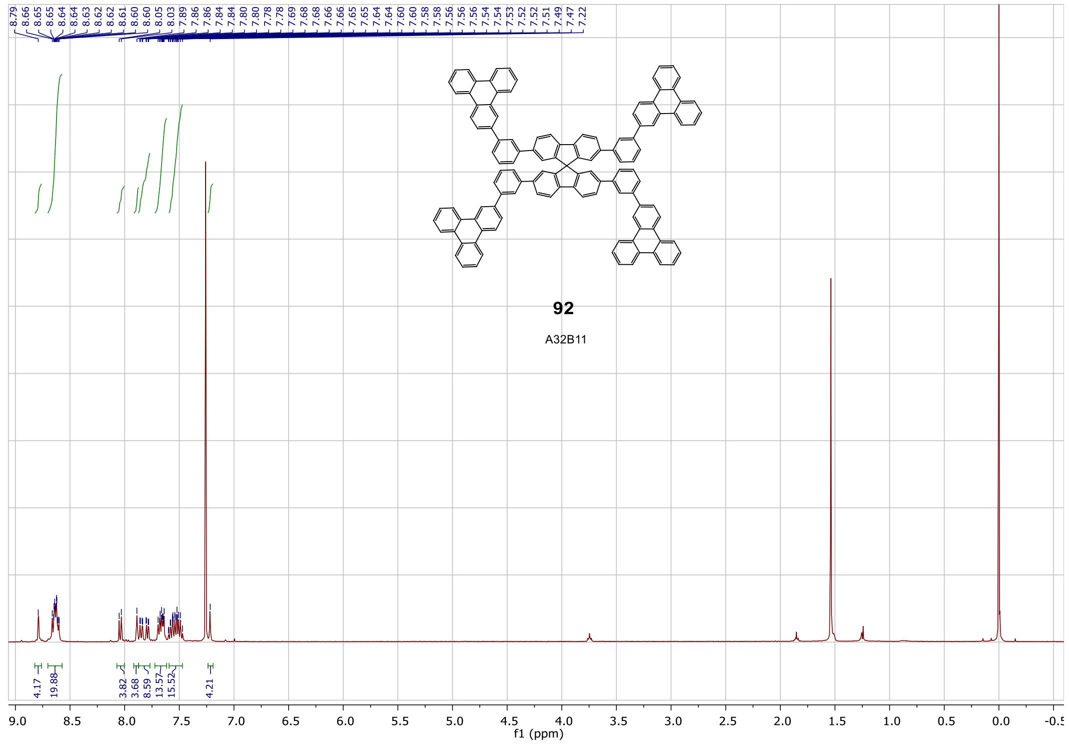

Supplementary Figure 136. ^{1}H NMR of **compound 92 (A32B11)** in $CDCl_3$.

^{1}H NMR (400 MHz, Chloroform-*d*) δ 8.79 (s, 4H), 8.70 – 8.57 (m, 21H), 8.04 (d, *J* = 8.0 Hz, 4H), 7.89 (s, 4H), 7.87 – 7.77 (m, 9H), 7.72 – 7.62 (m, 14H), 7.59 – 7.47 (m, 16H), 7.22 (s, 4H).

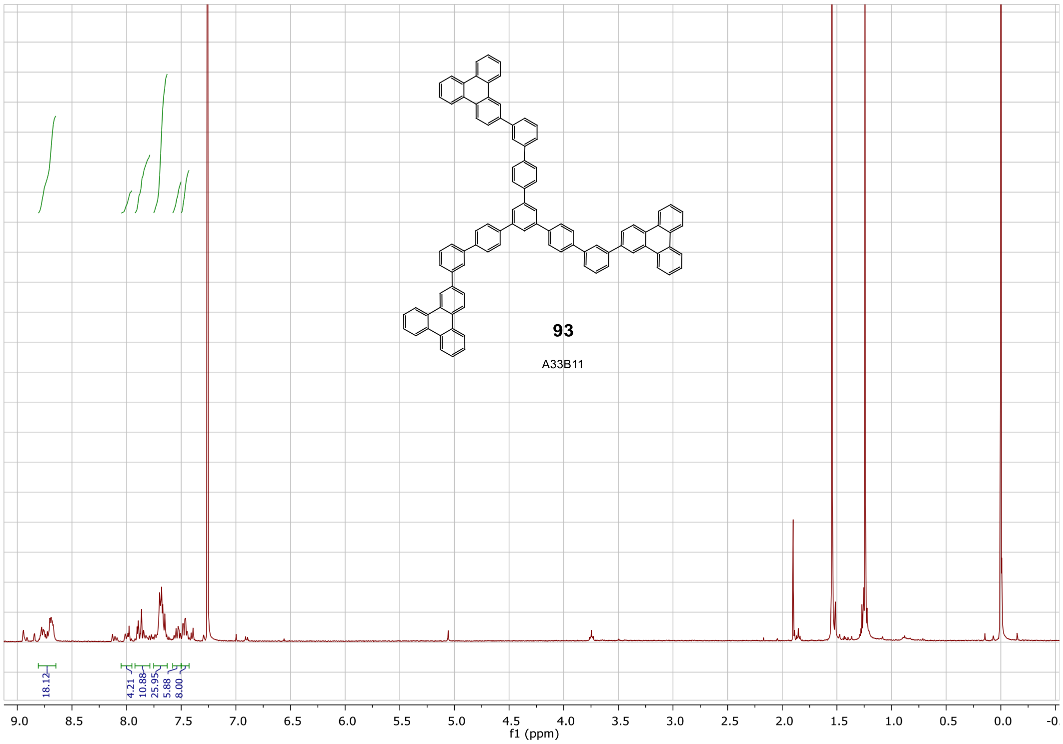

Supplementary Figure 137. ^{1}H NMR of **compound 93 (A33B11)** in $CDCl_3$.

^{1}H NMR (400 MHz, Chloroform-*d*) δ 8.81 – 8.65 (m, 18H), 8.05 – 7.95 (m, 4H), 7.92 – 7.79 (m, 11H), 7.75 – 7.63 (m, 26H), 7.58 – 7.50 (m, 6H), 7.50 – 7.43 (m, 8H).

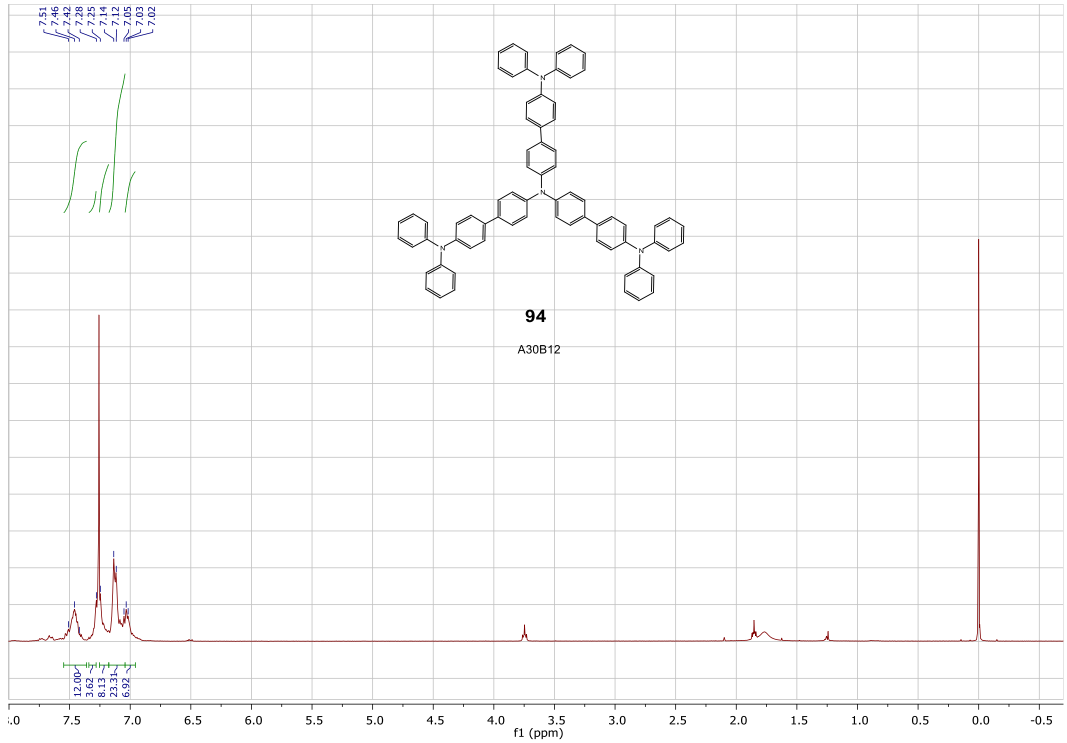

Supplementary Figure 138. ^{1}H NMR of **compound 94 (A30B12)** in $CDCl_3$.

^{1}H NMR (400 MHz, Chloroform-*d*) δ 7.55 – 7.36 (m, 12H), 7.34 – 7.28 (m, 4H), 7.25 – 7.18 (m, 8H), 7.18 – 7.04 (m, 23H), 7.04 – 6.96 (m, 7H).

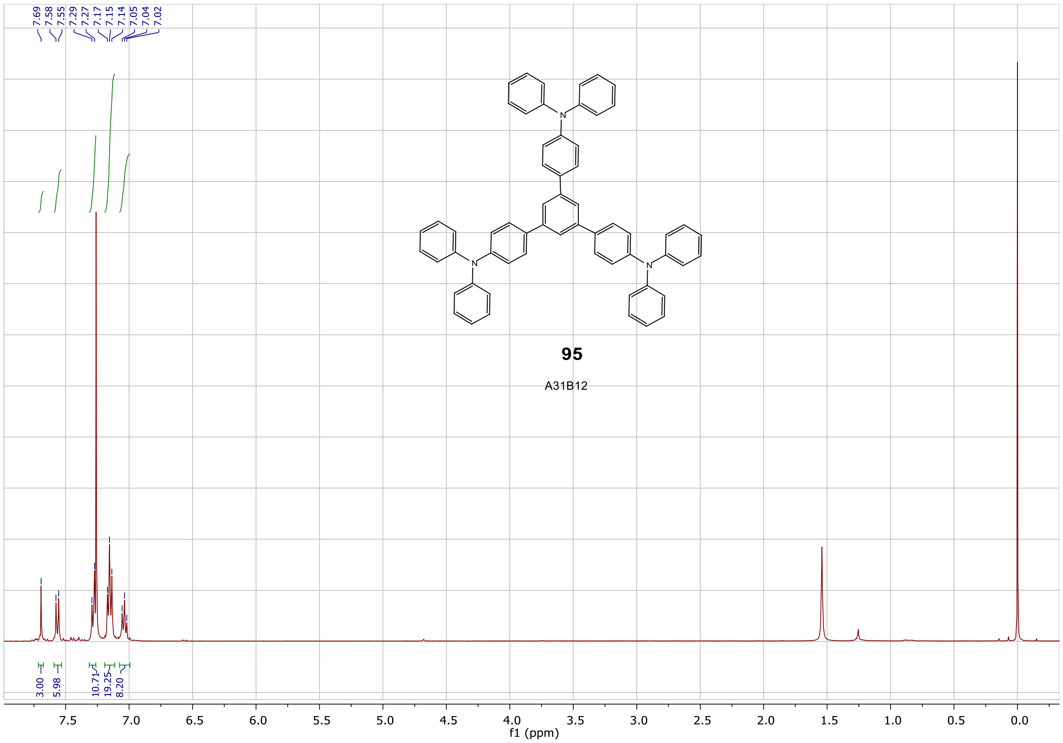

Supplementary Figure 139. ^{1}H NMR of **compound 95 (A31B12)** in $CDCl_3$.

^{1}H NMR (400 MHz, Chloroform-*d*) δ 7.69 (s, 3H), 7.57 (d, *J* = 8.6 Hz, 6H), 7.31 – 7.26 (m, 11H), 7.15 (t, *J* = 6.9 Hz, 19H), 7.04 (t, *J* = 7.3 Hz, 8H).

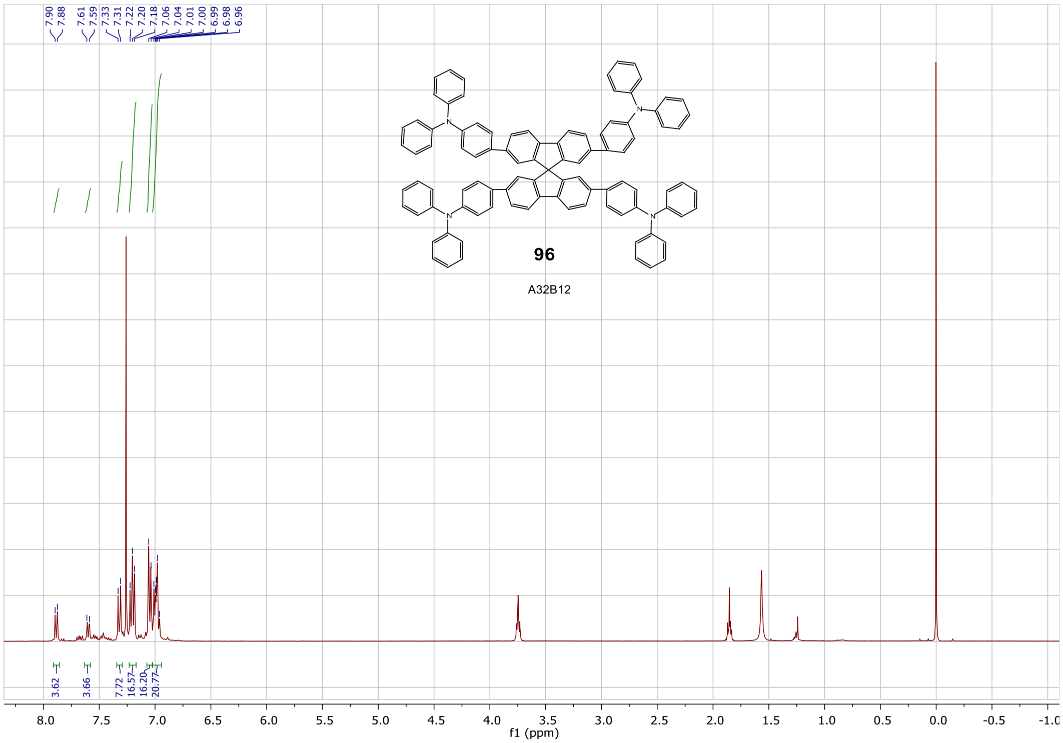

Supplementary Figure 140. ^{1}H NMR of **compound 96 (A32B12)** in $CDCl_3$.

^{1}H NMR (400 MHz, Chloroform-*d*) δ 7.89 (d, *J* = 7.9 Hz, 4H), 7.60 (d, *J* = 9.6 Hz, 4H), 7.32 (d, *J* = 8.7 Hz, 8H), 7.23 – 7.17 (m, 17H), 7.05 (d, *J* = 8.4 Hz, 16H), 7.02 – 6.94 (m, 21H).

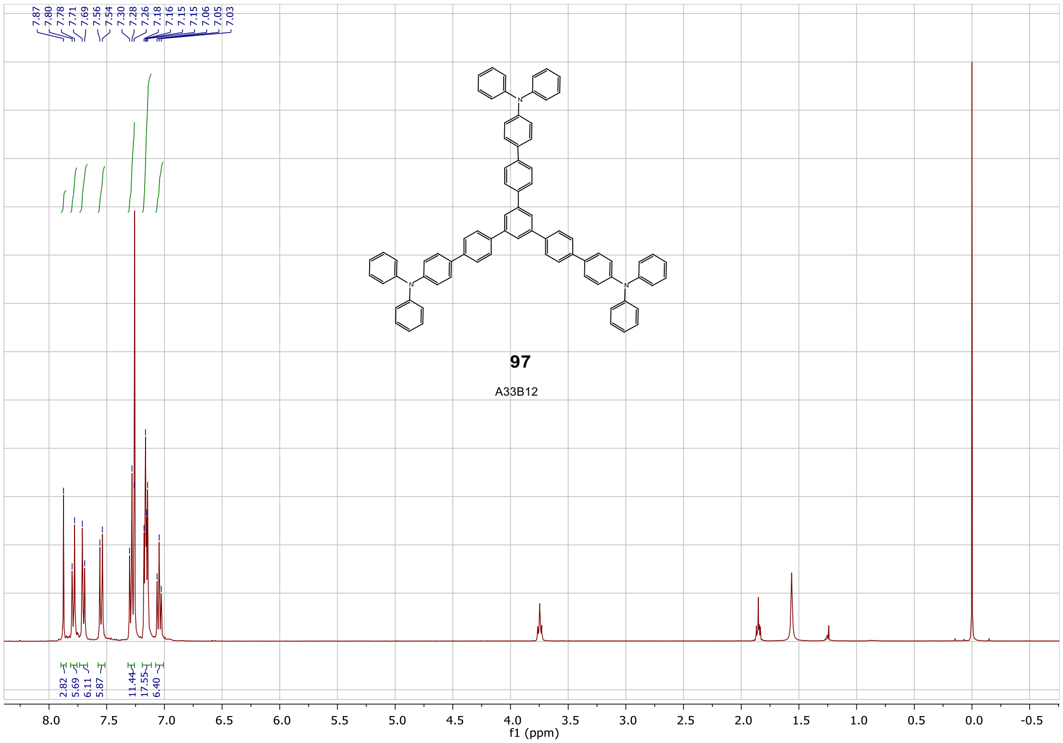

Supplementary Figure 141. ^{1}H NMR of **compound 97 (A33B12)** in $CDCl_3$.

^{1}H NMR (400 MHz, Chloroform-*d*) δ 7.87 (s, 3H), 7.79 (d, *J* = 8.4 Hz, 6H), 7.70 (d, *J* = 8.4 Hz, 6H), 7.55 (d, *J* = 8.7 Hz, 6H), 7.32 – 7.26 (m, 11H), 7.19 – 7.11 (m, 18H), 7.05 (t, *J* = 7.3 Hz, 6H).

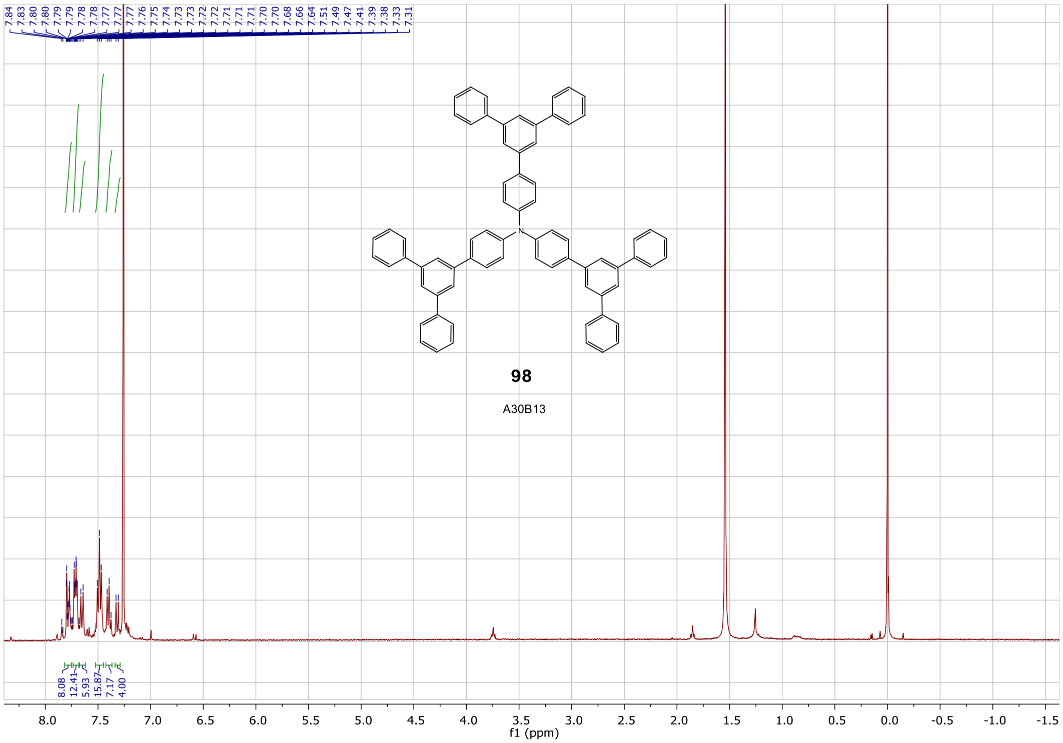

Supplementary Figure 142. ^{1}H NMR of **compound 98 (A30B13)** in $CDCl_3$.

^{1}H NMR (400 MHz, Chloroform-*d*) δ 7.82 – 7.75 (m, 8H), 7.74 – 7.68 (m, 12H), 7.65 (d, *J* = 8.6 Hz, 6H), 7.49 (t, *J* = 7.6 Hz, 16H), 7.39 (t, *J* = 7.3 Hz, 7H), 7.32 (d, *J* = 8.7 Hz, 4H).

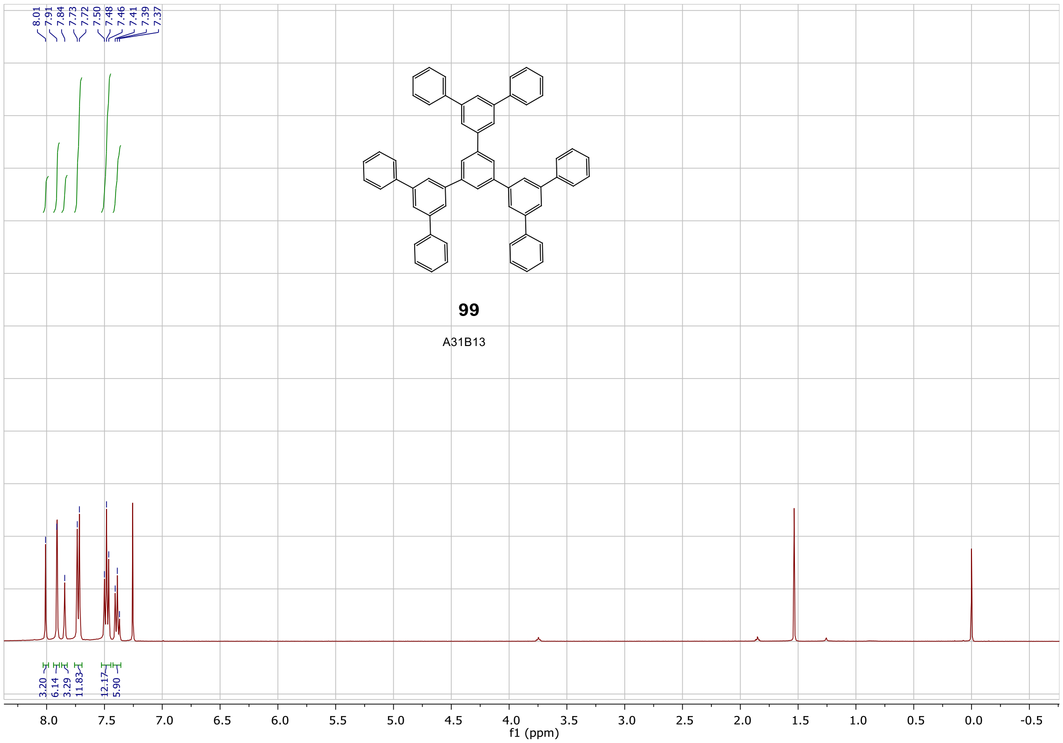

Supplementary Figure 143. ^{1}H NMR of **compound 99 (A31B13)** in $CDCl_3$.

^{1}H NMR (400 MHz, Chloroform-*d*) δ 8.01 (s, 3H), 7.91 (s, 6H), 7.84 (s, 3H), 7.73 (d, *J* = 7.2 Hz, 12H), 7.48 (t, *J* = 7.6 Hz, 12H), 7.39 (t, *J* = 7.4 Hz, 6H).

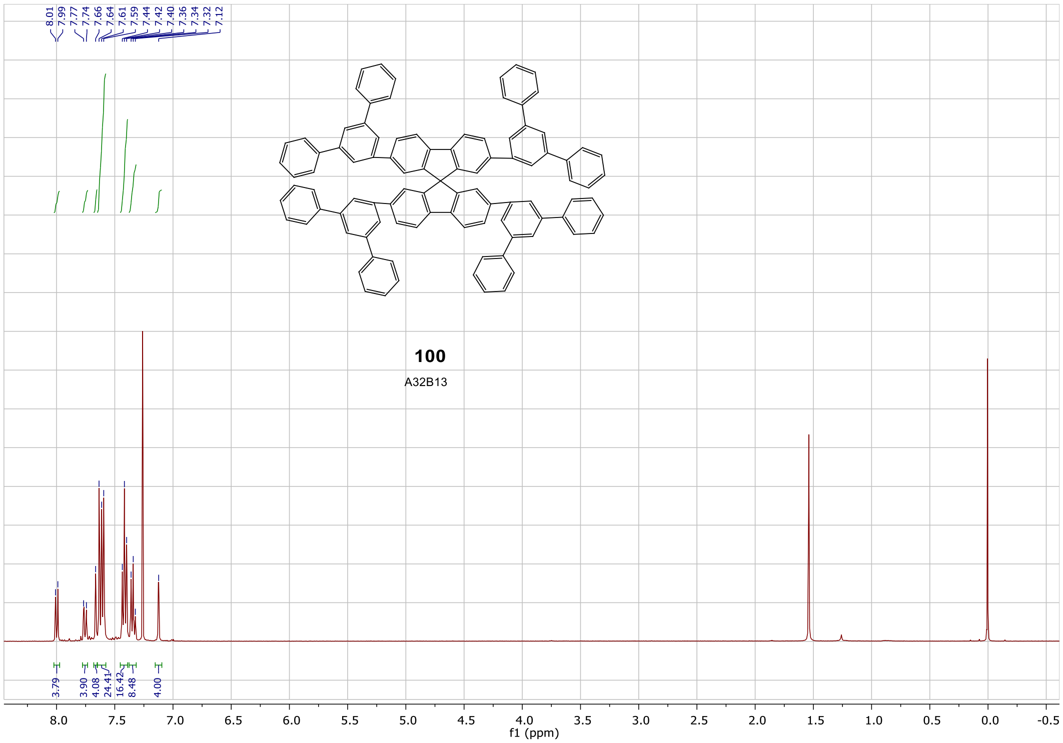

Supplementary Figure 144. ^{1}H NMR of **compound 100 (A32B13)** in $CDCl_3$.

^{1}H NMR (400 MHz, Chloroform-*d*) δ 8.00 (d, *J* = 7.9 Hz, 4H), 7.76 (d, *J* = 9.6 Hz, 4H), 7.66 (s, 4H), 7.65 – 7.58 (m, 24H), 7.42 (t, *J* = 7.4 Hz, 16H), 7.34 (t, *J* = 7.3 Hz, 8H), 7.12 (s, 4H).

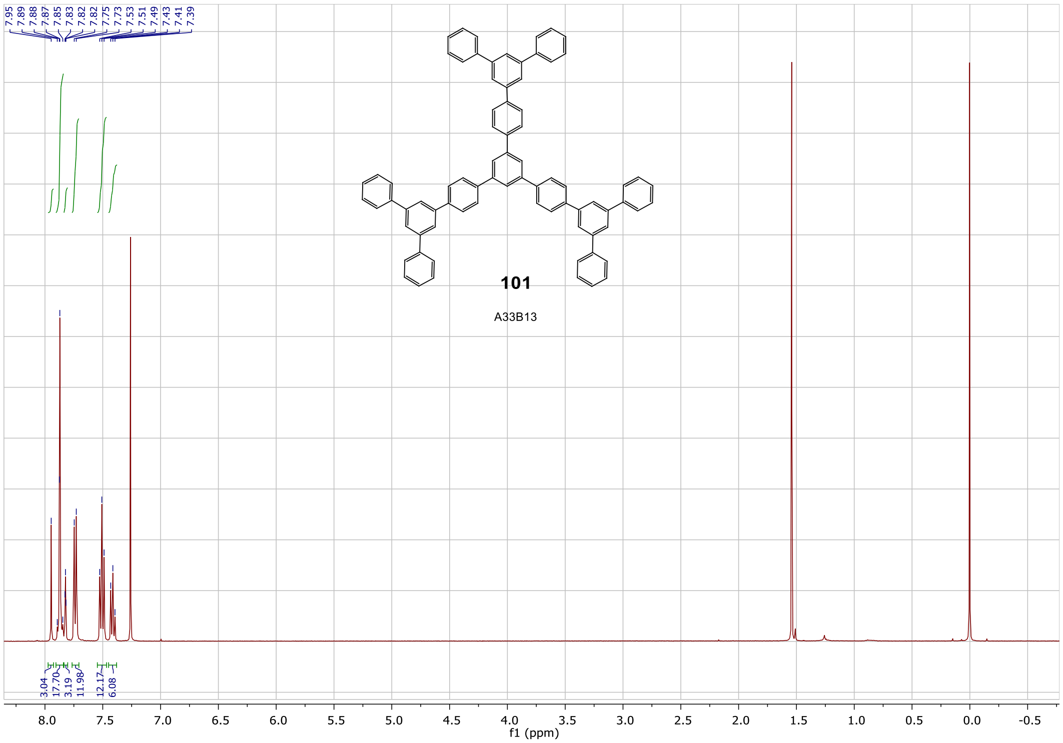

Supplementary Figure 145. ^{1}H NMR of **compound 101 (A33B13)** in $CDCl_3$.

^{1}H NMR (400 MHz, Chloroform-*d*) δ 7.95 (s, 3H), 7.91 – 7.84 (m, 18H), 7.84 – 7.80 (m, 3H), 7.74 (d, *J* = 7.1 Hz, 12H), 7.51 (t, *J* = 7.6 Hz, 12H), 7.41 (t, *J* = 7.4 Hz, 6H).

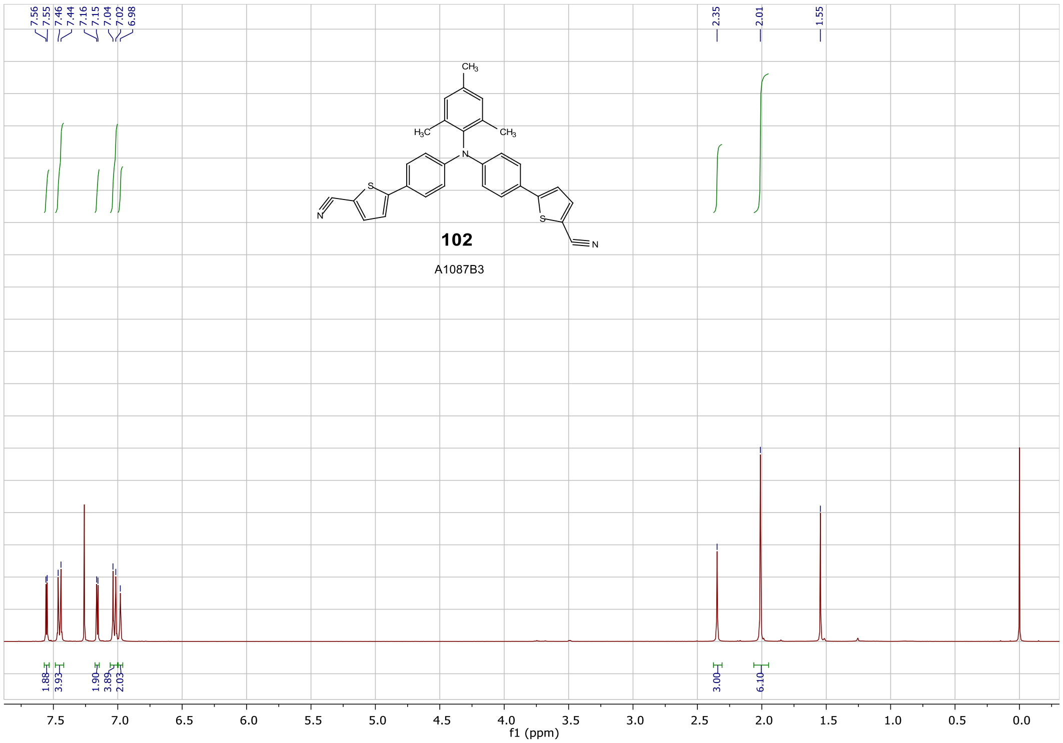

Supplementary Figure 146. ^{1}H NMR of **compound 102 (A1087B3)** in $CDCl_3$.

^{1}H NMR (400 MHz, Chloroform-*d*) δ 7.55 (d, *J* = 4.0 Hz, 2H), 7.45 (d, *J* = 8.8 Hz, 4H), 7.16 (d, *J* = 4.0 Hz, 2H), 7.03 (d, *J* = 8.8 Hz, 4H), 6.98 (s, 2H), 2.35 (s, 3H), 2.01 (s, 6H).

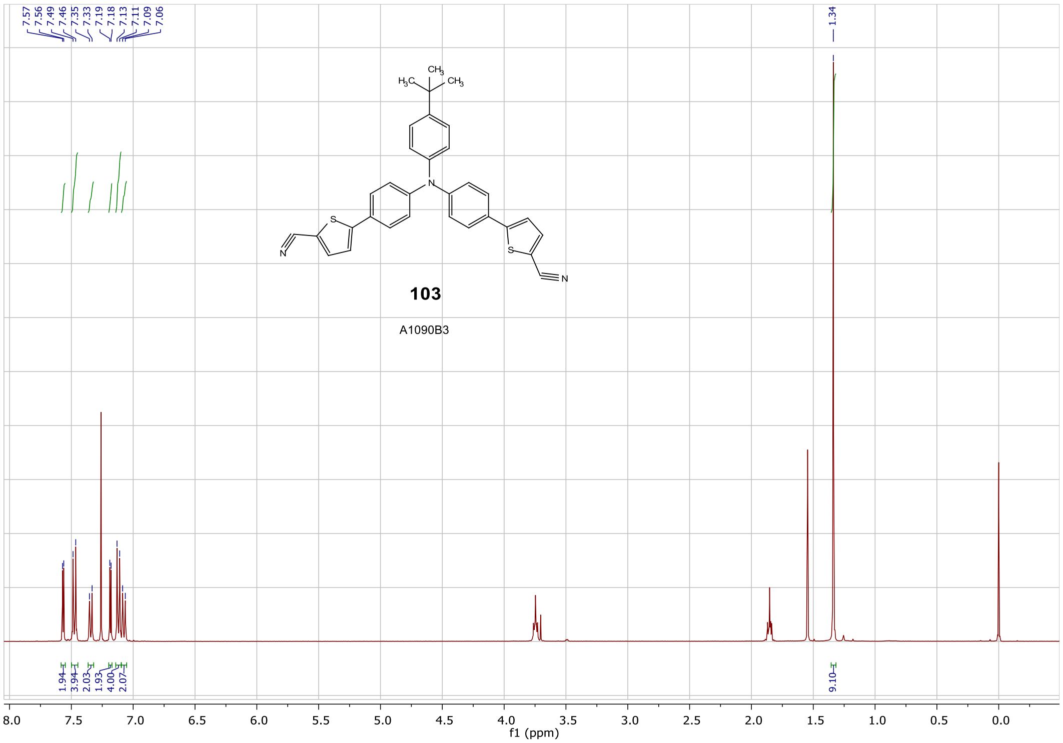

Supplementary Figure 147. ^{1}H NMR of **compound 103 (A1090B3)** in $CDCl_3$.

^{1}H NMR (400 MHz, Chloroform-*d*) δ 7.57 (d, *J* = 3.9 Hz, 2H), 7.48 (d, *J* = 8.7 Hz, 4H), 7.34 (d, *J* = 8.7 Hz, 2H), 7.18 (d, *J* = 3.9 Hz, 2H), 7.12 (d, *J* = 8.7 Hz, 4H), 7.07 (d, *J* = 8.7 Hz, 2H), 1.34 (s, 9H).

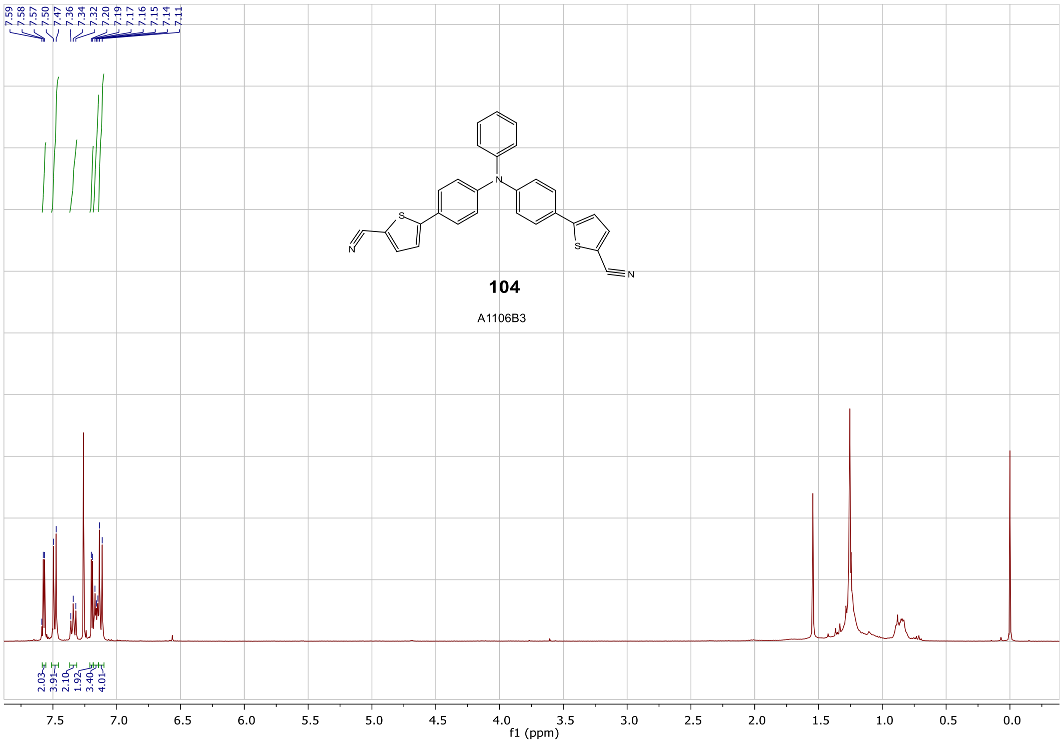

Supplementary Figure 148. ^{1}H NMR of **compound 104 (A11060B3)** in $CDCl_3$.

^{1}H NMR (400 MHz, Chloroform-*d*) δ 7.57 (d, *J* = 4.0 Hz, 2H), 7.48 (d, *J* = 8.7 Hz, 4H), 7.37 – 7.31 (m, 2H), 7.19 (d, *J* = 3.9 Hz, 2H), 7.18 – 7.14 (m, 3H), 7.12 (d, *J* = 8.7 Hz, 4H).

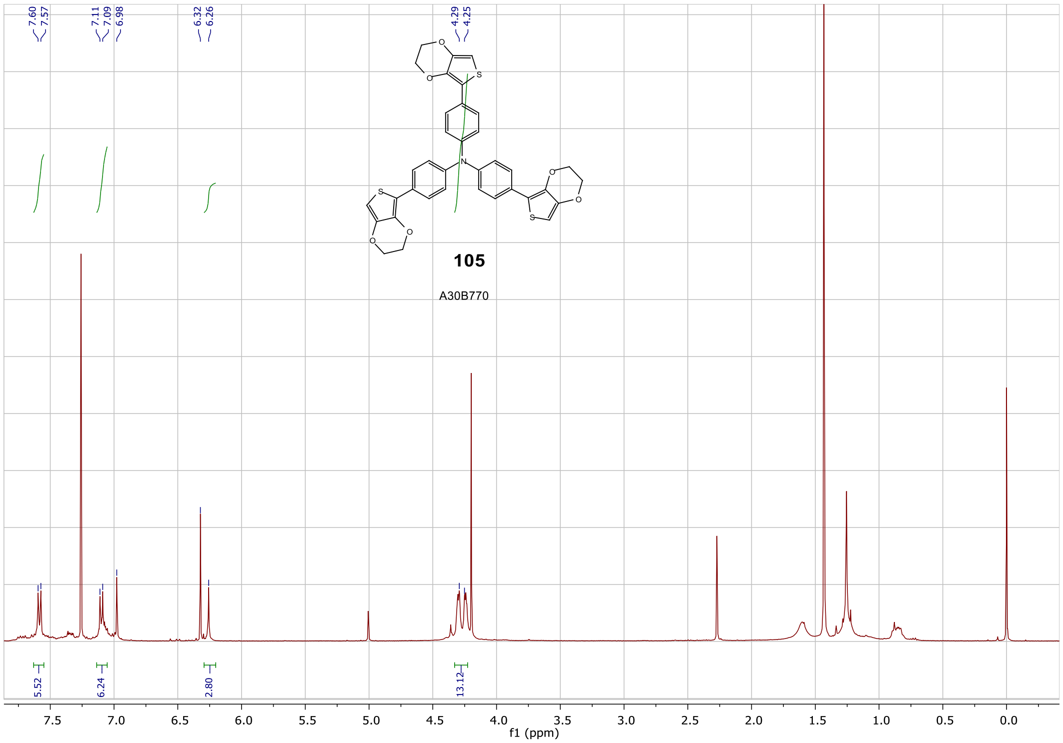

Supplementary Figure 149. ^{1}H NMR of **compound 105 (A30B770)** in $CDCl_3$.

^{1}H NMR (400 MHz, Chloroform-*d*) δ 7.58 (d, *J* = 8.8 Hz, 6H), 7.10 (d, *J* = 8.8 Hz, 6H), 6.26 (s, 3H), 4.27 (d, *J* = 16.3 Hz, 13H).

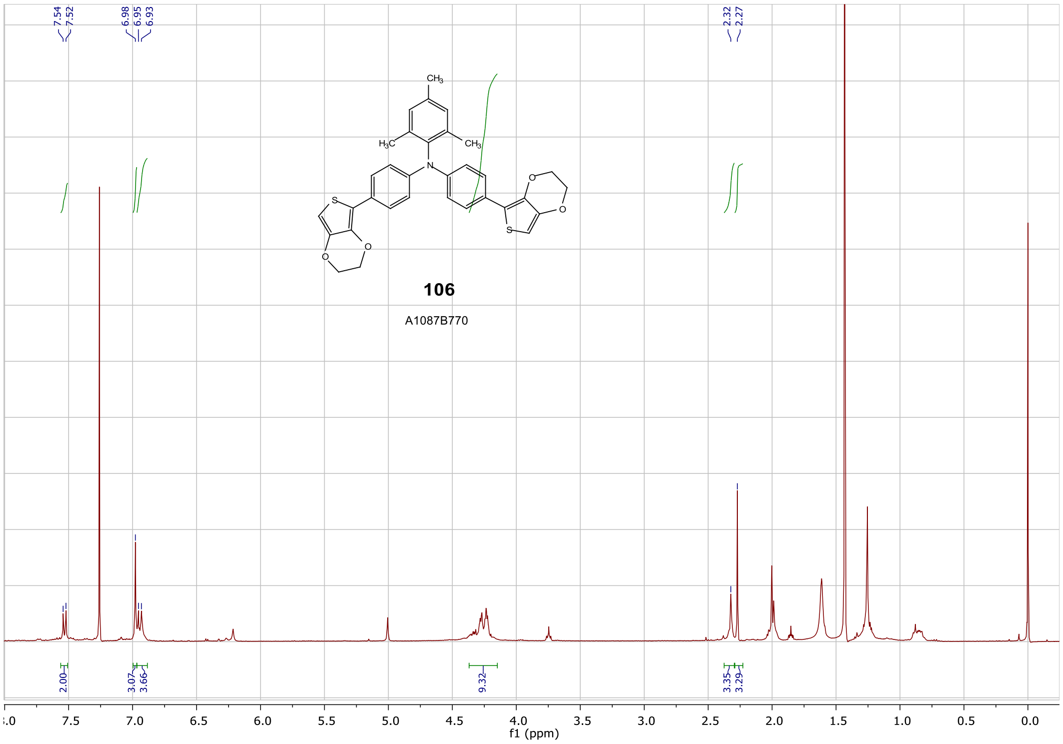

Supplementary Figure 150. ^{1}H NMR of **compound 106 (A1087B770)** in $CDCl_3$.

^{1}H NMR (400 MHz, Chloroform-*d*) δ 7.53 (d, *J* = 8.8 Hz, 2H), 6.98 (s, 4H), 6.94 (d, *J* = 9.3 Hz, 4H), 4.37 – 4.15 (m, 9H), 2.32 (s, 3H), 2.27 (s, 3H).

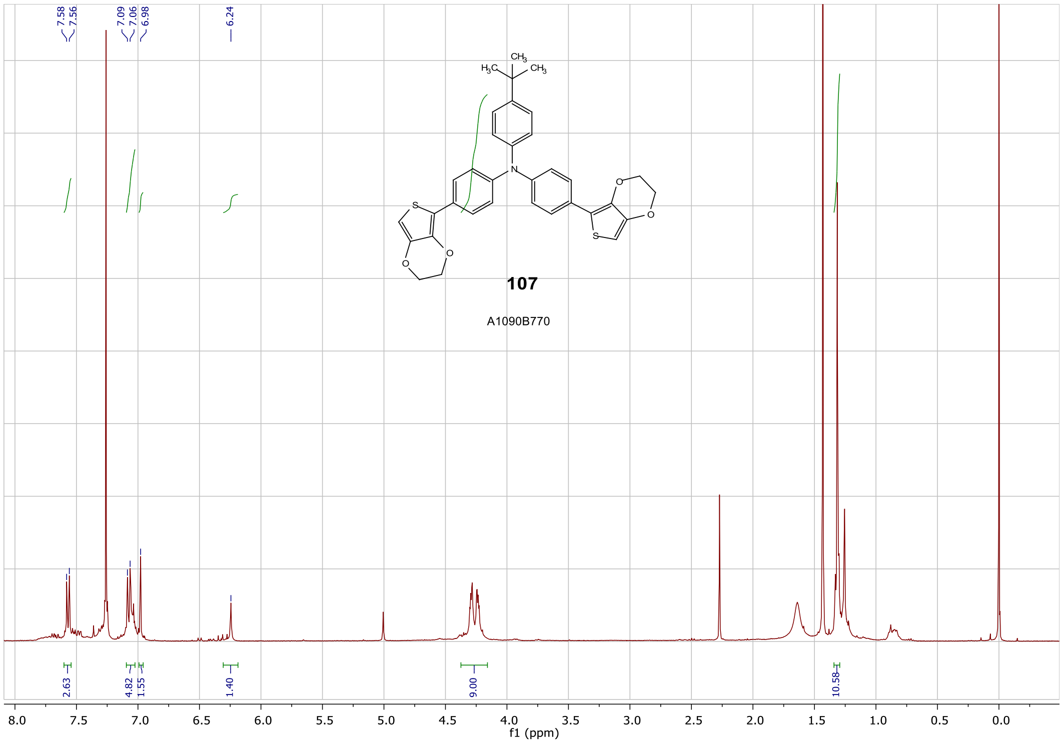

Supplementary Figure 151. ^{1}H NMR of **compound 107 (A1090B770)** in $CDCl_3$.

^{1}H NMR (400 MHz, Chloroform-*d*) δ 7.57 (d, *J* = 8.8 Hz, 3H), 7.07 (d, *J* = 8.8 Hz, 5H), 6.98 (s, 2H), 6.24 (s, 2H), 4.37 – 4.16 (m, 9H), 1.31 (d, *J* = 1.7 Hz, 11H).

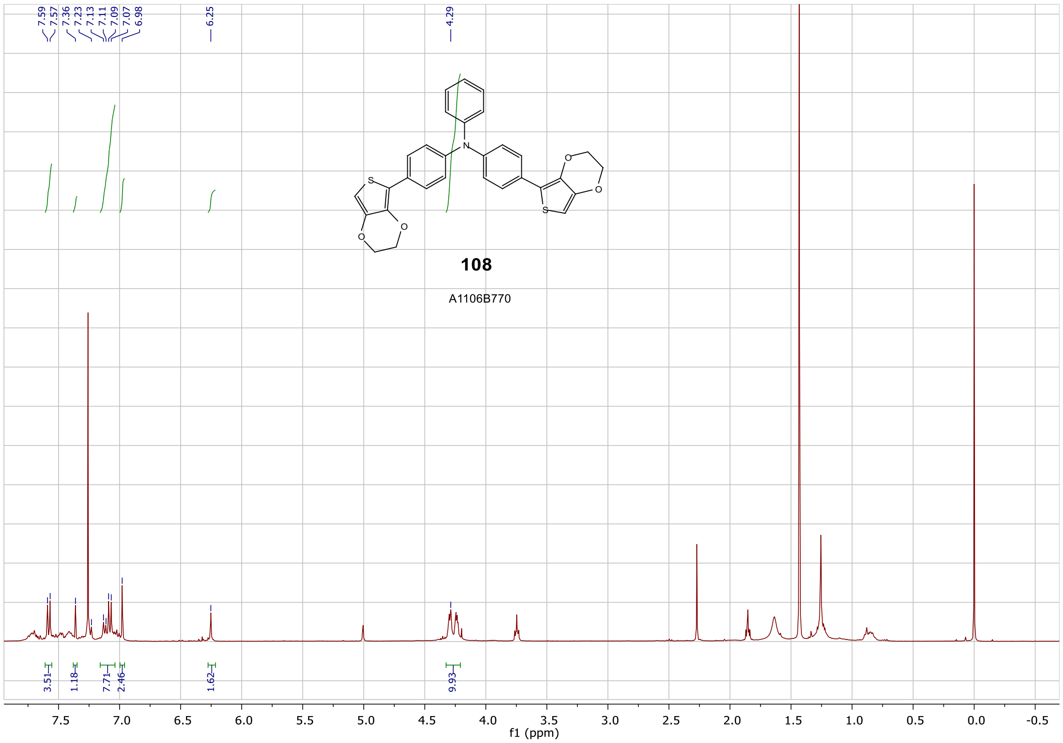

Supplementary Figure 152. ^{1}H NMR of **compound 108 (A1106B770)** in $CDCl_3$.

^{1}H NMR (400 MHz, Chloroform-*d*) δ 7.58 (d, *J* = 8.8 Hz, 4H), 7.36 (s, 1H), 7.16 – 7.04 (m, 8H), 6.98 (s, 2H), 6.25 (s, 2H), 4.33 – 4.21 (m, 10H).

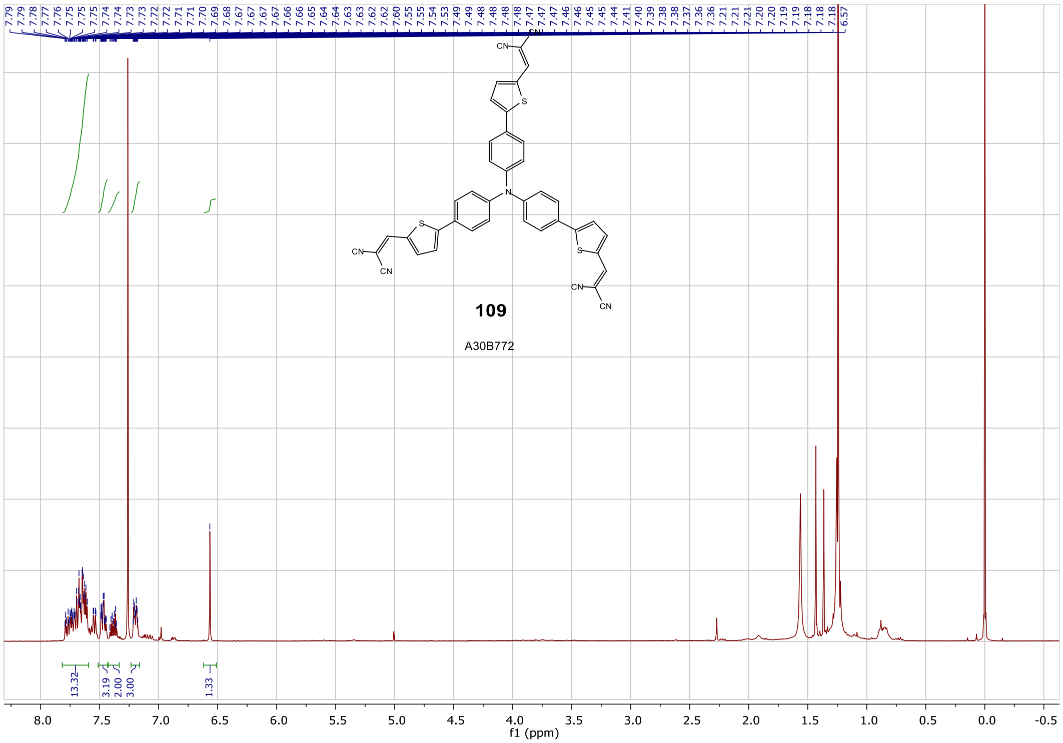

Supplementary Figure 153. ^1H NMR of **compound 109 (A30B772)** in $CDCl_3$.

^1H NMR (400 MHz, Chloroform-*d*) δ 7.82 – 7.59 (m, 13H), 7.51 – 7.43 (m, 3H), 7.43 – 7.33 (m, 2H), 7.23 – 7.16 (m, 3H), 6.57 (s, 1H).

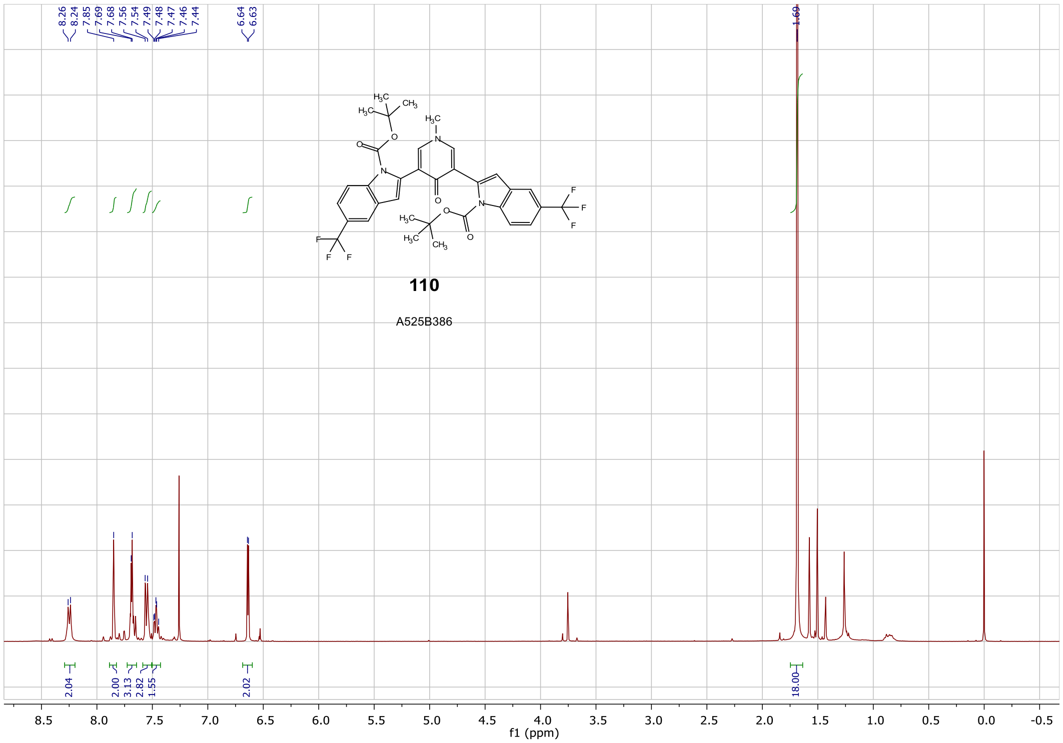

Supplementary Figure 154. ^{1}H NMR of **compound 110 (A525B386)** in $CDCl_3$.

^{1}H NMR (400 MHz, Chloroform-*d*) δ 8.25 (d, *J* = 8.6 Hz, 2H), 7.85 (s, 2H), 7.69 (d, *J* = 3.7 Hz, 3H), 7.55 (d, *J* = 8.9 Hz, 3H), 7.50 – 7.43 (m, 2H), 6.64 (d, *J* = 3.7 Hz, 2H), 1.69 (s, 18H).

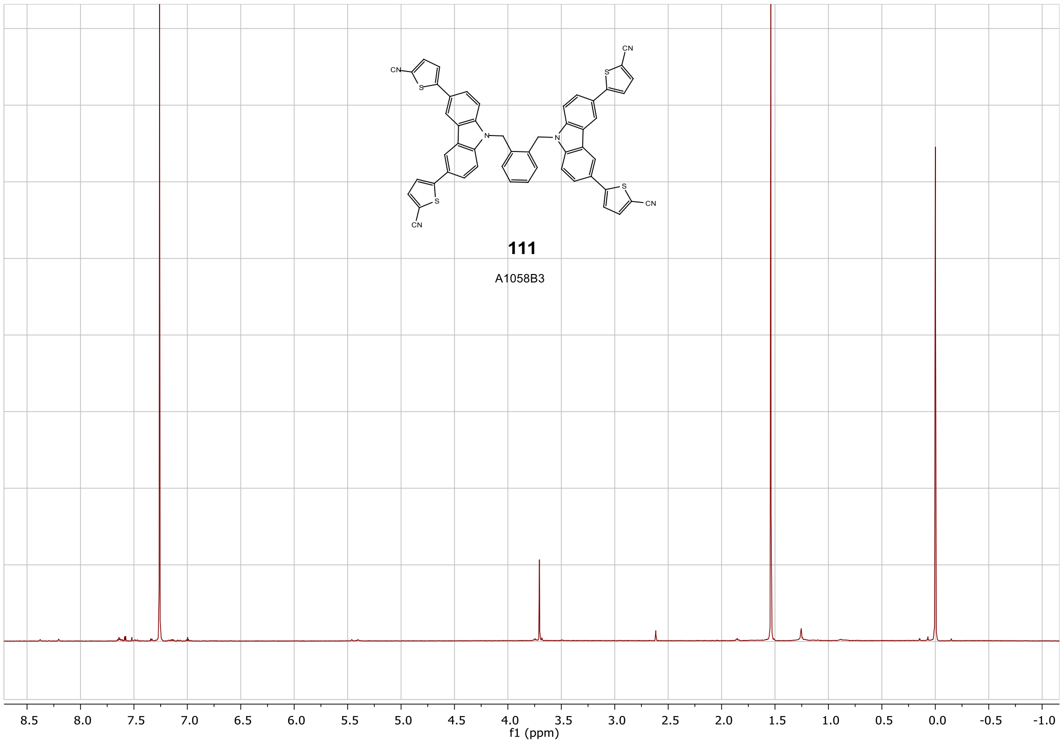

Supplementary Figure 155. ^{1}H NMR of **compound 111 (A1058B3)** in $CDCl_3$.

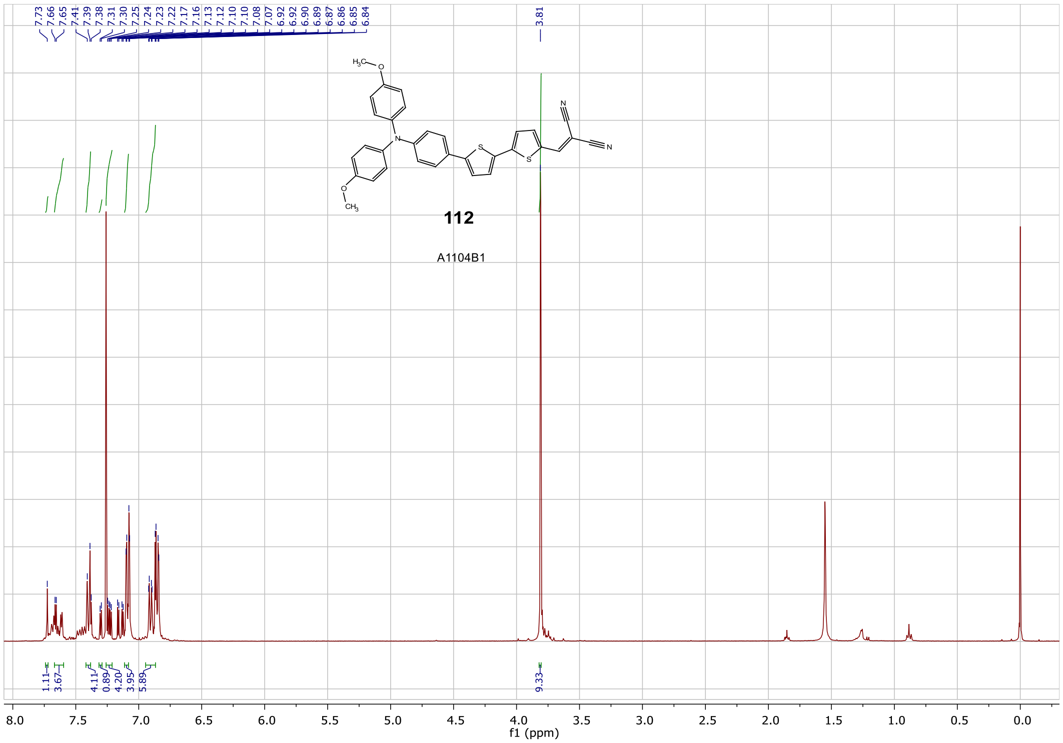

Supplementary Figure 156. ^{1}H NMR of **compound 112 (A1104B1)** in $CDCl_3$.

^{1}H NMR (400 MHz, Chloroform-*d*) δ 7.73 (s, 1H), 7.66 (d, *J* = 4.0 Hz, 4H), 7.40 (d, *J* = 8.5 Hz, 4H), 7.30 (d, *J* = 3.9 Hz, 1H), 7.23 (dd, *J* = 7.6, 4.1 Hz, 4H), 7.10 (d, *J* = 2.2 Hz, 4H), 6.95 – 6.87 (m, 6H), 3.81 (s, 9H).

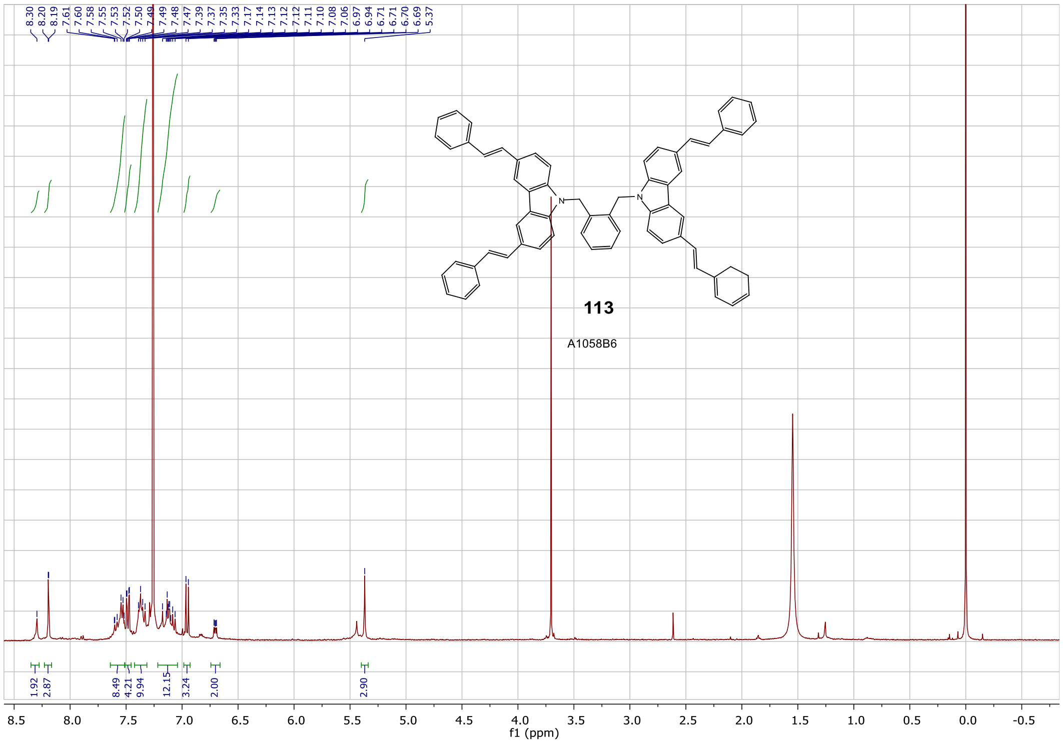

Supplementary Figure 157. ^{1}H NMR of **compound 113 (A1058B6)** in $CDCl_3$.

^{1}H NMR (400 MHz, Chloroform-*d*) δ 8.30 (s, 2H), 8.19 (d, *J* = 1.8 Hz, 3H), 7.64 – 7.51 (m, 8H), 7.51 – 7.46 (m, 4H), 7.43 – 7.31 (m, 10H), 7.22 – 7.04 (m, 12H), 6.95 (d, *J* = 8.7 Hz, 3H), 6.74 – 6.66 (m, 2H), 5.37 (s, 3H).

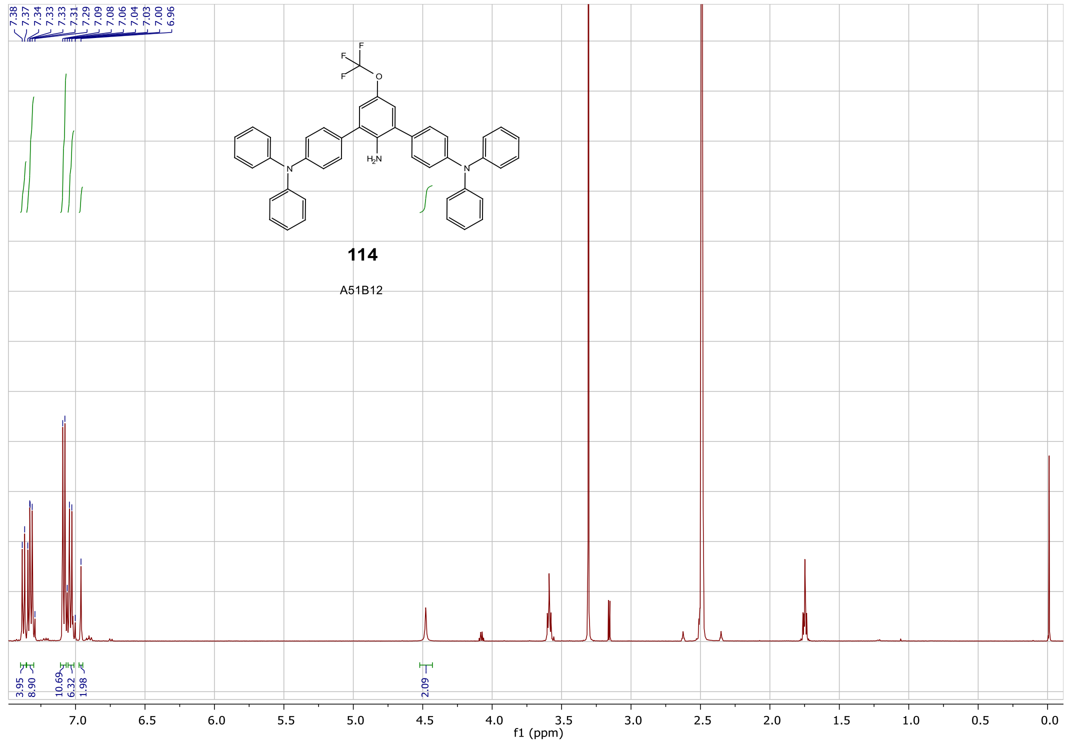

Supplementary Figure 158. ^{1}H NMR of **compound 114 (A51B12)** in $CDCl_3$.

^{1}H NMR (500 MHz, DMSO-d_6) δ 7.38 (d, J = 8.7 Hz, 4H), 7.35 – 7.30 (m, 9H), 7.08 (d, J = 7.8 Hz, 11H), 7.04 (d, J = 8.7 Hz, 6H), 6.96 (s, 2H), 4.48 (s, 2H).

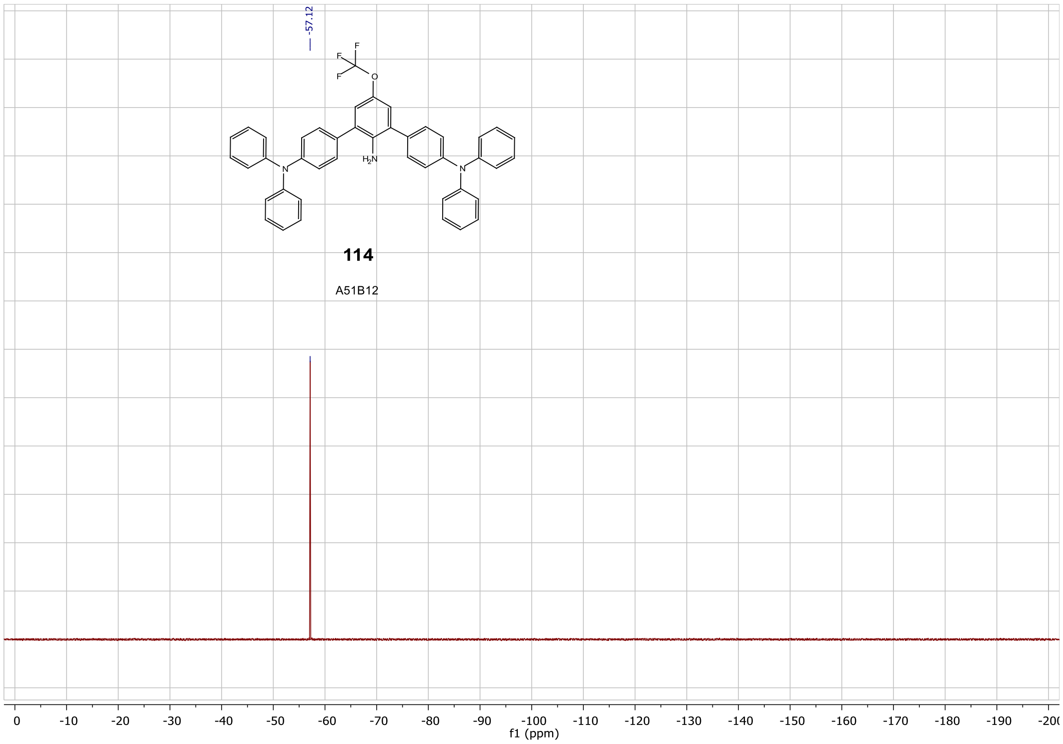

Supplementary Figure 159. ^{19}F NMR of **compound 114 (A51B12)** in $CDCl_3$.

^{19}F NMR (471 MHz, DMSO-d_6) δ -57.12.

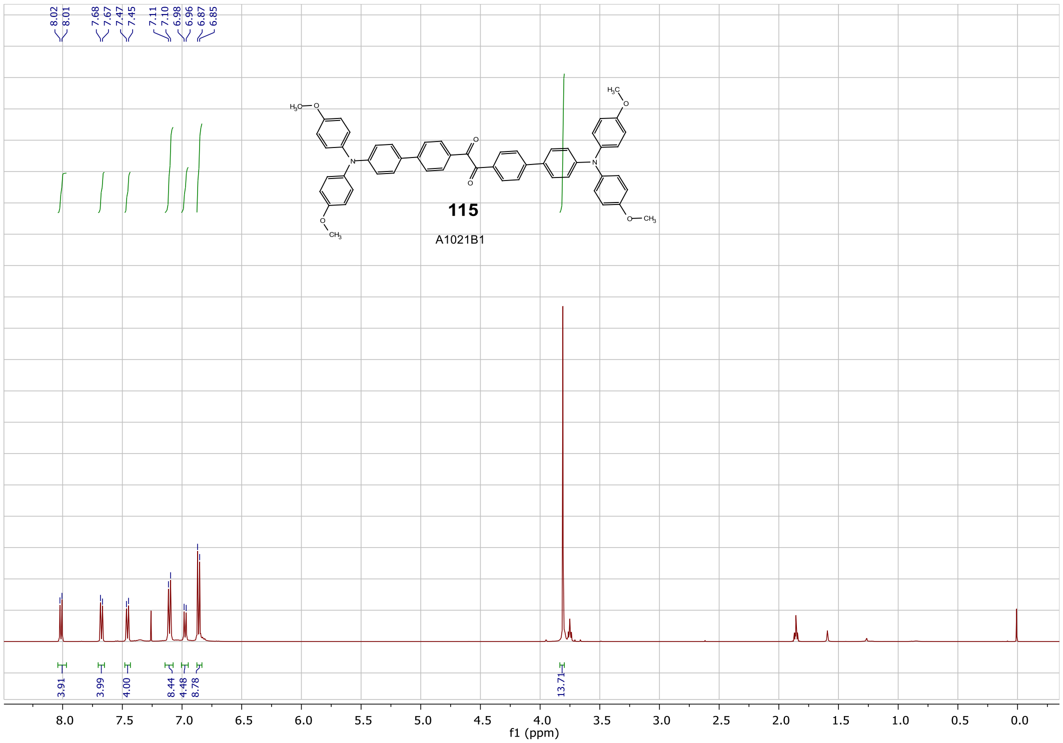

Supplementary Figure 160. ^{1}H NMR of **compound 115 (A1021B1)** in $CDCl_3$.

^{1}H NMR (500 MHz, Chloroform-*d*) δ 8.01 (d, *J* = 8.5 Hz, 4H), 7.67 (d, *J* = 8.5 Hz, 4H), 7.46 (d, *J* = 8.8 Hz, 4H), 7.10 (d, *J* = 8.9 Hz, 8H), 6.97 (d, *J* = 8.8 Hz, 4H), 6.86 (d, *J* = 8.9 Hz, 9H), 3.81 (s, 14H).

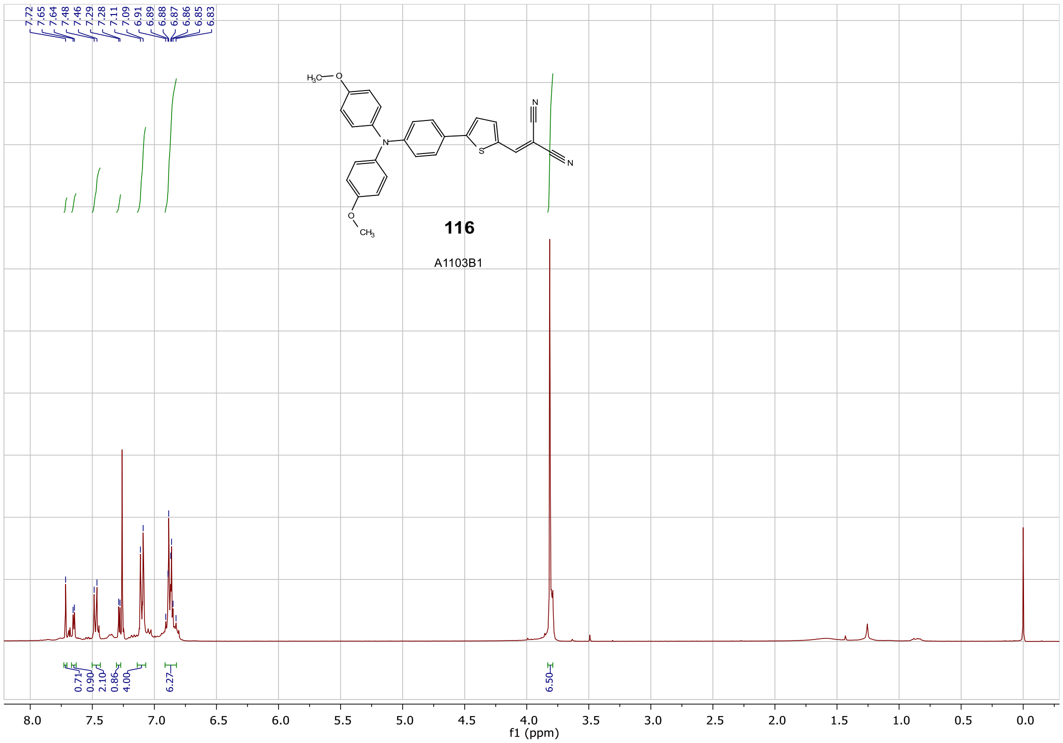

Supplementary Figure 161. ^{1}H NMR of **compound 116 (A1103B1)** in $CDCl_3$.

^{1}H NMR (400 MHz, Chloroform-*d*) δ 7.72 (s, 1H), 7.65 (d, *J* = 4.2 Hz, 1H), 7.47 (d, *J* = 8.8 Hz, 2H), 7.28 (d, *J* = 4.2 Hz, 1H), 7.10 (d, *J* = 8.9 Hz, 4H), 6.91 – 6.82 (m, 6H), 3.81 (d, *J* = 2.5 Hz, 6H).

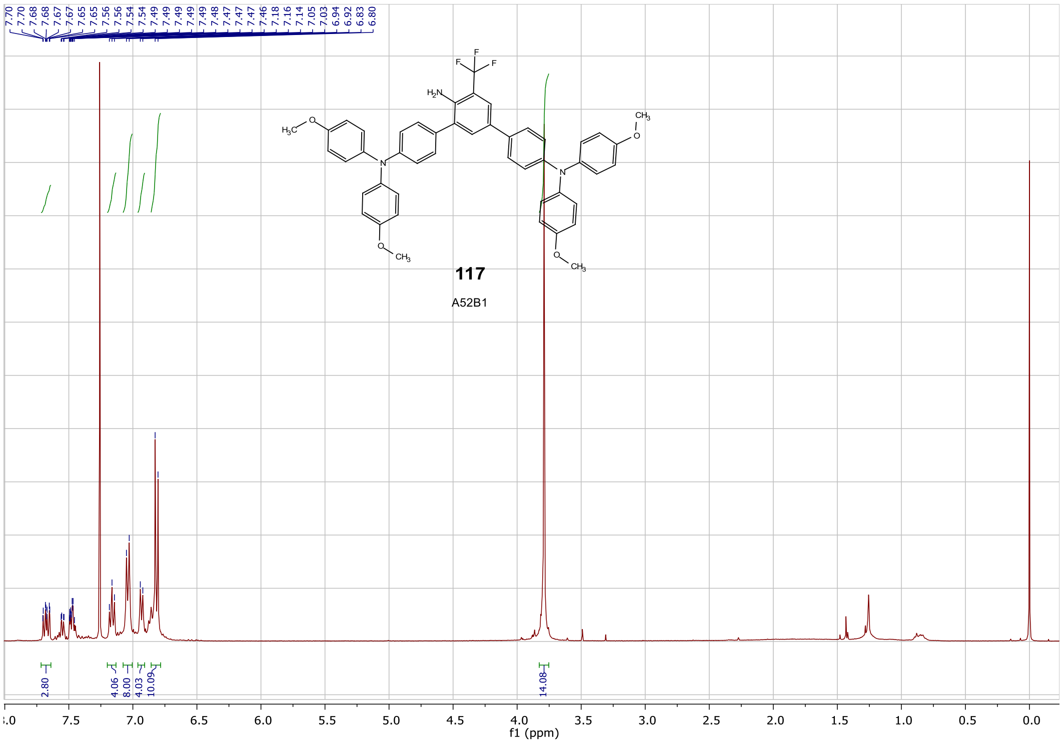

Supplementary Figure 162. ^{1}H NMR of **compound 117 (A52B1)** in $CDCl_3$.

^{1}H NMR (400 MHz, Chloroform-*d*) δ 7.72 – 7.64 (m, 3H), 7.16 (t, *J* = 7.9 Hz, 4H), 7.04 (d, *J* = 8.7 Hz, 8H), 6.93 (d, *J* = 7.8 Hz, 4H), 6.81 (d, *J* = 9.0 Hz, 10H), 3.79 (s, 14H).

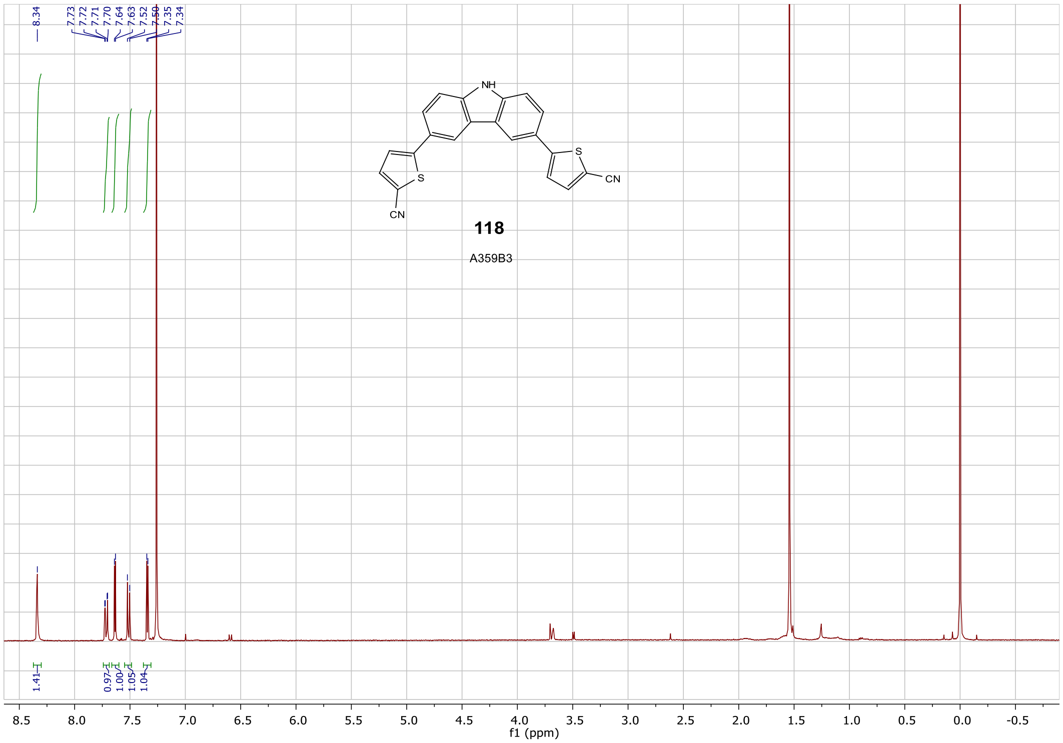

Supplementary Figure 163. ^{1}H NMR of **compound 118 (A359B3)** in $CDCl_3$.

^{1}H NMR (400 MHz, Chloroform-*d*) δ 8.34 (s, 1H), 7.74 – 7.69 (m, 1H), 7.64 (d, *J* = 3.9 Hz, 1H), 7.51 (d, *J* = 8.4 Hz, 1H), 7.34 (d, *J* = 3.9 Hz, 1H).

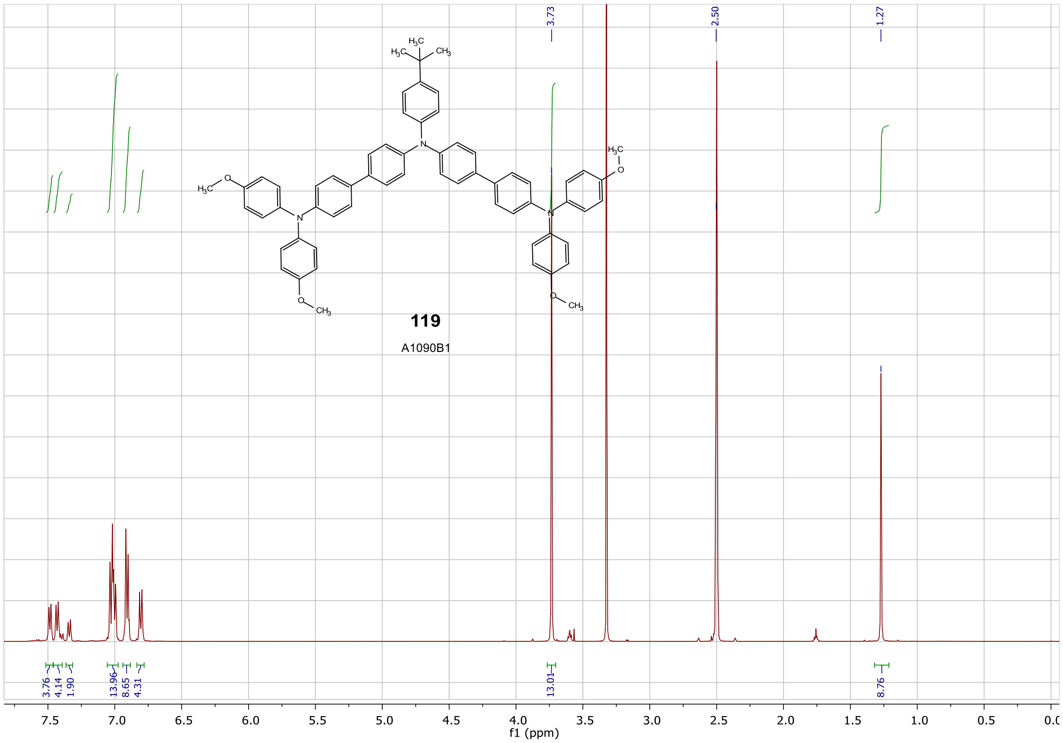

Supplementary Figure 164. ^{1}H NMR of **compound 119 (A1090B1)** in $CDCl_3$.

^{1}H NMR (500 MHz, DMSO-d_6) δ 7.48 (d, *J* = 7.9 Hz, 4H), 7.43 (d, *J* = 8.0 Hz, 4H), 7.34 (d, *J* = 7.9 Hz, 2H), 7.06 – 6.97 (m, 14H), 6.91 (d, *J* = 8.8 Hz, 9H), 6.81 (d, *J* = 8.5 Hz, 4H), 3.73 (d, *J* = 1.0 Hz, 13H), 1.27 (d, *J* = 1.1 Hz, 9H).

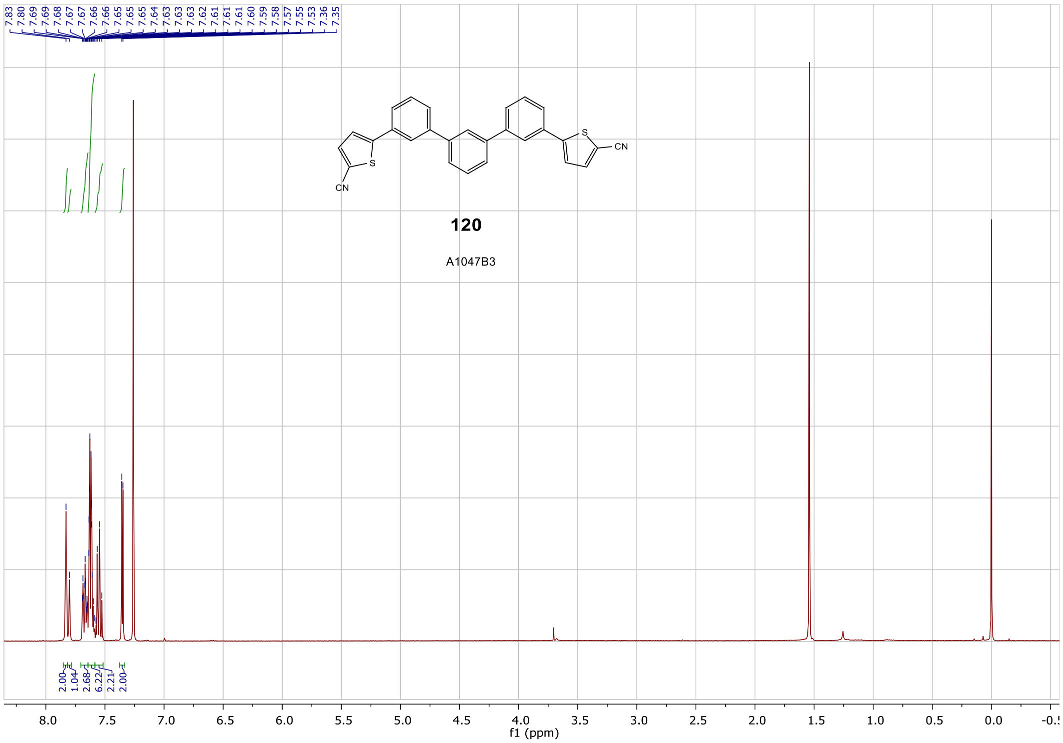

Supplementary Figure 165. ^{1}H NMR of **compound 120 (A1047B3)** in $CDCl_3$.

^{1}H NMR (400 MHz, Chloroform-*d*) δ 7.83 (s, 2H), 7.80 (s, 1H), 7.70 – 7.64 (m, 3H), 7.64 – 7.59 (m, 6H), 7.55 (t, *J* = 7.7 Hz, 2H), 7.35 (d, *J* = 3.9 Hz, 2H).

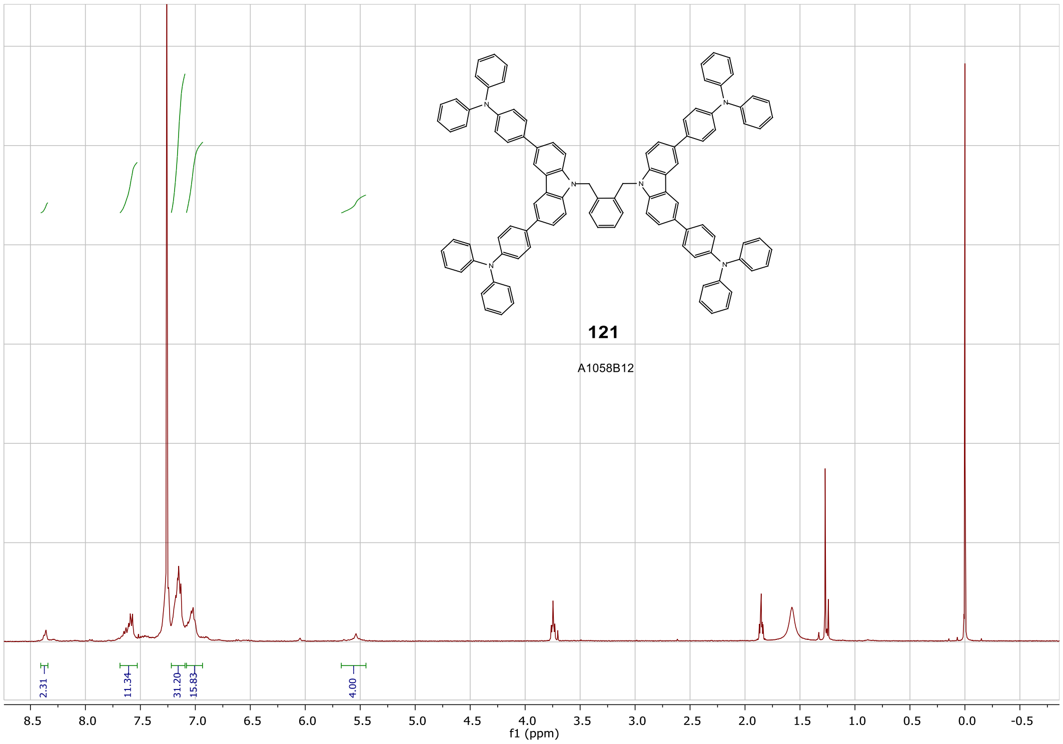

Supplementary Figure 166. ^{1}H NMR of **compound 121 (A1058B12)** in $CDCl_3$.

^{1}H NMR (400 MHz, Chloroform-*d*) δ 8.36 (s, 2H), 7.69 – 7.53 (m, 11H), 7.22 – 7.09 (m, 31H), 7.08 – 6.93 (m, 16H), 5.59 (s, 4H).

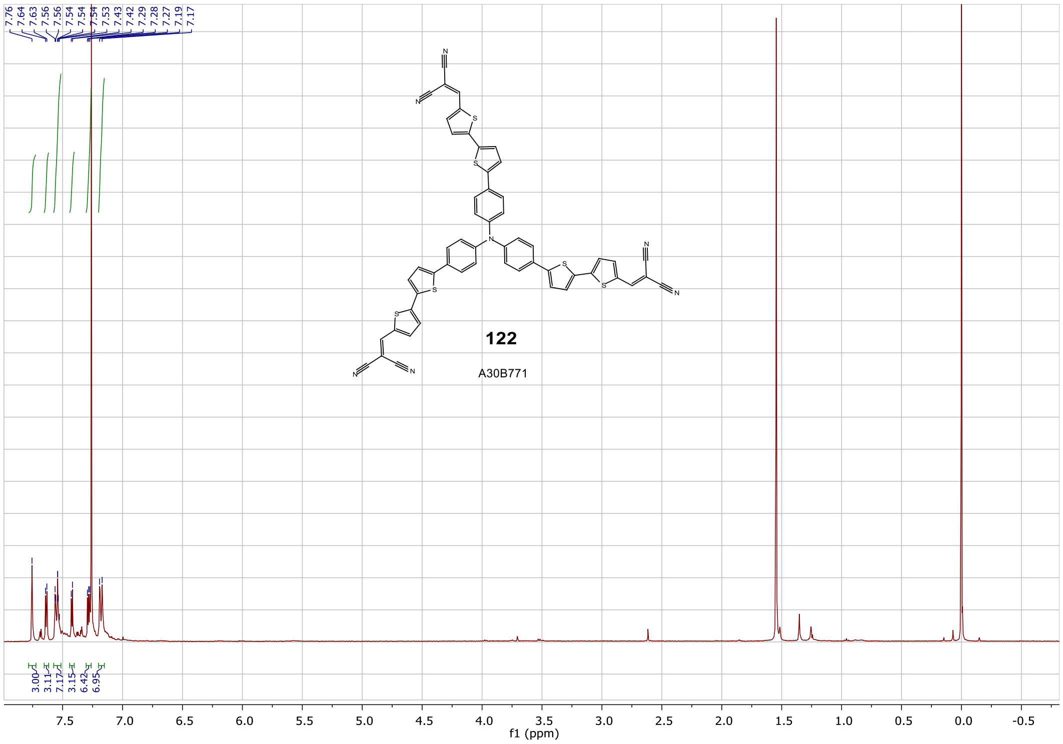

Supplementary Figure 167. ^{1}H NMR of **compound 122 (A30B771)** in $CDCl_3$.

^{1}H NMR (400 MHz, Chloroform-*d*) δ 7.76 (s, 3H), 7.64 (d, *J* = 4.2 Hz, 3H), 7.57 – 7.51 (m, 7H), 7.42 (d, *J* = 3.9 Hz, 3H), 7.30 – 7.26 (m, 6H), 7.18 (d, *J* = 8.6 Hz, 7H).

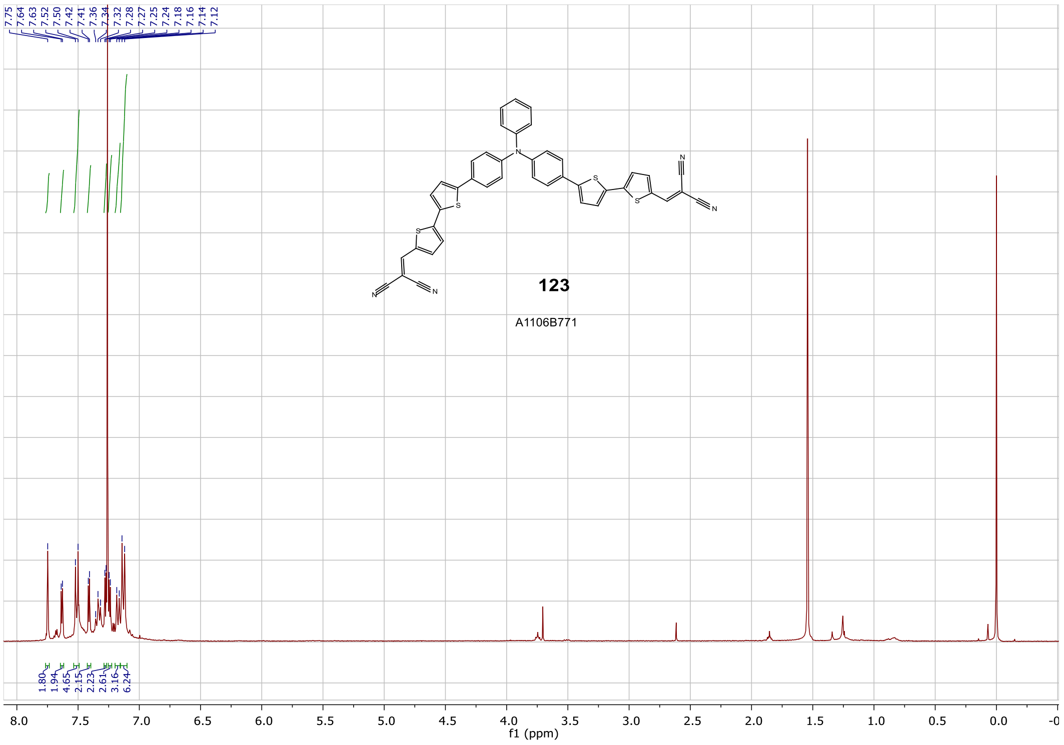

Supplementary Figure 168. ^{1}H NMR of **compound 123 (A1106B771)** in $CDCl_3$.

^{1}H NMR (400 MHz, Chloroform-*d*) δ 7.75 (s, 2H), 7.63 (d, *J* = 4.2 Hz, 2H), 7.51 (d, *J* = 8.7 Hz, 5H), 7.41 (d, *J* = 3.9 Hz, 2H), 7.28 (d, *J* = 4.1 Hz, 2H), 7.24 (d, *J* = 3.9 Hz, 3H), 7.17 (d, *J* = 8.2 Hz, 3H), 7.13 (d, *J* = 8.7 Hz, 6H).

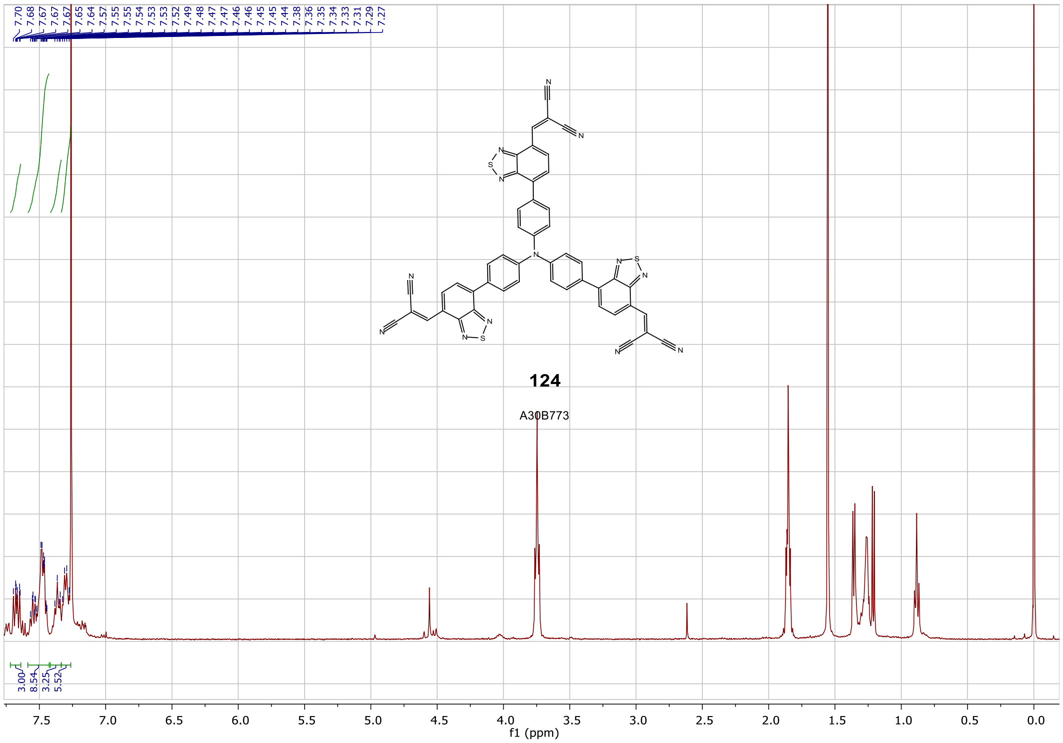

Supplementary Figure 169. ^{1}H NMR of **compound 124 (A30B773)** in $CDCl_3$.

^{1}H NMR (400 MHz, Chloroform-*d*) δ 7.72 – 7.64 (m, 3H), 7.59 – 7.43 (m, 9H), 7.42 – 7.33 (m, 3H), 7.33 – 7.26 (m, 6H).

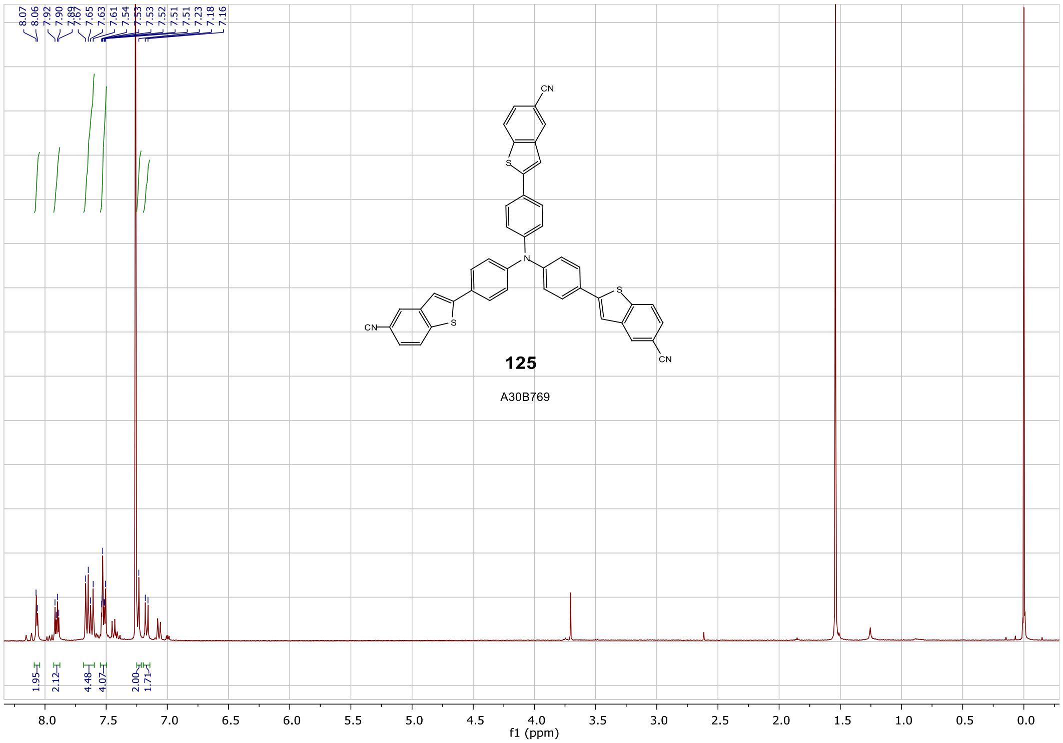

Supplementary Figure 170. ^{1}H NMR of **compound 125 (A30B769)** in $CDCl_3$.

^{1}H NMR (400 MHz, Chloroform-*d*) δ 8.07 (d, *J* = 4.2 Hz, 2H), 7.93 – 7.88 (m, 2H), 7.68 – 7.60 (m, 4H), 7.55 – 7.50 (m, 4H), 7.23 (s, 2H), 7.17 (d, *J* = 8.7 Hz, 2H).

References

1. Jain, A.; Ong, S. P.; Chen, W.; Medasani, B.; Qu, X.; Kocher, M.; Brafman, M.; Petretto, G.; Rignanese, G.-M.; Hautier, G.; Gunter, D.; Persson, K. A., FireWorks: a dynamic workflow system designed for high-throughput applications. *Concurrency Computat.: Pract. Exper.* **2015,** *27*, 5037-5059.
2. *Landrum, G. RDKit: Open-source cheminformatics software* **2016**.
3. Pracht, P.; Bohle, F.; Grimme, S., Automated exploration of the low-energy chemical space with fast quantum chemical methods. *Phys. Chem. Chem. Phys.* **2020,** *22*, 7169-7192.
4. Bannwarth, C.; Caldeweyher, E.; Ehlert, S.; Hansen, A.; Pracht, P.; Seibert, J.; Spicher, S.; Grimme, S., Extended tight-binding quantum chemistry methods. *WIREs Comput. Mol. Sci.* **2021,** *11*, e1493.
5. Balasubramani, S. G.; Chen, G. P.; Coriani, S.; Diedenhofen, M.; Frank, M. S.; Franzke, Y. J.; Furche, F.; Grotjahn, R.; Harding, M. E.; Hättig, C.; Hellweg, A.; Helmich-Paris, B.; Holzer, C.; Huniar, U.; Kaupp, M.; Marefat Khah, A.; Karbalaei Khani, S.; Müller, T.; Mack, F.; Nguyen, B. D.; Parker, S. M.; Perlt, E.; Rappoport, D.; Reiter, K.; Roy, S.; Rückert, M.; Schmitz, G.; Sierka, M.; Tapavicza, E.; Tew, D. P.; van Wüllen, C.; Voora, V. K.; Weigend, F.; Wodyński, A.; Yu, J. M., TURBOMOLE: Modular program suite for ab initio quantum-chemical and condensed-matter simulations. *J. Chem. Phys.* **2020,** *152*, 184107.
